# Structural Changes in the Carbon Sphere of a Dirhodium Complex Induced by Redox or Deprotonation Reactions

**DOI:** 10.1002/advs.202400072

**Published:** 2024-03-23

**Authors:** Clara Schweinzer, Peter Coburger, Hansjörg Grützmacher

**Affiliations:** ^1^ Department of Chemistry and Applied Biosciences ETH Zurich Vladimir‐Prelog‐Weg 1 Zurich 8093 Switzerland; ^2^ Department of Chemistry TU Munich Lichtenbergstrasse 4 85748 Garching bei München Germany; ^3^ LIFM IGCME School of Chemistry Sun Yat‐Sen University Guangzhou 510006 China

**Keywords:** alkynyl complexes, dinuclear complexes, low‐valent metals, metal‐metal bonds, rearrangements, redox chemistry, rhodium

## Abstract

A carbon‐rich molecule is synthesized, which mainly contains conjugated sp^2^ and sp hybridized carbon centers. Alkenyl and alkynyl binding sites are arranged such that this compound serves as ligand to a binuclear metal unit with a Rh^I^─Rh^I^ bond. Furthermore, CH units are placed in proximity to the metal centers. The dicationic complex [Rh_2_(bipy)_2_{Ph_2_Ptrop^(C≡CCy)2^}]^2+^(OTf^−^)_2_ allows to study possible responses of the carbon‐framework to redox reactions as well as deprotonation reactions. All products are, whenever possible, characterized by X‐ray diffraction (XRD) methods, NMR and EPR spectroscopy as well as electrochemical methods. It is shown that the carbon skeleton of the ligand framework undergoes C─C bond rearrangement reactions of remarkable diversity. In combination with DFT (density functional theory) studies, these results allow to gain insight into the electronic structure changes caused by metal sites in a carbon‐rich environment, which may be of relevance for the properties of metal particles on carbon support materials when they are exposed to hydrogen, electrons, or protons.

## Graphical abstract

A carbon rich ligand is synthesized, which binds to a dinuclear rhodium^I^ unit with a metal‐metal bond. The charge of the dicationic dirhodium complex is reduced by either adding electrons or removing protons. The carbon skeleton of the ligand framework undergoes C─C bond rearrangement reactions. Ring expansion is observed as well as alkyne to allenyl isomerization.

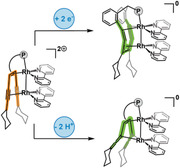

## 1 Introduction

**Figure 1:**
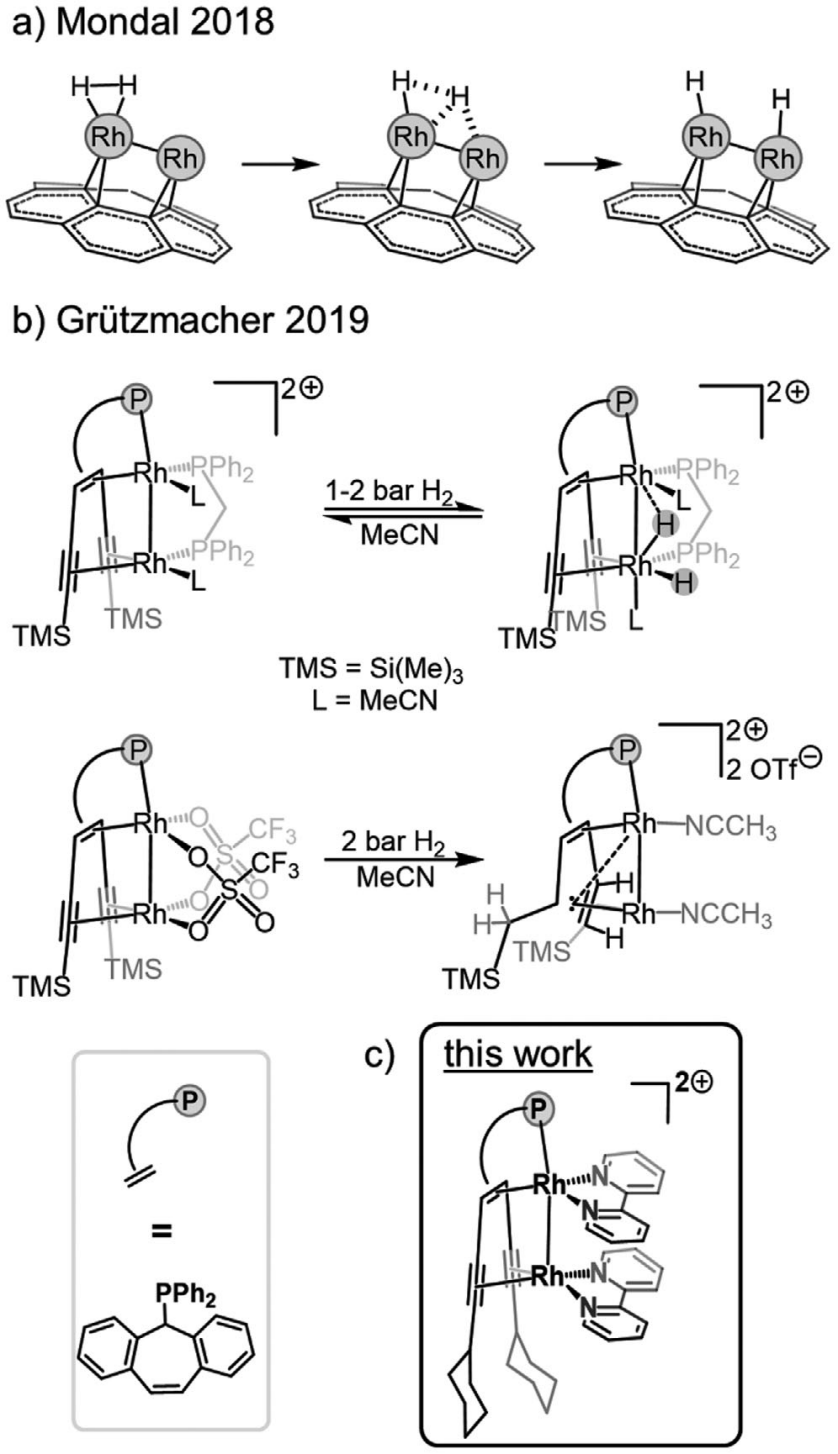
a) DFT studies of H_2_ splitting on a heterogenous small rhodium cluster and carbon support. b) H_2_ activation by dinuclear, molecular dirhodium complexes. c) Compound investigated in this work.

The investigation of redox induced changes of the properties and reactivity of transition metal complexes belongs to one of the core disciplines of organometallic chemistry^[^ ^1^, ^2^, ^3^, ^4^, ^5^, ^6^ ^]^ and finds applications ranging from synthetic chemistry to the development of molecular machines.^[^ ^7^, ^8^ ^]^ Classic and especially well studied examples are “ring‐slippage” rearrangements (often a change from the η^6^ to η^4^ or η^2^ bonding mode), which occur when (poly)arene complexes are subjected to redox reactions.^[^ ^3^, ^9^, ^10^, ^11^, ^12^ ^]^ Mostly mononuclear complexes were investigated but these phenomena were also observed with polynuclear complexes.^[^ ^13^, ^14^, ^15^ ^]^ Relevant to this work are also redox promoted C─C coupling reactions of metal vinylidene or alkynyl complexes.^[^ ^5^, ^6^, ^16^ ^]^ The fundamental organometallic reaction steps may also be of relevance for the chemistry of metal atoms or small particles deposited onto carbon support materials such as carbon black, activated carbon, and glassy carbon.^[^ ^17^, ^18^, ^19^ ^]^ The detailed structure in these materials is not clearly defined and a mixture of different C─C bond types and C*_n_* rings of different sizes with *n* = 5, 6, and 7 are encountered.^[^ ^20^, ^21^, ^22^ ^]^ How does the carbon material respond to chemical processes occurring at the metal centers? An answer to this question is difficult to give. A recent DFT (density functional theory) study investigated possible interactions between small rhodium clusters, (Rh)*_n_*, and a carbon support (**Figure** **1**a).^[^ ^23^ ^]^ It was found that the presence of the metal modifies the hybridization at the carbon centers and, indeed, the ring size which (Rh)*_n_* is coordinated to plays a role for the efficiency of in H_2_ activation.

We recently synthesized ligands containing a central 5*H*‐dibenzo[*a,d*]cyclohepten‐5‐yl (trop) unit to which a diphenylphosphanyl group and two alkynyl units are attached (Ph_2_Ptrop^(C≡CR)2^, R = TMS, Ph; TMS = SiMe_3_).^[^ ^24^, ^25^ ^]^ The overall concave structure of this compound and its electron accepting properties provided by the conjugated R─C≡C─C═C_trop_─C≡C─R unit allows to bind two low‐valent metal centers in close proximity. When the homobimetallic low‐valent dirhodium complex [Rh_2_{Ph_2_Ptrop^(C≡CS)2^}(L)_2_(dppm)]^2+^ (OTf^−^)_2_ (L = MeCN, dppm = diphenylphosphinomethane) was exposed to dihydrogen gas, reversible addition of H_2_ to the Rh_2_ unit was observed. A compound could be identified, which closely resembles the ones proposed by DFT for small Rh clusters units deposited on carbon (Figure 1b). On the other hand, the complex [Rh_2_{Ph_2_Ptrop^(C≡CS)2^}(OTf)_2_] reacted irreversibly with two equivalents of H_2_ hydrogen, which were transferred to carbon centers within the ligand skeleton.

In this study, we prepared a slightly modified Ph_2_Ptrop^(C≡CR)2^‐type ligand with R = Cy (cyclohexyl), which contains CH groups in proximity to a dinuclear Rh_2_ core (Figure 1c).

Significant C─C bond rearrangements of the extended conjugated carbon ligand framework were observed upon chemical redox and deprotonation reactions. The electron transfer processes were investigated with electrochemical studies and DFT calculations.

## 2 Results and Discussion

**Figure 2:**
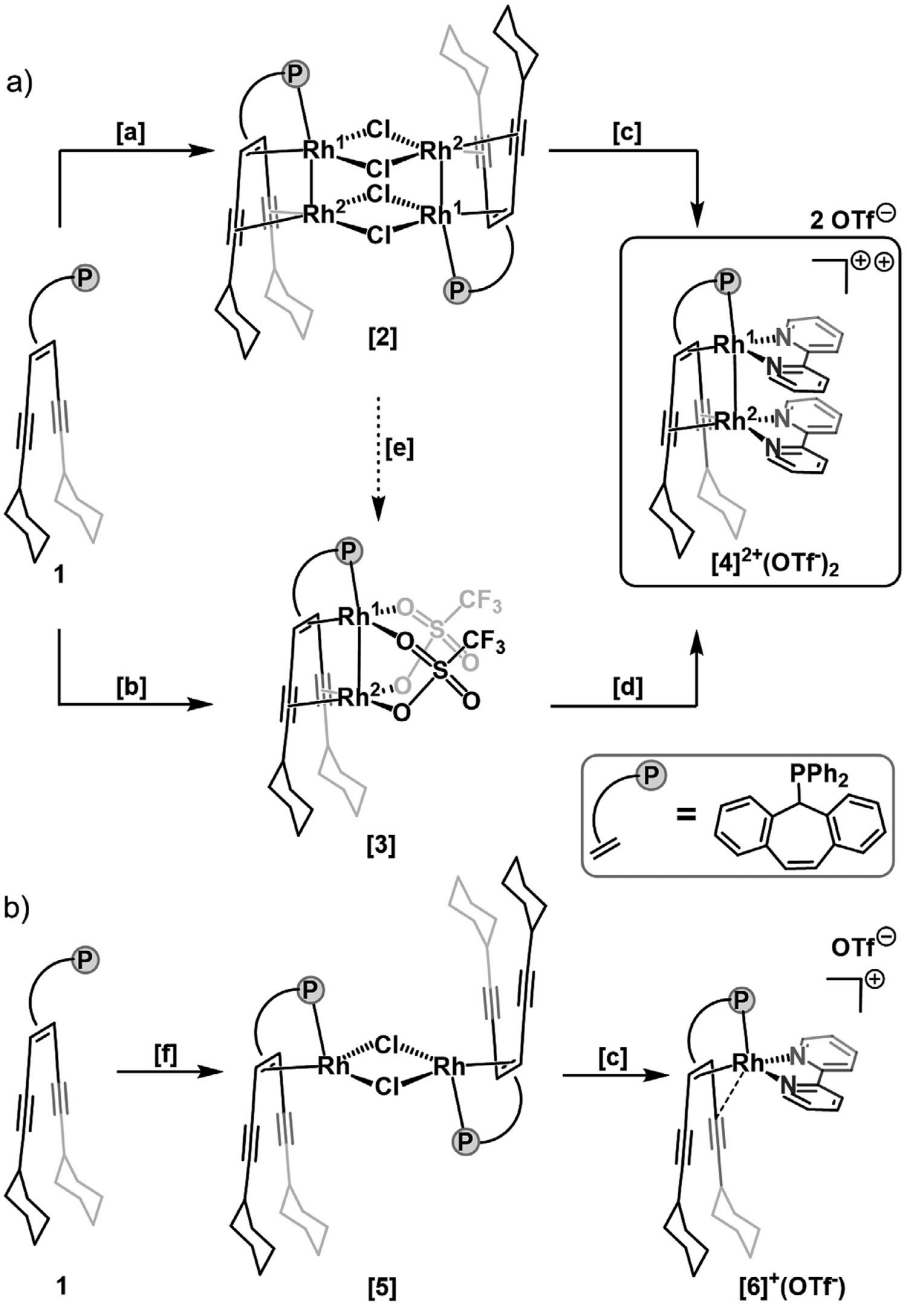
a) Synthetic routes to obtain complex **[4]** ^2+^(OTf^−^)_2_. b) Synthesis of complex **[6]** ^+^(OTf^−^). Conditions: [a] [Rh_2_(µ_2_‐Cl)_2_(COE)_4_], benzene, 18 h; [b] [Rh_2_ (COE)_4_(µ_2_‐OTf)_2_], THF, 2 h; [c] KOTf, bipy, THF, 4 h; [d] bipy, THF, 1 h; [e] AgOTf, DCM, 4 h; [f] [Rh_2_(µ_2_‐Cl)_2_(H_2_C = CH_2_)_4_], benzene, 18 h.

The ligand Ph_2_Ptrop^(C≡CCy)2^, **1** (Cy = cyclohexyl) was prepared as previously reported using a slightly modified procedure (see the ESI for details and^[^ ^24^, ^25^ ^]^). The dicationic, dinuclear Rh^I^ bis(2,2′‐bipyridine) (bipy) complex [Rh_2_(bipy)_2_{Ph_2_Ptrop^(C≡CCy)2^}]^2+^ (OTf^−^)_2_, **[4]** ^2+^(OTf^−^)_2_, can be synthesized by combining [Rh_2_(µ_2_‐Cl)_2_(COE)_4_] (COE = cyclooctene) with **1** to first form the chloride‐bridged tetranuclear complex **[2]**. Upon reaction of **[2]** with bipy and potassium triflate (KOTf) the dinuclear complex **[4]** ^2+^(OTf^−^)_2_ is formed (see **Figure** **2**a). Alternatively, [Rh_2_(COE)_4_(*µ*_2_‐κO,κO’‐OTf)_2_] can be used to form complex **[3]**, which reacts to complex **[4]** ^2+^(OTf^−^)_2_ simply by adding bipy. The molecular structures of all compounds were confirmed by X‐ray diffraction (XRD) methods using single crystals; a plot of the structure of **[4]** ^2+^(OTf^−^)_2_ is shown in Figure 4a (for plots of the other structures see the SI and CCDC depository).

In the dinuclear complexes **[2]**, **[3]**, and **[4]** ^2+^ the Rh^1^─Rh^2^ unit is tightly embedded in the ligand framework and especially Rh^1^ interacts strongly with the olefinic C1═C2 unit of the ligand. This interaction is reflected in short distances between Rh^1^ and the centroid (ct1) of the C1═C2 (C═C_trop_) bond (average 1.923 Å). The C1═C2 bond distance is elongated from 1.366 Å in **1** to ≈1.503 Å (average) in complexes **[2]**–**[4]** ^2+^. Coordination of the alkynyl units C3≡C4/C6≡C7 (average 1.203 Å) to Rh^2^ likewise leads to an elongation of the C≡C bonds (average 1.243 Å) but to a much lesser extent than for C1═C2. All bimetallic complexes show Rh─Rh distances in between 2.63 and 2.80 Å (see **Table** **1**) that are in the range of single bonds^[^ ^26^, ^27^, ^28^, ^29^ ^]^ and have been previously observed in related dinuclear rhodium trop phosphane complexes.^[^ ^24^, ^25^ ^]^ The nature of the metal‐metal interaction can be explained in a simplified way as follows: Not taking the Rh─Rh interaction into account, the coordination sphere around Rh^1^ corresponds to a trigonal pyramid. This is an unusual and rare coordination sphere for a 16‐valence electron metal complex, which renders the metal center Lewis‐acidic (vide infra).^[^ ^30^ ^]^ On the contrary, Rh^2^ resides in a 16‐valence electron square‐planar environment (again not considering the Rh─Rh interaction), which makes the metal center Lewis‐basic. This is true for all complexes **[2]**–**[4]** ^2+^ and consequently, the Rh─Rh bonds in all complexes can be viewed as a dative Rh→Rh donor‐acceptor interaction. This assumption is fully supported by DFT calculations, where a molecular orbital was found representing this donor‐acceptor interaction (see Figure S57, Supporting Information). QTAIM (quantum theory of atoms in molecules) was used to locate a bond critical point (BCP) between Rh^1^ and Rh^2^ (for a contour map of the Laplacian of the electron density of complex **[4]** ^2+^ see Figure S56, Supporting Information) and the electron localization function (ELF) shows a value of 0.208, which indicates a rather weak, but observable Rh─Rh bond.

**Table 1:** Selected bond lengths [Å], ^31^P, ^103^Rh and ^13^C chemical shifts [ppm], coupling constants [Hz] and redox potentials [V].

	[6]^+^	[4]^2+^	[4]^•+^	[7]	[8]^+^	[9]^a)^
Rh^1^─Rh^2^	‐	2.7923(4)	2.7096(6)	2.7109(6)	2.6930(9)	2.693
C1‐C2	1.502(3)	1.512(6)	1.508(7)	‐	1.509(7)	1.461
Rh^1^─ct_(C1‐C2)_	1.93624(14)	1.9292(3)	1.9398(4)	2.076(6)^b)^	1.9454(2)	2.003
C3‐C4	1.213(4)	1.232(6)	1.211(8)	1.450(9)	1.298(15)	1.296
C6‐C7	1.210(4)	1.239(6)	1.185(9)	1.418(8)	1.278(9)	1.325
Rh^2^‐ct_(C3‐C4)_	‐	2.0925(3)	2.1660(5)	1.9937(4)	2.035(9)^c)^	2.056^c)^
Rh^2^‐ct_(C6‐C7)_	‐	2.0835(3)	2.1505(4)	1.9990(4)	2.0084(9)	2.013^d)^
^31^P	111.6	96.4	–^e)^	17.8	78.2	61.3
^103^Rh^1^	‐7086	‐7043	–^e)^	‐7168	‐7343	‐7606
^103^Rh^2^	‐	‐743	–^e)^	–^f)^	‐5373	‐6183
^1^ *J*_P‐Rh1_	225.4	192.8	‐^e)^	180.7	194.8	191.7
^2^ *J*_P‐Rh2_	‐	9.3	–^e)^	20.9	29.5	52.3
C1/C2	47.3	37.6	–^e)^	143.0	37.9	37.9
C6/C3	107.9	87.2	‐^e)^	72.2	87.1/109.6	114.6
C7/C4	78.7	78.4	–^e)^	118.1	85.3/184.0	184.4
E^1/2^_(red)_	‐1.63 ‐2.06^g)^	‐1.35 ‐1.61	–	–	‐1.89 ‐2.28	–
E^1/2^_(ox)_	0.53^g)^	0.57^g)^	–	‐1.01 ‐1.37	‐0.50	–

^a)^ Calculated bond lengths.

^b)^ Rh^1^─C1 bond length.

^c)^ Rh^2^─C3 bond length.

^d)^ Rh^2^─C6 bond length.

^e)^ Paramagnetic radical.

^f)^ Not detected.

^g)^ Peak potential at 100 mV s^−1^.

In order to support this assumption on the nature of the Rh─Rh bond, we prepared the mononuclear rhodium complex [Rh(bipy)(Ph_2_Ptrop^(C≡CCy)2^)]^+^ (OTf^−^), **[6]** ^+^(OTf^−^), by first reacting the labile precursor complex [Rh_2_(µ_2_‐Cl)_2_(H_2_C═CH_2_)_4_] with **1**. This gives the dinuclear complex **[5]** as product, which is subsequently split to the desired mononuclear complex **[6]** ^+^(OTf^−^) upon addition of KOTf and bipy (Figure 2b). Single crystals were grown and subjected to an XRD analysis. The solid‐state structure of the complex cation **[6]** ^+^ is shown in Figure 4b. Indeed, the coordination sphere around the Rh center in **[6]** ^+^ is best described as a slightly distorted trigonal pyramid (deviation of the Rh center from the best plane through C1,C2,N1,N2 = 0.252(2) Å). In comparison to the dinuclear dication **[4]** ^2+^, the Rh^1^─C distances in **[6]** ^+^ are similar (Rh─ct_(C1‐C2)_ 1.936 Å, C1═C2 1.504 Å). Noteworthy is the long interaction of 2.435(3) Å between Rh^1^ and one of the carbon centers, C3, of one of the alkynyl units reflecting the Lewis‐acidity of the 16 e Rh^I^ center in a trigonal pyramidal environment as mentioned above.

All compounds **[2]**–**[6]** ^+^ were fully characterized by NMR spectroscopy in solution and selected data are listed in Table 1. Compared to the uncoordinated molecule **1** (δ^31^P = −14.0 ppm), the complexes **[2]–[4]** ^2+^ show significantly high‐frequency shifted ^31^P NMR resonances at δ^31^P = 101.2, 106.0, and 96.4 ppm, respectively. The complexes **[5]** and **[6]** ^+^ show an even larger shift to δ^31^P = 113 and 112 ppm, respectively. Coupling of the ^31^P nucleus with both ^103^Rh nuclei in the dinuclear complexes leads to characteristic doublets of doublets with ^1^ *J*_RhP_ = 180–195 Hz and ^2^ *J*_RhP_ = 9–52 Hz (see Table 1; and Figure S15, Supporting Information for a spectrum of **[4]** ^2+^ as an example)). The ^13^C nuclei of the coordinated C═C_trop_ and C≡C─Cy units of all dinuclear complexes **[2]**, **[3]**, and **[4]** ^2+^ show resonances within a narrow window of 37.–38.9 ppm (C═C_trop_), and 83.5–87.2 (*C* ≡C─Cy) or 68.6–78.4 ppm (C≡ *C* ─Cy). Upon coordination, the resonances Δδ_av_ = δ_(ligand)_ – δ_(complex)_ shift to lower frequencies, which is especially pronounced for the C═C_trop_ unit which is shifted ≈92 ppm. The Δδ_av_ is much smaller for the alkyne units (17.3 to 14.2 ppm). These findings are fully in accord with the structural parameters discussed above, which show a strong Rh^1^ to C═C_trop_ interaction while the Rh^2^ alkynyl interactions are weaker. For the complexes **[5]** and **[6]** ^+^ with only one Rh^I^ center coordinated to **1**, the Δδ_av_(C═C_trop_) = 77.4 ppm is less pronounced. This underscores the assumption that in the dinuclear complexes Rh^2^ donates electron density to Rh^1^, which in turn donates more electron density back into the π*‐orbital of the C═C_trop_ unit. In the mononuclear complex **[6]** ^+^, only one set of ^13^C NMR resonances at δ^13^C(*C* ≡C─Cy) = 118.8 ppm and δ^13^C(C≡ *C* ─Cy) = 78.7 ppm is observed indicating that coordination of the alkyne unit is fluxional on the NMR time scale in solution. The ^103^Rh NMR shifts were determined indirectly by ^1^H‐^103^Rh HMBC (heteronuclear multiple bond correlation) and spectra for **[4]** ^2+^ are shown in Figures S16 and S17 (Supporting Information). The chemical shifts of Rh^1^ in complexes **[2]–[4]** ^2+^ range from −6724 to −7043 ppm [vs Rh(acac)_3_ (acac = acetylacetonate)] and correspond well to literature values.^[^ ^24^, ^31^, ^32^ ^]^ We could also determine the chemical shift for Rh^2^, which is observed at about δ^103^Rh = −700 ppm for complexes **[3]** and **[4]** ^2+^, but is significantly more negative for **[2]** at δ^103^Rh = −4736 ppm. To the best of our knowledge, these are the first reported data for Rh^I^ alkyne complexes.

**Figure 3:**
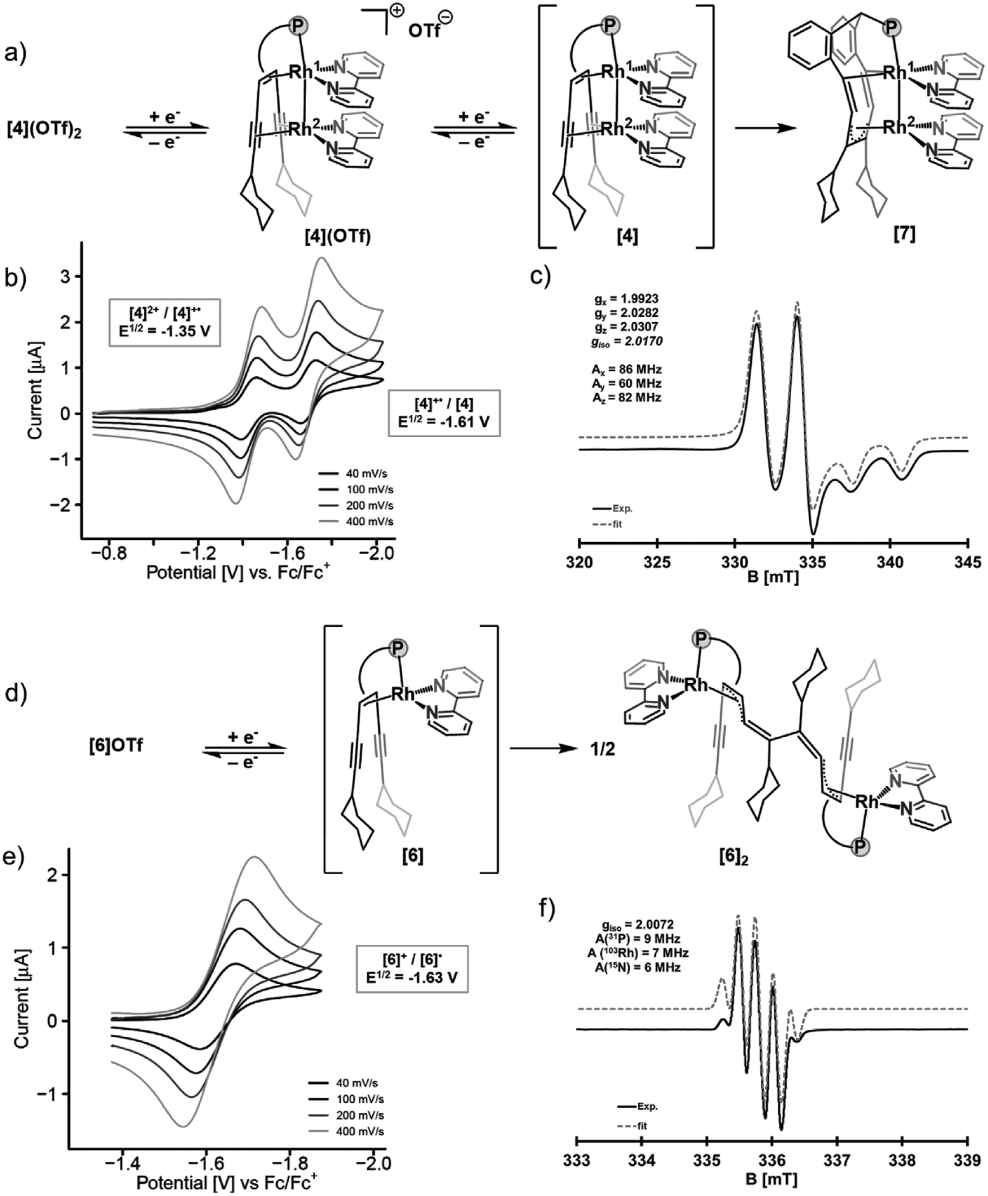
a) Reduction reaction of **[6]** ^+^ via **[6]** ^•^ to **[6]**_2_ with CoCp*_2_ as reductant in THF. b) Redox wave at E^½^ = −1.63 V (vs Fc/Fc^+^) of **[6]** ^+^ at different scan rates. Conditions: 1 mm analyte, 100 mm [*n*Bu_4_N]PF_6_ electrolyte, 1,2‐Dimethoxyethane (DME), working electrode (WE): glassy carbon (GC), counter electrode (CE): Pt on TiO_x_, reference electrode (RE): Ag/Ag^+^. c) EPR spectrum of **[6]** ^+^ (generated in situ from **[6]** ^+^ with one equivalent of CoCp*_2_ at room temperature in toluene/acetonitrile). d) Reduction of **[4]** ^2+^ via **[4]** ^•+^ and **[4]** to **[7]** with CoCp*_2_ as reductant in THF. e) Redox waves at E^½^ = −1.35 and E^½^ = −1.61 V (vs Fc/Fc^+^) of **[4]** ^2+^ at different scan rates. Conditions: 1 mm analyte, 100 mm [*n*Bu_4_N]PF_6_ electrolyte, THF, WE: Pt, CE: Pt on TiO_x_, RE: Ag/Ag^+^. f) EPR spectrum of **[4]** ^•+^ in frozen solution in toluene/acetonitrile.

**Figure 4:**
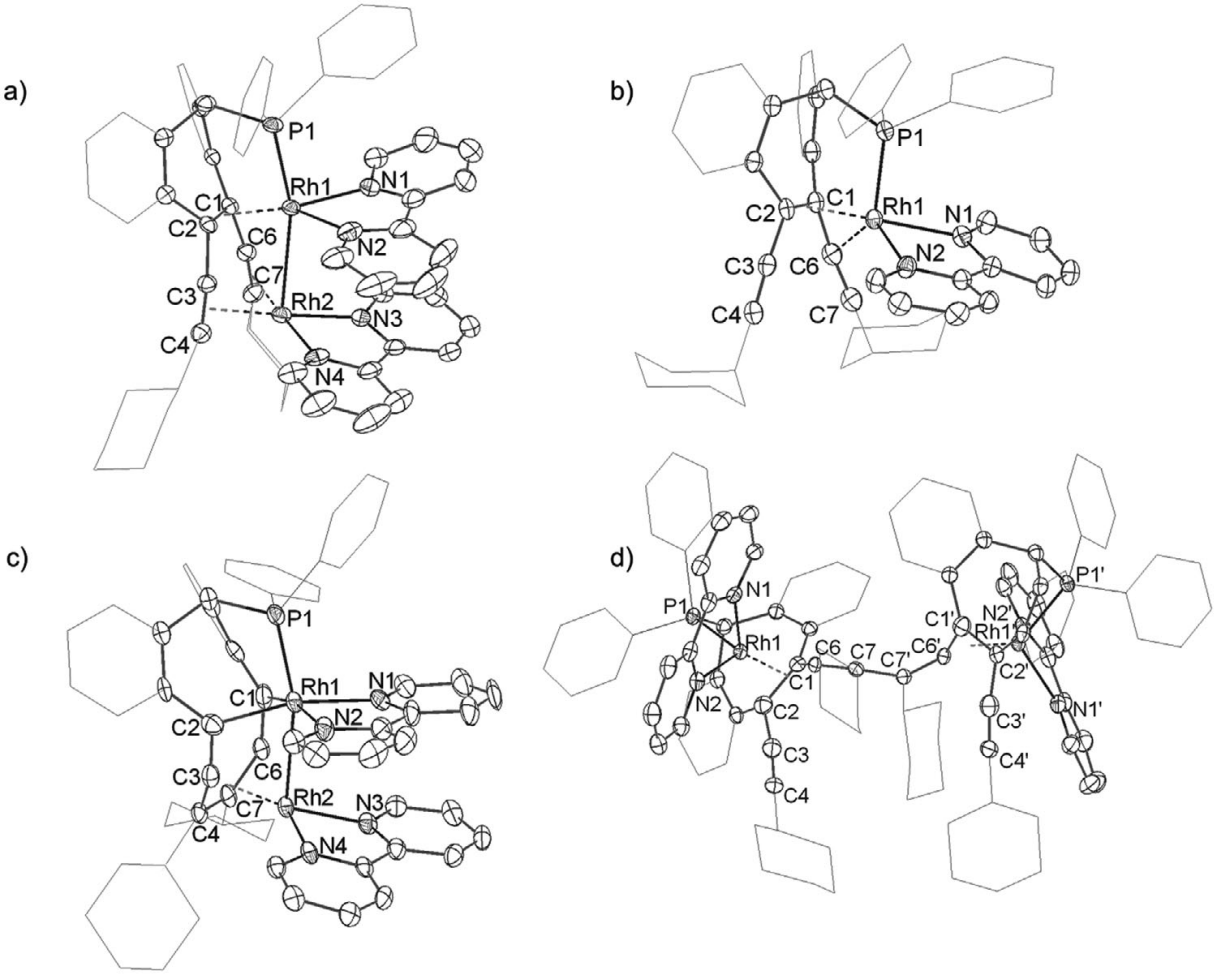
Solid state structures of a) **[4]** ^2+^, b) **[6]** ^+^, c) **[7]**, and d) **[6]**_2_. Hydrogen atoms, counter ions, and solvent molecules omitted for clarity.

The cyclic voltammogram (CV) of the mononuclear rhodium complex **[6]** ^+^ shows a reversible reduction at E^½^ = −1.63 V versus Fc/Fc^+^ (ferrocene/ferrocenium) (**Figure** **3**a,b), which is more negative than the ones in related trop phosphane rhodium(I) complexes (E^½^ = −1.00 and −1.19 V)^[^ ^33^ ^]^ and trop amine complexes (E^½^ = −1.27 to −1.46 V).^[^ ^34^ ^]^ A second, irreversible reduction wave is observed at E^p^ = −2.06 V, which exhibits a triangular peak shape resulting from an adsorption phenomenon of the generated species (see Figure S54, Supporting Information). The mono reduced neutral complex [Rh(bipy)(Ph_2_Ptrop^(C≡CCy)2^)]^•^, **[6]** ^•^, was generated in situ with CoCp*_2_ (decamethylcobaltocene) as chemical reductant and detected by EPR spectroscopy, shown in Figure 3c. With respect to **[6]** ^+^, DFT calculations show a slight change of the structure of **[6]** ^•^, which now shows a nearly perfect trigonal pyramid with a deviation of Rh from the trigonal plane 0.32 Å. The spin density of the unpaired electron is mostly located on the bipy ligand, only 22% are based on the rhodium center. This finding is in line with previous data of formally d^9^‐Rh° complexes with trop‐type ligands and distorted tetrahedral structures. Those likewise show relatively low spin densities of 24%–36% on the metal center and are best described as delocalized organometallic radicals.^[^ ^35^ ^]^ Although, small structural changes may lead to electromeric structures in which >80% of the spin density is located on the metal center.^[^ ^36^, ^37^ ^]^ Remarkably, when the reduction is performed on larger scale with the intention to isolate **[6]** ^•^, reaction between two such radicals is observed. C─C bond formation between two β‐alkyne‐C7 atoms gives exclusively the dimer **[6]**_2_ (see Figure 3a and a plot of the solid state structure in **Figure** **4**d). Comparable dimerization reactions have been detected in redox reactions of metal alkynyl complexes before.^[^ ^5^, ^6^, ^16^ ^]^

The CV of the dinuclear complex **[4]** ^2+^(OTf^−^)_2_ in THF (tetrahydrofuran) shows two reversible redox events at E^½^  = −1.35 and E^½^ = −1.61 V vs Fc/Fc^+^ (see Figure 3e), which are attributed to the processes **[4]** ^2+^ + e ⇆ **[4]** ^•+^ and **[4]** ^•+^ + e ⇆ **[4]** (Figure 3d). From the separation of the redox waves ΔΔE = 0.26 V, the equilibrium constant at room temperature is calculated to be *K*_disp_ = 4.0 × 10^−5^ for the disproportionation 2 × **[4]** ^•+^ ⇆ **[4]** ^2+^ + **[4]**, which should allow the observation and eventually even isolation of **[4]** ^•+^. Indeed, if complex **[4]** ^2+^ is treated with one equivalent of reductant (sodium naphthalenide in THF or CoCp*_2_ in *o*‐difluorobenzene (DFB)), the dark red paramagnetic complex **[4]** ^•+^(OTf^−^) is obtained. An XRD measurement using a single crystal allowed to determine the structure of **[4]** ^•+^(OTf^−^). Compared to **[4]** ^2+^, the Rh─Rh bond [2.7096(6) Å] is shortened while no significant differences in bond lengths were observed for the C═C_trop_ and C≡C bonds within the accuracy of the measurement (see Table 1). The radical cation **[4]** ^•+^ was further characterized by EPR spectroscopy. A rhombic g‐tensor with a small anisotropy and an isotropic g‐value (2.0170 vs 2.0023 of the free electron) indicates that the unpaired electron is mainly localized on the ligand scaffold with little spin density on the dinuclear Rh─Rh unit (Figure 3f). This is confirmed by DFT calculations, which show that the spin density is mainly located on the bipy ligand coordinated to Rh^2^ (bipy: 0.52 e, Rh^1^: 0.06, Rh^2^: 0.09 e) (see Figure S60, Supporting Information).

The use of two equivalents of a strong reductant leads to the formation of a poorly soluble, green complex **[7]** (λ_max_ = 696 nm). The solid‐state structure was determined by X‐ray crystallography, which surprisingly did not appear to be the one of the fully reduced complex **[4]**. Instead, the structure of isolated complex **[7]** shows a rearrangement of the carbon ligand framework (see Figure 4c): Cleavage of the C═C_trop_ double bond of the central seven membered ring occurred, where the C1─C2 distance increases from 1.512(6) to 2.564(9) Å. This is accompanied by the formation of a new C─C bond between the C4 and C7 carbon atoms of the former alkyne units (the C4‐C7 distance shrinks from 3.224(6) Å in **[4]** ^2+^ to 1.468(8) Å in **[7]**). As a result of this rearrangement, the dinuclear Rh─Rh unit (Rh^1^─Rh^2^ 2.7109(6) Å) is now bound to a curved eleven‐membered carbon‐cycle with benzo groups attached in 9,10 and 12,13 position and two cyclohexyl substituents in 4,7 positions. To the best of our knowledge, a comparable ring expansion has not been reported before.

All carbon centers are sp/sp^2^‐hybridized, apart from the sp^3^‐hybridized carbon center C11, which carries the PPh_2_ group at the bow of the eleven‐membered ring. A conjugated delocalized π‐electron system is formed in which the C─C distances vary between 1.315 Å (average C2–C3/C1–C6) and 1.470 Å (average C1–C9/C2–C13). The Rh^1^ center is bound to the phosphorus atom [2.2017(15) Å], Rh^2^ [Rh^1^─Rh^2^ = 2.7109(6) Å], the two carbon centers C1 [2.073(7) Å] and C2 [2.076(6) Å] and one bipy ligand. The other Rh^2^ center is coordinated to the second bipy ligand, Rh^1^, and to the C6‐C7‐C4‐C3 unit at the stern of the C_11_ ring (Rh^2^‐ct1 1.7332(4) Å; ct1 = centroid of C6‐C7‐C4‐C3). Formally, the ─C1 = C6 = C7(Cy)‐C4(Cy) = C3 = C2─ part of the C_11_ ring can therefore be described as dianionic bis(allenyl) to which Rh^1^ is bound via two Rh─C σ─bonds while Rh^2^ is coordinated in a η^4^‐fashion. This renders the C_11_ ring an eight‐electron donor ligand in total. Following the arguments brought to the fore in the description of **[4]** ^2+^ (vide supra), four electrons of the C_11_ ring are donated to Rh^1^, which together with the phosphanyl group (2 e) and the bipy ligand (4 e) reaches a valence electron configuration of 18. Rh^2^ retains its 16 valence electron count as discussed above. This description is supported by calculation of IBOs (intrinsic bond orbitals), which indeed show that the ligand carries two negative charges and both rhodium centers remain in the formal oxidation state of +1. A QTAIM calculation shows a bond critical point is located between Rh^1^ and Rh^2^, indicating a metal‐metal single bond. The contour map of the Laplacian of the electron density of complex **[7]** is shown in Figure S56 (Supporting Information). The electron density at the BCP in **[7]** (0.048) shows a slightly higher value compared to dication **[4]** ^2+^ (0.042) indicating a stronger interaction between the rhodium centers in the latter, which is in line with the shorter Rh─Rh distance observed in **[7]**. Furthermore, analysis of the energy parameters at the BCP indicates that the Rh─Rh interaction in both species is best described as a weak dative metal‐metal interaction.

**Figure 5:**
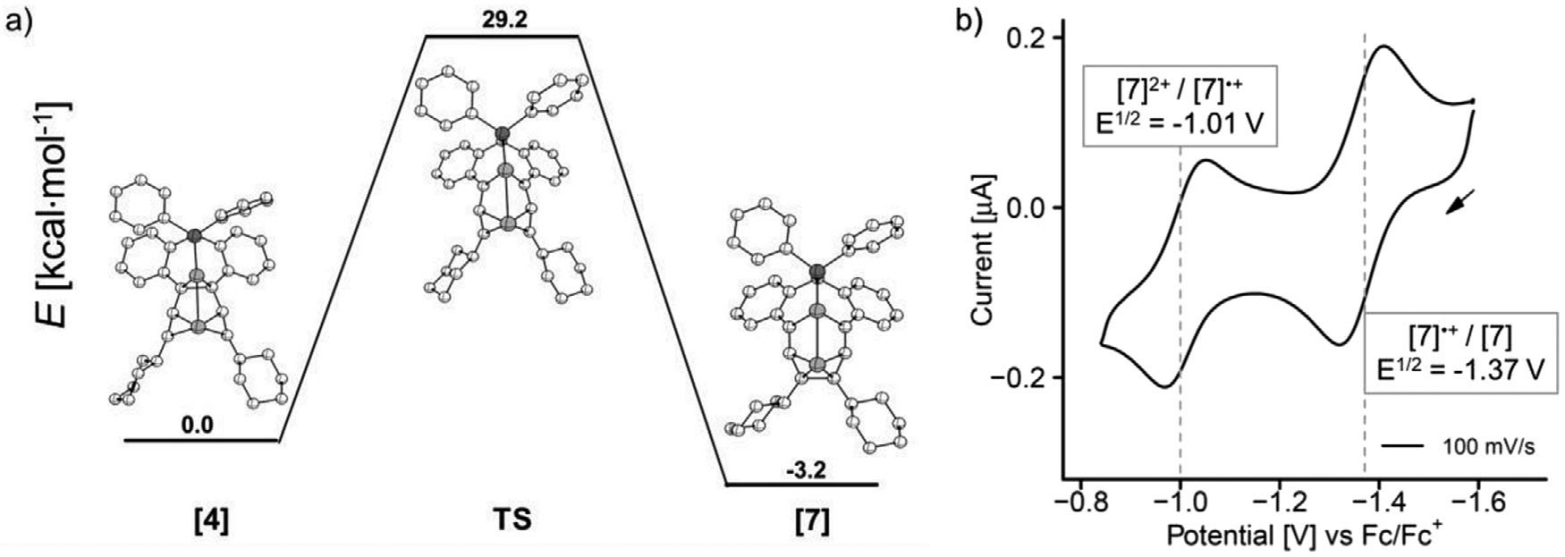
a) Minimum energy path for the interconversion between the native form of double reduced complex **[4]** to the rearranged complex **[7]** via the transition state **TS**. Calculated structures, bipy ligands and H atoms omitted for clarity. b) Redox waves at E^½^  = ‐1.37 and E^½^ = ‐1.01 V (vs Fc/Fc^+^) of **[7]** in THF, OCP and scan direction are shown by an arrow. Conditions: 1 mm analyte, 100 mm [*n*Bu_4_N]PF_6_ electrolyte, WE: Pt, CE: Pt on TiO_x_, RE: Ag/Ag^+^, 100 mV s^−1^ scan rate.

The conversion of the neutral fully reduced complex **[4]** to the rearranged product **[7]** was calculated by DFT methods (**Figure** **5**a). Complex **[7]** is 3.2 kcal mol^−1^ more stable than neutral **[4]**. The rearrangement proceeds smoothly via opening of the C1═C2 bond and concomitant closure of the C4–C7 distance via one activated complex **TS** at the transition state, which is 29.2 kcal mol^−1^ higher in energy than **[4]**. These results are in agreement with the fact that two reversible redox waves are observed in the CV of **[4]** ^2+^ indicating that **[4]** is sufficiently long‐lived on the time scale of the CV experiment, while it rearranges to **[7]** upon attempts of isolation. The ^31^P NMR chemical shifts of **[4]** and **[7]** were calculated by DFT methods (δ^31^P = 54 and 7 ppm, respectively). The latter is closer to the experimentally observed chemical shift of δ^31^P = 18.9 ppm and we therefore assume that **[7]** is the predominant species in solution.

A CV of the rearranged complex **[7]** was recorded and shows the expected two reduction events (Figure 5b): Starting from the open‐circuit‐potential (OCP) at −1.59 V, the complex can be oxidized at E½ = −1.37 and −1.01 V (both considered quasi‐reversible). Note that the potentials are shifted to less negative potentials when compared to the reductions of **[4]** ^2+^ (E½ = −1.35 and −1.61 V) indicating that **[7]** ^•+^ and **[7]** ^2+^ likely retain the overall structure of **[7]** although follow‐up reactions might occur after the second oxidation on the time scale of the CV experiment. The formation of side products was verified by ^31^P NMR spectroscopy (see Figure S42, Supporting Information), which shows that indeed **[4]** ^2+^ is partially regenerated upon chemical oxidation of **[7]**.

A bulk electrolysis experiment was performed in order to investigate whether the rearranged complex **[7]** can be prepared electrochemically. The electrosynthesis was set up in a divided cell with a Pt mesh working electrode and Zn‐wire counter electrode as sacrificial anode with a Ag/Ag^+^ reference electrode in the working compartment. During electrolysis, a dark green solid formed on the cathode. This solid product was separated from the reaction mixture and dissolved in pyridine. Indeed, NMR spectroscopic characterization revealed formation of complex **[7]**, which is hence an alternative and simple way of its preparation.

**Figure 6:**
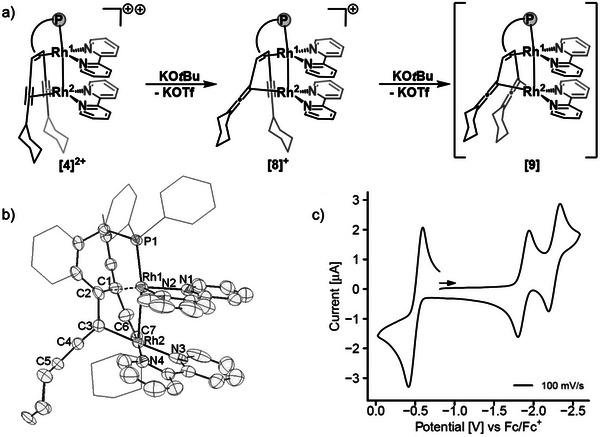
a) Synthesis of deprotonated complexes **[8]** ^+^ and **[9]** with KO*t*Bu in THF. b) Solid state structure of, **[8]** ^+^ hydrogen atoms, counter ion and solvent molecules are omitted for clarity. c) Redox waves of **[8]** ^+^ in THF, the open circuit potential (OCP) and scan direction are shown by an arrow. Conditions: 1 mm analyte, 100 mm [*n*Bu_4_N]PF_6_ electrolyte, WE: Pt, CE: Pt on TiO_x_, RE: Ag/Ag^+^, 100 mV s^−1^ scan rate.

Finally, we investigated the possibility to deprotonate the dication **[4]** ^2+^, which contains a propargylic proton at each of the cyclohexyl groups. This would be an alternative way to convert the dicationic complex stepwise into a neutral one. Moreover, base assisted alkynyl/allene isomerization are a well‐established object of research although often harsh reaction conditions or addition of Lewis‐acids are required.^[^ ^38^, ^39^, ^40^, ^41^, ^42^ ^]^ These isomerization reactions are related to a certain extent to the rearrangement we observed upon reduction of **[4]** ^2+^. We reacted **[4]** ^2+^ with one equivalent of NaO*t*Bu (sodium tert‐butoxide), which caused a color change from red to deep green (λ_max_ = 630 nm) and cleanly led to the formation of a new product **[8]** ^+^ (**Figure** **6**a). This compound was crystallized, and its structure analyzed by XRD methods (the solid‐state structure is depicted in Figure 6b). Indeed, the CH unit of the cyclohexyl substituent in α‐position to the alkynyl group was deprotonated and an allenyl unit, R_2_C═C═C─R is formed [C5 = C4 1.339(13) Å; C4 = C3 1.298(15) Å], which coordinates with its anionic terminus to Rh^2^ [C3‐Rh^2^ 2.035(11) Å]. The second, previously coordinated, carbon center C4 of the former alkynyl unit is now at a distance of 2.856(10) Å from Rh^2^. Related structural motifs have been observed in mono‐ and dinuclear rhodium complexes,^[^ ^43^, ^44^, ^45^, ^46^ ^]^ or in Ru and Os clusters, where however the allenyl fragment additionally coordinates side‐on to a second metal center.^[^ ^47^, ^48^ ^]^

With respect to **[4]** ^2+^, the Rh─Rh bond in **[8]** ^+^ is significantly shortened to 2.6930(9) Å. The ^31^P NMR spectrum shows a doublet of doublets at δ^31^P = 78 ppm (^1^ *J*_PRh1_ = 193 Hz; ^2^ *J*_PRh2_ = 30 Hz) at lower frequencies compared to **[4]** ^2+^. Major differences between the bis(alkynyl) complex **[4]** ^2+^ and allenyl complex **[8]** ^+^ are observed in the ^103^Rh and ^13^C NMR spectra. While δ^103^Rh^1^ is similar in **[4]** ^2+^ (δ^103^Rh = −7043 ppm) and **[8]** ^+^ (δ^103^Rh = −7343 ppm), the resonance of the Rh^2^ nucleus is shifted >4500 ppm to lower frequencies (**[4]** ^2+^: δ^103^Rh^2^ = −743 ppm; **[8]** ^+^: δ^103^Rh^2^ = −5373 ppm). In the ^13^C NMR spectrum of **[8]** ^+^ signals at δ^13^C = 109.6 (*C* ═C═C_cy_), 184.0 (C═ *C* ═C_cy_) and 94.6 ppm (C═C═ *C_cy_*), are attributed to the κ^1^‐C coordinated allenyl. Signals at 87.1 and 85.3 ppm are assigned to the remaining η^2^‐bound alkynyl unit.

The redox properties of **[8]** ^+^ were investigated with CV. Again, two reversible redox waves were observed at E^½^ = −1.89 and −2.28 V (Figure 6c). The notable shift to more negative potentials with respect to **[4]** ^2+^ is attributed to the reduced positive charge in **[8]** ^+^ and conversion of a π‐electron withdrawing alkynyl unit into a σ‐electron donating allenyl unit. DFT calculations indicate that the reduction takes place at the bipy ligands. Additionally, a reversible oxidation event was observed at −0.50 V, which is shifted ≈1 V to more negative potentials when compared to the oxidation of **[6]** ^+^ or **[4]** ^2+^ (see also Table 1). None of these electrochemically generated species have been found to be stable enough to allow for isolation.

A second equivalent of KO*t*Bu was added to **[8]** ^+^ in an attempt to obtain neutral **[9]**, in which both propargylic CH units in the cyclohexyl substituents are deprotonated. Time dependent NMR studies showed formation of **[9]**, although the compound started to decompose (or rearrange) into an unidentified species after 1–2 h. The presence of **[9]** was confirmed by in situ NMR spectroscopy and complemented by DFT calculations of geometry and chemical shifts. The experimental value of δ^31^P = 61.3 ppm is in reasonable agreement with the calculated shift of 49 ppm (theoretical values were referenced to complex **[4]** ^2+^). When compared to complexes **[4]** ^2+^ and **[8]** ^+^, the ^31^P chemical shift is at significantly lower frequencies and the ^2^ *J*_P‐Rh2_ coupling increased even more to 52.3 Hz (see Table 1; and Figure S43, Supporting Information). As expected, the proton resonances of the cyclohexyl group indicate *C*_s_ symmetry for compound **[4]** ^2+^. In the mono allenyl complex **[8]** ^+^ the two cyclohexyl units become asymmetric, which is reflected in a complex ^1^H NMR spectrum. In **[9]** only one set of ^1^H resonances is detected again reflecting the *C*_s_ symmetry. Moreover, the ^1^H resonances of the propargylic CH nuclei have disappeared (for details of the NMR spectra see Figures S37 and S44, Supporting Information). Likewise, the ^13^C NMR spectra of **[9]** indicate the absence of the alkyne groups and presence of a *C*_s_ symmetric compound where the signals of the κ^1^‐C coordinated allenylide units appear at δ^13^C = 114.6 (*C* ═C═C_cy_), 184.4 (C═ *C* ═C_cy_), and 89.6 ppm (C═C═ *C_cy_*). These values only slightly differ from related compound **[8]** ^+^ (see Table 1). The chemical shift for Rh^1^ in **[9]** (δ^103^Rh^1^ = −7606 ppm) is found at more negative values compared to complexes **[4]** ^2+^ and **[8]** ^+^. The resonance of the Rh^2^ nucleus follows the same trend from δ^103^Rh^2^ = −743 ppm in **[4]** ^2+^ to −5373 ppm in **[8]** ^+^ to −6183 ppm in complex **[9]**. Although an experimental structure determination is not possible, DFT calculations give some insight into a possible structure of complex **[9]**, which contains a dianionic conjugated bisallenyl fragment. This structural motif consists of two allenyl fragments with C3 and C6 as anionic 2e‐s‐donors. Both are conjugated via the double bond of the trop‐moiety (see formula of **[9]** in Figure 6a; Figure S64, Supporting Information).

## 3 Conclusion

The molecules Ph_2_Ptrop^(C≡CR)2^ contain an extended conjugated carbon framework composed mainly of alkenyl (sp^2^) and alkynyl (sp) carbon centers as well as some CH (sp^3^) units. The special design of these compounds allows to bind low‐valent metal centers in close proximity such that a Rh^I^Rh^I^ dimeric unit^[^ ^24^ ^]^ can be tightly fixed on this “carbon‐bed”. The metal‐metal bond in these complexes is best described as a donor–acceptor bond, where – neglecting the M─M bond – one d^8^‐metal center in a trigonal pyramidal environment serves as Lewis‐acid^[^ ^24^ ^]^ and the other, in a square planar environment, as donor. Based on the presented experiments in combination with previous work, an amazing diversity of reactivity becomes evident, which seem to be genuine to the low‐valent dinuclear Rh^I^Rh^I^ complexes (while the mono‐nuclear complexes show different reactivity): i) Upon reaction with molecular hydrogen, the alkynyl unit of the Ph_2_Ptrop^(C≡CR)2^ ligand may be converted into a carbene donor unit.^[^ ^24^ ^]^ ii) Consecutive reduction by two electrons converts the dication [Rh_2_(bipy)_2_{Ph_2_Ptrop^(C≡CCy)2^}]^2+^ **[4]** ^2+^ to neutral **[4]** (as indicated by reversible redox waves in the cyclic voltammogram), but **[4]** subsequently rearranges in an unprecedented and concerted manner into complex **[7]**, which contains dibenzocyclo(undecatriene) as central part of the ligand. According to DFT calculations, this curved eleven‐membered carbon cycle carries a two‐fold negative charge. EPR spectra and DFT calculations indicate that in the reduction process of **[4]** ^2+^ most of the electron density in **[4]** ^•+^ and **[4]** is shifted mainly to one of the redox non‐innocent bipy ligands^[^ ^49^, ^50^, ^51^ ^]^ bound to Rh^2^ (in **[4]** ^•+^ this bipy ligand carries a 0.52 e charge, in **[4]** ≈1 e). In course of the slightly exergonic rearrangement process, this charge is depleted from the bipy ligand, which becomes almost neutral, and localized in the C_11_ ring. The Rh─Rh bond is relatively little affected by these changes but, interestingly, becomes shorter in the reduced complexes indicating a strengthening of the Rh─Rh bond in the latter. Overall, the Rh─Rh bond is best viewed as a very weak Rh^2^‐donor→Rh^1^‐acceptor bond, which is significantly longer than a typical Rh─Rh single bond as observed in rhodium(II) acetate, [Rh_2_(O_2_CMe)_4_]:2.29 Å.^[^ ^52^ ^]^ Oxidation of **[7]** partly re‐establishes the central dibenzocycloheptenyl unit and regenerates **[4]** ^2+^ to which two alkynyl substituents are bound, indicating that this rearrangement process is at least partially reversible. iii) Stepwise deprotonation of the CH units in the cyclohexyl substituent in proximity to the Rh^I^_2_(bipy)_2_ core of **[4]** ^2+^ affect again the alkynyl units, which rearrange one after the other into allenyl units to give complexes **[8]** ^+^ and **[9]**. Although the reactions reported here have certainly limited model character with respect to metal particles on carbon support materials, they may nevertheless indicate what transformations and reactions are possible when such materials are exposed to hydrogen, redox processes, or deprotonation reactions with bases.

## Conflict of Interest

The authors declare no conflict of interest.

## Supporting information

Supporting Information

Supporting Information

## Data Availability

The data that support the findings of this study are available from the corresponding author upon reasonable request.
