# Supplementary material for: Structural Changes in the Carbon Sphere of a Dirhodium Complex Induced by Redox or Deprotonation Reactions
Source: Adv Sci (Weinh). 2024 Mar 23;11(22):2400072. doi: 10.1002/advs.202400072 (PMC11165463; doi:10.1002/advs.202400072)
Supplement: Supplementary file 1 — Supporting Information [file ADVS-11-2400072-s002.pdf]

ADVANCED SCIENCE
Open Access

Supporting Information

for *Adv. Sci.*, DOI 10.1002/advs.202400072

Structural Changes in the Carbon Sphere of a Dirhodium Complex Induced by Redox or Deprotonation Reactions

*Clara Schweinzer, Peter Coburger and Hansjörg Grützmacher**

WILEY-VCH

Supporting Information

Structural Changes in the Carbon Sphere of a Dirhodium Complex Induced by Redox or Deprotonation Reactions

*Clara Schweinzer, Peter Coburger and Hansjörg Grützmacher**

General remarks

All reactions and manipulations were carried out under an argon atmosphere using standard Schlenk and glovebox techniques unless stated otherwise. Solvents were either obtained from an MBraun Solvent Purification System, or dried and stored according to common procedures. Br_2TropOH[24,53] and $[Rh_2(COE)_4OTf_2]$[54] were synthesized according to literature procedures. All other compounds are commercially available. NMR spectra were recorded with a Bruker 200, 300, 400 or 500 MHz NMR spectrometer at room temperature unless stated otherwise. NMR spectra were referenced to TMS (^{1}H and ^{13}C), $BF_3{\cdot}OEt_2$ (^{11}B), $CFCl_3$ (^{19}F), H_3PO_4 (^{31}P), $Rh(acac)_3$ (^{103}Rh). Chemical shifts [δ] are given as dimensionless numbers in ppm and the absolute values of the coupling constants are given in Hertz [Hz]. Multiplicities are abbreviated as singlet (s), doublet (d), triplet (t), quartet (q) and broad (br). The chemical shift (^{103}Rh) values are in ppm and were calculated by determining the absolute frequency of the cross peak and relating it to the arbitrary reference frequency ($\Xi(^{103}$Rh$)$ = 3.16 MHz at 100.00 MHz).

Alkyne carbons are indicated as $C_{C\equiv C}$, aromatic carbon and hydrogens as C_{ar} and CH_{ar}, quarternary carbons as C_q, benzylic as C_{benz} and CH_{benz}, propargylic on the cyclohexyl group as CH_{cy}, rest of the cyclohexyl group as CH_{ali} and olefinic as C_{olef}. Single crystals suitable for X-ray diffraction were coated with polyisobutylene oil in a glovebox, transferred to a nylon loop and then transferred to the goniometer of an Oxford SynergyS, Oxford Excalibur or D8-Venture diffractometer equipped with a copper X-ray tube (λ = 1.5406 Å).

Experimental Procedures

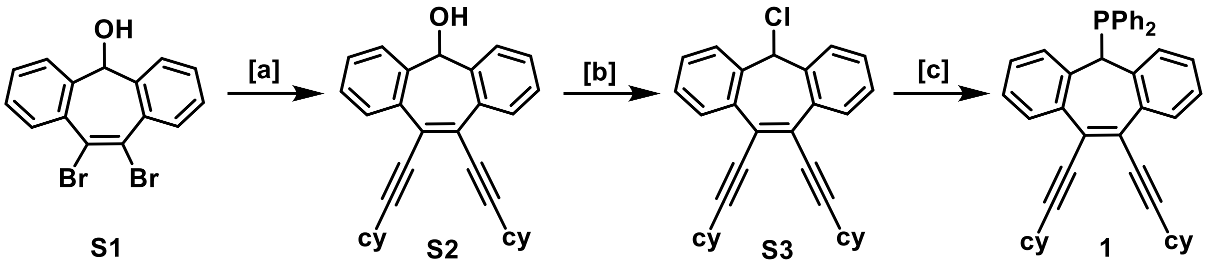

Scheme S1. Synthesis of ligand **1**, adapted from[24,25]. Conditions: [a] cyC≡CH, 5 mol% $[Pd(PPH_3)_4]$, CuI, toluene, Et_3N, 60°C. [b] $SOCl_2$, DCM, 0°C. [c] 1. $HPPh_2$, toluene/hexane, 50°C, 2. Na_2CO_3, water.

Synthesis of (cyC≡C)$_2$tropOH (S2)

Br_2TropOH (**S1**) (5.0 g, 13.7 mmol, 1 eq.) was dissolved in a 1:1 mixture of toluene and triethylamine (150 mL). Tetrakis(triphenylphosphine) palladium (0) (0.79 g, 0.05 eq.) and copper(I) iodide (0.13 g, 0.05 eq.) and degassed cyclohexylacetylene (5.5 mL, 4.5 g, 3 eq.) were added and the resulting mixture was heated to 60°C for 3 days. The resulting dark mixture was filtered over a pad of celite. The filtrate was then concentrated in vacuo and the resulting dark brown residue triturated with n-hexane until a red-brown solid was obtained. The product was purified by column chromatography (silica, *n*-hexane / DCM / ethylacetate 14 / 3 / 1, Rf = 0.3) and recrystallized from the eluent solvent. **S2** was obtained as off-white powder (1.8 g, 31.3 %).

^{1}H NMR (300 MHz, $CDCl_3$) δ 7.72 (d, *J* = 7.7 Hz, 2H, CH_{ar}), 7.62 (d, *J* = 7.7 Hz, 2H, CH_{ar}), 7.32 (t, *J* = 7.6 Hz, 2H, CH_{ar}), 7.18 (t, *J* = 7.6 Hz, 2H, CH_{ar}), 5.22 (s, 1H, CH_{benz}), 2.74 (tt, *J* = 8.7, 4.0 Hz, 2H, CH_{cy}), 2.45 (d, *J* = 3.5 Hz, 1H, O*H*), 2.00 – 1.72 (m, 8H, CH_{ali}), 1.68 – 1.47 (m, 3H, CH_{ali}), 1.43 – 1.29 (m, 6H, CH_{ali}). ^{13}C NMR (75 MHz, Chloroform-*d*) δ 142.57 (C_{ar}), 132.65 (C_{ar}), 129.00 (C_{ar}), 128.41 (C_{ar}), 128.29 (C_{ar}), 126.19 (C_{ar}), 120.58 (C_{ar}), 102.70 (C_{ar}), 82.48 ($C_{C≡C}$), 70.01 ($C_{C≡C}$), 32.78 (C_{ali}), 32.75 (C_{ali}), 30.41 (C_{cy}), 26.15 (C_{ali}), 24.98 (C_{ali}).

Synthesis of (cyC≡C)2tropCl (S3)

(CyCC)$_2$tropOH (**S2**) (1.7 g, 4.2 mmol, 1 equiv.) was dissolved in dry DCM (80 mL) and cooled to 0°C. Next, a solution of distilled $SOCl_2$ (0.34 mL, 4.6 mmol, 1.1 equiv.) and pyridine (0.38 mL, 4.6 mmol, 1.1 equiv.) in dry DCM (20 mL) was added dropwise and stirred for 30 minutes at 0°C and another 30 minutes at room temperature. The solvent was then removed using an external cooling trap. The residue was suspended in little amounts of toluene, filtered over celite to remove pyridinium chloride and the filtrate was concentrated in vacuo. The toluene was evaporated, and the resulting sticky solid was triturated with hexane until a yellowish solid (1.5 g, 80 %) was obtained. NMR spectra showed a mixture of endo and exo conformers present in solution.

^{1}H NMR (300 MHz, $CDCl_3$) δ 1.0-2.0 (m, 20H, CH_{ali}), 2.69 (m, 2H, CH_{cy}), 5.57 (s, 0.42H, C*H*Cl, major conformer), 5.96 (s, 0.58 H, C*H*Cl, minor conformer), 7.40 – 7.02 (m, 6H, CH_{ar}), 7.76 – 7.59 (m, 2H, CH_{ar}), 7.91 (dd, *J* = 8.0, 1.9 Hz, 1H, CH_{ar}). ^{13}C NMR (75 MHz, $CDCl_3$) δ 139.40 (C_{ar}), 138.83 (C_{ar}), 134.94 (C_{ar}), 133.21 (C_{ar}), 130.96 (C_{ar}), 129.10 (C_{ar}), 128.73 (C_{ar}), 128.69 (C_{ar}), 128.40 (C_{ar}), 128.30 (C_{ar}), 128.27 (C_{ar}), 126.96 (C_{ar}), 126.75 (C_{ar}),

125.36 (C_{ar}), 122.70 (C_{ar}), 103.16 ($C_{C\equiv C}$, conformer 1), 102.94 ($C_{C\equiv C}$, conformer 2), 82.66 ($C_{C\equiv C}$, conformer 1), 82.07 ($C_{C\equiv C}$, conformer 2), 65.60 (CH_{benz}, conformer 1), 60.27 (CH_{benz}, conformer 2), 32.74 (C_{ali}), 32.69 (C_{ali}), 32.65 (C_{ali}), 30.43 (C_{cy}), 30.29 (C_{cy}), 26.10 (C_{ali}), 26.07 (C_{ali}), 25.02 (C_{ali}), 24.90 (C_{ali}).

Synthesis of (cyC≡C)$_2$tropPPh$_2$ (1)

(CyCC)$_2$tropCl (**S3**) (1.40 g, 3.2 mmol, 1 equiv.) was suspended in a mixture of dry toluene (16 mL) and dry *n*-hexane (8 mL). Then, diphenylphosphine (0.63 mL, 3.5 mmol, 1.1 equiv.) was added. The reaction mixture was stirred at 50°C over the weekend, while yellow precipitate was formed. The solution mixture was heated to reflux (80°C) for 30 minutes and after it was cooled down degassed sodium carbonate (7 mL, 10% in H_2O) was added. The solution was heated to 50°C for 3 hours before it was heated to reflux (80°C) for 30 minutes. After cooling down, the toluene phase was separated from the aqueous phase and extracted twice with toluene. The solvent was evaporated and the red oil was washed and triturated with *n*-hexane until a powder was obtained. The bright red solid was stirred vigorously in acetonitrile (4 mL) and a pinkish powder was precipitated out of the solution. The product was filtrated, dried, washed with *n*-hexane and dried under vacuum. The off-white powder can be recrystallized from hot toluene. Yield 1.0 g, 53 %. Colourless crystals suitable for X-ray diffraction were obtained from slow diffusion of *n*-hexane in a solution of benzene.

^{1}H NMR (300 MHz, C_6D_6) δ 8.11 (dd, J = 7.8, 1.4 Hz, 2H, CH_{ar}), 7.45 (ddd, J = 8.2, 6.8, 1.8 Hz, 4H, CH_{ar}), 7.06 – 6.57 (m, 12H, CH_{ar}), 4.82 (d, $^2J_{PH}$ = 5.6 Hz, 1H, CH_{benz}), 2.86 – 2.57 (m, 2H, CH_{cy}), 1.93 – 1.55 (m, 12H, CH_{ali}), 1.44 – 1.06 (m, 8H, CH_{ali}). ^{13}C NMR (75 MHz, C_6D_6) δ 141.49 (d, J = 8.9 Hz, CH_{ar}), 138.97 (CH_{ar}), 138.68 (CH_{ar}), 136.22 (d, J = 4.4 Hz, CH_{ar}), 133.92 (d, $^2J_{CP}$ = 20.1 Hz, 4 C, CH_{ar}), 130.54 (CH_{ar}), 130.40 (d, $^4J_{CP}$ = 6.3 Hz, 2 C, C_{olef}), 129.21, 129.17, 128.70, 128.37, 128.07, 127.98, 126.29, 101.97 (s, 2 C, *C*≡C), 84.40 (s, 2 C, $C_{C\equiv C}$), 56.83 (d, $^1J_{CP}$ = 21.9 Hz, 1 C, CH_{benz}), 32.94 (s, 1 C, CH_2), 32.89 (s, 1 C, C_{ali}), 30.56 (s, 2 C, CH_{cy}), 26.29 (s, 1 C, C_{ali}), 24.90 (s, 4 C, C_{ali}). ^{31}P{^{1}H} NMR (121.5 MHz, C_6D_6): δ -14.0 (s, 1 P, TropP). Anal. calcd for $C_{43}H_{41}P\cdot(Na_2CO_3)_{0.1}$ C 86.37, H 6.90; found: C 86.69, H 7.18.

[((cyC≡C)$_2$TropPPh$_2$)Rh$_2$(μ-Cl)$_2$]$_2$ ([2])

$[Rh_2(\mu\text{-}Cl)_2(COE)_4]$ (244 mg, 0.340 mmol, 1 equiv.) and $(cyC{\equiv}C)_2tropPPh_2$ (**1**) (200 mg, 0.340 mmol, 1 equiv.) were stirred in benzene (2 mL). After 15 minutes a precipitate started to appear. The reaction mixture was stirred overnight and then filtrated. The solid was washed with *n*-hexane until all cyclooctene was removed. The orange-red solid was then dried under reduced pressure. Yield: 240 mg (82 %). Bright red crystals were obtained by slow diffusion of hexane into a solution of DCM.

^1H NMR (300 MHz, CD_2Cl_2) δ 7.93 (d, J = 7.6 Hz, 2H, CH_{ar}), 7.61 (t, J = 9.0 Hz, 4H, CH_{ar}), 7.45 – 6.84 (m, 16H, CH_{ar}), 4.86 (dd, J = 14.2 Hz, 2.6 Hz, 1H, CH_{benz}), 2.60 – 2.47 (m, 2H, CH_{cy}), 2.65 – 2.02 (m, 6H, CH_{ali}), 1.95 – 1.51 (m, 8H, CH_{ali}), 1.50 – 1.08 (m, 6H, CH_{ali}). ^{13}C NMR (75 MHz, CD_2Cl_2) δ 135.83 (d, J = 7.5 Hz, 2C, C_{ar}), 134.47 (dd, J = 4.3, 1.7 Hz, 2C, C_{ar}), 132.92 (d, J = 9.4 Hz, 2C, C_{ar}), 130.63 (CH_{ar}), 130.07 (CH_{ar}), 129.04 (d, J = 2.4 Hz, 2C, C_{ar}), 127.92, 127.84, 127.39, 126.79, 126.66, 126.34, 125.36, 83.49 (d, J = 14.4 Hz, 2C, $C_{C\equiv C}$), 69.85 (d, J = 7.4 Hz, 2C, $C_{C\equiv C}$), 49.26 (d, $^1J_{CP}$ = 26.1 Hz, 1C, CH_{benz}), 38.89 (d, J = 15.1 Hz, 2C, C_{olef}), 34.78 (C_{ali}), 33.13 (d, J = 26.4 Hz) (C_{ali}), 25.29 (C_{ali}), 25.11 (d, J = 4.6 Hz, C_{ali}). ^{31}P NMR (121 MHz, CD_2Cl_2) δ 101.23 (dd, $^1J_{PRh}$ = 212.4 Hz, $^2J_{PRh}$ = 4.5 Hz, TropP). ^{103}Rh NMR (15.8 MHz, CD_2Cl_2): δ -6724 (d, $^1J_{RhP}$ = 212.8 Hz, Rh^1), -4736 (d, $^2J_{PRh}$ = 108.5 Hz, Rh^2). Anal. calcd for $C_{86}H_{82}Cl_4P_2Rh_4\cdot(CH_2Cl_2)$: C 57.54, H 4.66; found: C 57.83, H 4.85.

$[((cyC{\equiv}C)_2TropPPh_2)Rh_2OTf_2]$ ([3])

$(CyC{\equiv}C)_2tropPPh_2$ (**1**) (100 mg, 0.170 mmol, 1 eq.) was stirred with $[Rh_2(COE)_4OTf_2]$ (160 mg, 0.170 mmol, 1 eq.) for 1 h in THF (5 mL). The solvent was removed in vacuo and the dark brown solid washed with hexane until all COE was removed. The product can be recrystallized from THF/hexane. Yield: 160 mg (87 %). Crystals suitable for X-ray diffraction were grown from slow diffusion of *n*-hexane into a solution of [**3**] in DCM.

^1H NMR (500 MHz, Acetonitrile-d_3) δ 8.03 (dd, J = 7.7, 1.3 Hz, 2H, CH_{ar}), 7.65 (tt, J = 7.1, 1.7 Hz, 2H, CH_{ar}), 7.57 (ddt, J = 9.9, 6.8, 1.5 Hz, 4H, CH_{ar}), 7.54 – 7.46 (m, 7H, CH_{ar}), 7.44 – 7.32 (m, 4H, CH_{ar}), 5.50 (dd, J = 15.3, 2.7 Hz, 1H, CH_{benz}), 2.88 (td, J = 10.7, 5.3 Hz, 2H, CH_{cy}), 2.54 (d, J = 12.2 Hz, 2H, CH_{ali}), 2.47 (dd, J = 12.6, 4.1 Hz, 2H, CH_{ali}), 2.05 – 1.77 (m, 9H, CH_{ali}), 1.67 (dddd, J = 15.1, 13.0, 9.9, 3.5 Hz, 5H, CH_{ali}), 1.58 – 1.46 (m, 2H, CH_{ali}). ^{13}C NMR (126 MHz, Acetonitrile-d_3) δ 135.48 (C_{ar}), 134.36 (C_{ar}), 133.05 (d, J = 9.2 Hz, C_{ar}), 131.63 (C_{ar}), 129.24 (d, J = 6.8 Hz, C_{ar}), 128.77 (C_{ar}), 128.52 – 128.18 (m, C_{ar}), 127.74 (C_{ar}), 126.86 (C_{ar}), 83.48 (d, J = 12.2 Hz, $C_{C\equiv C}$), 68.58 (d, J = 6.7 Hz, $C_{C\equiv C}$), 47.54 (d, J = 27.2 Hz,

CH_{benz}), 37.51 (d, J = 15.9 Hz, C_{olef}) 35.19 (C_{ali}), 33.79 (C_{ali}), 33.53 (C_{ali}), 25.59 (C_{ali}), 25.35 (C_{ali}). ^{19}F NMR (471 MHz, Acetonitrile-d_3) δ -79.30 (s, OTf). ^{31}P NMR (162 MHz, Acetonitrile-d_3) δ 106.03 (dd, $^{1}J_{PRh}$ = 191.7 Hz, $^{2}J_{PRh}$ = 5.7 Hz, TropP); (121 MHz, CD_2Cl_2) δ 97.24 (dd, $^{1}J_{PRh}$ = 217.8 Hz, $^{2}J_{PRh}$ = 3.8 Hz, TropP). ^{103}Rh NMR (15.8 MHz, Acetonitrile-d_3): δ -6973 (d, $^{1}J_{RhP}$ = 197.2 Hz, Rh^1), -688 (d, $^{2}J_{RhP}$ = 194.1 Hz, Rh^2). Anal. calcd for $C_{45}H_{41}F_6O_6PRh_2S_2$: C 49.46, H 3.78; found: C 49.30, H 4.29.

[((cyC≡C)$_2$TropPPh$_2$)Rh$_2$bipy$_2$](OTf)$_2$ ([4](OTf)$_2$)

Method 1: Rh_2OTf_2 ([**3**]) (100 mg, 0.092 mmol, 1 eq.) and bipy (29 mg, 0.183 mmol, 2 eq.) were stirred in THF (5 mL) for 10 min. After a few minutes a solid started to appear (the product is only moderately soluble in THF). The solvent was removed and washed with hexane to obtain a dark red powder. Yield: 122 mg (95 %).

Method 2: [((cyC≡C)$_2$TropPPh$_2$)Rh$_2$(μ-Cl)$_2$]$_2$ ([**2**]) (100 mg, 0.058 mmol, 1 eq.) and bipy (36 mg, 0.231 mmol, 4 eq.) were stirred for 5 min in 5 mL THF, then KOTf (43 mg, 0.231 mmol, 4 eq.) was added and the mixture was stirred overnight. The solid KCl was filtered off, washed with 5mL MeCN and the solution dried in vacuo. The dark red powder was washed with hexane and Et_2O. Yield: 130 mg (80 %).

Crystals were grown from slow diffusion of hexane into a solution of THF.

^{1}H NMR (500 MHz, CD_3CN) δ 8.42 (d, J = 5.3 Hz, 2H, CH_{ar}), 8.35 (d, J = 7.8 Hz, 2H, CH_{ar}), 8.11 (d, J = 5.7 Hz, 2H, CH_{ar}), 7.90 (td, J = 7.8, 1.6 Hz, 2H, CH_{ar}), 7.83 (d, J = 8.0 Hz, 2H, CH_{ar}), 7.56 (p, J = 7.4, 6.4 Hz, 6H, CH_{ar}), 7.35 (t, J = 7.5 Hz, 2H, CH_{ar}), 7.29 (dd, J = 7.4, 5.8 Hz, 2H, CH_{ar}), 7.26 (d, J = 7.5 Hz, 2H, CH_{ar}), 7.20 (t, J = 7.7 Hz, 2H, CH_{ar}), 6.96 (td, J = 7.9, 2.4 Hz, 4H, CH_{ar}), 6.81 – 6.73 (m, 7H, CH_{ar}), 5.27 (dd, J = 15.2, 2.8 Hz, 1H, CH_{benz}), 2.59 (ddd, J = 11.4, 7.8, 3.6 Hz, 2H, CH_{cy}), 2.51 (d, J = 13.1 Hz, 2H, CH_{ali}), 2.19 (d, J = 12.7 Hz, 2H, CH_{ali}), 1.91 – 1.63 (m, 10H, CH_{ali}), 1.45 – 1.23 (m, 5H, CH_{ali}). ^{13}C NMR (126 MHz, CD_3CN) δ 156.28 (C_{ar}), 154.87 (C_{ar}), 153.31 (C_{ar}), 152.12 (C_{ar}), 151.50 (C_{ar}), 144.40 (C_{ar}), 141.64 (C_{ar}), 139.77 (C_{ar}), 136.85 (d, J = 7.9 Hz, C_{ar}), 136.49 (C_{ar}), 133.09 (d, J = 9.0 Hz, C_{ar}), 131.94 (d, J = 2.7 Hz, C_{ar}), 131.65 (C_{ar}), 130.25 (d, J = 6.9 Hz, C_{ar}), 129.40 (C_{ar}), 129.12 (d, J = 10.1 Hz, C_{ar}), 128.67 (C_{ar}), 128.33 (C_{ar}), 128.03 (C_{ar}), 127.50 (C_{ar}), 123.95 (C_{ar}), 123.43 (C_{ar}), 88.18 (d, J = 11.7 Hz, $C_{C≡C}$), 79.42 (d, J = 6.4 Hz, $C_{C≡C}$), 49.26 (d, J = 26.9 Hz, C_{benz}), 38.58 (d, J = 14.0 Hz, C_{olefin}), 37.28 (C_{cy}), 34.84 (C_{ali}), 34.78 (C_{ali}), 32.32 (C_{ali}), 26.94 (C_{ali}), 26.53 (C_{ali}), 26.19 (C_{ali}), 23.36 (C_{ali}). ^{19}F NMR (471 MHz, CD_3CN) δ -79.29 (s, OTf). ^{31}P NMR (202 MHz, CD_3CN) δ 96.32 (dd, J = 192.6, 9.4 Hz, TropP). ^{103}Rh NMR (16 MHz,

CD_3CN) δ -7045 (d, $^1J_{RhP}$ = 194.2 Hz, Rh^1), -744 (d, $^2J_{RhP}$ = 189.3 Hz, Rh^2). UV-vis (THF): λ_{max} = 535, 390 nm. Anal. calcd for $C_{65}H_{57}F_6N_4O_6PRh_2S_2\cdot(K_1C_1F_3O_3S_1)_{0.2}$: C 54.28, H 3.89, N 3.88; found: C 54.29, H 4.12, N 4.01.

[((cyC≡C)$_2$TropPPh$_2$)Rh$_2$bipy$_2$](OTf) ([4](OTf))

[((cyC≡C)$_2$TropPPh$_2$)Rh$_2$bipy$_2$]OTf$_2$ ([**4**](OTf)$_2$) (22 mg, 0.016 mmol, 1 eq.) was stirred with sodium naphthalenide solution (0.1 M in THF, 157 µL, 0.016 mmol, 1 eq.) and 15-crown-5 (3.5 mg, 0.016 mmol, 1 eq.) for 1 h. The solution was filtrated and placed in a freezer at -20°C to obtain a crystalline, dark red, paramagnetic product, that was washed with hexane and Et_2O. Yield: 10 mg (60 %). Crystals suitable for X-ray diffraction were grown from a saturated THF solution at -20 °C. UV-Vis (MeCN): λ_{max} = 579, 390 nm. Anal. calcd for $C_{64}H_{57}F_3N_4O_3PRh_2S$: C 61.20, H 4.57, N 4.46; found: C 61.40, H 4.81, N 4.74.

[((cyC≡C)$_2$TropPPh$_2$)Rh(µ-Cl)]$_2$ ([5])

[Rh$_2$(µ-Cl)$_2$(H$_2$C=CH$_2$)$_4$] (66 mg, 0.170 mmol, 0.5 equiv.) and (cyC≡C)$_2$tropPPh$_2$ (**1**) (200 mg, 0.340 mmol, 1 equiv.) were stirred in benzene (2 mL). After 15 minutes a precipitate started to appear. The reaction mixture was stirred for 3 h and then filtrated. The orange solid was washed with *n*-hexane twice and dried under reduced pressure. Yield: 190 mg (77 %). Bright red crystals were obtained from a toluene solution at -20 °C.

^1H NMR (300 MHz, $CDCl_3$) δ 8.11 (dd, *J* = 7.6, 1.5 Hz, 1H, CH_{ar}), 7.89 – 7.64 (m, 2H, CH_{ar}), 7.38 – 7.09 (m, 5H, CH_{ar}), 7.01 (d, *J* = 7.6 Hz, 1H, CH_{ar}), 4.90 (dd, *J* = 13.4, 3.3 Hz, 1H, CH_{benz}), 2.74 (td, *J* = 9.6, 8.4, 4.3 Hz, 1H, CH_{cy}), 2.16 – 2.01 (m, 2H, CH_{ali}), 1.91 (td, *J* = 9.3, 4.5 Hz, 2H, CH_{ali}), 1.85 – 1.74 (m, 1H, CH_{ali}), 1.66 (d, *J* = 6.9 Hz, 1H, CH_{ali}), 1.50 (d, *J* = 12.6 Hz, 4H, CH_{ali}). ^{13}C NMR (75 MHz, $CDCl_3$) δ 137.10 (C_{ar}), 137.06 (C_{ar}), 135.18 (C_{ar}), 135.09 (C_{ar}), 133.79 (C_{ar}), 133.66 (C_{ar}), 131.52 (C_{ar}), 130.89 (C_{ar}), 130.70 (C_{ar}), 130.66 (C_{ar}), 128.86 (C_{ar}), 128.39 (C_{ar}), 128.20 (C_{ar}), 128.16 (C_{ar}), 128.06 (C_{ar}), 127.50 (C_{ar}), 127.20 (C_{ar}), 112.41 ($C_{C\equiv C}$), 112.35 ($C_{C\equiv C}$), 57.96 (d, *J* = 12.1 Hz, CH_{olefin}), 50.03 (d, *J* = 30.3 Hz, CH_{benz}), 32.17 (C_{ali}), 32.15 (C_{ali}), 31.27 (C_{ali}), 26.01 (C_{ali}), 25.01 (C_{ali}), 24.98 (C_{ali}). ^{31}P NMR (202 MHz, $CDCl_3$) δ 113.45 (d, $^1J_{RhP}$ = 267.8 Hz, TropP). ^{103}Rh NMR (16 MHz, CD_2Cl_2) δ -7405.6 (d, $^1J_{RhP}$ = 269.8 Hz, Rh). Anal. calcd for $C_{86}H_{82}Cl_4P_2Rh_2$: C 71.03, H 5.68; found: C 69.83, H 6.26.

[((cyC≡C)$_2$TropPPh$_2$)Rhbipy](OTf) ([6](OTf))

[((cyC≡C)$_2$TropPPh$_2$)Rh(μ-Cl)]$_2$ ([**5**]) (100 mg, 0.058 mmol, 1 eq.) and bipy (18 mg, 0.156 mmol, 2 eq.) were stirred for 5 min in THF (5 mL), then KOTf (22 mg, 0.156 mmol, 2 eq.) was added and the mixture was stirred for 3 h. The solid KCl was filtered off, and the solvent removed in vacuo. The dark red powder was washed with hexane and Et_2O. Yield: 125 mg (91 %). Single crystals were grown from a chloroform solution layered with *n*-hexane.

^{1}H NMR (200 MHz, THF-d_8) δ 8.81 (d, J = 7.9 Hz, 4H, CH_{ar}), 8.25 (d, J = 7.8 Hz, 2H, CH_{ar}), 8.12 (t, J = 8.4 Hz, 2H, CH_{ar}), 7.46 – 7.13 (m, 10H, CH_{ar}), 7.09 – 6.86 (m, 8H, CH_{ar}), 5.25 (dd, J = 13.8, 3.2 Hz, 1H, CH_{benz}), 2.60 – 2.35 (m, 2H, CH_{cy}), 1.73 – 1.12 (m, 20H, CH_{ali}). ^{13}C NMR (126 MHz, $CDCl_3$) δ 153.91 (C_{ar}), 152.04 (C_{ar}), 140.19 (C_{ar}), 137.02 (C_{ar}), 135.83 (C_{ar}), 131.91 (C_{ar}), 131.84 (C_{ar}), 131.23 (C_{ar}), 129.94 (C_{ar}), 129.56 (C_{ar}), 129.35 (C_{ar}), 128.37 (C_{ar}), 128.29 (C_{ar}), 128.14 (C_{ar}), 127.83 (C_{ar}), 127.78 (C_{ar}), 127.09 (C_{ar}), 126.17 (C_{ar}), 123.83 (C_{ar}), 107.85 (d, J = 5.9 Hz, $C_{C≡C}$), 78.54 ($C_{C≡C}$), 50.93 (d, J = 29.1 Hz, CH_{benz}), 47.11 (d, J = 12.7 Hz, C_{olefin}), 32.33 (C_{ali}), 31.15 (C_{cy}), 24.88 (C_{ali}), 24.86 (C_{ali}). ^{19}F NMR (188 MHz, THF-d_8) δ -79.00 (s, OTf). ^{31}P NMR (81 MHz, THF-d_8) δ 111.61 (d, $^1J_{RhP}$ = 225.4 Hz, TropP); (202 MHz, $CDCl_3$) δ 111.07 (d, $^1J_{RhP}$ = 226.4 Hz, TropP). ^{103}Rh NMR (16 MHz, THF-d_8) δ -7086.43 (d, $^1J_{RhP}$ = 229.0 Hz, Rh). Anal. calcd for $C_{54}H_{49}F_3N_2O_3PRhS$: C 65.06, H 4.95, N 2.81; found: C 65.24, H 5.14, N 4.15.

[((6,7-dicyclohexyl-2,10-dibenzyl-cycloundeca-4,5,7,8-tetraene)diphenylphosphine)Rh$_2$bipy$_2$] ([7])

[((cyC≡C)$_2$TropPPh$_2$)Rh$_2$bipy$_2$]OTf$_2$ ([**4**](OTf)$_2$) (30 mg, 0.02 mmol, 1 eq.) was dissolved in 1 mL DFB and CoCp*$_2$ (16 mg, 0.05 mmol, 2.2 eq.) was added as a solution in DFB (1 mL). The mixture was stirred overnight while a green solid started to appear. The solid was filtrated off and suspended in THF. The mixture was dried and the remaining dark green solid washed with hexane and Et_2O. Yield: 20 mg, 83%. Crystals suitable for X-ray diffraction were grown from slow diffusion of *n*-hexane into a solution of pyridine.

^{1}H NMR (500 MHz, THF-d_8) δ 7.89 (d, J = 5.8 Hz, 2H, CH_{ar}), 7.74 (d, J = 6.1 Hz, 2H, CH_{ar}), 7.51 (d, J = 6.7 Hz, 2H, CH_{ar}), 7.19 (d, J = 8.1 Hz, 2H, CH_{ar}), 7.11 (d, J = 7.3 Hz, 2H, CH_{ar}), 7.07 – 6.92 (m, 8H, CH_{ar}), 6.82 (dt, J = 6.4, 3.4 Hz, 10H, CH_{ar}), 6.64 (t, J = 7.5 Hz, 2H) , CH_{ar}, 6.45 (t, J = 6.3 Hz, 2H, CH_{ar}), 5.98 (t, J = 6.4 Hz, 2H, CH_{ar}), 5.09 (dd, J = 16.2, 2.6 Hz, 1H, CH_{benz}), 2.56 (tt, J = 11.4, 3.4 Hz, 2H, CH_{cy}), 2.15 (d, J = 12.5 Hz, 2H, CH_{ali}), 1.98 (d, J = 13.4 Hz, 2H, CH_{ali}), 1.83 – 1.68 (m, 6H, CH_{ali}), 1.60 (q, J = 10.7 Hz, 4H, CH_{ali}), 1.41 – 1.24

(m, 6H, C*H*$_{ali}$). ^{1}H NMR (300 MHz, Pyridine- *d*$_5$) δ 8.18 (d, J = 4.5 Hz, 2H, C*H*$_{ar}$), 7.99 (d, J = 5.6 Hz, 2H, C*H*$_{ar}$), 7.94 (dd, J = 7.6, 1.5 Hz, 2H, C*H*$_{ar}$), 7.42 (d, J = 6.3 Hz, 1H, C*H*$_{ar}$), 7.32 (t, J = 6.8 Hz, 1H, C*H*$_{ar}$), 7.18 – 6.91 (m, 10H, C*H*$_{ar}$), 6.74 (t, J = 7.5 Hz, 2H, C*H*$_{ar}$), 6.58 (t, J = 6.6 Hz, 2H, C*H*$_{ar}$), 6.15 (t, J = 6.3 Hz, 2H, C*H*$_{ar}$), 5.50 (dd, J = 16.3, 1.9 Hz, 1H, C*H*$_{benz}$), 2.74 (d, J = 11.0 Hz, 2H, C*H*$_{cy}$), 2.38 (d, J = 12.2 Hz, 2H, C*H*$_{ali}$), 1.98 (d, J = 11.7 Hz, 2H, C*H*$_{ali}$), 1.85 – 1.69 (m, 14H, C*H*$_{ali}$), 1.68 – 1.61 (m, 3H, C*H*$_{ali}$), 1.35 – 1.24 (m, 12H, C*H*$_{ali}$). ^{13}C NMR (126 MHz, Pyridine-*d*$_5$) δ 152.83 (CH$_{ar}$), 151.65 (CH$_{ar}$), 143.00 (d, *J* = 18.6 Hz, C-Rh), 141.57 (C$_q$), 133.26 (d, *J* = 10.0 Hz, CH$_{ar}$), 132.16 (d, *J* = 6.8 Hz, CH$_{ar}$), 129.38 (CH$_{ar}$), 128.94 (CH$_{ar}$), 128.20 (d, *J* = 8.6 Hz, CH$_{ar}$), 127.72 (CH$_{ar}$), 127.24 (CH$_{ar}$), 121.98 – 121.18 (m, CH$_{ar}$), 121.66 (d, *J* = 6.4 Hz, CH$_{ar}$), 121.58 (CH$_{ar}$), 120.67 (CH$_{ar}$), 118.08 (dd, *J* = 11.8, 5.4 Hz, C$_{allyl2}$), 114.49 (CH$_{ar}$), 72.19 (d, *J* = 6.1 Hz , C$_{allyl1}$), 47.80 (d, *J* = 31.3 Hz, CH$_{benz}$), 39.60 (C$_{cy}$), 37.92 (C$_{ali}$), 36.05 (C$_{ali}$), 27.77 (C$_{ali}$), 27.65 (C$_{ali}$), 27.08 (C$_{ali}$). ^{31}P NMR (203 MHz, THF-*d*$_8$) δ 18.85 (dd, *J* = 180.6, 21.0 Hz, TropP). (Pyridine-*d*$_5$) δ 18.64 (dd, *J* = 180.7, 21.8 Hz, TropP). ^{103}Rh NMR (16 MHz, THF-*d*$_8$) δ -7167.6 (d, $^{1}J_{RhP}$ = 180.8 Hz, Rh^1). ^{103}Rh NMR (16 MHz, Pyridine-*d*$_5$) δ -7163.3 (d, $^{1}J_{RhP}$ = 179.2 Hz, Rh^1). No signal for Rh^2 could be detected due to low solubility. UV-vis (THF): λ_{max} = 860, 700 nm. Anal. calcd for $C_{63}H_{57}N_4PRh_2\cdot(CoC_{20}H_{30}F_3O_3S)_{0.4}$: C 66.05, H 5.36, N 4.32; found: C 65.80, H 5.59, N 5.52.

[((cy=C=C)(cyC≡C)TropPPh$_2$)Rh$_2$bipy$_2$](OTf) ([8](OTf))

[((cyC≡C)$_2$TropPPh$_2$)Rh$_2$bipy$_2$]OTf$_2$ ([**4**](OTf)$_2$) (20 mg, 14.2 mmol, 1 eq.) was dissolved in 1 mL THF and KO*t*Bu (1.6 mg, 14.2 mmol, 1 eq.) was added dropwise as 5 M solution in THF, while the dark red solution immediately turned dark green. After stirring for 15 min, the solvent was removed in vacuo and the dark green solid was washed with Et$_2$O and hexane. Yield: expected 17.9 mg (%). Crystals suitable for X-ray diffraction were grown from slow evaporation of Et$_2$O into a solution of THF.

^{1}H NMR (500 MHz, THF-*d*$_8$) δ 9.33 (d, *J* = 7.8 Hz, 1H, C*H*$_{ar}$), 9.07 (d, *J* = 5.8 Hz, 1H, C*H*$_{ar}$), 8.35 (dd, *J* = 10.1, 6.3 Hz, 3H, C*H*$_{ar}$), 8.22 (dd, *J* = 23.2, 8.1 Hz, 2H, C*H*$_{ar}$), 8.01 – 7.75 (m, 4H, C*H*$_{ar}$), 7.54 (q, *J* = 9.2, 8.7 Hz, 2H, C*H*$_{ar}$), 7.38 – 7.14 (m, 3H, C*H*$_{ar}$), 7.14 – 7.01 (m, 8H, C*H*$_{ar}$), 6.85 (q, *J* = 6.3, 5.5 Hz, 4H, C*H*$_{ar}$), 6.82 – 6.71 (m, 4H, C*H*$_{ar}$), 6.68 – 6.57 (m, 1H, C*H*$_{ar}$), 4.92 (d, *J* = 12.5 Hz, 1H, C*H*$_{benz}$), 2.69 (d, *J* = 10.4 Hz, 3H, C*H*$_{ali}$), 2.57 (s, 1H, C*H*$_{ali}$), 2.36 – 2.24 (m, 1H, C*H*$_{ali}$), 2.14 (d, *J* = 14.2 Hz, 4H, C*H*$_{ali}$), 2.02-1.55 (m, 11H, C*H*$_{ali}$), 1.54 – 1.21 (m, 1H, C*H*$_{ali}$). ^{13}C NMR (126 MHz, THF-*d*$_8$) δ 184.01 (C=*C*=C), 157.27 (C$_{ar}$), 154.69 (C$_{ar}$), 154.31 (C$_{ar}$), 153.89 (C$_{ar}$), 153.48 (C$_{ar}$), 152.21 (C$_{ar}$), 149.86 (C$_{ar}$), 144.20 (d, *J* = 9.1

Hz, C_{ar})), 140.73 (d, *J* = 8.1 Hz, C_{ar})), 138.48 (d, *J* = 4.5 Hz, C_{ar}), 137.71 (C_{ar}), 133.08 (d, *J* = 9.1 Hz, C_{ar}), 132.96 (d, *J* = 9.1 Hz, C_{ar}), 130.13 (CH_{ar}), 128.44 (dd, *J* = 9.1, 2.7 Hz, C_{ar}), 127.87 (d, *J* = 17.7 Hz, C_{ar}), 126.19 (d, *J* = 3.6 Hz, C_{ar}), 125.94 (C_{ar}), 125.42 (C_{ar}), 109.55 (d, *J* = 22.7 Hz, C=C=*C*-Rh), 94.62 ($C_{cy,\,q}$), 87.09 (d, *J* = 5.4 Hz, C≡C), 85.25 (d, *J* = 5.4 Hz, C≡C), 52.40 (d, *J* = 22.7 Hz, CH_{benz}), 37.90 (d, *J* = 25.4 Hz, C_{olefin}), 36.59 (C_{ali}), 35.63 (C_{ali}), 34.77 (CH_{cy}), 28.09 (C_{ali}), 27.71 (C_{ali}), 27.04 (C_{ali}). ^{31}P NMR (203 MHz, THF-d_8) δ 78.46 (dd, *J* = 193.9, 29.7 Hz, TropP). ^{19}F NMR (471 MHz, THF-d_8) δ -79.21 (s, OTf). ^{103}Rh NMR (16 MHz, THF-d_8) δ -7342.5 (d, $^{1}J_{RhP}$ = 195 Hz, Rh^1), -5372.4 (d, $^{2}J_{RhP}$ = 227 Hz, Rh^2). UV-vis (THF): λ_{max} = 630, 370 nm. Anal. calcd for $C_{64}H_{56}F_3N_4O_3PRh_2S\cdot(KCF_3O_3S)_{0.3}$: C 58.89, H 4.30, N 4.27; found: C 58.48, H 4.70, N 4.04.

[((cy=C=C)$_2$TropPPh$_2$)Rh$_2$bipy$_2$] ([9])

Because of the instability of the product, the complex was generated *in-situ* in an NMR tube. [((CyC≡C)$_2$TropPPh$_2$)Rh$_2$bipy$_2$]OTf$_2$ ([**4**](OTf)$_2$) (10 mg, 7.1 mmol, 1 eq.) was dissolved in THF-d_8 (0.4 mL) and KO*t*Bu-d_9 (1.72 mg, 14.2 mmol, 2 eq.) was added in two portions and shaken. The solution turned green immediately, NMR spectra were recorded when ^{31}P NMR showed full conversion or at several time points were recorded after shaking for 10 min. See also Figure S44 and Figure S45.

^{1}H NMR (400 MHz, THF-d_8) δ 9.45 (d, *J* = 7.9 Hz, 1H, CH_{ar}), 9.27 (t, *J* = 7.2 Hz, 2H, CH_{ar}), 8.61 (d, *J* = 4.9 Hz, 2H, CH_{ar}), 8.48 (d, *J* = 7.9 Hz, 1H, CH_{ar}), 8.31 (d, *J* = 7.7 Hz, 1H, CH_{ar}), 7.81 (td, *J* = 7.7, 2.0 Hz, 1H, CH_{ar}), 7.51 (d, *J* = 8.1 Hz, 1H, CH_{ar}), 7.43 (t, *J* = 7.3 Hz, 1H, CH_{ar}), 7.37 (d, *J* = 8.1 Hz, 1H, CH_{ar}), 7.35 – 7.15 (m, 4H, CH_{ar}), 7.09 – 6.97 (m, 6H, CH_{ar}), 6.86 – 6.78 (m, 5H, CH_{ar}), 6.73 (dd, *J* = 7.2, 3.0 Hz, 6H, CH_{ar}), 6.56 (dd, *J* = 7.8, 4.9 Hz, 1H, CH_{benz}), 4.57 (d, *J* = 13.0 Hz, 1H, CH_{ali}), 2.73 (dt, *J* = 13.0, 4.9 Hz, 1H, CH_{ali}), 2.39 (ddd, *J* = 12.8, 9.4, 4.4 Hz, 1H, CH_{ali}), 2.27 (dt, *J* = 13.0, 4.4 Hz, 2H, CH_{ali}), 1.97 (ddd, *J* = 13.2, 9.3, 4.2 Hz, 1H, CH_{ali}), 1.91 – 1.76 (m, 1H, CH_{ali}), 1.68 – 1.31 (m, 3H, CH_{ali}). ^{13}C NMR (126 MHz, THF-d_8) δ 184.40 (s, C=*C*=C), 154.93 (s, CH_{ar}), 154.81 (s, CH_{ar}), 153.25 (s, CH_{ar}), 152.35 (s, CH_{ar}), 150.08 (s, C_q), 147.06 (d, J_{CP} = 12.5 Hz, C_q), 138.61 (s, CH_{ar}), 134.82 (s, CH_{ar}), 132.93 (d, *J* = 10.2 Hz, CH_{ar}), 131.77 (s, CH_{ar}), 128.41 (s, CH_{ar}), 127.86 (s, CH_{ar}), 127.79 (s, CH_{ar}), 124.18 (d, *J* = 6.4 Hz, CH_{ar}), 121.09 (d, *J* = 13.5 Hz, CH_{ar}), 114.64 (d, J_{CRh} = 25.9 Hz, C=C=*C*-Rh), 89.56 (s, $C_{cy,\,q}$), 53.98 (d, J_{CP} = 19.4 Hz, CH_{benz}), 37.05 (s, C_{olef}), 35.69 (s, $CH_{2,\,ali}$), 28.55 (s, $CH_{2,\,ali}$), 28.40 (s, $CH_{2,\,ali}$), 26.55 (s, $CH_{2,\,ali}$), 25.98 (s, $CH_{2,\,ali}$). ^{31}P NMR

(202 MHz, THF-d_8) δ 60.95 (dd, J = 193.2, 54.3 Hz, TropP). ^{103}Rh NMR (16 MHz, THF-d_8) δ -7606.0 (d, $^{1}J_{RhP}$ = 192 Hz, Rh^1), -6183 (d, $^{2}J_{RhP}$ = 54 Hz, Rh^2).

NMR Spectra

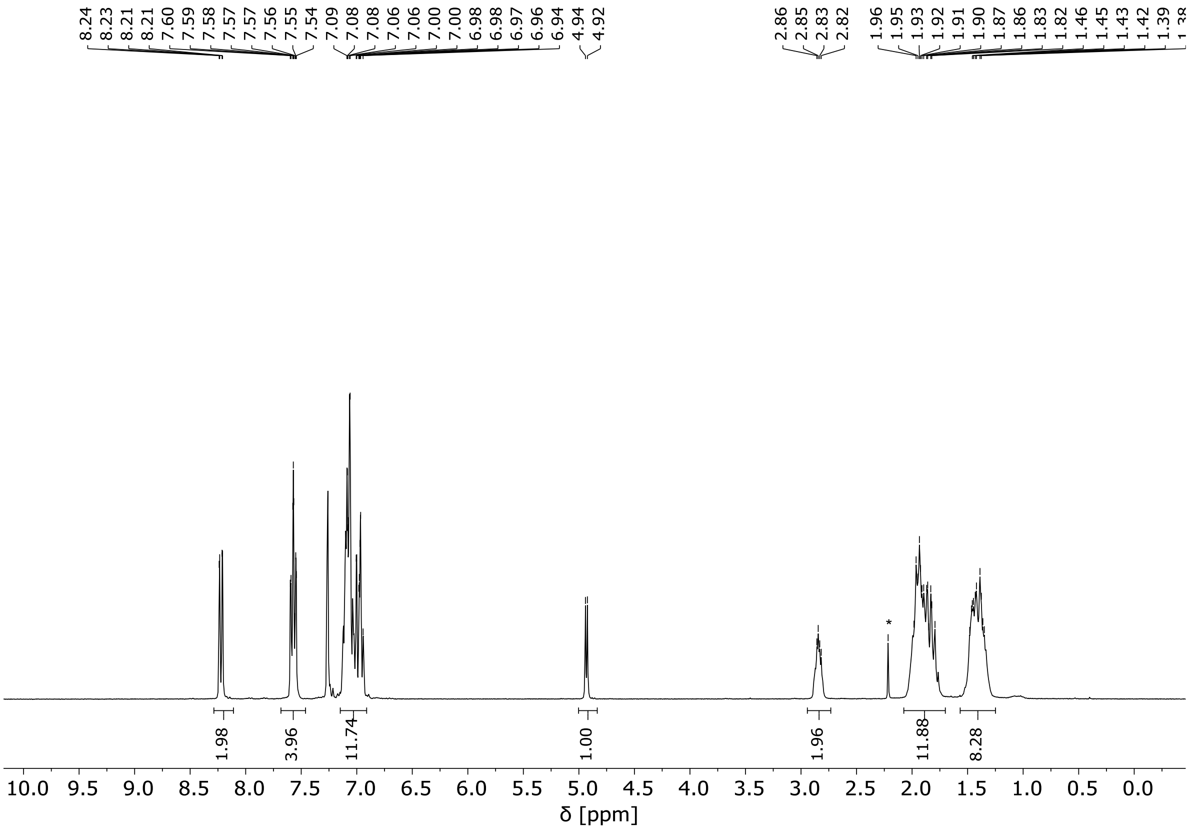

Figure S1: ^{1}H NMR spectrum of **1** in $CDCl_3$. Residual water is marked with a star (*).

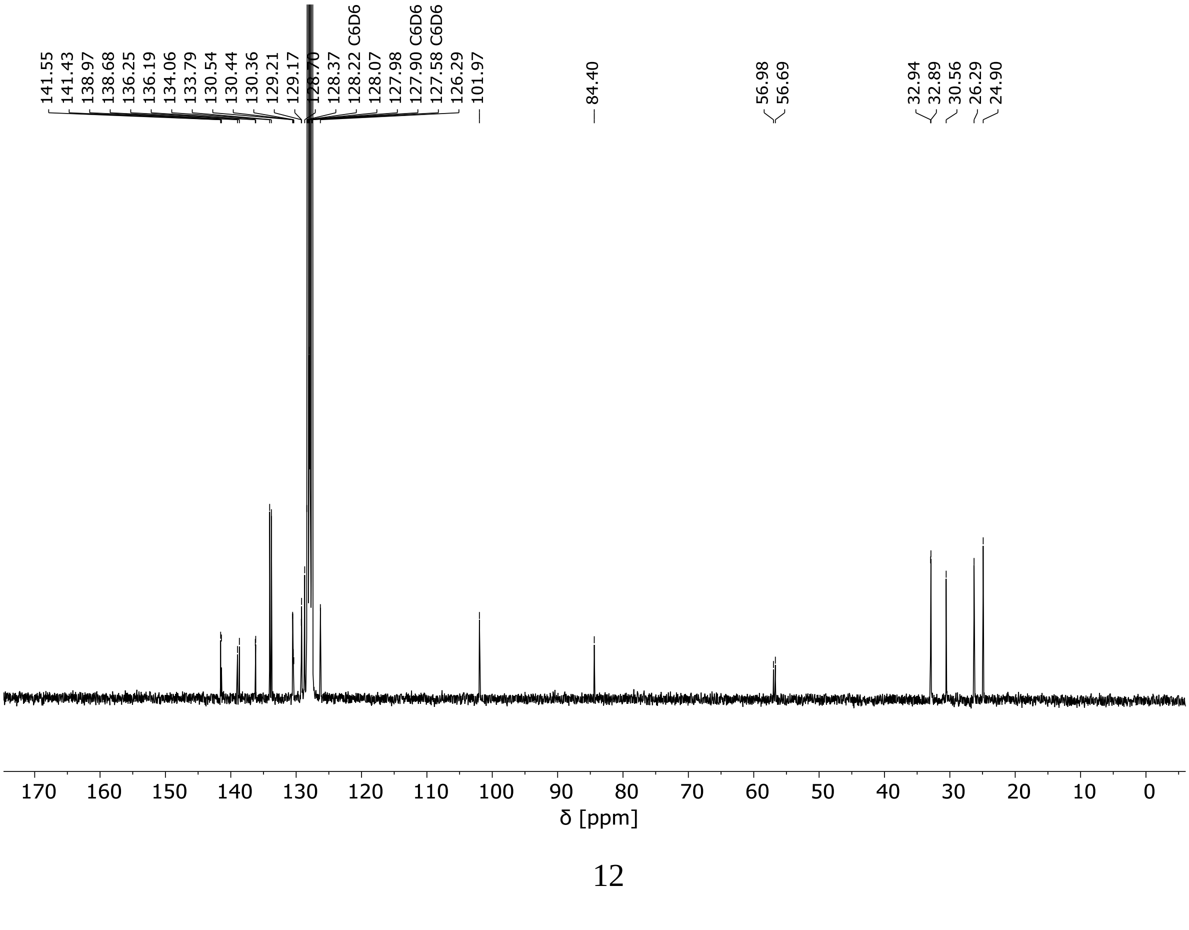

Figure S2**:** ^{13}C NMR spectrum of **1** in $CDCl_3$.

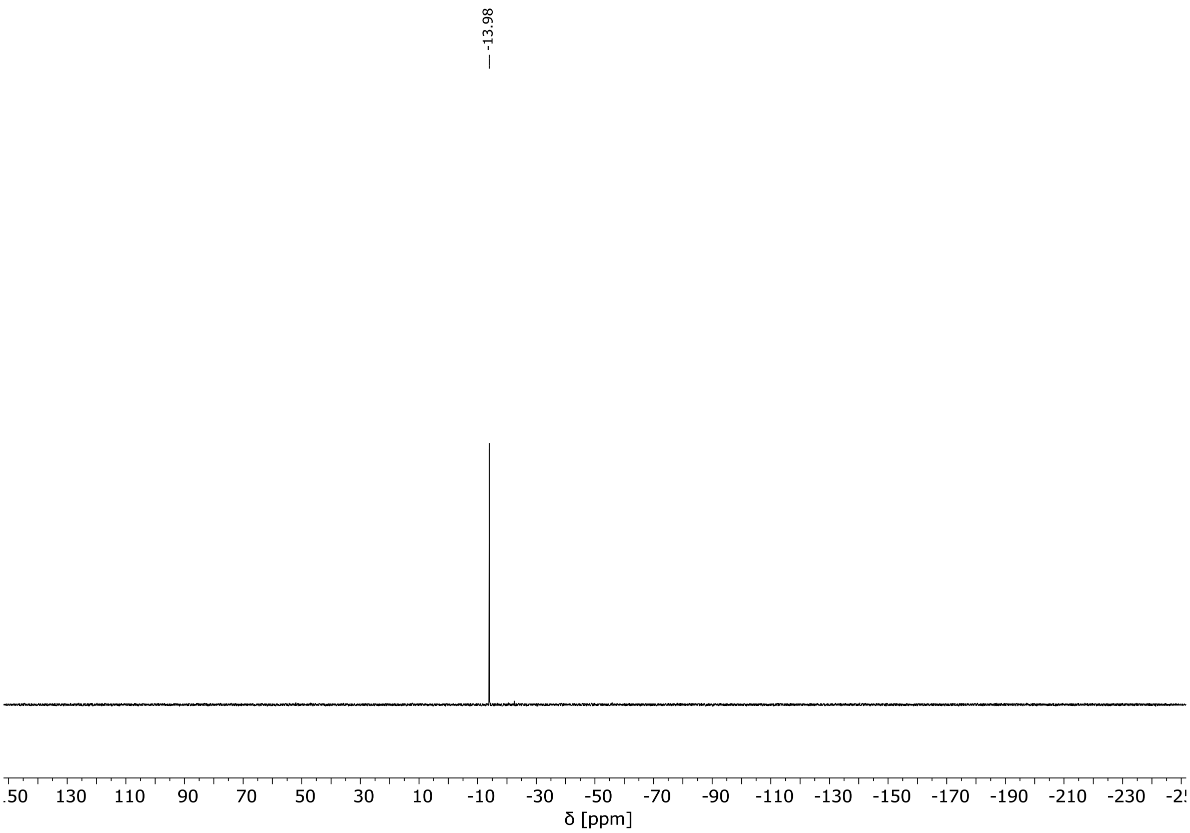

Figure S3: ^{31}P NMR spectrum of **1** in $CDCl_3$.

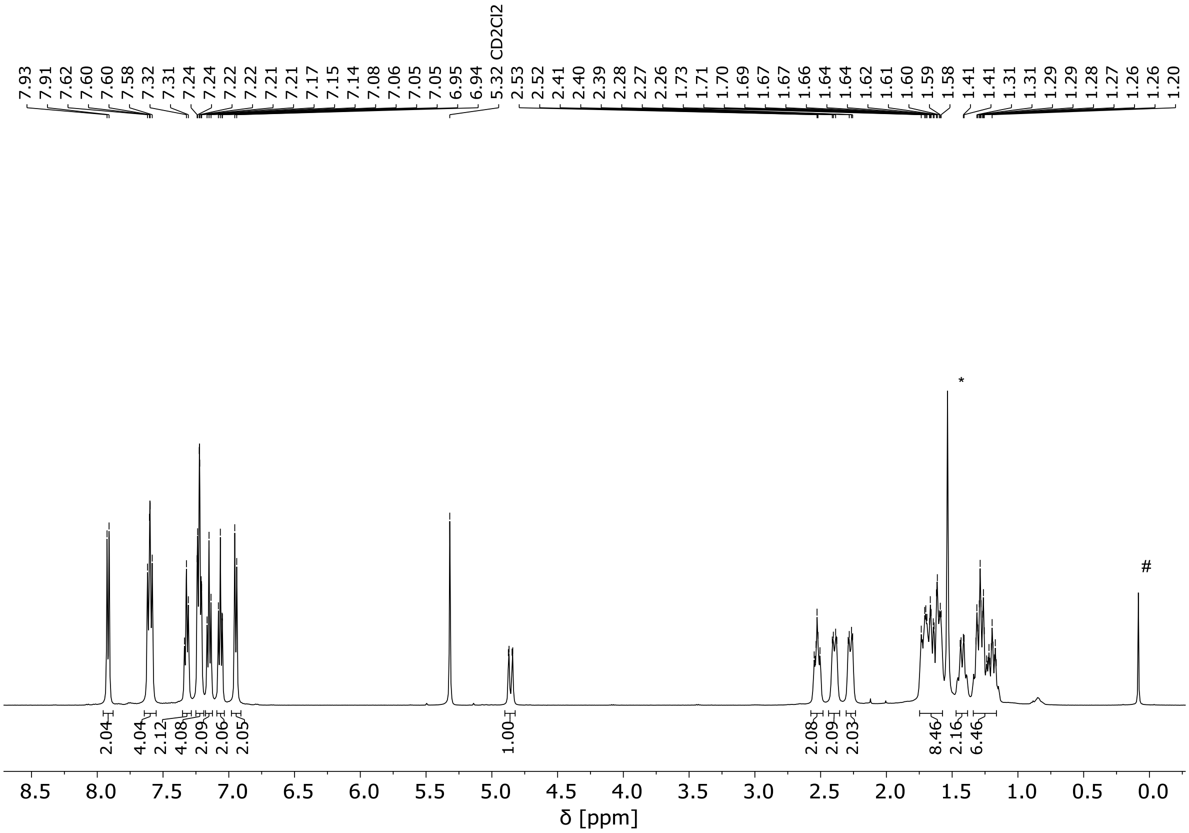

Figure S4**:** ^{1}H NMR spectrum of [**2**] in CD_2Cl_2. Residual water is marked with a star (*), silicon grease with a hashtag (#).

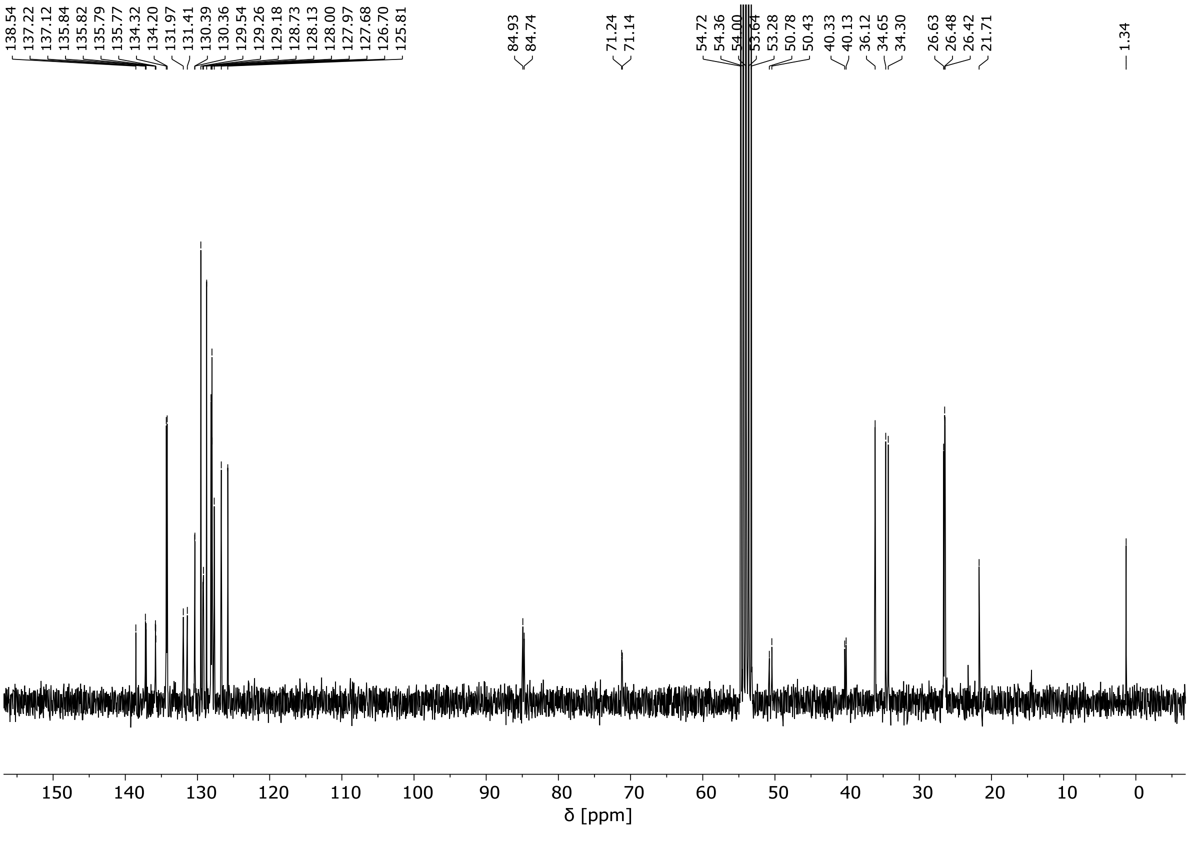

Figure S5: ^{13}C NMR spectrum of [**2**] in CD_2Cl_2.

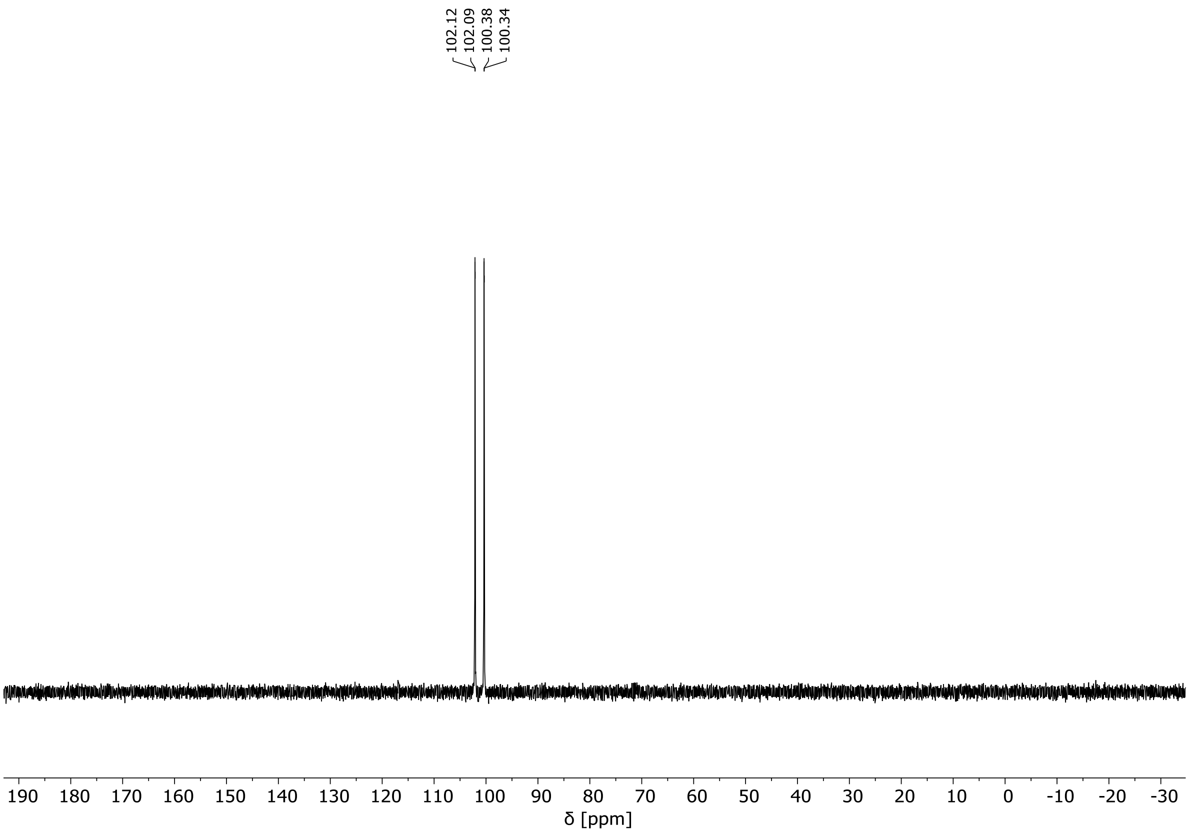

Figure S6: ^{31}P NMR spectrum of [**2**] in CD_2Cl_2.

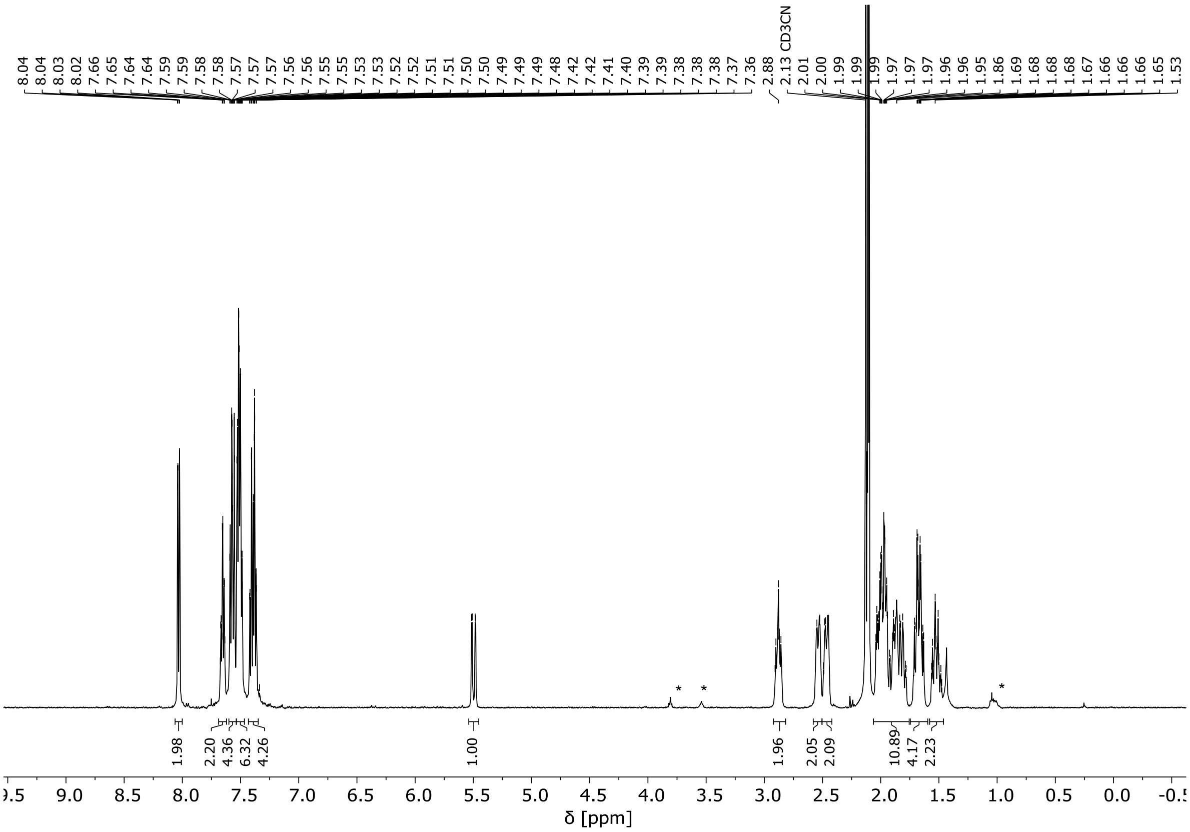

Figure S7**:** ^{1}H NMR spectrum of [**3**] in CD_3CN. Residual solvent is marked with a star (*).

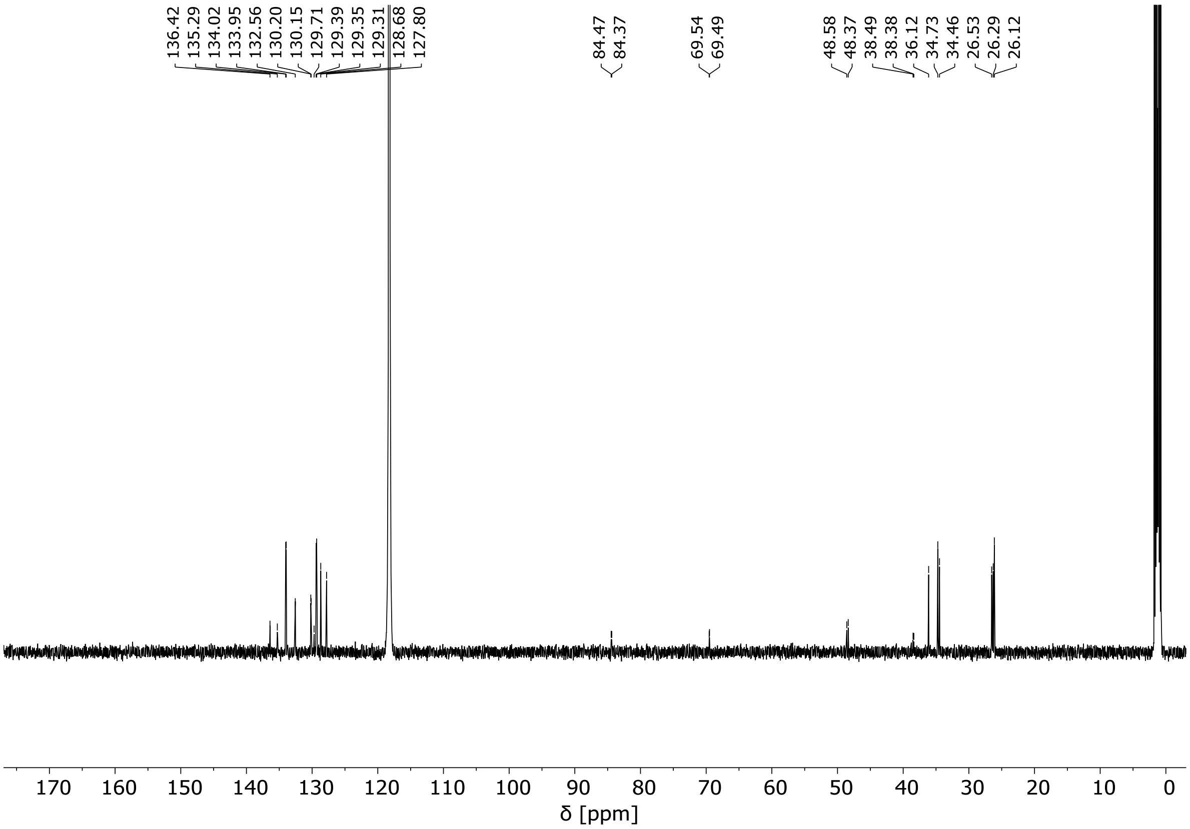

Figure S8**:** ^{13}C NMR spectrum of [**3**] in CD_3CN.

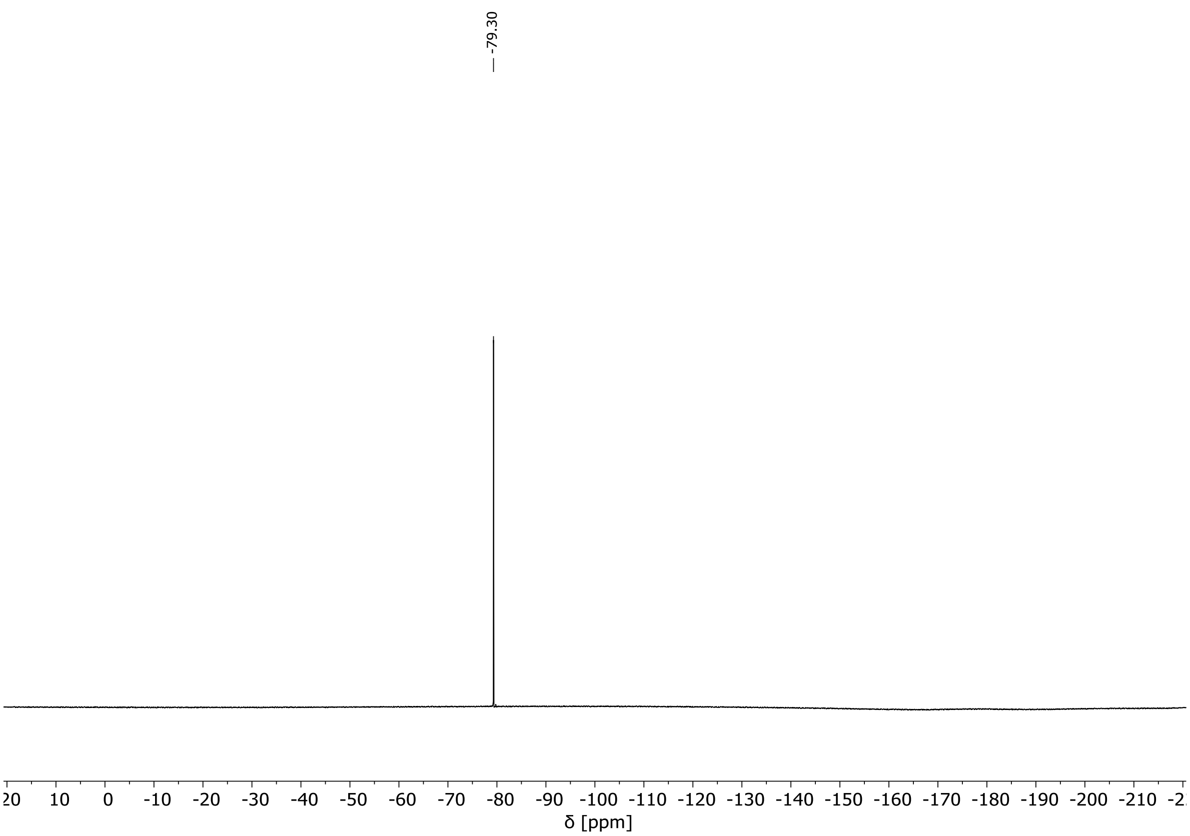

Figure S9**:** ^{19}F NMR spectrum of [**3**] in CD_3CN.

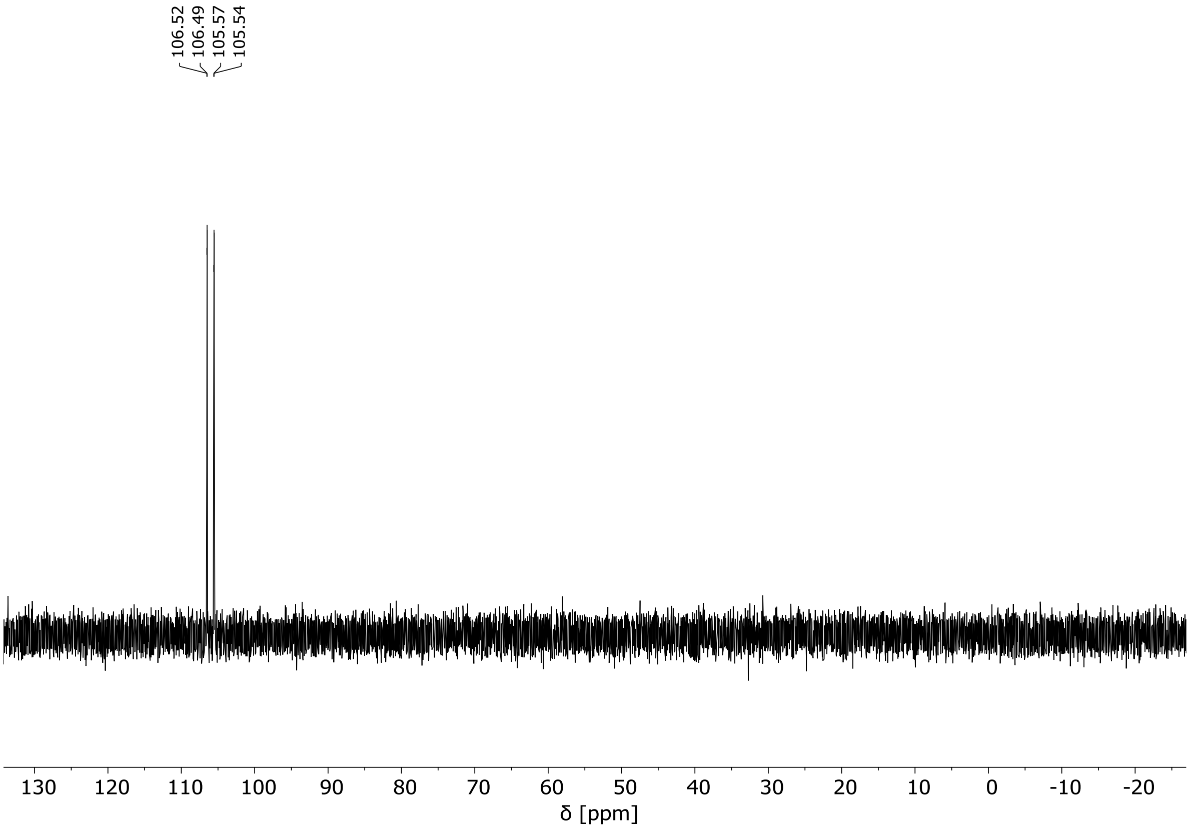

Figure S10: ^{31}P NMR spectrum of [**3**] in CD_3CN.

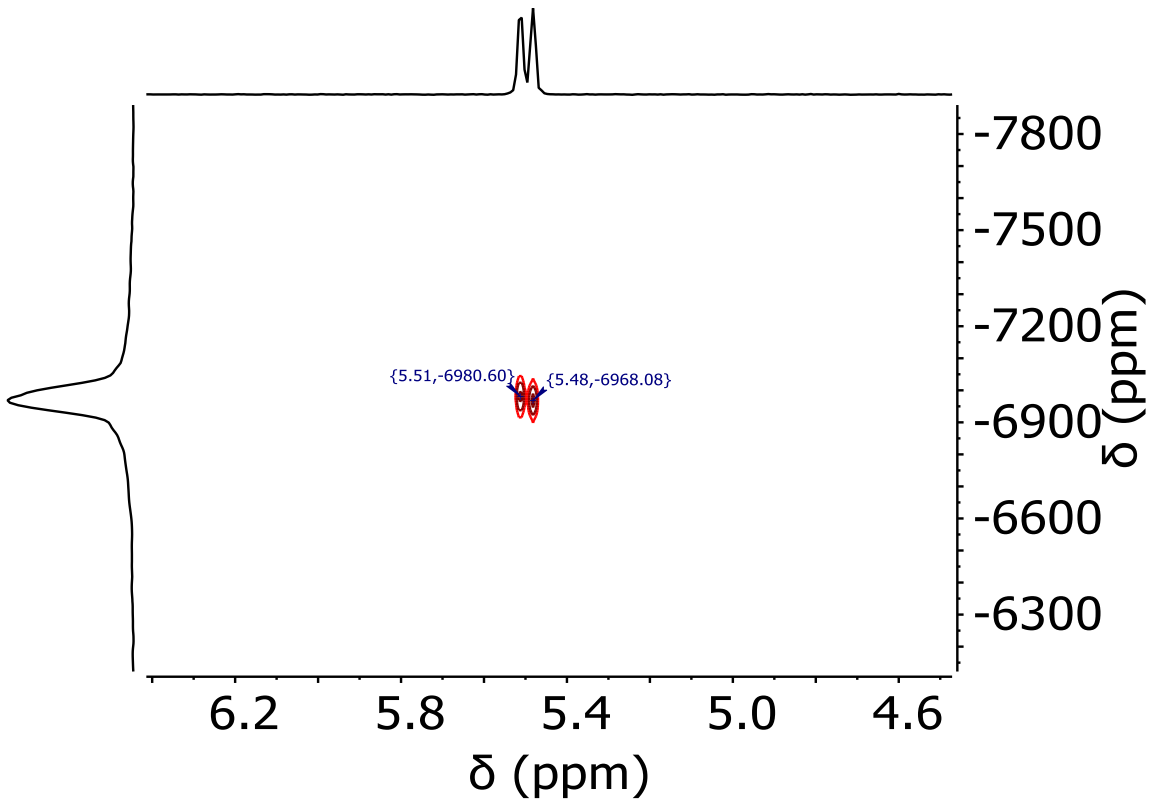

Figure S11**:** ^{103}Rh-^{1}H HMBC NMR spectrum of [**3**] in CD_3CN.

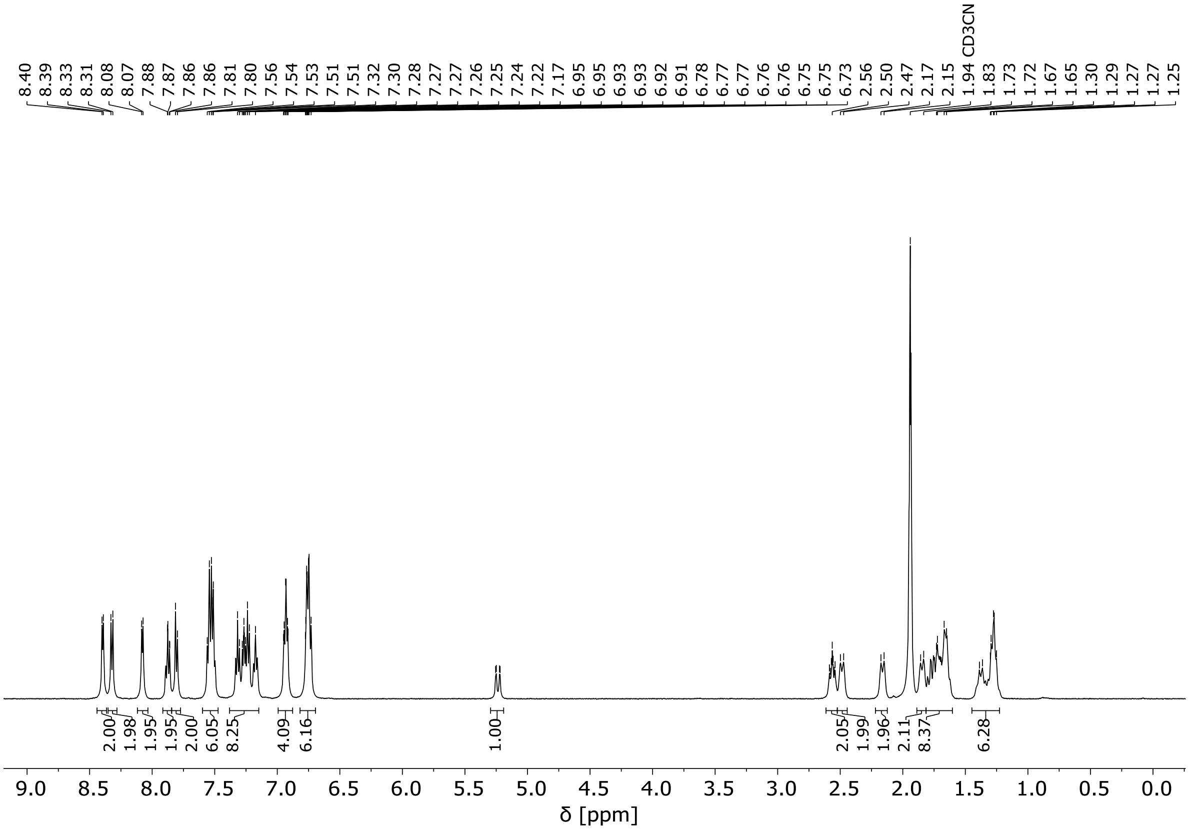

Figure S12**:** ^{1}H NMR spectrum of [**4**](OTf)$_2$ in CD_3CN.

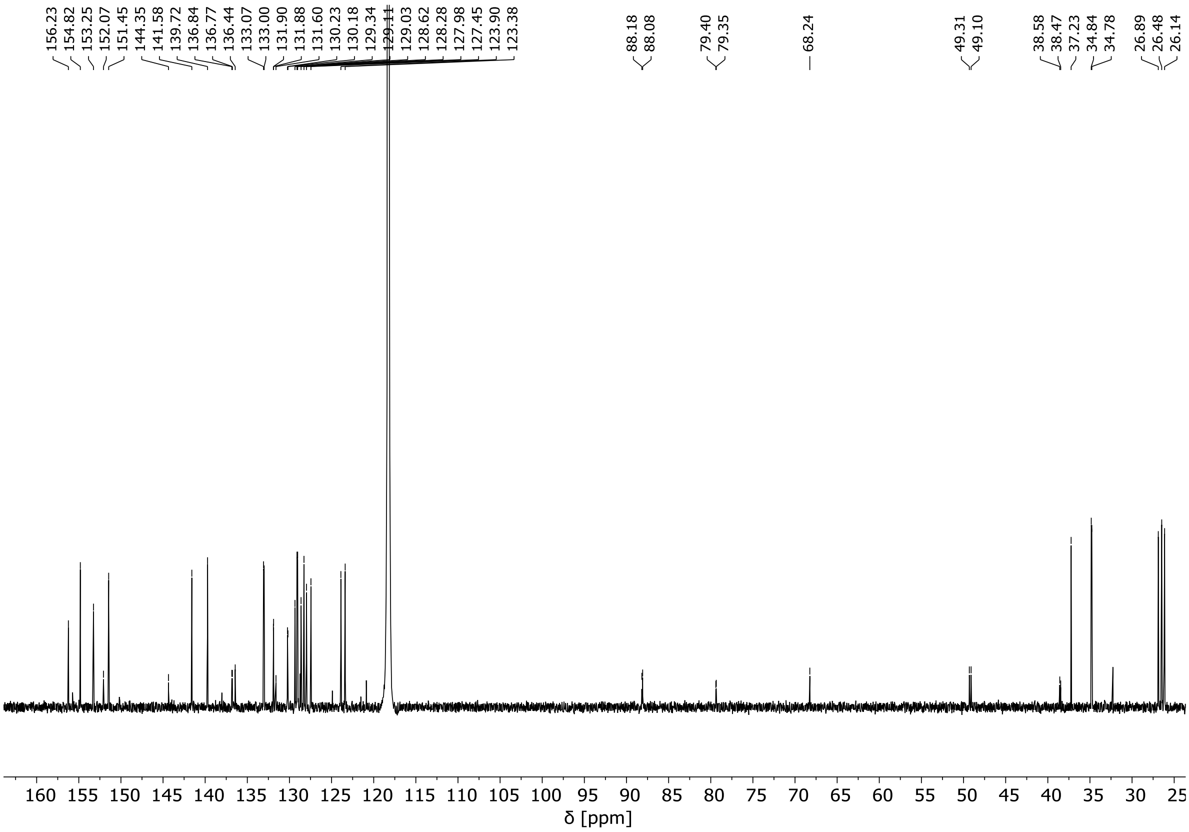

Figure S13**:** ^{13}C NMR spectrum of [**4**](OTf)$_2$ in CD_3CN.

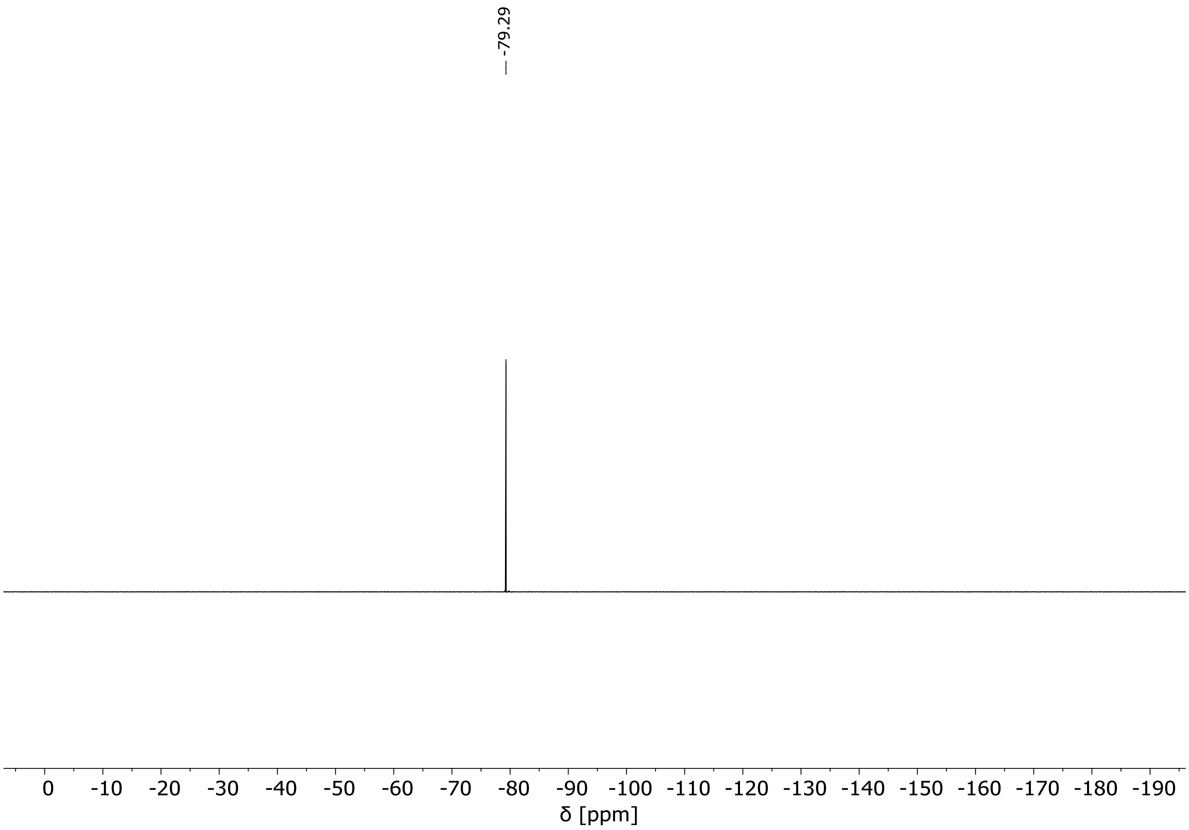

Figure S14**:** ^{19}F NMR spectrum of [**4**]$(OTf)_2$ in CD_3CN.

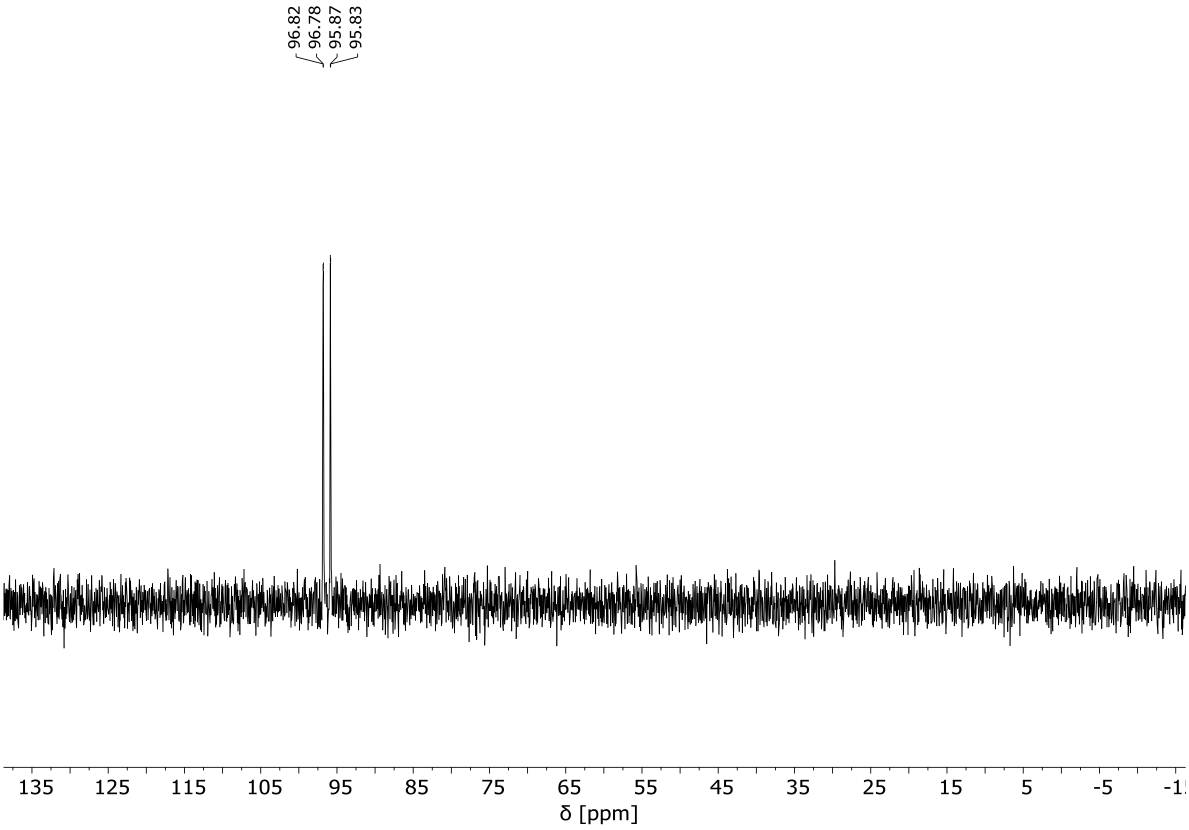

Figure S15**:** ^{31}P NMR spectrum of [**4**]$(OTf)_2$ in CD_3CN.

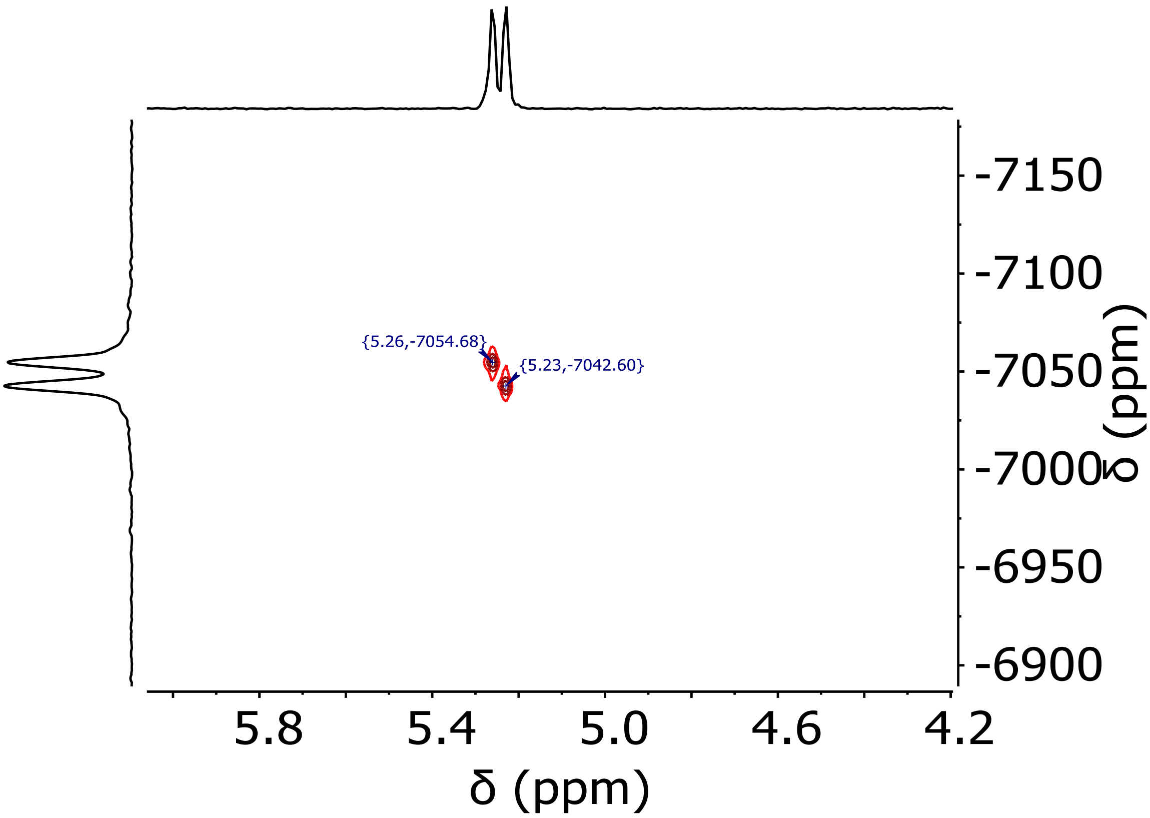

Figure S16**:** ^{103}Rh-^{1}H HMBC NMR spectrum of [**4**]$(OTf)_2$ in CD_3CN.

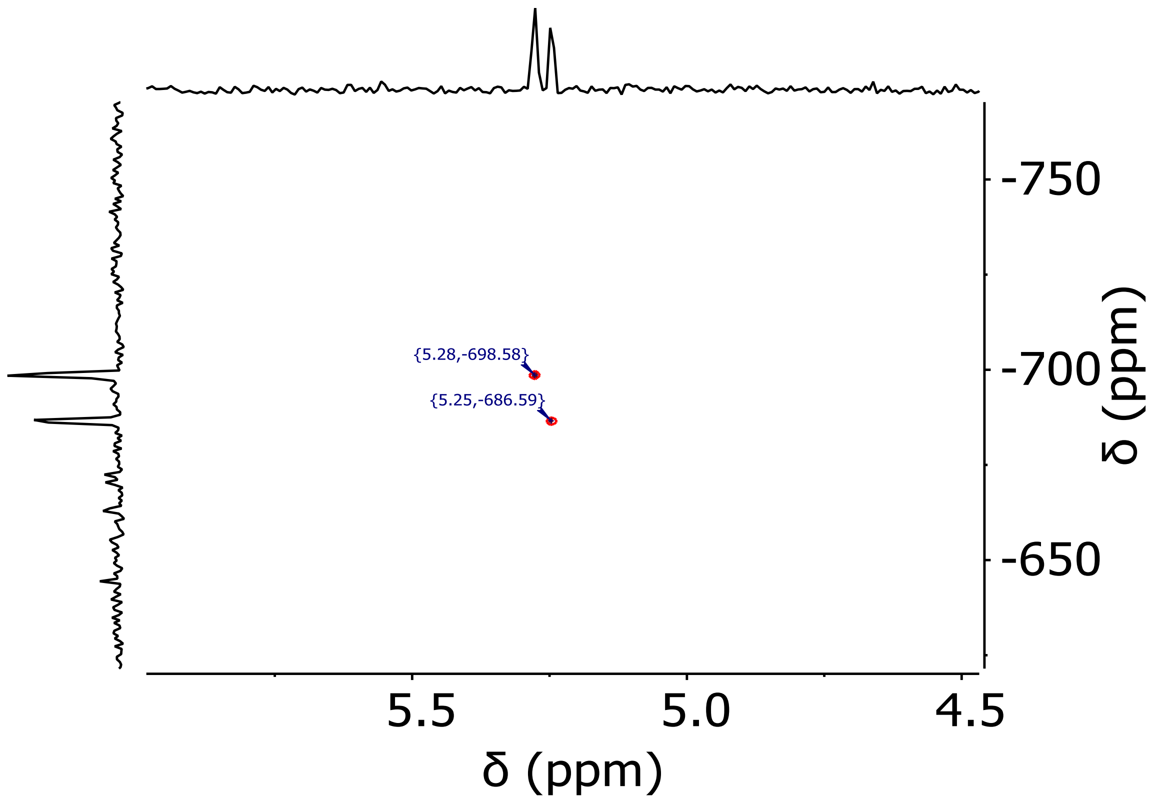

Figure S17**:** ^{103}Rh-^{1}H HMBC NMR spectrum of [**4**](OTf)$_2$ in CD_3CN.

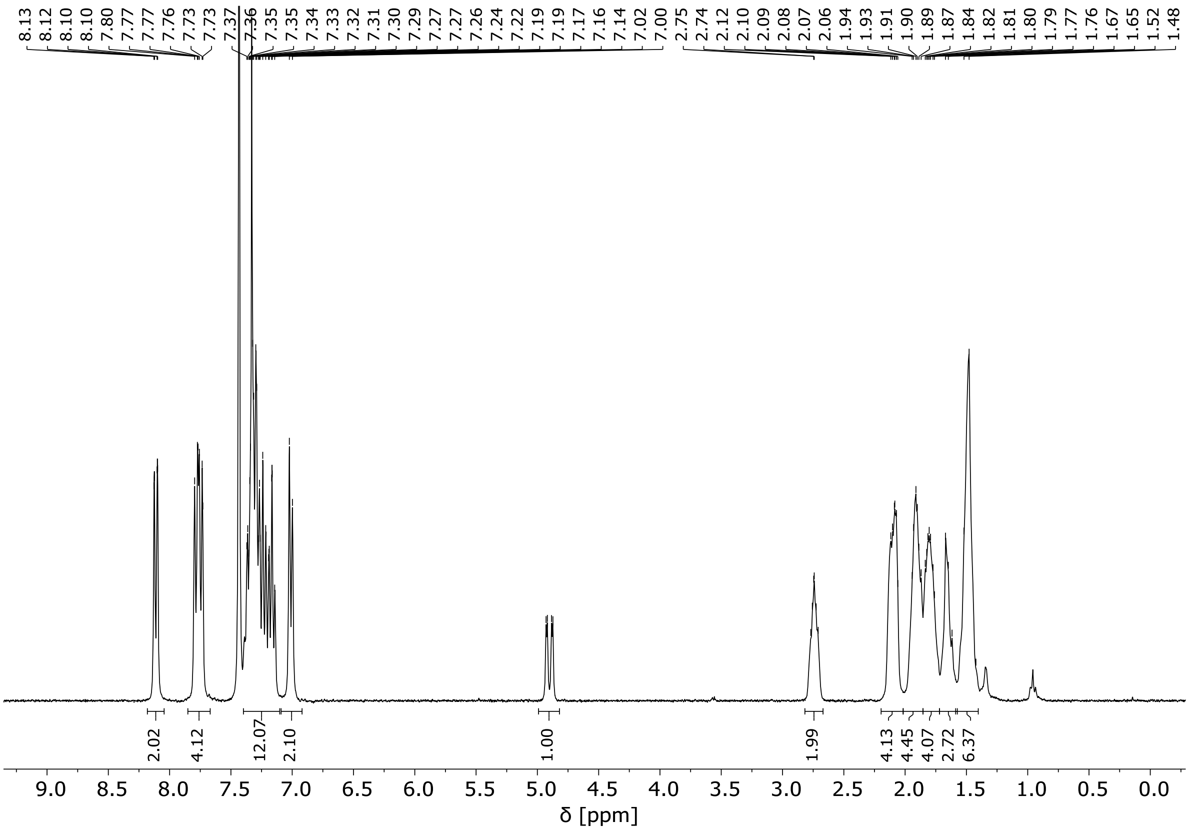

Figure S18**:** ^{1}H NMR spectrum of [**5**] in $CDCl_3$.

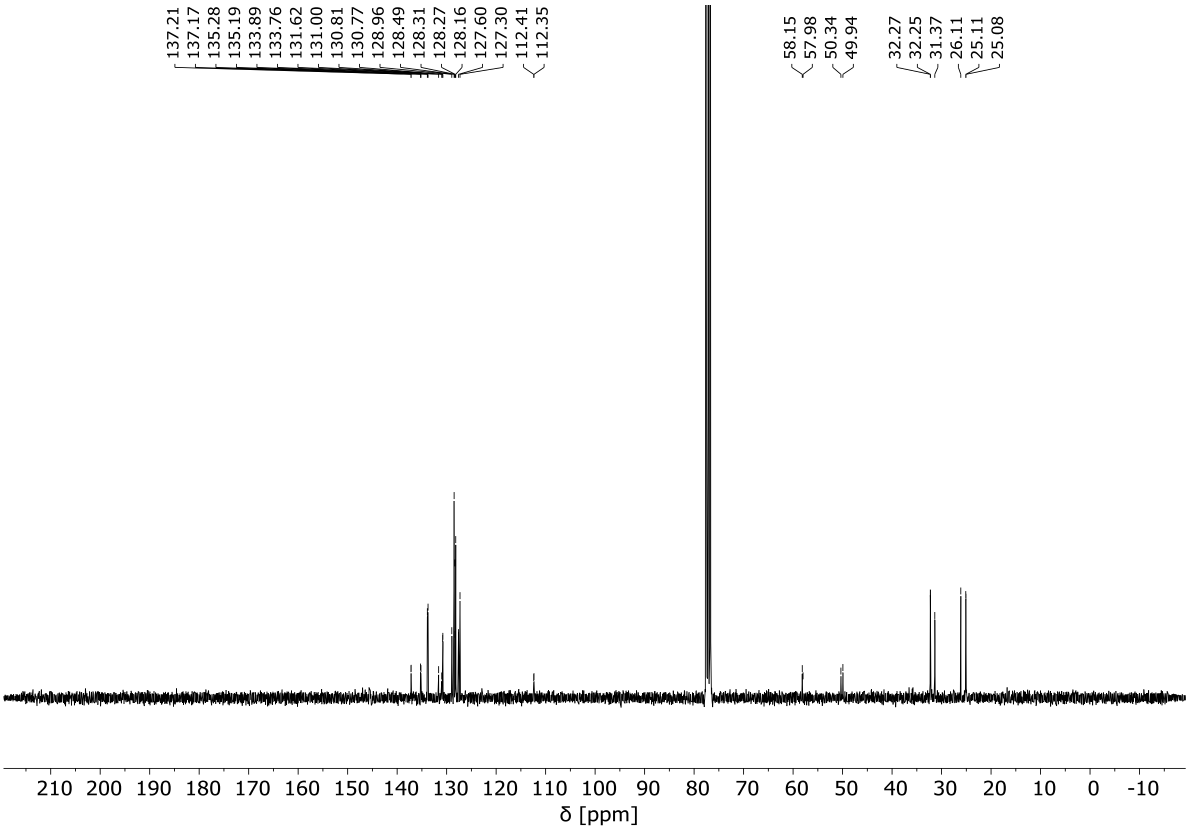

Figure S19**:** ^{13}C NMR spectrum of [**5**] in $CDCl_3$.

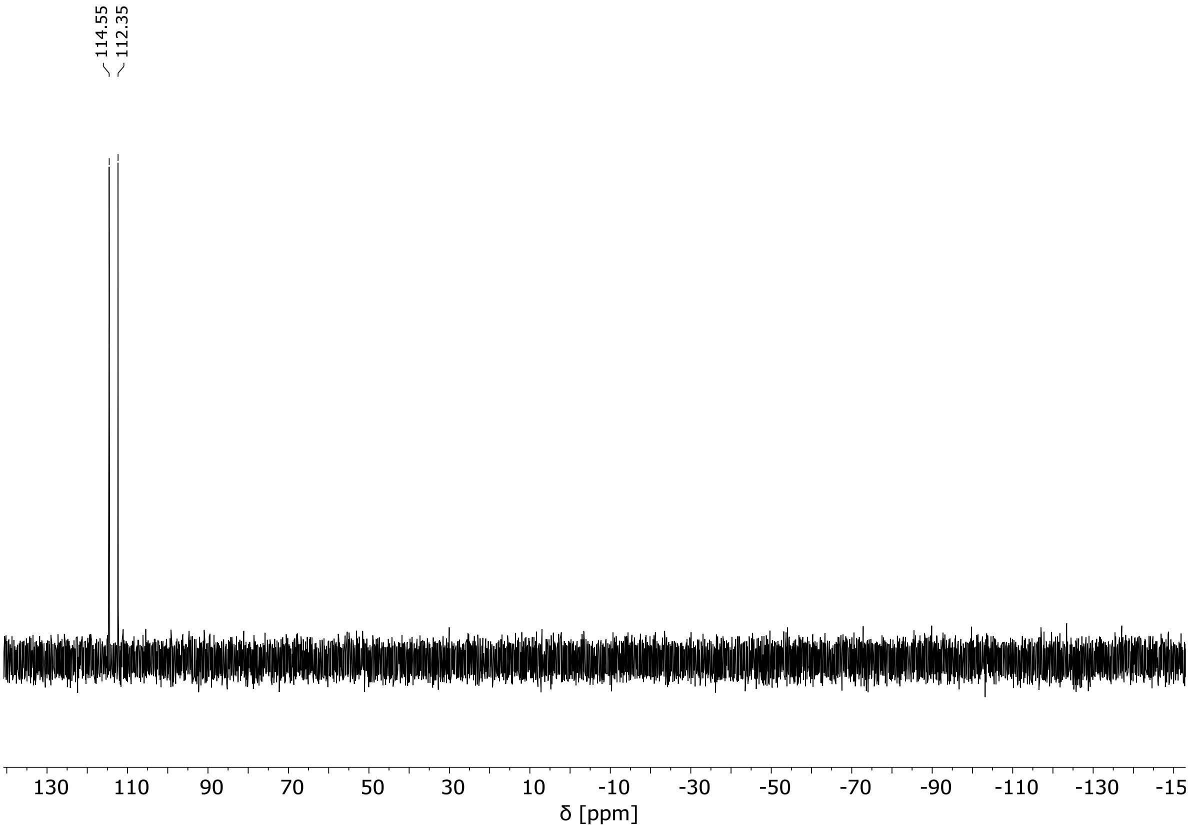

Figure S20**:** ^{31}P NMR spectrum of [**5**] in $CDCl_3$.

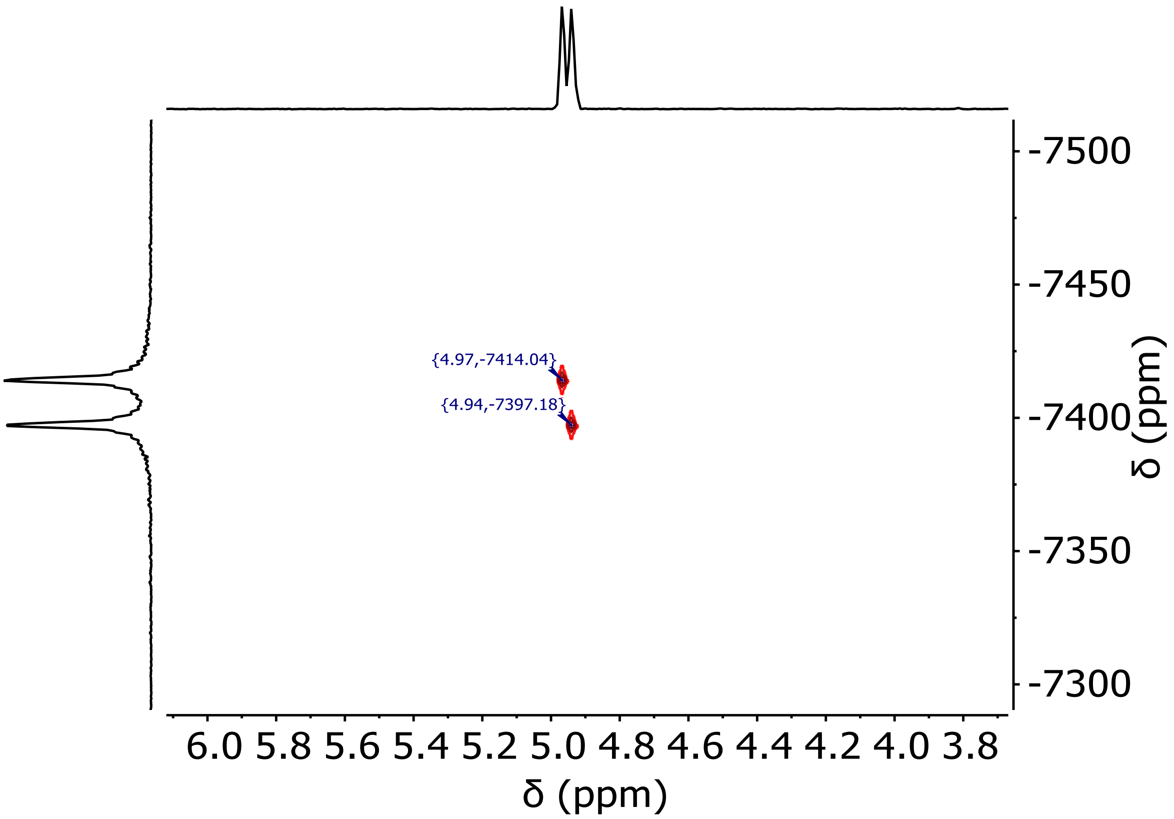

Figure S21: ^{103}Rh-^{1}H HMBC NMR spectrum of [**5**] in CD_2Cl_2.

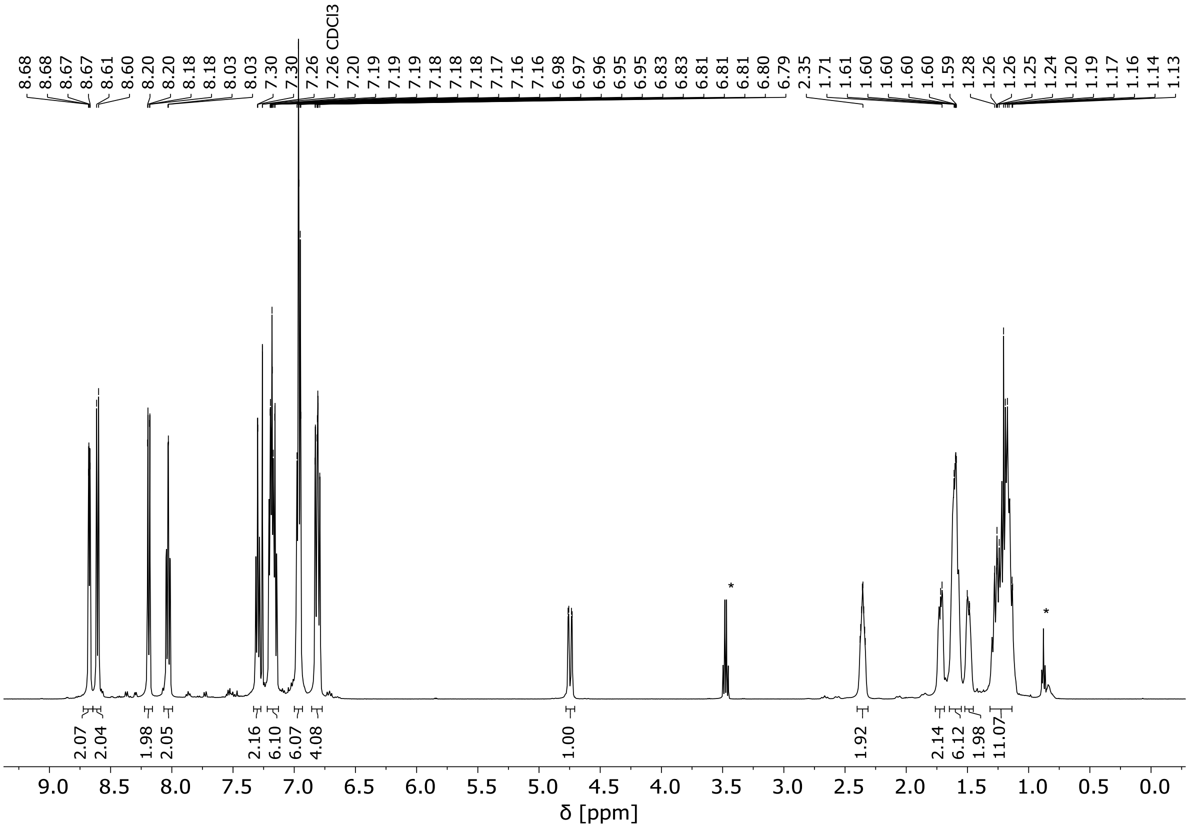

Figure S 22: ^{1}H NMR spectrum of [**6**](OTf) in $CDCl_3$. Residual solvent (Et_2O, hexane) is marked with a star (*).

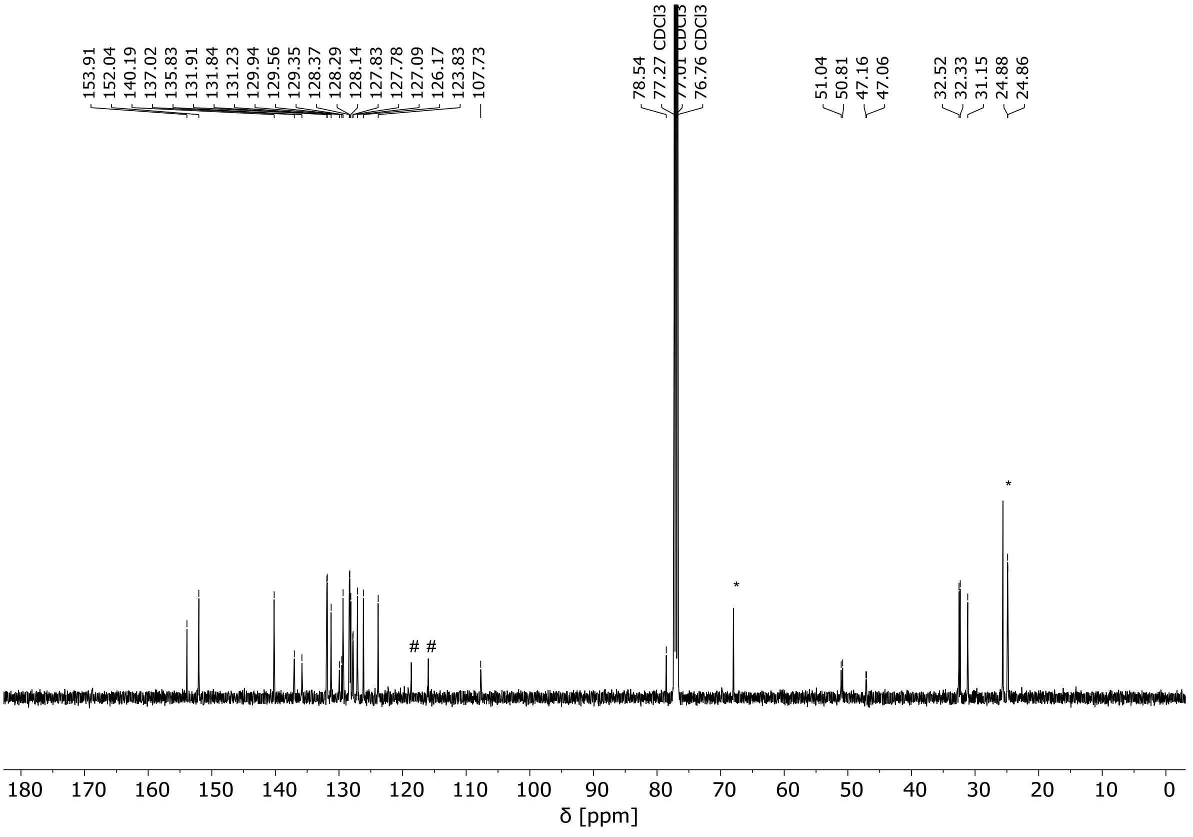

Figure S23: ^{13}C NMR spectrum of [**6**](OTf) in $CDCl_3$, added $CFCl_3$ standard is marked with a hashtag (#), residual THF is marked with a star (*).

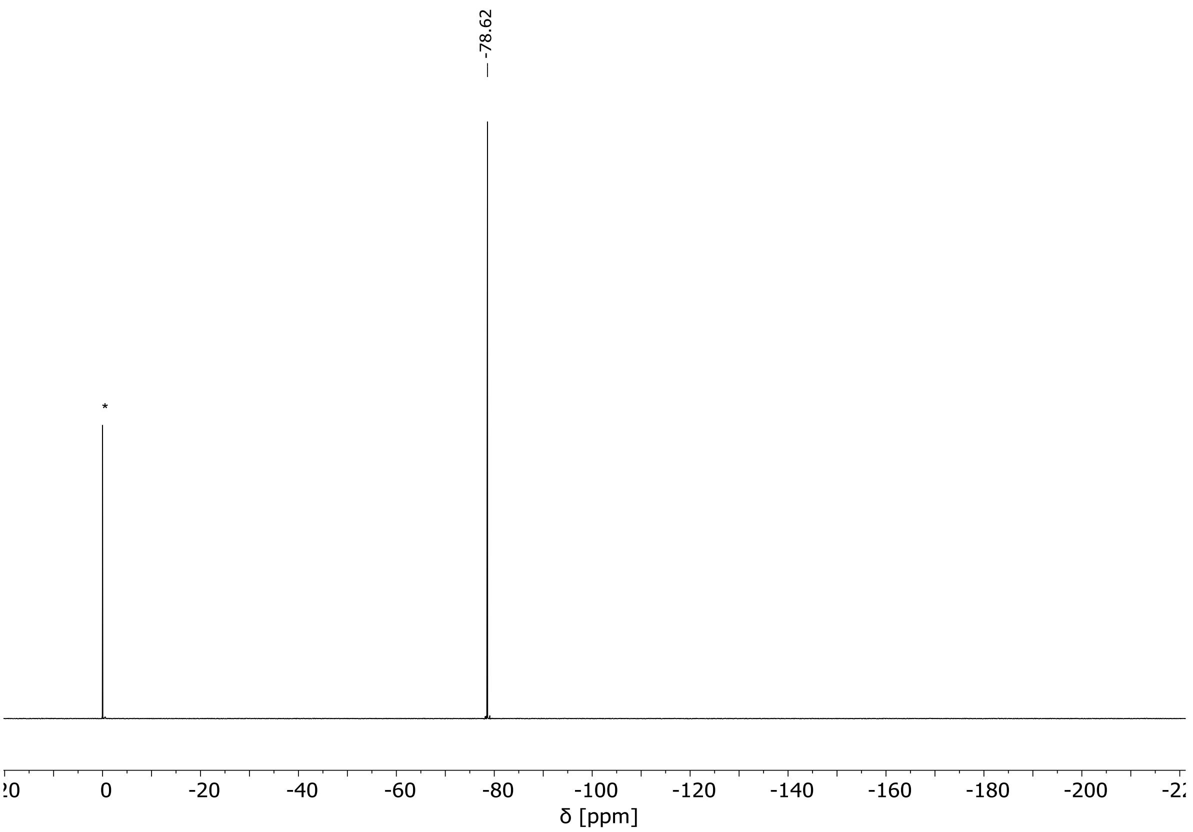

Figure S24: ^{19}F NMR spectrum of [**6**](OTf) in $CDCl_3$, added $CFCl_3$ standard is marked with a star (*).

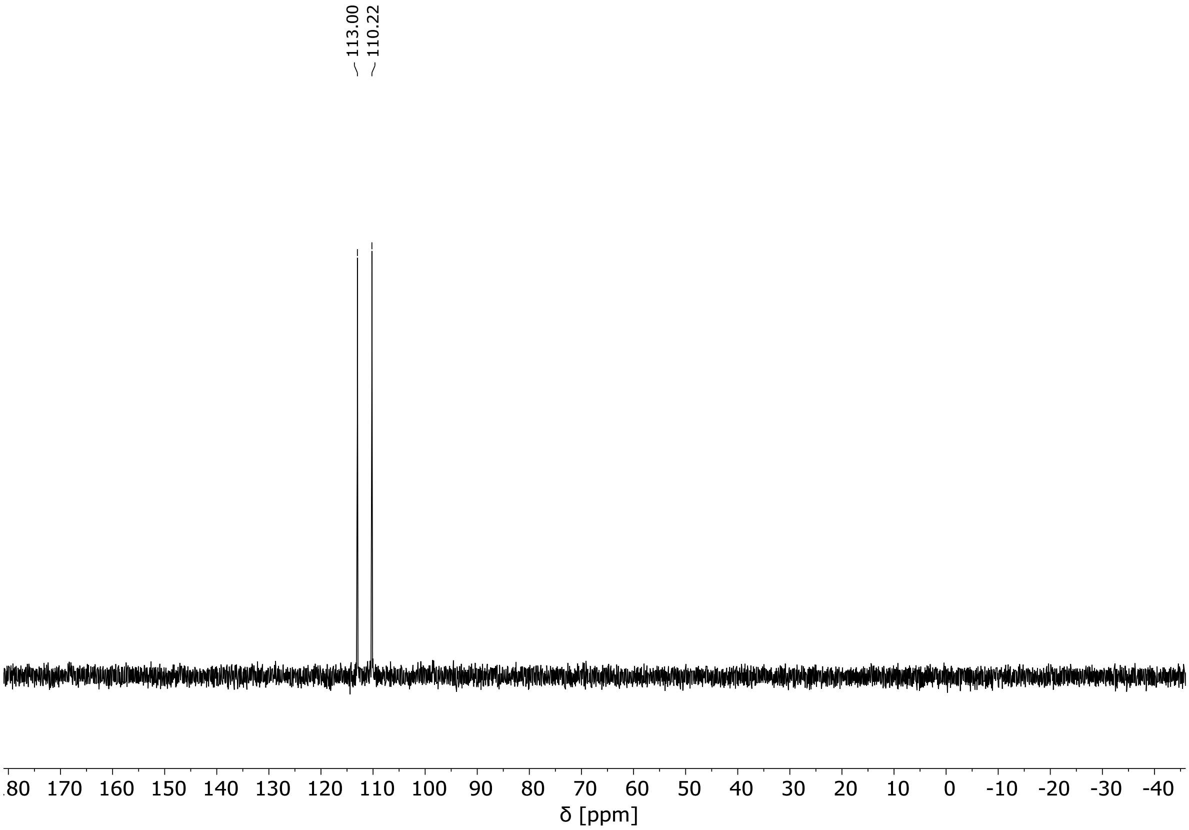

Figure S25**:** ^{31}P NMR spectrum of [**6**](OTf) in THF-d_8.

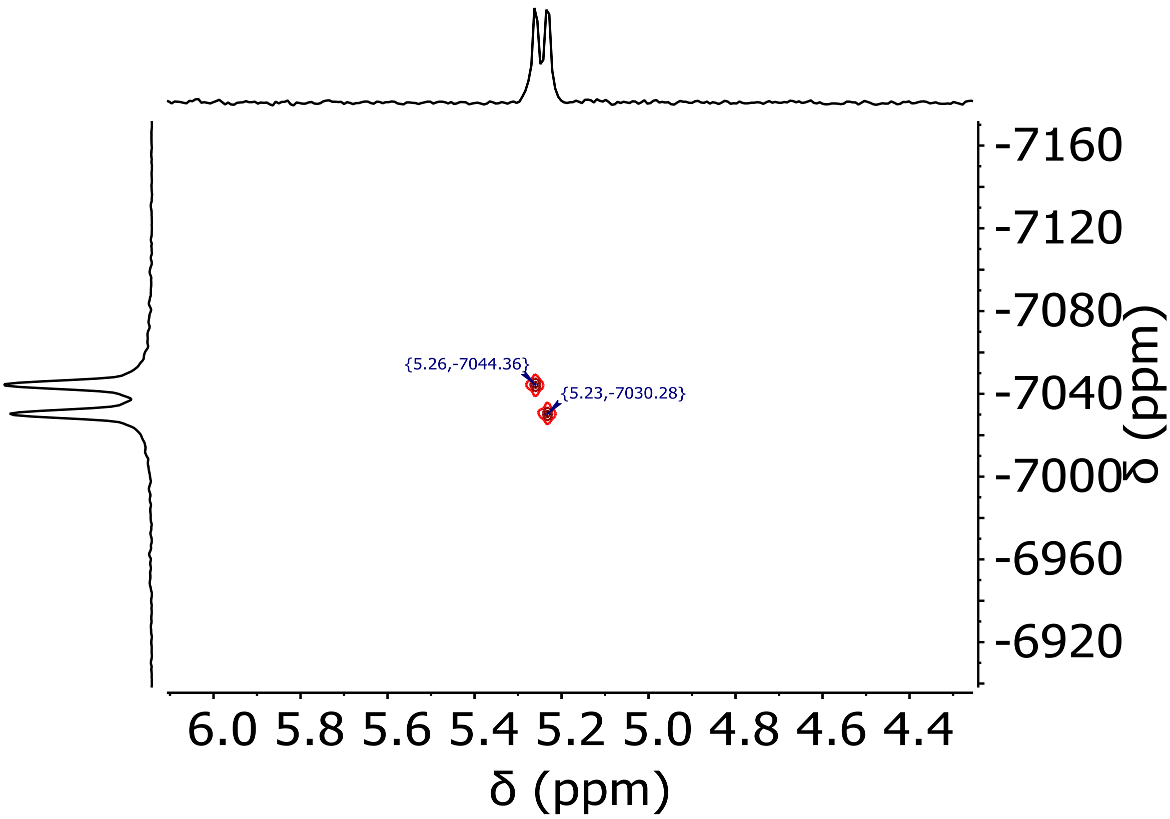

Figure S26**:** ^{103}Rh-^{1}H HMBC NMR spectrum of [**6**](OTf) in $CDCl_3$.

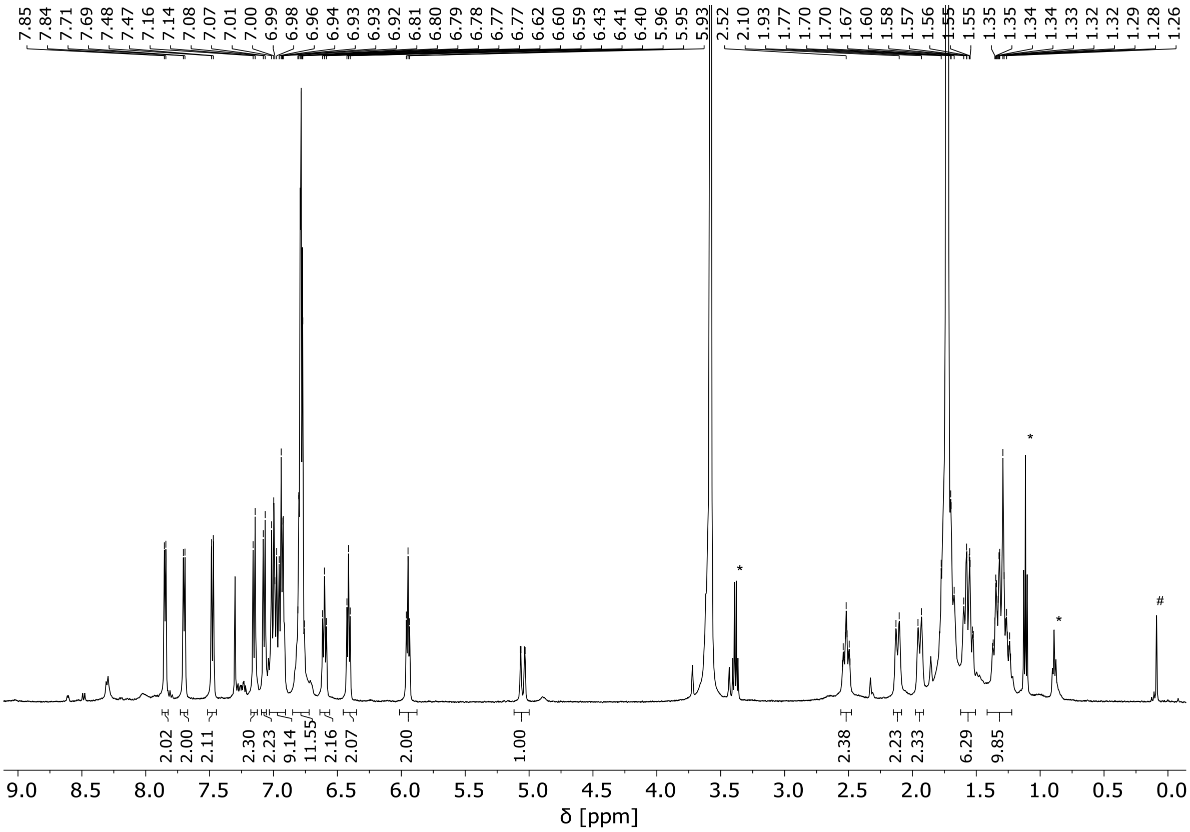

Figure S27**:** ^{1}H NMR spectrum of [**7**] in THF-d_8. Residual solvent (Et_2O, hexane) is marked with a star (*), silicon grease with (#).

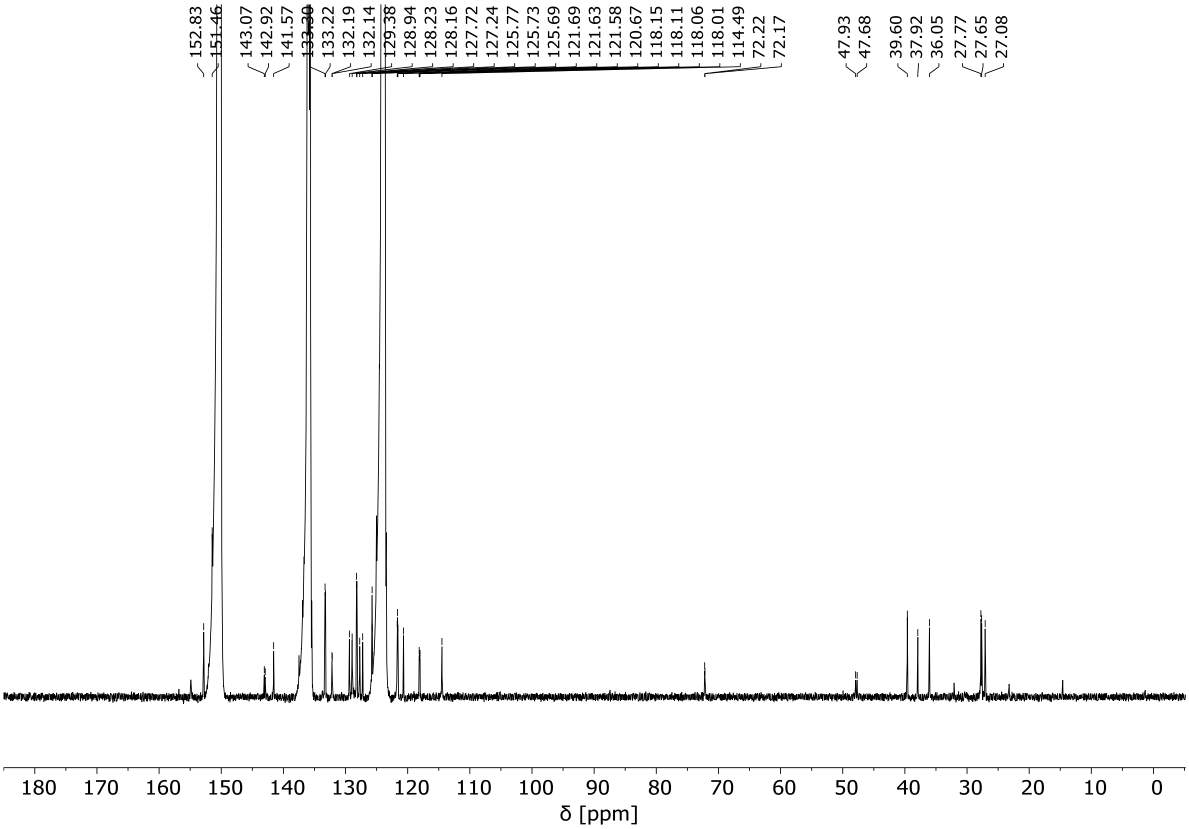

Figure S28**:** ^{13}C NMR spectrum of [**7**] in pyridine-d_5.

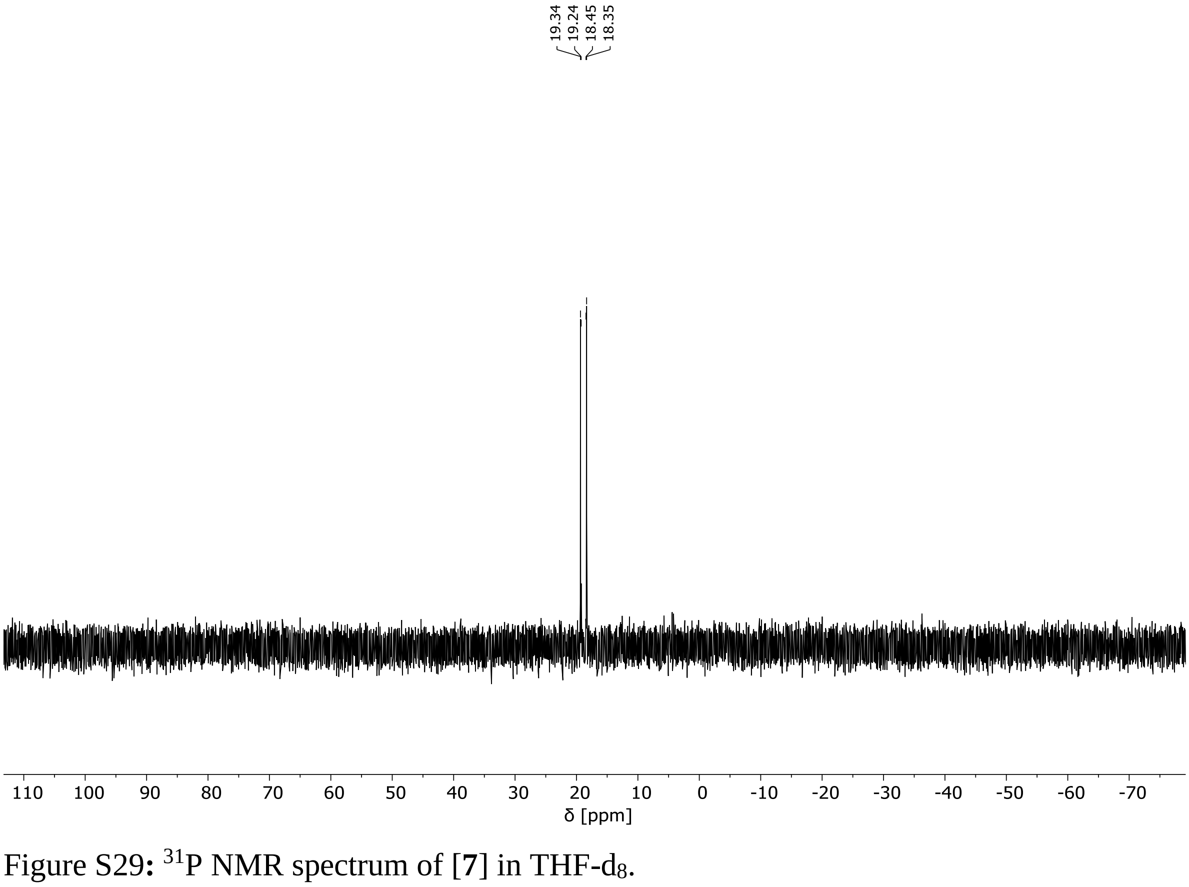

Figure S29**:** ^{31}P NMR spectrum of [**7**] in THF-d_8.

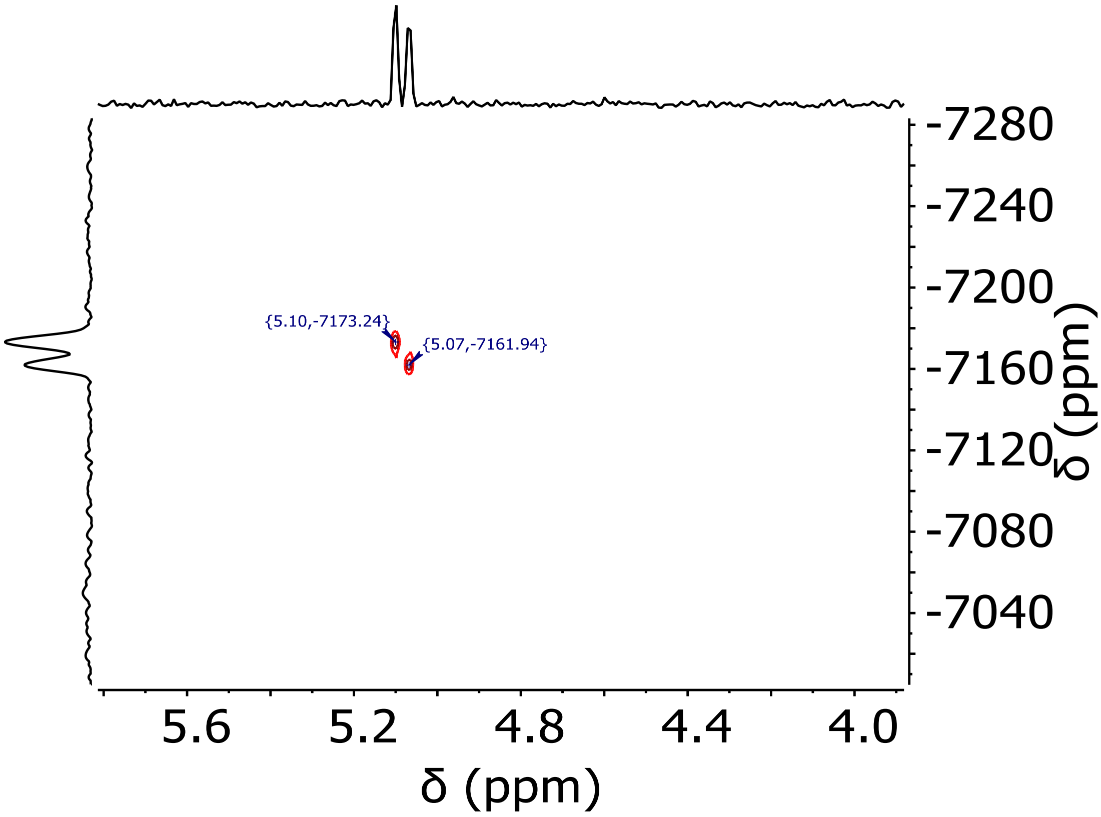

Figure S30**:** ^{103}Rh-^{1}H HMBC NMR spectrum of [**7**] in THF-d_8.

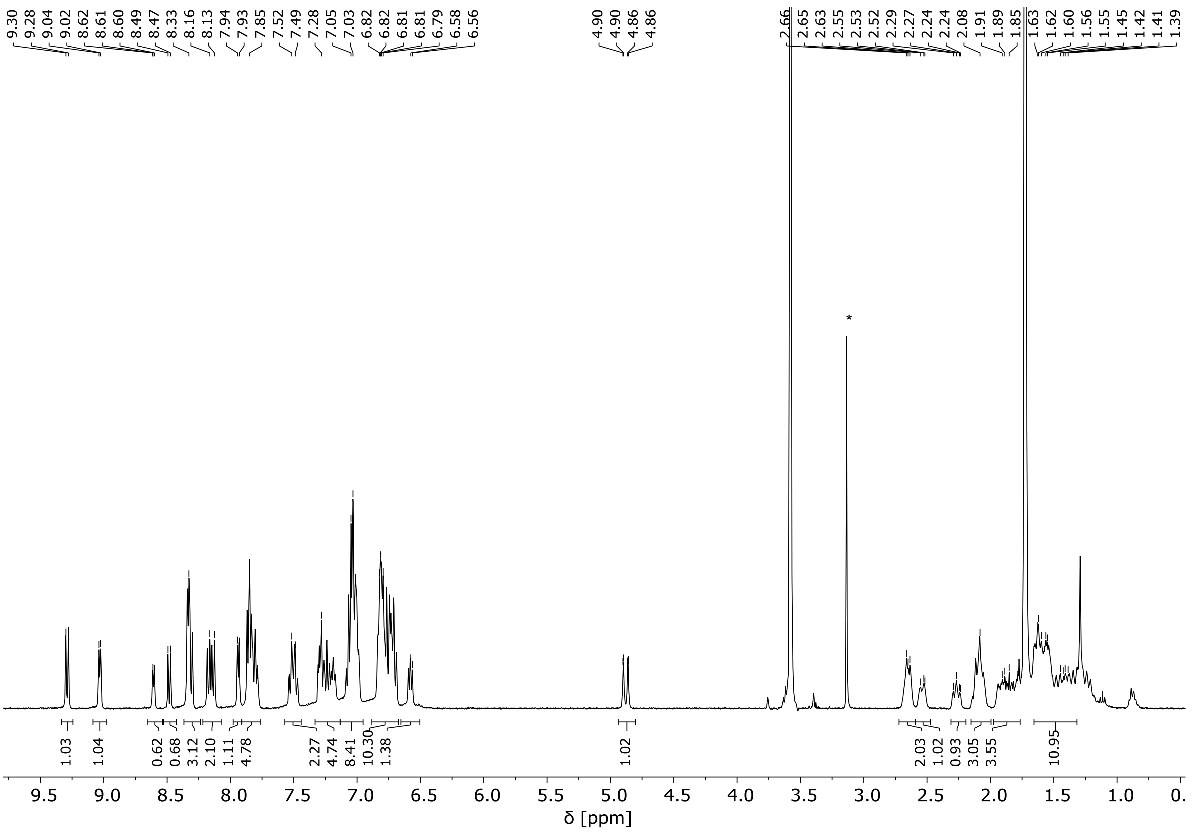

Figure S31**:** ^{1}H NMR spectrum of [**8**](OTf) in THF-d$_8$. Residual *t*BuO*H* is marked with a star (*).

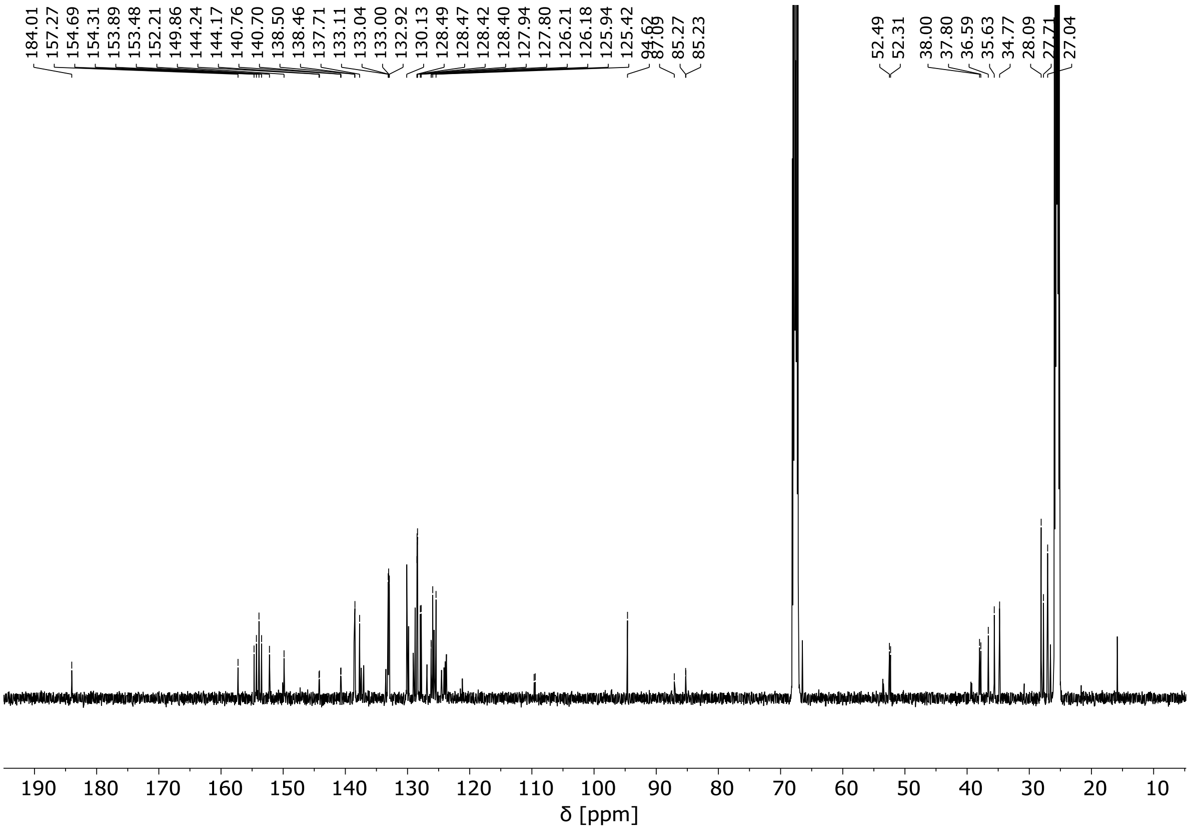

Figure S32**:** ^{13}C NMR spectrum of [**8**](OTf) in THF-d$_8$.

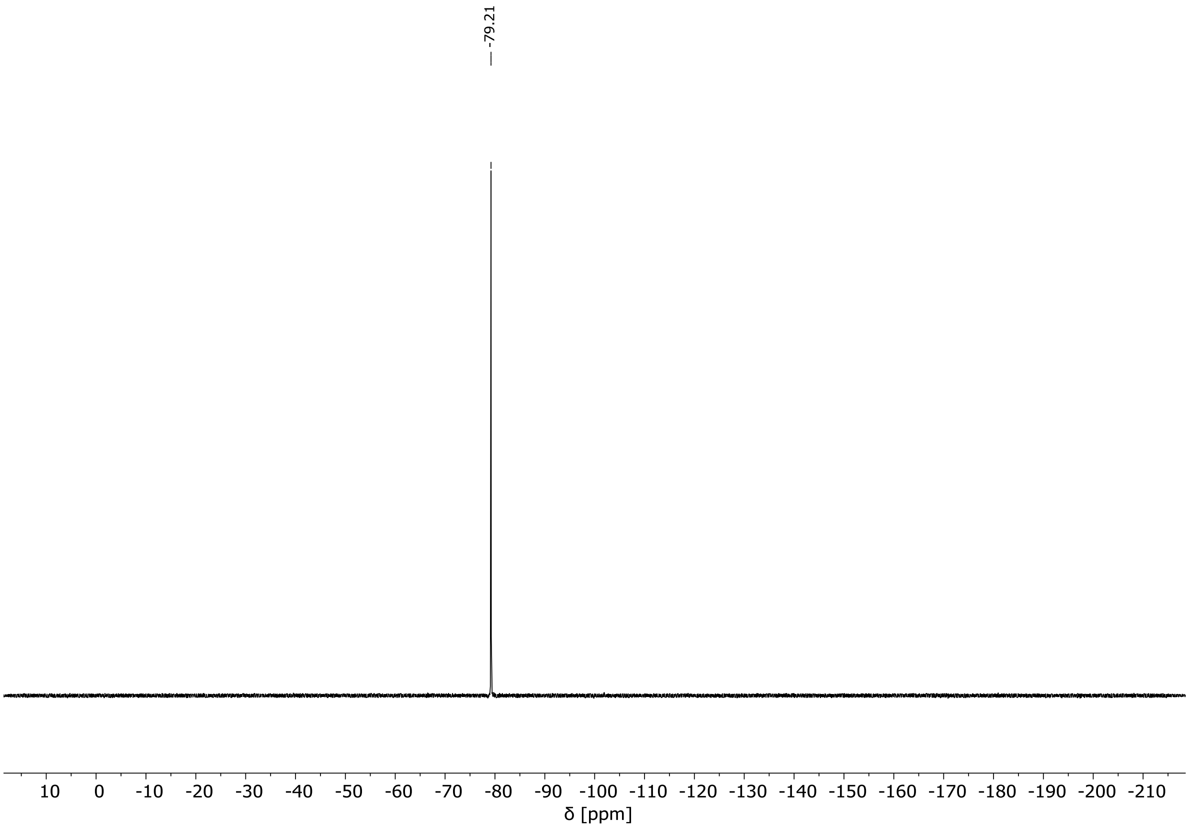

Figure S33**:** ^{19}F NMR spectrum of [**8**](OTf) in THF-d_8.

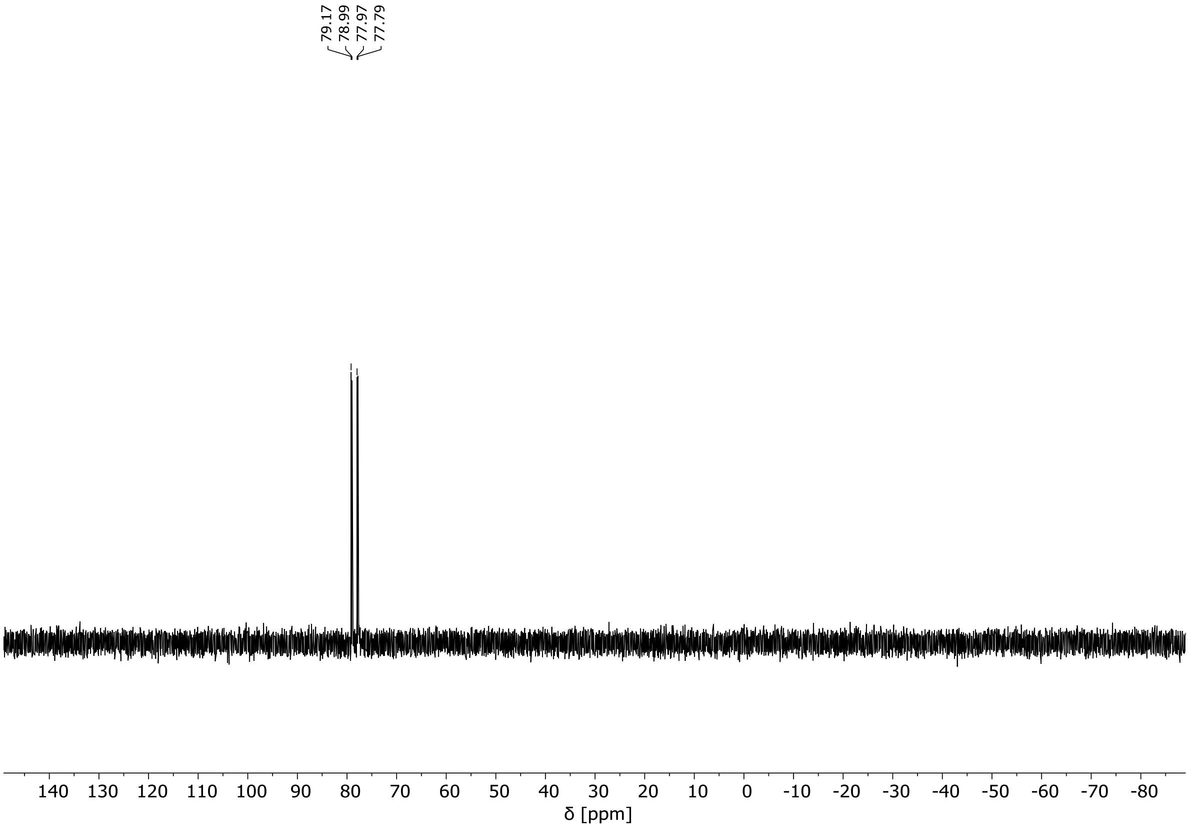

Figure S34**:** ^{31}P NMR spectrum of [**8**](OTf) in THF-d_8.

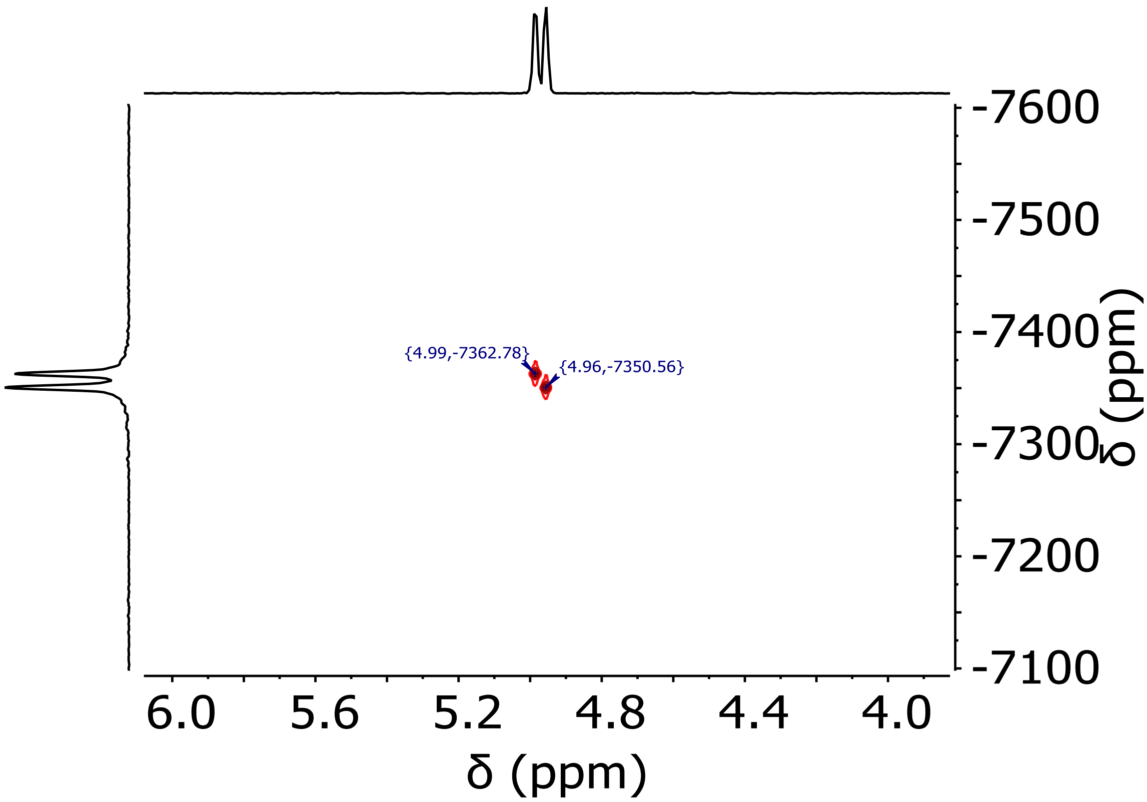

Figure S35**:** ^{103}Rh-^{1}H HMBC NMR spectrum of [**8**](OTf) in THF-d_8.

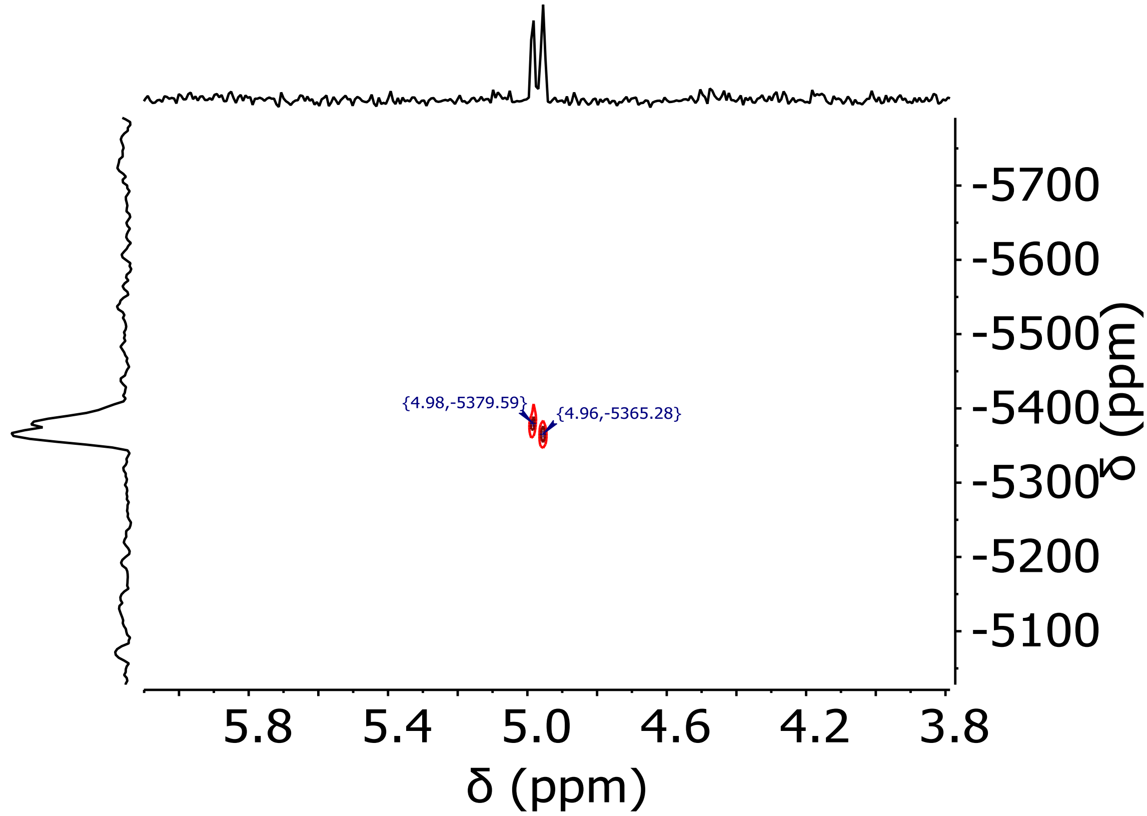

Figure S36**:** ^{103}Rh-^{1}H HMBC NMR spectrum of [**8**]$^{+}$ in THF-d$_8$.

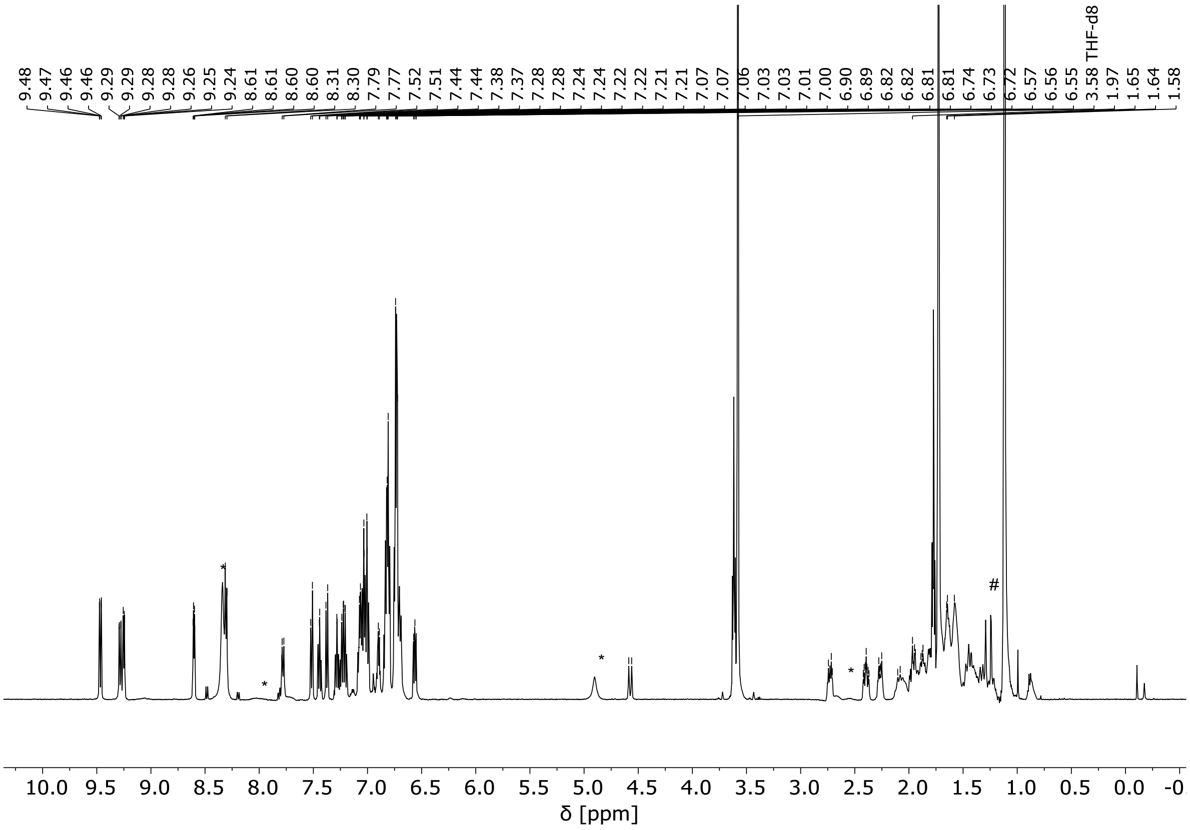

Figure S37**:** ^{1}H NMR spectrum of [**9**] in THF-d$_8$ (*in situ*). Residual complex [**8**](OTf) is marked with a star (*), *tert*-butanol with a hashtag (#). See also Figure S44.

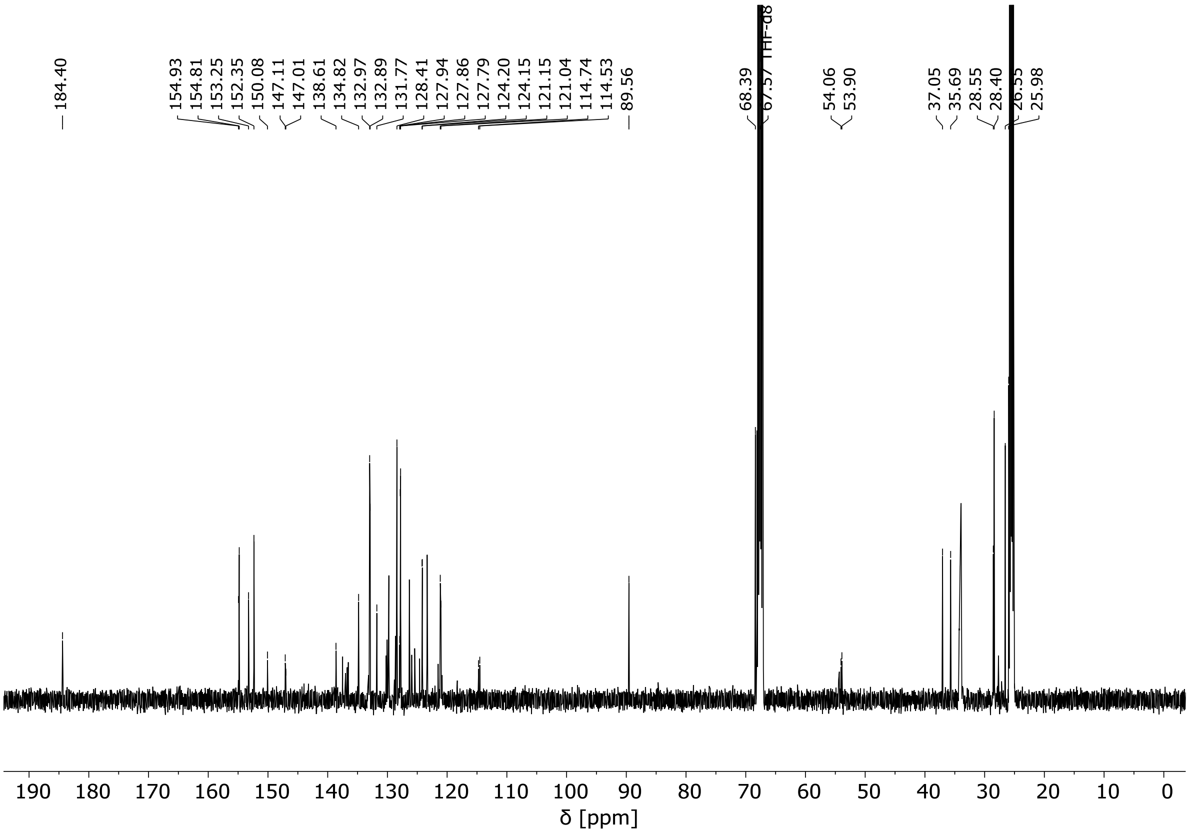

Figure S38**:** ^{13}C NMR spectrum of [**9**] in THF-d_8.

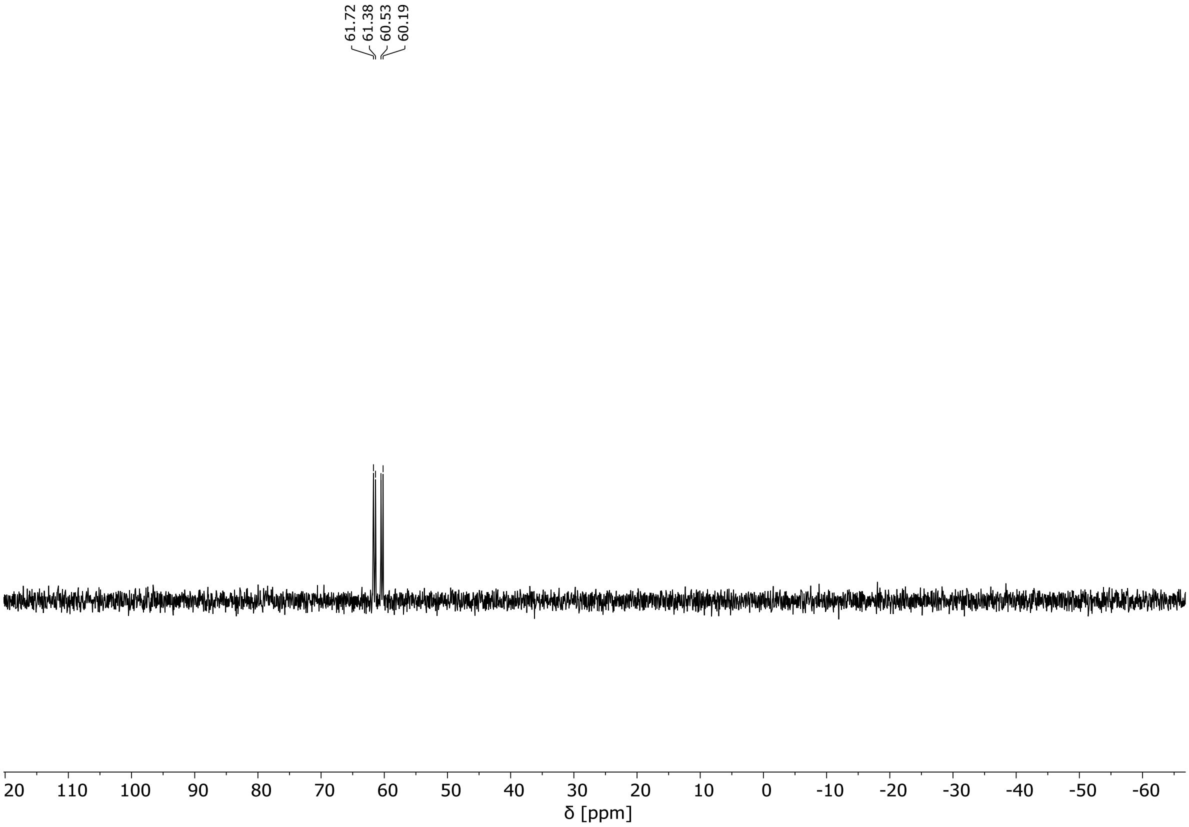

Figure S39**:** ^{31}P NMR spectrum of [**9**] in THF-d_8.

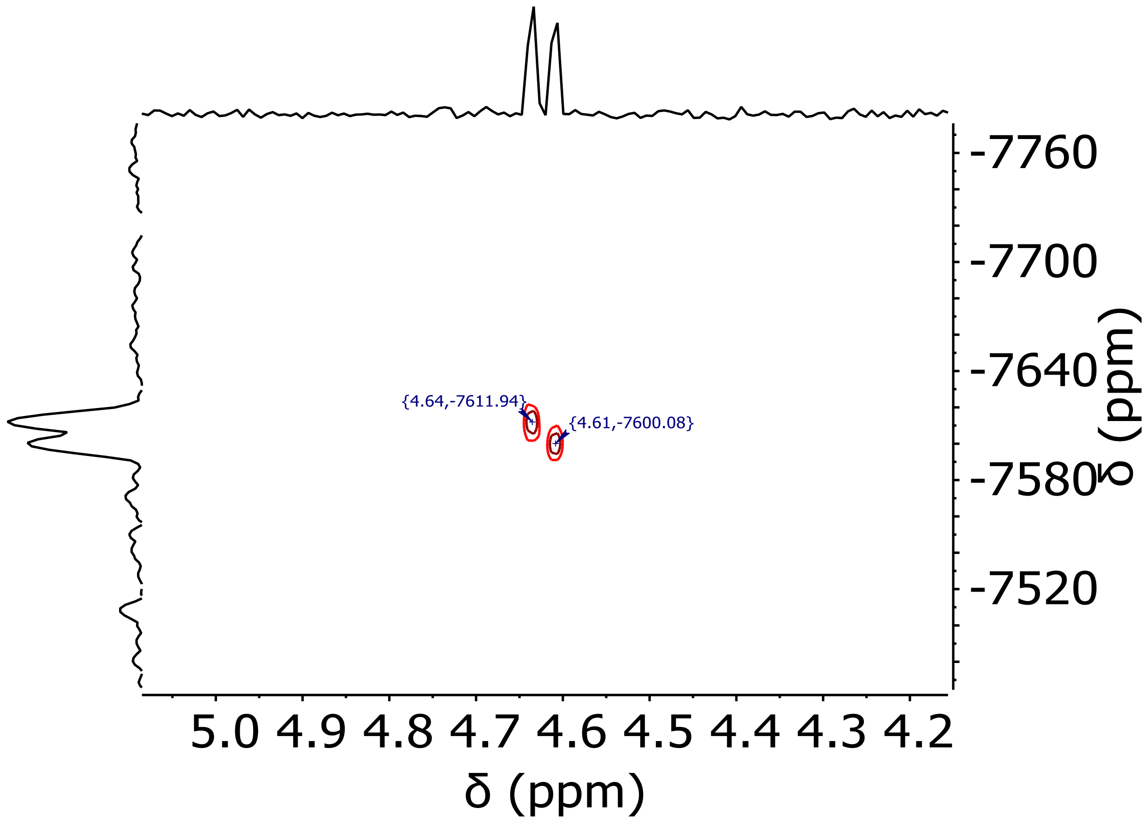

Figure S40**:** ^{103}Rh-^{1}H HMBC NMR spectrum of [**9**] in THF-d_8.

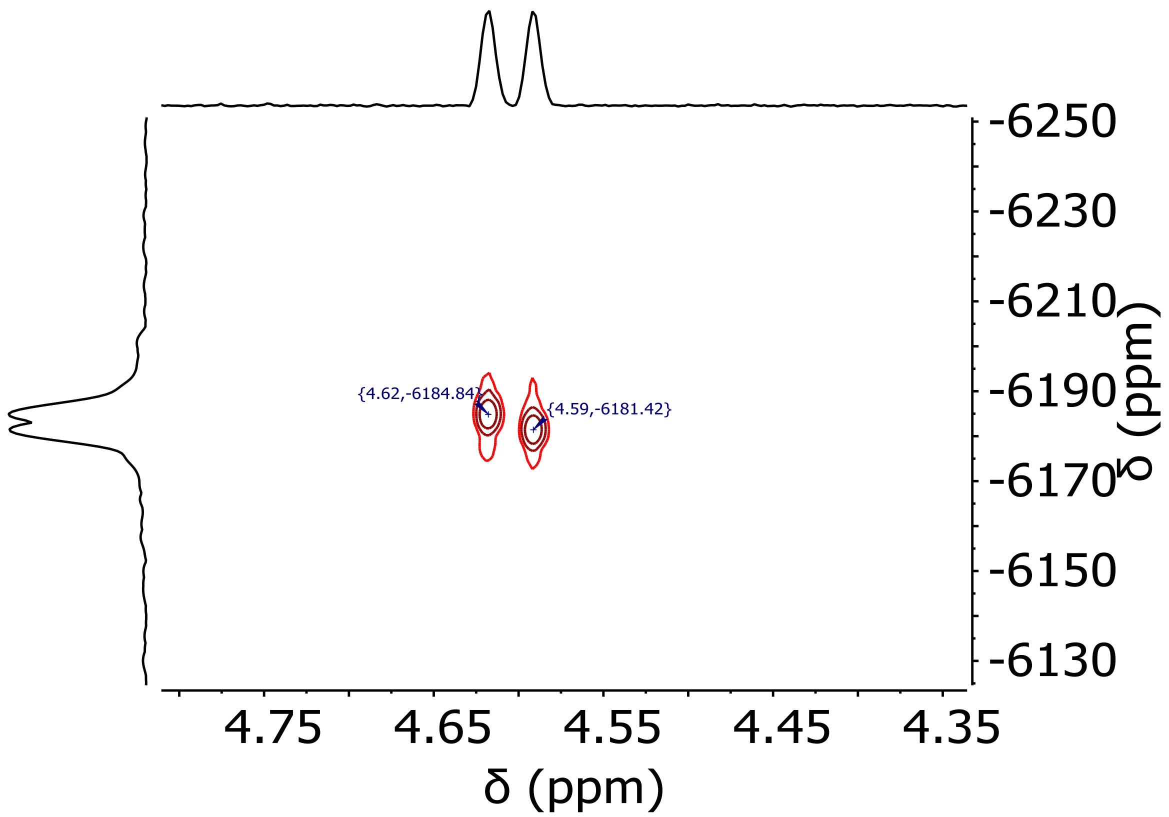

Figure S41: ^{103}Rh-^{1}H HMBC NMR spectrum of [**9**] in THF-d_8.

Further NMR Data

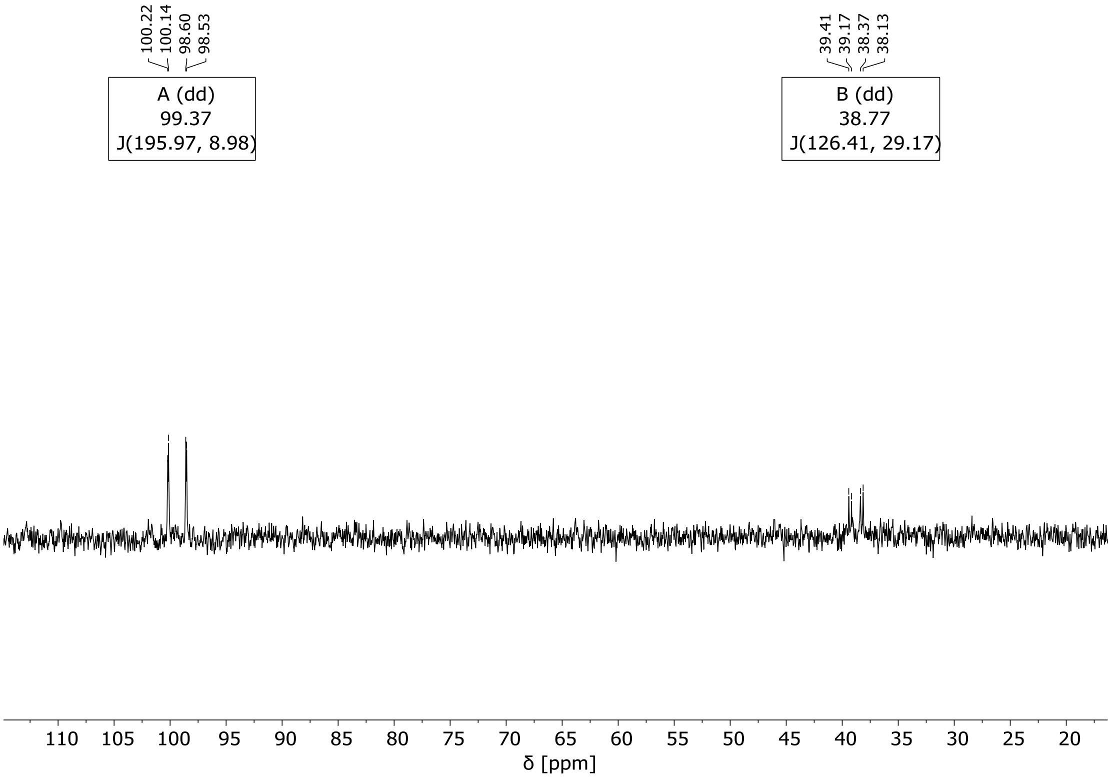

Figure S42**:** ^{31}P NMR spectra of the reoxidation of [**7**] with 2 eq. of $FcPF_6$ in THF.

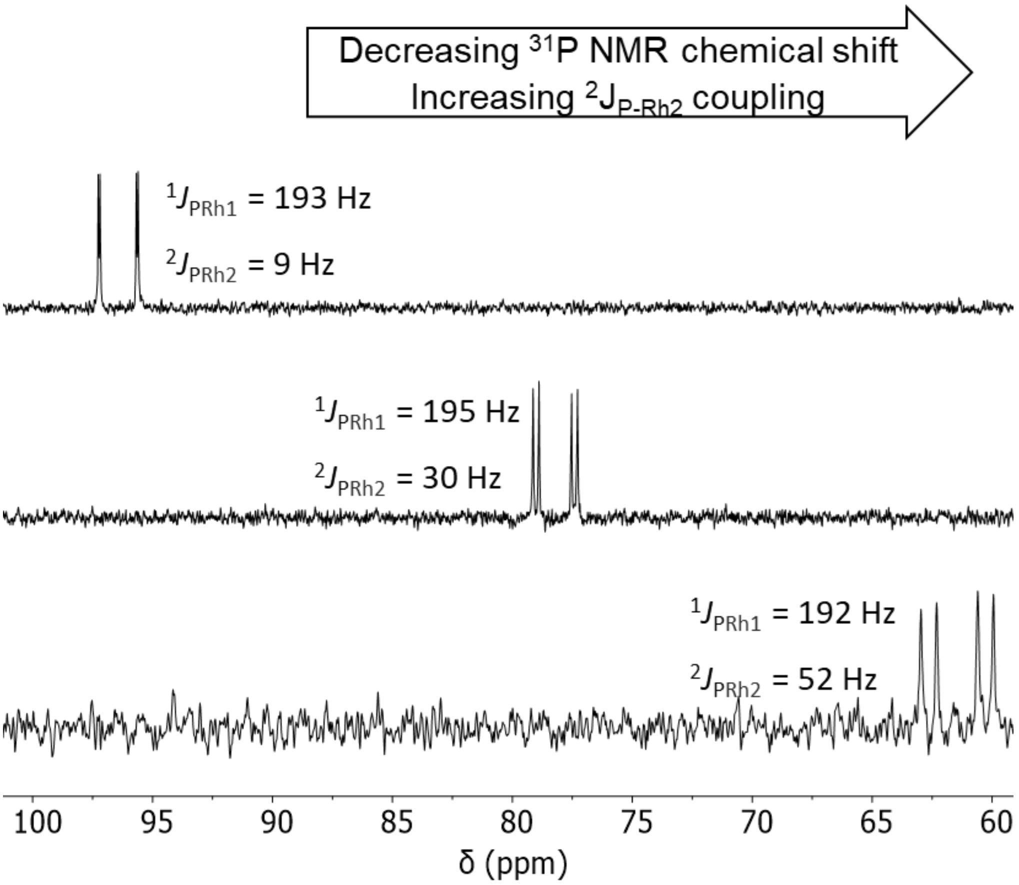

Figure S43: Comparison of ^{31}P NMR Data of [**4**]$^{2+}$, [**8**]$^{+}$ and [**9**] (top to bottom).

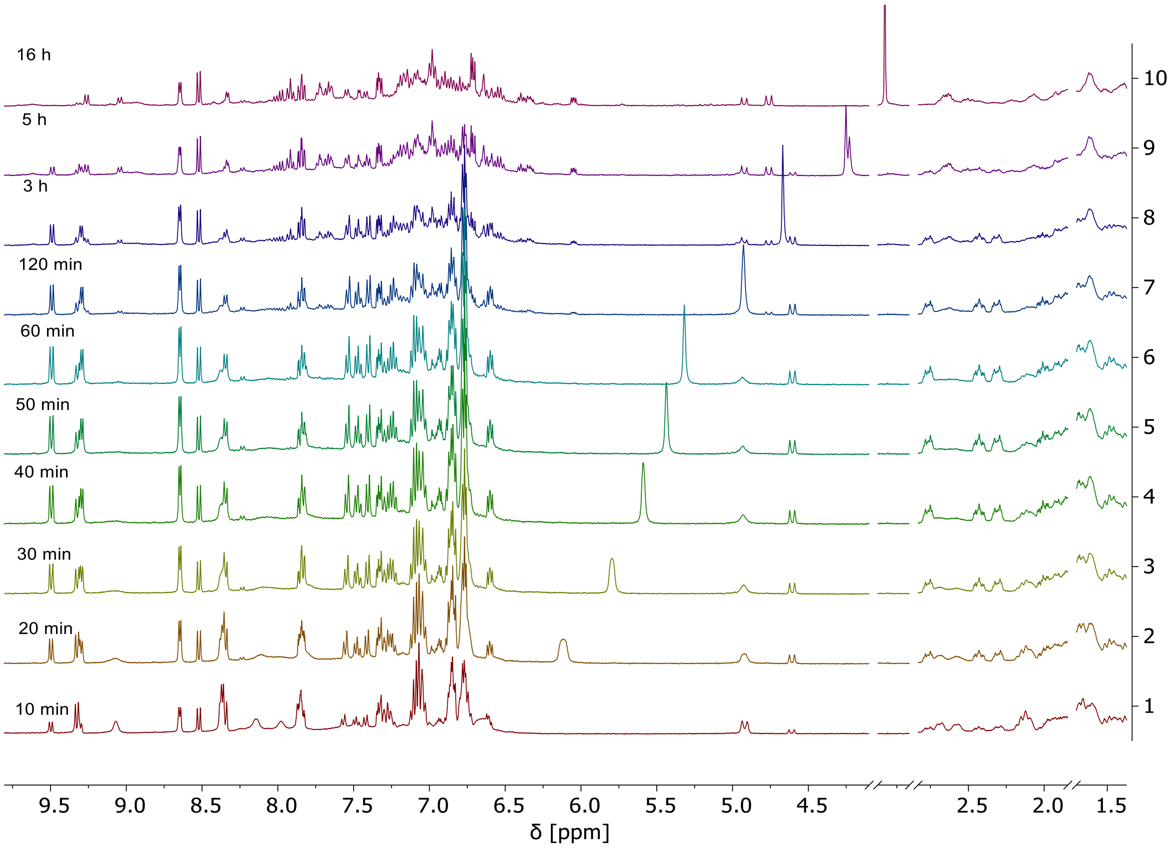

Figure S44**:** ^{1}H NMR spectra of the synthesis of [**9**] at different time points in THF-d_8. Significant is the signal of the benzylic proton at around 4.6 ppm.

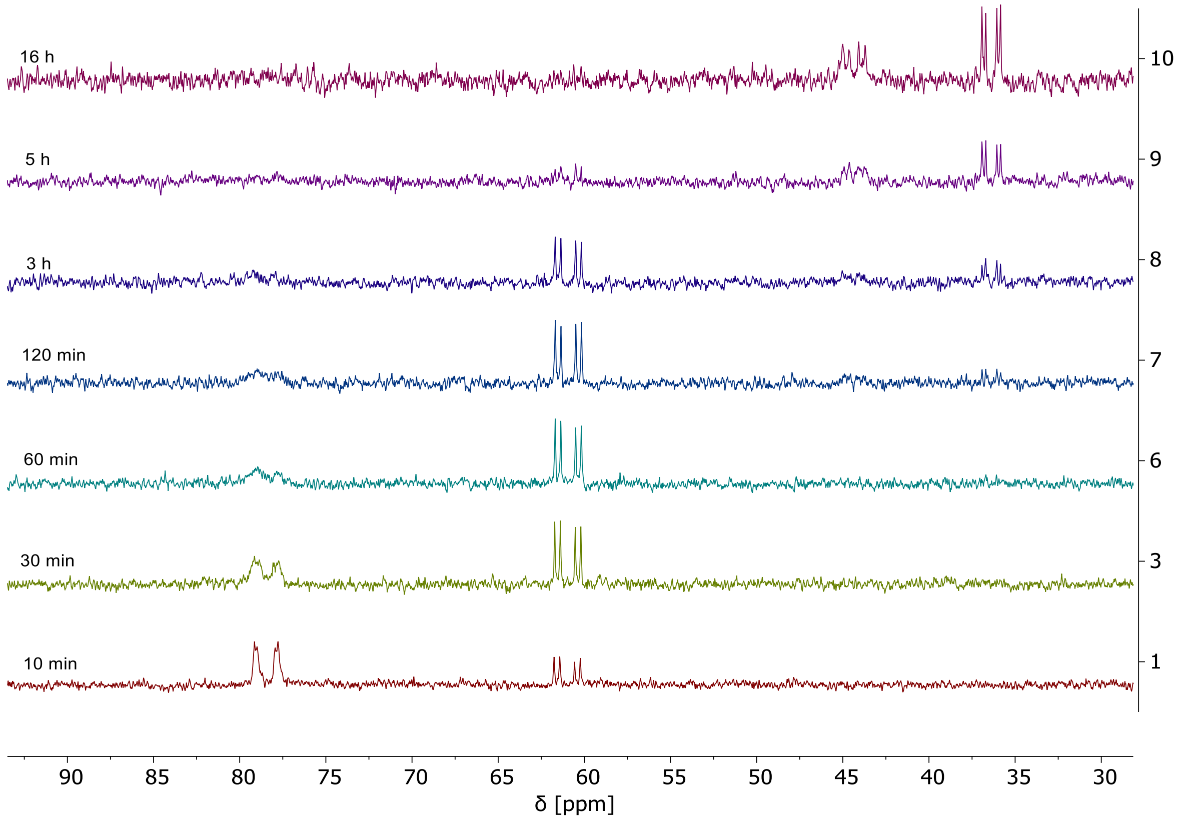

Figure S45**:** ^{31}P NMR spectra of the synthesis of [**9**] at different time points in THF-d_8. Signal of [**9**] appears and disappears at around 60 ppm.

UV/Vis Spectra

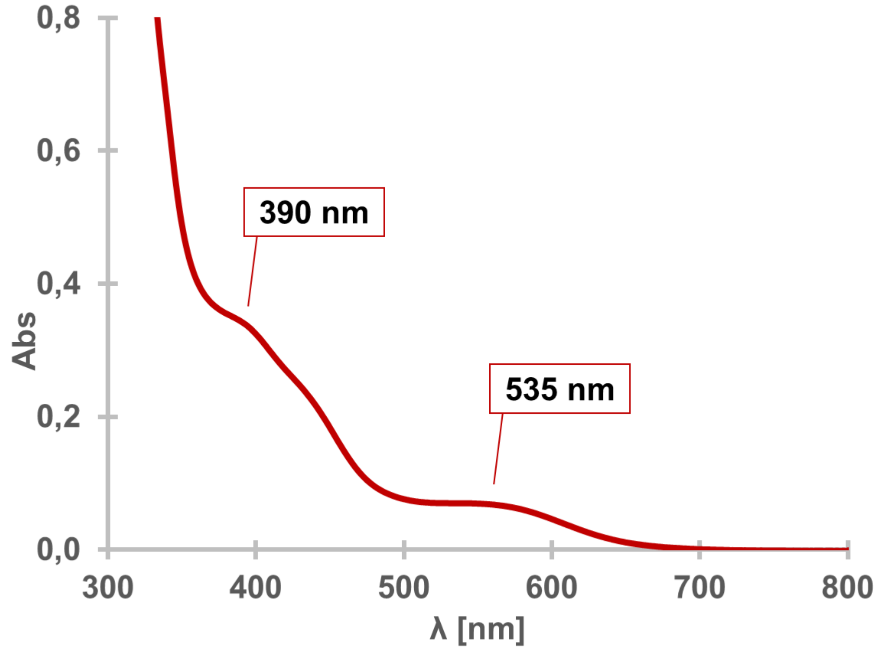

Figure S46**:** UV/VIS-spectrum of a solution of [**4**]$(OTf)_2$ in THF. Layer thickness: 2 mm.

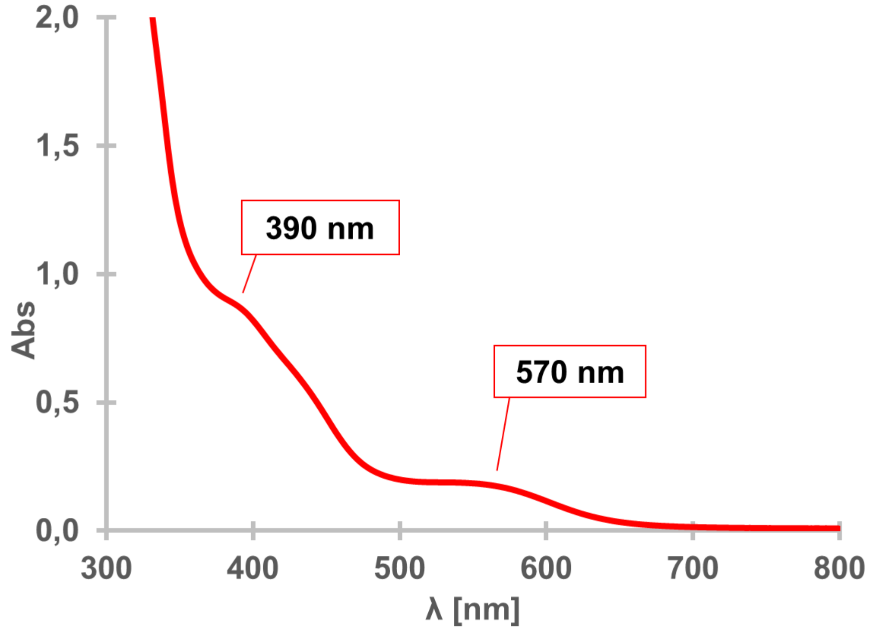

Figure S47**:** UV/VIS-spectrum of a solution of complex [**4**](OTf) in MeCN. Layer thickness: 2 mm.

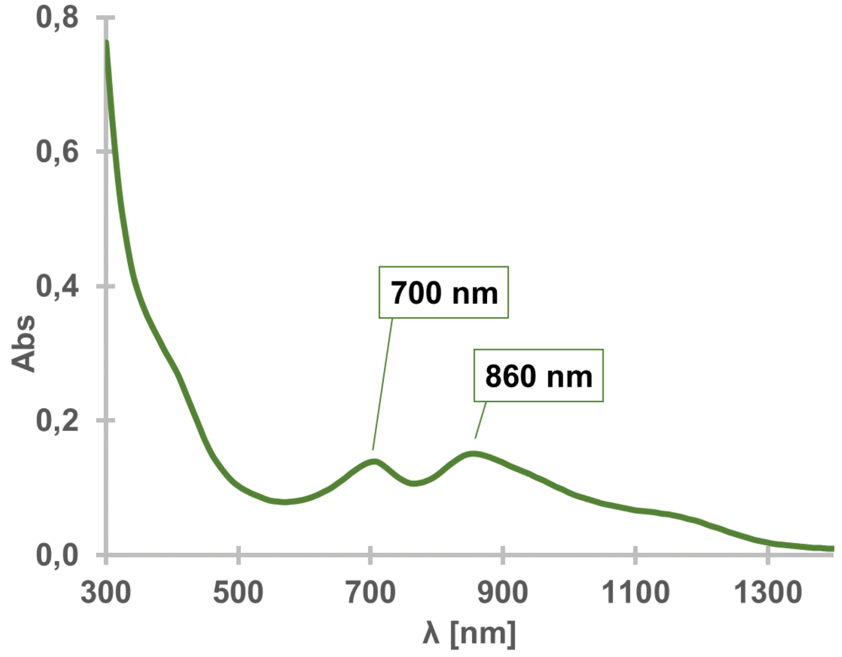

Figure S48**:** UV/VIS-spectrum of a solution of **7** in THF. Layer thickness: 2 mm.

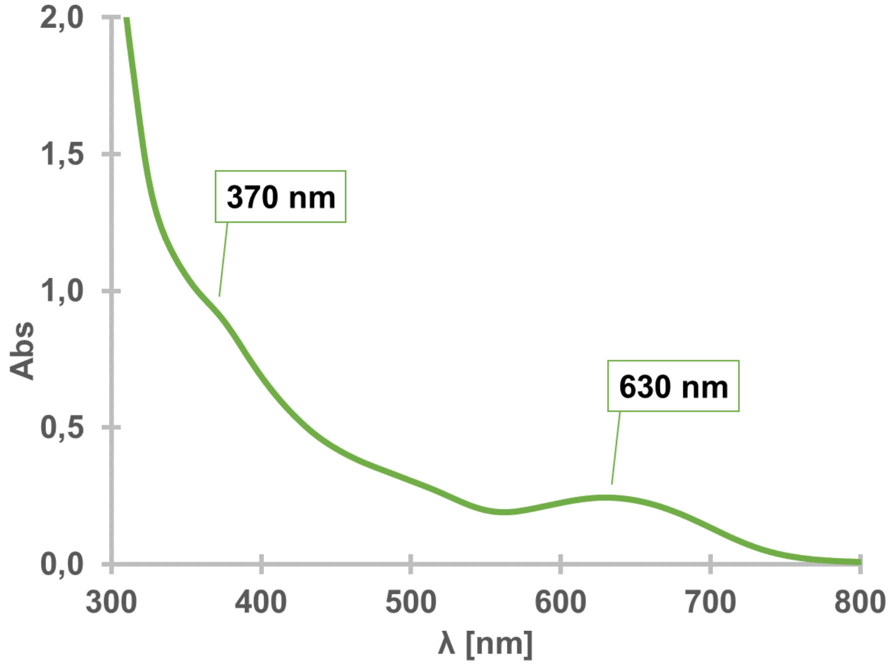

Figure S49**:** UV/VIS-spectrum of a solution of complex **8** in THF. Layer thickness: 2 mm.

EPR spectroscopy

The EPR spectra of the supported materials were recorded on a Magnettech MS-5000 (Freiberg Instruments) in a quartz EPR tube equipped with a J Young valve, loaded under inert atmosphere. EPR spectra of [**4**]$^{•+}$ and [**6**]$^{•}$ were recorded in a 1:1 mixture of toluene MeCN at 113 K (frozen solution) or 298 K. The frozen-solution spectrum of [**4**]$^{•+}$ is shown in Figure S50 and spectrum of [**6**]$^{•}$ in Figure S51 together with the simulated spectrum.

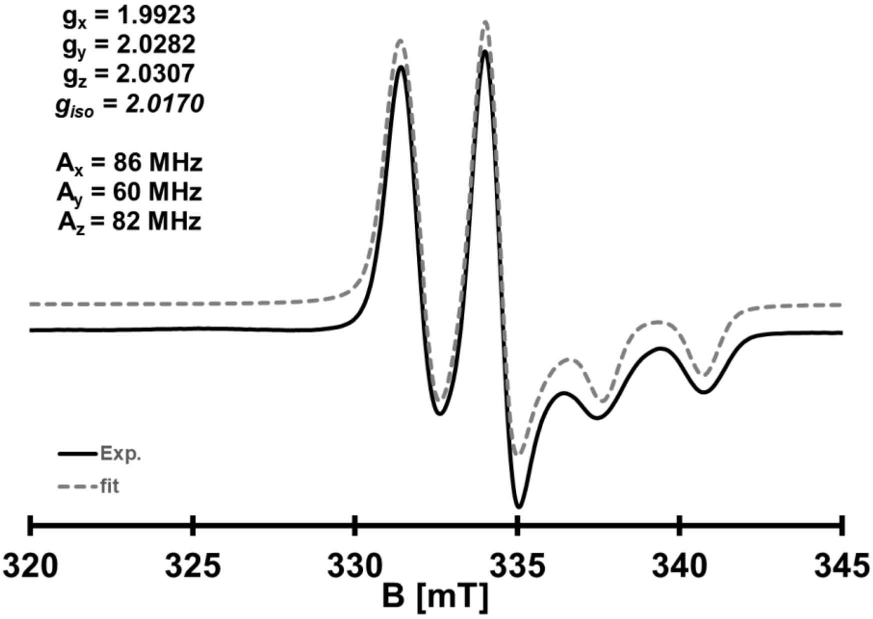

Figure S50**:** EPR spectrum of [**4**]$^{•+}$ in a toluene acetonitrile 1:1 mixture at 113 K with overlay of simulated spectrum. Fitted parameters are listed in the inset.

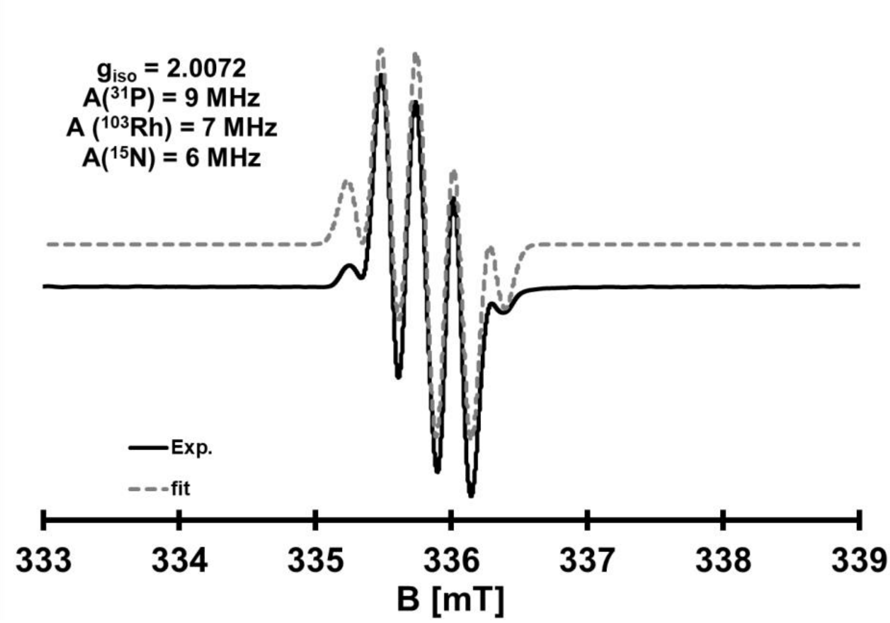

Figure S51: EPR spectrum of [**6**]$^{•}$ in a toluene acetonitrile 1:1 mixture at 298 K with overlay of simulated spectrum. Fitted parameters are listed in the inset.

Electrochemical Investigations

Cyclic voltammograms were recorded on an EG&G Princeton Applied Research Potentiostat / Galvanostat Model 263A controlled by the software Model 250 Research Electrochemistry Software (M270) 4.41 by PerkinElmer™ instruments Princeton Applied Research. All measurements were recorded with a Pt disc working electrode, Pt on TiO_x counter electrode and Ag/Ag^+ reference electrode. Measurements were performed with 100 mM [$n$$Bu_4N$][$PF_6$] and 1 mM analyte in 5 mL THF (unless otherwise stated). All potentials are given vs. Fc/Fc^+ as internal reference. No iR compensation was performed for scan rates below 400 mV/s.

The free ligand **1** shows a quasi-reversible reduction at -2.56 V followed by two irreversible reductions (-2.98 and -3.12 V). For comparison, a clearly irreversible reduction peak at -1.48 V is observed for neutral complex [**3**] (no changes in redox features for all combinations of solvent MeCN or THF and Pt or GC WE).

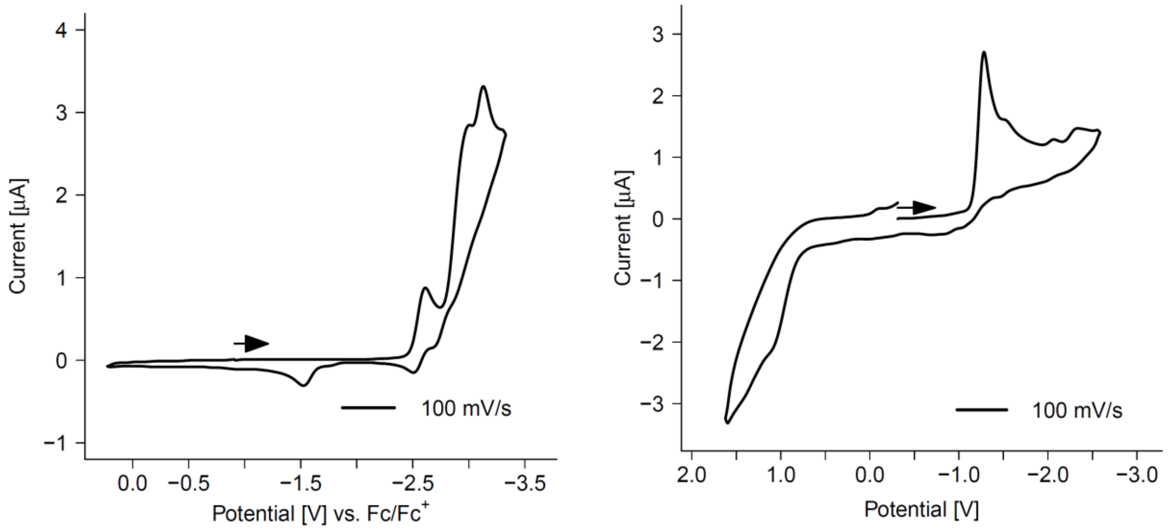

Figure S52**:** Cyclic voltammogram of **1** (left) and complex [**3**] (right) under standard conditions.

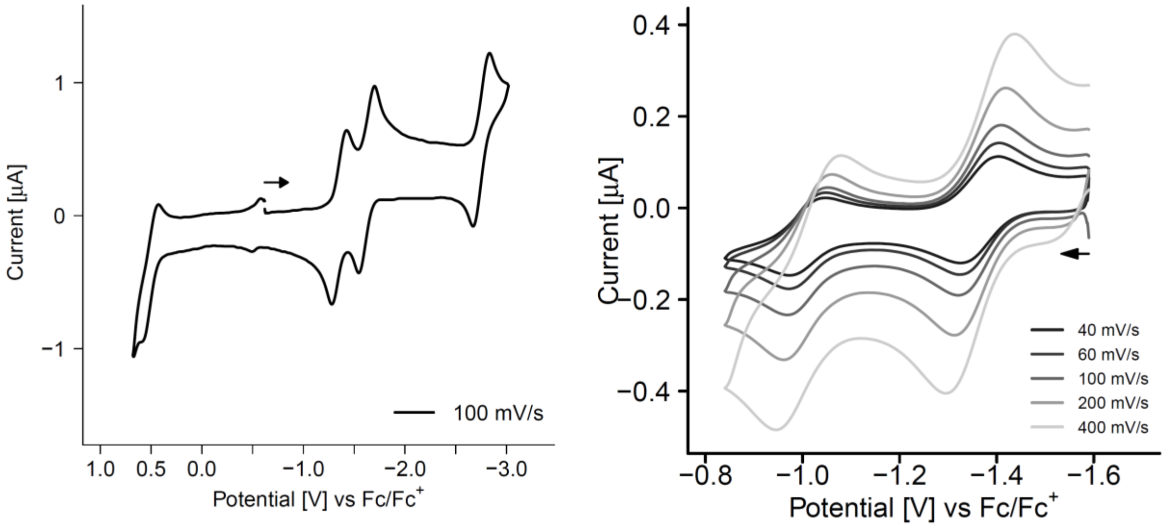

Figure S53**:** Cyclic voltammogram of complex [**4**](OTf)$_2$ in THF, GC WE, full window (left) and CV of [**7**] scan-rate dependent measurements (right).

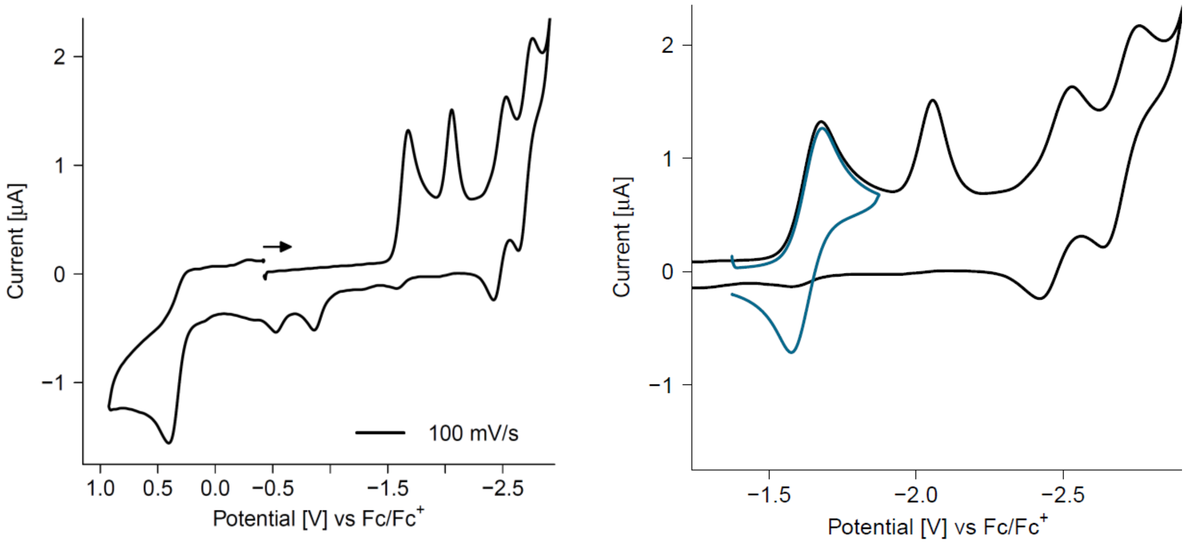

Figure S54**:** Cyclic voltammogram of complex [**6**](OTf) in DME (left) and zoom-in at the reductive window of [**6**](OTf) with overlay of isolated reduction event (right).

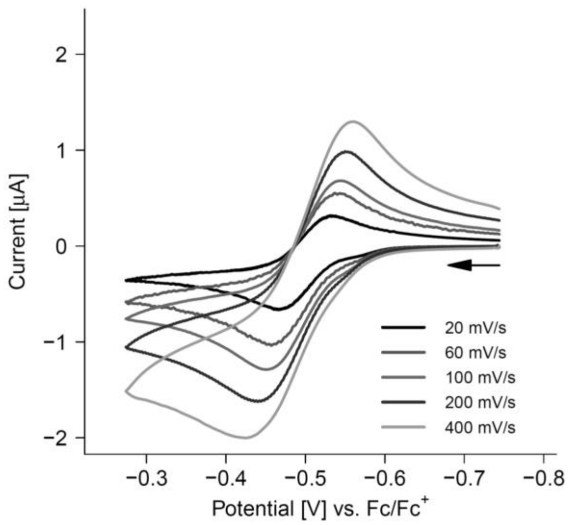

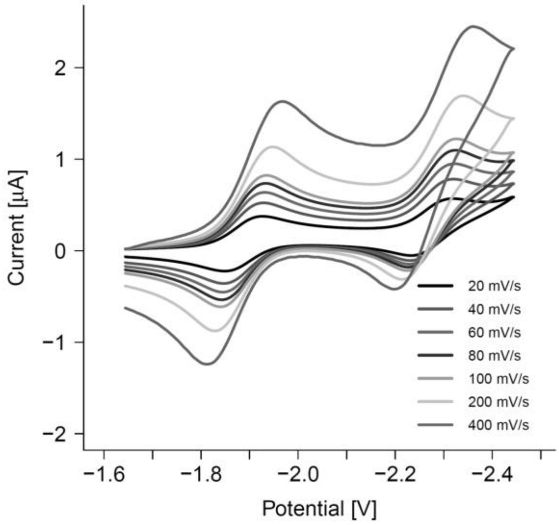

Figure S55: Cyclic voltammogram of complex [**8**]$^{+}$ in THF, scan-rate dependent measurements. Left: oxidative window, right: reductive window.

Calculations

General considerations

All calculations were carried out on the ETH Euler cluster with the ORCA program package.[55,56] Unless stated otherwise, calculations were carried out on isolated molecules. Density fitting techniques, also called resolution-of-identity approximation (RI[57]), were used for GGA and meta-GGA calculations and the RIJCOSX[58] approximation was used for hybrid-GGA calculations. Dispersion corrections with the Becke-Johnson damping (D3BJ)[59,60] were used for all DFT calculations. Orbital pictures were rendered with the software Chemcraft. All geometries were obtained using the BP97-3c functional and the def2-TZVP basis set. Approximate transition state structures were obtained using the nudged elastic band method. These approximate structures were used in a subsequent saddle-point geometry optimization.

Description of the Rh-Rh bond

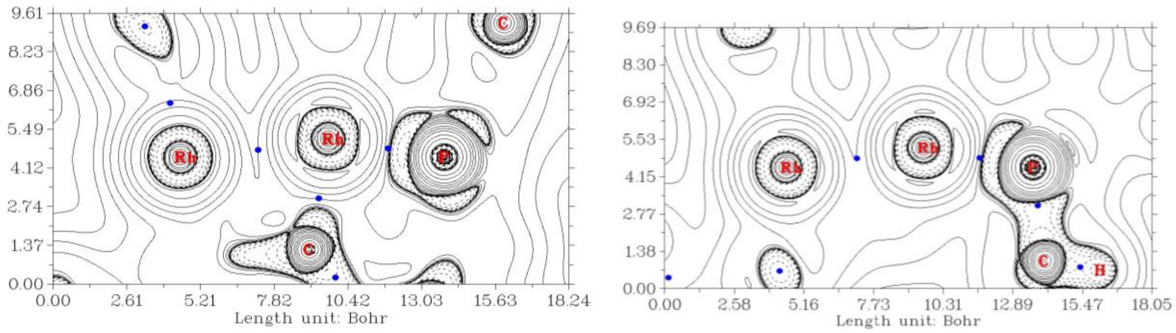

Figure S56**.** Contour map of the Laplacian of the electron density of dicationic complex [**4**](OTf)$_2$ (left) and of neutral complex [**7**] (right).

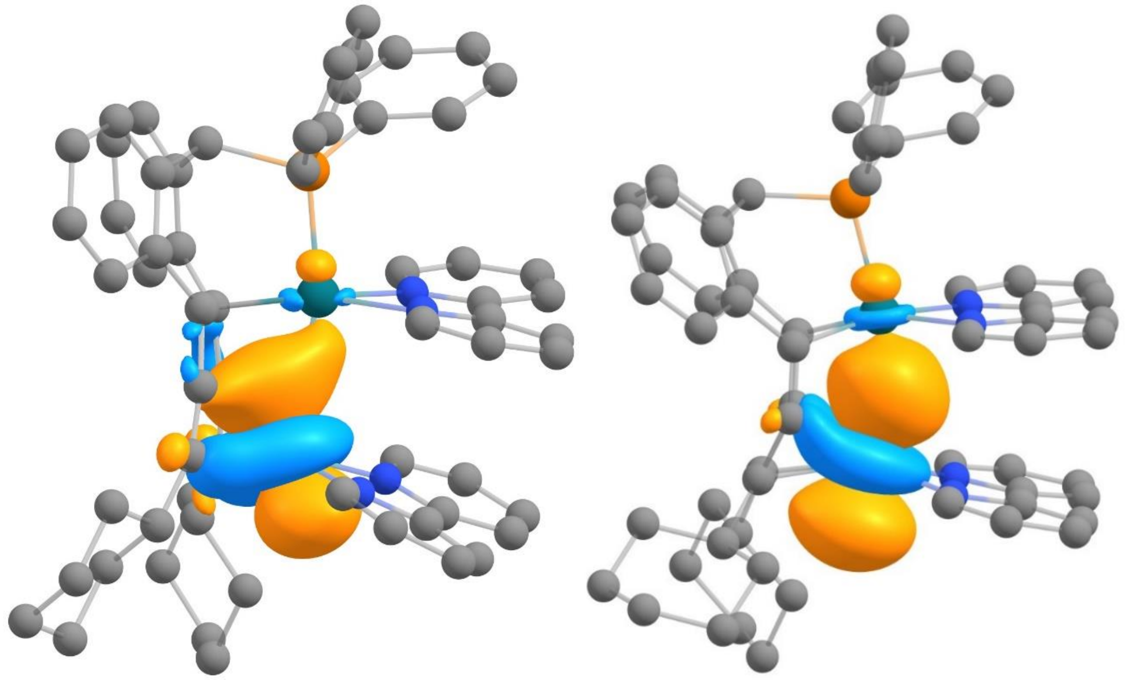

Figure S57**.** Relevant IBOs for the description of the Rh-Rh dative bond of dicationic complex [**4**](OTf)$_2$ (left) and of neutral complex **7** (right).

Molecular orbital plots

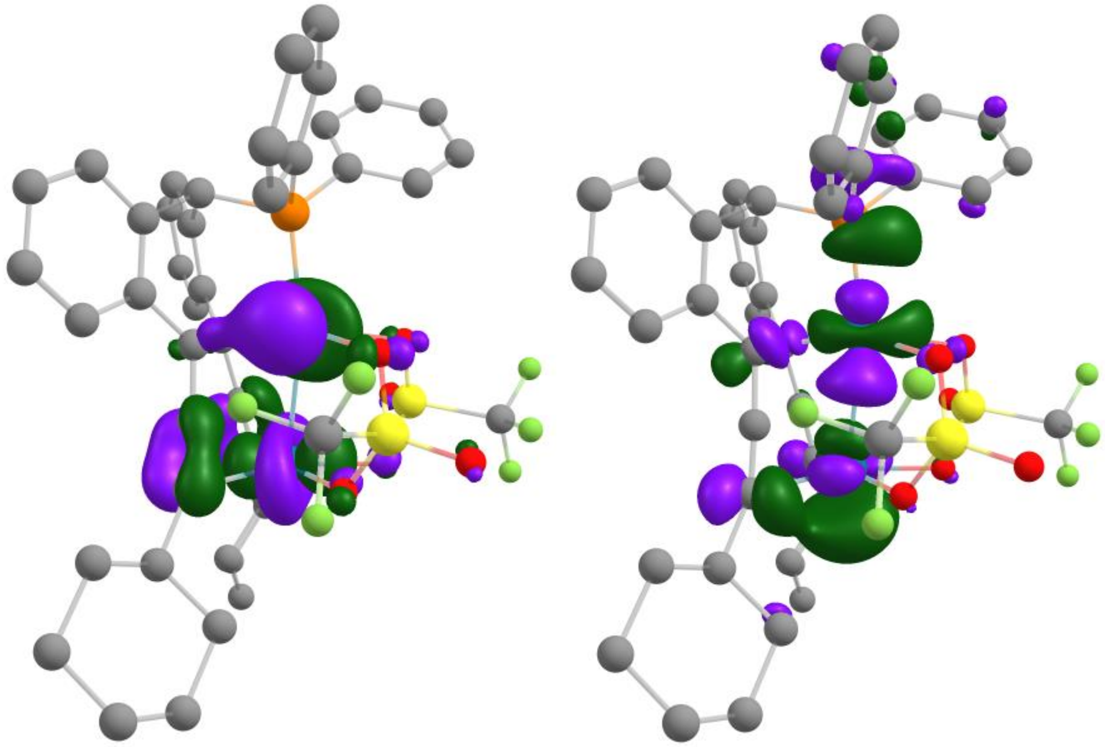

Figure S58**.** HOMO (left) and LUMO (right) of complex [**3**], surface isovalue = 0.04.

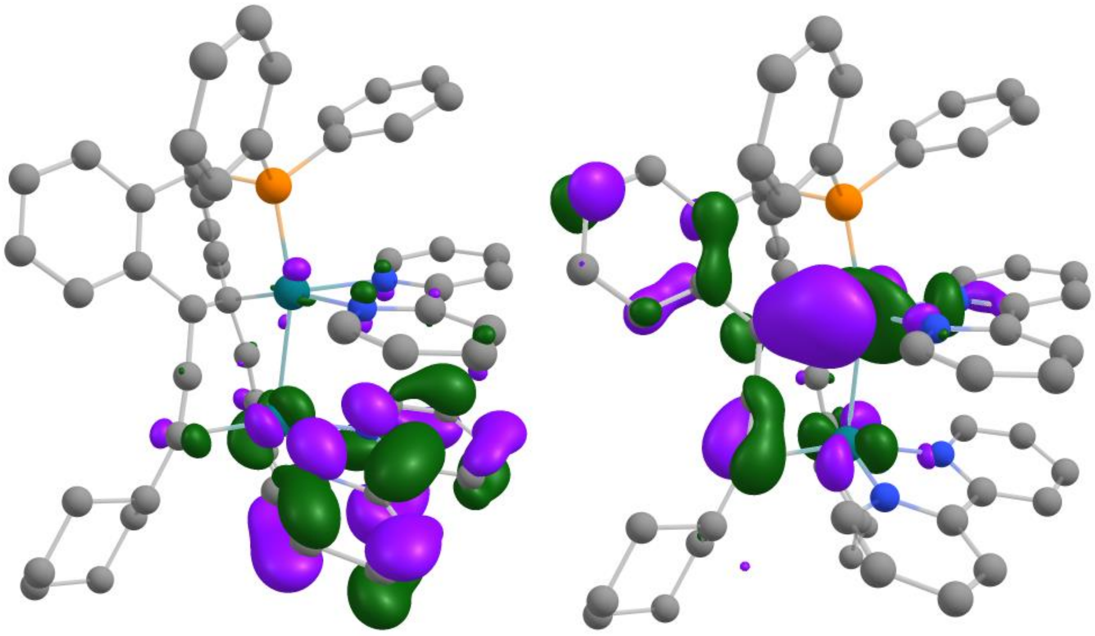

Figure S59**.** HOMO (left) and LUMO (right) of complex [**4**](OTf)$_2$, surface isovalue = 0.04.

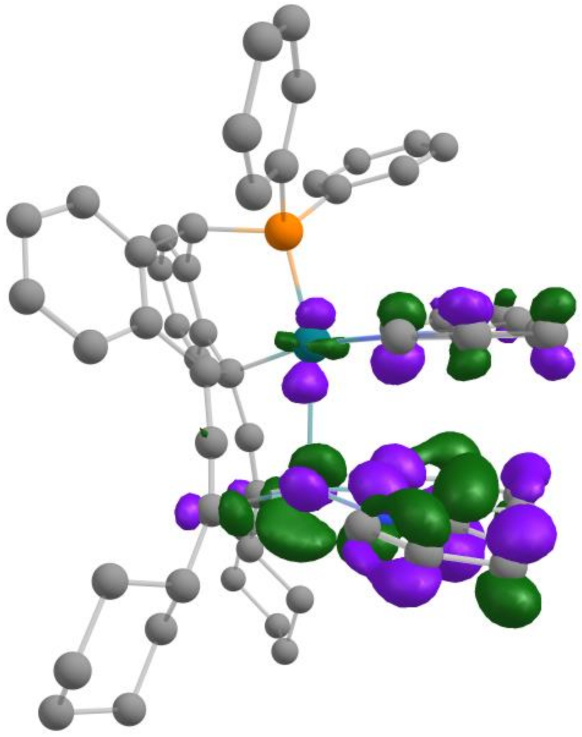

Figure S60**.** SOMO of complex [**4**]$^{•+}$, surface isovalue = 0.04. Spin density distribution: Rh^1 6%, Rh^2 9%, bipy2 52%.

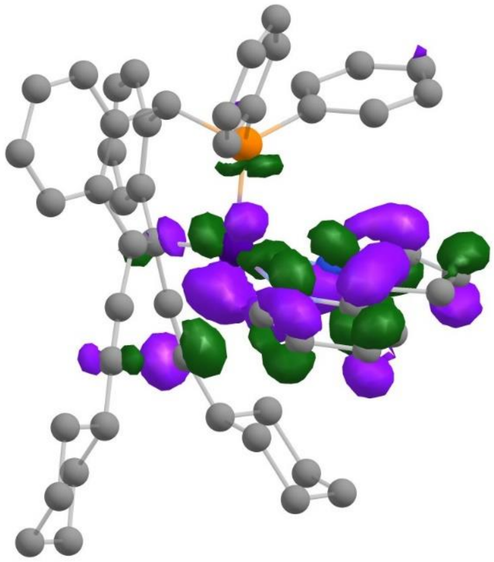

Figure S61**.** SOMO of complex [**6**]$^{\bullet}$, surface isovalue = 0.04. Spin density distribution: Rh^1 22%.

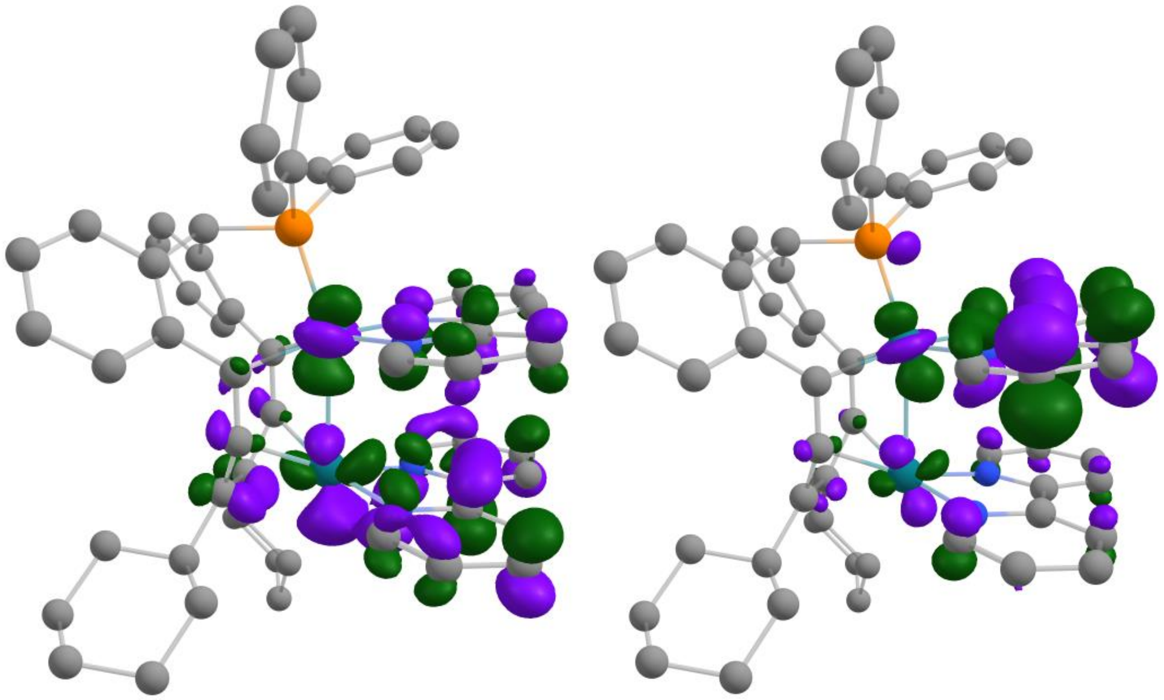

Figure S62**.** HOMO (left) and LUMO (right) of complex [**7**], surface isovalue = 0.04.

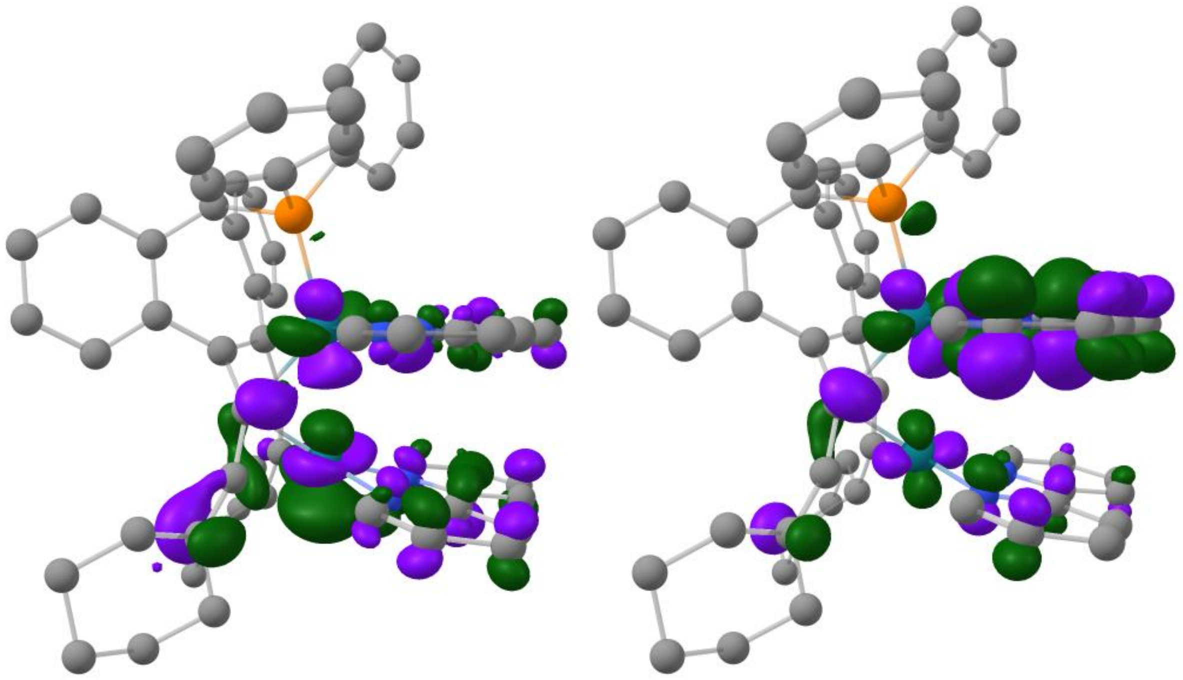

Figure S63**.** HOMO (left) and LUMO (right) of complex [**8**]$^{+}$, surface isovalue = 0.04.

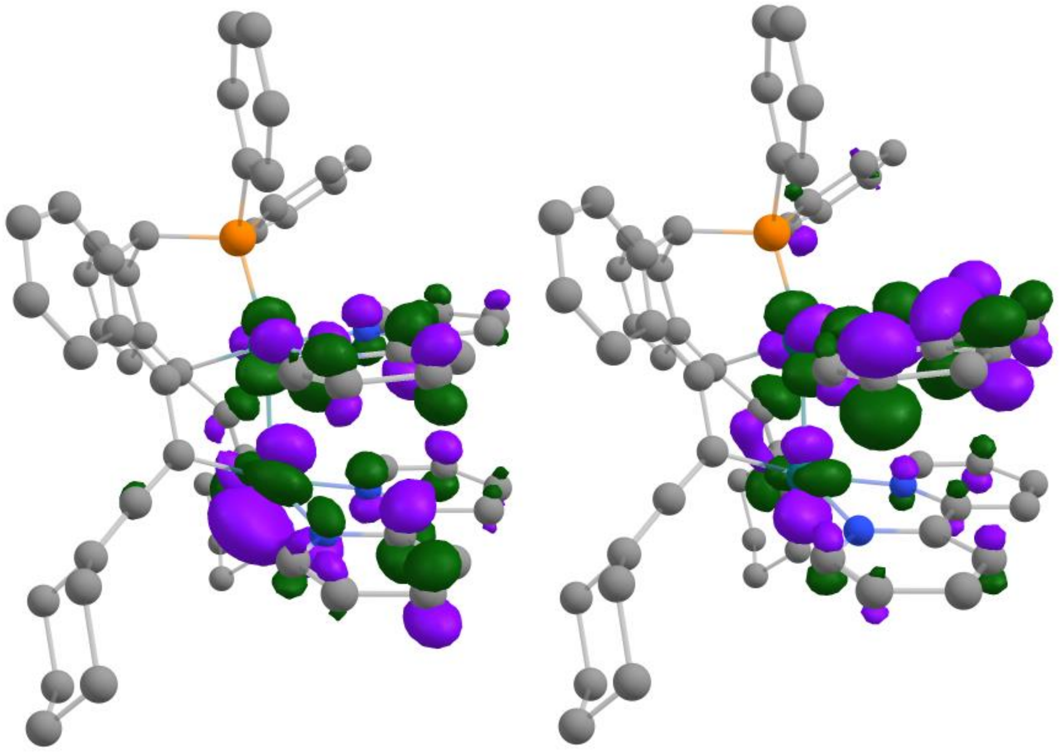

Figure S64**.** HOMO (left) and LUMO (right) of complex **[9]**, surface isovalue = 0.04.

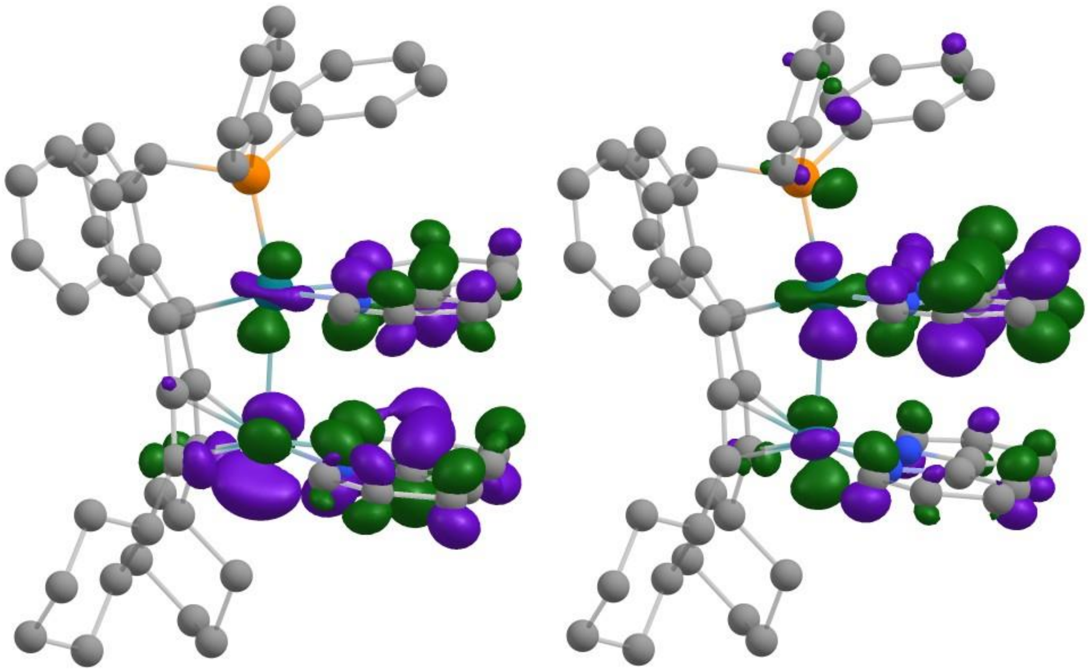

Figure S65**.** HOMO (left) and LUMO (right) of complex [**4**], surface isovalue = 0.04. Around one electron is delocalized on bipy2.

Cartesian coordinates of optimized structures

[3]

Rh	1.44789212562840	2.50027710261643	4.76655939080567
Rh	3.36751379943817	0.89563316755371	5.56960719314868
S	1.57808929283078	-0.45426067639409	3.22973430985967
S	0.73222332841463	0.57717972803101	7.32485471233298
P	0.16618405593815	4.13064275309465	4.10191513336803
F	1.26710984736855	-0.16075259798991	0.58960272761145
F	-0.62258257861756	-1.54870096742882	6.42698449702735
F	3.07617187292136	-1.24057319318068	1.15060235491161
F	2.97853809540995	0.92288021228525	1.39404503271713
F	0.10627877137547	-1.74994380225309	8.47172238408265
F	-1.60136321631881	-0.46338275095379	8.04372522899595
O	2.87386074645647	-0.48451273288909	4.03424478800144
O	1.97019981782943	-0.14696759304506	6.78684079320231
O	0.74701282647898	0.77770637545864	3.43786405193515
O	0.86259957527519	-1.72819093967483	3.19185224621206
O	0.02317332498713	1.38626201003603	6.28379091424384
O	0.91591524046770	1.19844915760388	8.64292591875634
C	3.24772395564604	4.54500313183372	3.26721911626273
C	3.18246035614417	3.51046183591272	4.30913079040887
C	2.30169546863883	5.58219137737081	3.18168675571345
C	4.08783599254207	2.39964317917741	4.25228722945739
C	-1.70534634113071	5.56623047018542	5.68801417460361
H	-1.04660519380032	6.41829632191343	5.65399928253156
C	2.76898924835382	3.74729300811122	5.73163416892450
C	1.18654112902751	5.70946123126484	4.17863348206968
H	0.53606138111807	6.52000784269808	3.85588233892431
C	1.41580639257292	7.24325126788492	6.13054752838364
H	0.86073544202384	7.97358287040669	5.55603740493775
C	2.42737817914304	5.06095502139918	6.28863012074723
C	-1.50095521324851	4.80870273972772	1.94231594182829
H	-2.12536061640919	5.31135593500680	2.66700985430939
C	-3.41066590466225	3.37774054553713	5.82534259906869

H	-4.06647869086132	2.52096851170743	5.88613923425648
C	1.68431932696896	6.00344097441974	5.56023492436906
C	4.96150041656962	1.50691476055223	4.31839313283061
C	-0.36762539726322	4.11326060286921	2.35976124919677
C	-2.22498432646317	3.28264823325288	5.11869836110682
H	-1.95492126189515	2.35347585376884	4.63811213393960
C	-0.42641913700137	-0.90769187318013	7.57758050937793
C	6.20657232752665	0.80323669153201	4.02252081541637
H	6.89307662969605	1.01596026443496	4.85064643312340
C	-1.36603013396434	4.38146546502512	5.04140058635134
C	4.44013825176448	1.30091375105174	8.53962944923935
H	3.76355096472741	0.48401103921798	8.80685584677235
C	0.42056302227150	3.44781509260687	1.42429748769377
H	1.28666911627190	2.89868701864726	1.75589617039486
C	3.89793568091927	1.91083303080403	7.33461680402782
C	-3.74763450048753	4.56279125584449	6.46752101337205
H	-4.67067339876555	4.63217685909411	7.02575096511887
C	2.27070069918433	-0.21979345255610	1.47247455769684
C	2.83867257995296	5.38728490334747	7.58309174011979
H	3.40095815528282	4.66251515029537	8.15148712913241
C	-1.83447385315923	4.84288670572667	0.59700580281758
H	-2.71785635878579	5.37763219506125	0.27649923331905
C	3.35716848586651	6.38482016940796	1.15641858050193
H	3.38468062229558	7.09223237741644	0.34001243493665
C	3.35759113393617	2.76140284403478	6.59232635360642
C	1.83771807296631	7.55758431673522	7.41185027807877
H	1.60957049537092	8.52551473121088	7.83490559084835
C	2.54759516676627	6.61745451936736	8.14417951456273
H	2.87750500370668	6.84175731734727	9.14856786144481
C	4.23545223527173	4.46066780549933	2.28296614970523
H	4.96017455820058	3.66286889977032	2.33795948530110
C	2.37263317518522	6.48288085862577	2.12627118023602
H	1.62801361175599	7.26410991386432	2.05660309821122
C	6.38469323136450	0.07197564601363	9.56490429064309

H	7.38778405298492	-0.31279079764951	9.37853342869205
H	5.76828638494754	-0.78920871175189	9.83505352137060
C	-2.89449623791931	5.65410800786825	6.39785571861008
H	-3.14953756469000	6.57603989167561	6.90167074996505
C	6.03689412438536	-0.71826737154766	3.95252619860408
H	5.61178101965619	-1.08516522977981	4.88618195118456
H	5.31238442098639	-0.95597603594658	3.17602946517009
C	4.45542568277611	2.29777932444020	9.70374627327601
H	5.07047019531804	3.15628087599851	9.42278698450407
H	3.44389668952560	2.66145136587457	9.87430562500271
C	7.36306242082039	-1.40069827231092	3.66014216912470
H	8.04966687639913	-1.23721741313664	4.49586136571496
H	7.21137155027317	-2.47778061056187	3.59217090497783
C	-1.04311893033128	4.18290882011118	-0.33424480666603
H	-1.30874957473825	4.20709797924654	-1.38197148729451
C	6.84139418317239	1.34163397394914	2.73352110138741
H	6.13943929066005	1.18179906818639	1.91325446031844
H	6.99277573323115	2.41748358890328	2.82062943255820
C	4.29494749083237	5.36655403669222	1.23904737379636
H	5.06827049535844	5.27269220425644	0.48977106061206
C	5.01845501789898	1.65356441394619	10.96146435726844
H	5.05317640605263	2.38889855384539	11.76591536849252
H	4.33809774021700	0.86564110489733	11.29329834937093
C	0.08366778520193	3.48664413291368	0.08110278592700
H	0.69518525566542	2.96151609188345	-0.63869398859308
C	7.99262112921236	-0.87032442971473	2.38033725779726
H	7.35418447845205	-1.13153156358303	1.53249981663485
H	8.95577763183276	-1.34951299369966	2.20273995333432
C	5.83056423210075	0.70228230057298	8.29711569348886
H	5.77145937364932	-0.03198052981935	7.49345184692678
H	6.49827049801726	1.49488250320765	7.95125190903545
C	8.15867929690517	0.64125058990228	2.43487840432597
H	8.88585989987450	0.89776208186882	3.21012847313134
H	8.56634067335296	1.01578230352702	1.49533806977652

C	6.39999171247542	1.06196227615669	10.72123160735528
H	6.76663886894765	0.57820504928007	11.62697186518542
H	7.10291458947593	1.86860895618029	10.49437587649973

[4]$^{2+}$

Rh	1.40584799539869	2.98195861066527	3.82042591131766
Rh	0.98922686860984	5.11882885991823	2.08551375059108
P	1.99018808002983	1.04424611534591	4.68446680738690
N	-0.36834390820663	6.02543296002359	3.34006738940905
N	2.16377181099997	6.58982900057988	2.95565026503799
N	-0.18751236191601	3.34066901462900	5.22824669530675
C	2.22504398483363	2.41795122714542	1.95942200365843
N	2.26969916395757	4.26920068796197	5.35497622848756
C	0.72731947677742	-0.31558507383689	2.54961273874634
C	-0.59859419842220	4.19105935111142	0.97063078643497
C	-1.07021210643335	0.64727903312832	1.27247163555059
H	-1.53001032513561	1.50470541365091	0.80517091349090
C	0.13751833903092	0.81180550262902	1.95697444608917
C	3.16398159306125	0.12015719944069	2.38789841517152
C	2.02023693033811	-0.21765990398967	3.29769030118057
H	2.22968719777461	-1.17101363349038	3.77875668338462
C	-0.00309531874962	3.26070770857605	1.55264488702460
C	0.74344202701906	2.14878207714404	2.05907349199640
C	2.45247788812380	3.68605883448506	1.34783964855912
C	3.66799903440617	0.91432556440766	5.37333431694358
C	-0.13615330431721	-0.45492643742599	5.77560079646942
H	-0.24917347890883	-0.92099063413829	4.81109299084075
C	0.08392578775329	7.13668420413291	3.97833982041868
C	3.23394976517630	1.36609699307067	1.74206039657627
C	4.18079868965995	-0.80835252904071	2.20477018879200
H	4.12223836450783	-1.75870662417343	2.71681132114033
C	1.48088083402499	7.47583569913927	3.72846997912118
C	2.56555116847478	4.72369091440924	0.66634744803199
C	4.70911284700471	1.60187841048627	4.74901488717466

H	4.49495582875082	2.23512538374898	3.90274421912965
C	-1.52264272247136	4.85679166838274	0.04754664327738
H	-1.10007550446696	4.68269284415990	-0.94903960230383
C	0.12015136503189	-1.55973165066565	2.40915665786296
H	0.59386627861527	-2.42743400108442	2.84822384889848
C	-1.67464041197468	-0.59191255872803	1.15637063058064
H	-2.60343367563539	-0.68948248480832	0.61348790443871
C	-1.60624704431843	5.58116177351450	3.59012294804971
H	-1.89510713332854	4.68610623155405	3.06592277511329
C	3.95512588054414	0.04539576702289	6.42633345947997
H	3.16465419909489	-0.50974172376312	6.90767006072759
C	0.87964242171718	0.47227982897280	5.99986884543896
C	-1.62449128117720	6.37499941108837	0.22217910149347
H	-2.05145564654601	6.59635501955335	1.20058930376679
H	-0.62878102224458	6.82033811098041	0.20999664624550
C	-1.07358419178950	-1.70673297819487	1.72216238515094
H	-1.52552064202806	-2.68276183163582	1.62516207176992
C	4.33542766637255	1.62503141512396	0.92154794892824
H	4.40120400422285	2.57383420486207	0.41152447917268
C	-1.01989398826992	-0.77972266296594	6.79543517163801
H	-1.79261017565101	-1.51359314422418	6.61689573215670
C	5.34132483388672	0.69127844949854	0.74537606325662
H	6.17828269662739	0.91796122978257	0.10097630397813
C	-0.74186798438201	7.83048670792090	4.85462237618723
H	-0.38440543485126	8.71659023183481	5.35323304231724
C	5.26737680655496	-0.53392044553393	1.39102107376710
H	6.04759923973562	-1.26953931641552	1.26375542498168
C	6.01437980201023	1.43292537831879	5.17846461332263
H	6.81662936304785	1.95790137430722	4.67972322979750
C	3.47905365381078	6.76119425285580	2.76993398622208
H	3.97142408179965	6.00214806903325	2.18419282196942
C	0.98233004757787	1.08562433425028	7.25223923558359
H	1.76818935745180	1.80390998904524	7.43898902584989
C	-2.46327059077937	6.22699399316538	4.45738386800749

H	-3.45485143326620	5.83448976604518	4.61875657194360
C	5.26373257008694	-0.11438789925386	6.85701148055086
H	5.47839918757195	-0.78817842482102	7.67374913864917
C	3.00946645531496	5.64403320198199	-0.37647877937183
H	3.03634306654764	6.65770401690004	0.03619570922919
C	-0.90749589600566	-0.17520813318055	8.03822139664910
H	-1.59162256274963	-0.43668217299515	8.83240908468847
C	-1.35259287029932	2.68987730115048	5.16418981726022
H	-1.48198986149108	2.03701037875466	4.31391896449279
C	2.12384924203319	8.58549104880646	4.26474217161869
H	1.57410193439414	9.30137909915921	4.85368926646629
C	1.40894283903035	4.74565015000216	6.28480536540696
C	-2.50603129069303	6.98075606754369	-0.85863666372897
H	-2.57715921615425	8.05787971312004	-0.71318209138963
H	-2.03525135179809	6.83260317417153	-1.83347999551161
C	-2.02731741952370	7.37900333819119	5.09410698594566
H	-2.67565169290101	7.91742393854440	5.76874791368282
C	0.09568631117536	0.76006305513471	8.26337131147531
H	0.19222251983952	1.22764688769569	9.23289314727651
C	-2.91751733907362	4.21638001041097	0.06858638702889
H	-2.83623803148554	3.14133376311320	-0.08356499397283
H	-3.35903397199723	4.36431864117062	1.05700122198397
C	1.80638075212020	5.72083592342835	7.19388079164197
H	1.10964017870702	6.10763047140172	7.92004018876150
C	0.06804585909446	4.15180414819870	6.27210378107404
C	6.29375570541873	0.57989879980496	6.23817695975597
H	7.31221018019562	0.44706700422085	6.57301177331370
C	-0.87827671782503	4.34258532139728	7.27404249566941
H	-0.66637864681158	4.97722989644190	8.11950828254201
C	3.98349896944725	5.68693022204412	6.22294319915425
H	5.00948687353800	6.01695469477582	6.17256011249833
C	2.02083576428128	5.66544776095910	-1.55384962362394
H	1.93056291546445	4.65171432822026	-1.94727690759924
H	1.03578940368607	5.95432880771004	-1.19214534327572

C	3.52351929043458	4.72978959074732	5.33841653997184
H	4.16859913159683	4.30630020902235	4.58755302654415
C	-3.89073268033197	6.34986732761012	-0.85947120145244
H	-4.40305152716460	6.60438012766959	0.07239321389801
H	-4.49436610333261	6.76739731680139	-1.66346636894097
C	3.47316055520841	8.78002295074615	4.03265558503970
H	3.97423235180560	9.64838326131460	4.43300588607607
C	4.16699673454104	7.84150345673936	3.28398782240594
H	5.22309690616777	7.94531453459892	3.08886572679426
C	-3.81209270770671	4.83691444715732	-0.99550413804816
H	-3.42154341310822	4.58016529627835	-1.98276527371463
H	-4.80594409920912	4.39523674753606	-0.93669146892228
C	3.10463393568197	6.19728790878867	7.16525126262827
H	3.42605665511590	6.94666304850506	7.87316392634546
C	4.42235332267346	5.28850640403540	-0.86126300554819
H	5.11857962612862	5.29433110648224	-0.02140451541389
H	4.40739566630973	4.26906128306150	-1.25063582592003
C	-2.32931196532752	2.82803457427589	6.13150948634835
H	-3.25068822652159	2.27262203371542	6.05000054773745
C	2.49312721532344	6.61421011216877	-2.64477500159140
H	1.79448318814389	6.58345148046536	-3.47993986133636
H	2.47179491537160	7.63886877043015	-2.26416001698497
C	-2.08768877990696	3.67506331479630	7.20304944449413
H	-2.82328835561726	3.79887139951682	7.98379011549897
C	3.89978835702623	6.27700520326503	-3.11509176486893
H	3.89263085117719	5.30182968065833	-3.60756302067783
H	4.22876186992660	6.99588396292383	-3.86355061624230
C	4.87993327178269	6.24540870448559	-1.95223300597824
H	4.98202945627805	7.24968246662728	-1.53272942268876
H	5.87149920984889	5.95452756238435	-2.29643463632966

[4]$^{\bullet+}$

Rh	1.42719953171011	3.01186662969045	3.82074066613992
Rh	0.94097062375508	5.12727890876414	2.12271533878615

P	1.99306705939547	1.07847344501140	4.68722082245080
N	-0.45964823277008	5.96176039717241	3.35313703475882
N	2.06167329974394	6.57393579364880	3.04658946245631
N	-0.08819906456043	3.45990185572079	5.25411683360378
C	2.19683375034808	2.45115505396780	1.94866093450137
N	2.39450911026057	4.29975319604517	5.25714750981868
C	0.70476350390947	-0.28901447186380	2.58045187727068
C	-0.58058496697794	4.20498994698219	0.90300694464556
C	-1.13087458579934	0.67615043768834	1.36658187051071
H	-1.60561904530922	1.53693850724225	0.92167753038140
C	0.10556782053395	0.84061660901868	1.99989426665303
C	3.14143518452625	0.14942506634601	2.37962082997972
C	2.01055746504376	-0.19416855628140	3.30456140026904
H	2.22803096568026	-1.15048736935632	3.77621460145804
C	-0.02881950285641	3.27917516887144	1.53311104846697
C	0.71360438958605	2.17842369963327	2.06965677484425
C	2.41680044895179	3.72363827089018	1.33923270046403
C	3.67067197939713	0.88275080576971	5.37825380649263
C	-0.15719405070542	-0.38229832062959	5.80707992381639
H	-0.28041715285846	-0.86264552684067	4.85076749297692
C	-0.00516285640331	6.99296736581489	4.14011679787100
C	3.20304144786009	1.39705989961231	1.73313347737053
C	4.16149769362100	-0.77499540741145	2.19213032017402
H	4.10829362136910	-1.72317816562348	2.70959458709934
C	1.37163465655594	7.34679735283544	3.95377783486028
C	2.51295849652363	4.77931885577579	0.68123685951898
C	4.73169286947791	1.53093055312934	4.74700882490255
H	4.53457966491228	2.16201465807380	3.89574364481612
C	-1.45362493955555	4.83913083518781	-0.08952956098676
H	-0.91554285418282	4.77170201259036	-1.04204743715667
C	0.08531749417499	-1.53019493810512	2.46950910452897
H	0.56884001558401	-2.39562437020142	2.90337119603358
C	-1.74913189460134	-0.55888336061759	1.28339622049446
H	-2.70213878881761	-0.65017227065005	0.78183024136590

C	-1.72575851837736	5.53677175216145	3.50136047694678
H	-2.00543497497596	4.69879874368281	2.88437513767774
C	3.93469993294459	0.02312998007293	6.44380840684433
H	3.12693980174929	-0.49970413445353	6.93304136692145
C	0.88127908762860	0.52269099771569	6.01348829753949
C	-1.72309292607448	6.32516726204010	0.15863942277641
H	-2.25242320330588	6.44170609376425	1.10430692292183
H	-0.77831533900933	6.85606924095872	0.27191252132520
C	-1.13458673691166	-1.67596455275109	1.82988668585987
H	-1.59710329451410	-2.64973836620886	1.75875508204161
C	4.30289206724811	1.65620999481409	0.90947798252196
H	4.36178154534803	2.60746876748600	0.40365193434097
C	-1.05523724645141	-0.65935425786473	6.82904205462603
H	-1.85015732837774	-1.37199367138573	6.65969318655186
C	5.31350294141350	0.72744336257964	0.73202152979371
H	6.14924621852230	0.96033781327544	0.08730813822999
C	-0.87399728943084	7.61153296668664	5.04625960112510
H	-0.51787512432431	8.42621373744972	5.65677914541171
C	5.24644845662969	-0.49852241772170	1.37661115687603
H	6.03042839267254	-1.23095460721924	1.25048632162714
C	6.03208874248605	1.33148170553780	5.17897073730314
H	6.84716581374510	1.82992314483021	4.67338766816938
C	3.37354000696226	6.80170977116713	2.86709101527364
H	3.87268452493592	6.12839148621978	2.18937369162531
C	0.99113321704118	1.16209559633211	7.25232255658983
H	1.78586725918829	1.87485105056852	7.42156023020065
C	-2.60768194903299	6.10821341515073	4.38330481881313
H	-3.61246429333668	5.72351088484159	4.45920871675848
C	5.23769888001865	-0.16638809263509	6.88002070097056
H	5.43213757259715	-0.83207382800090	7.70904400898612
C	2.98636539001995	5.69336331380736	-0.35633127391642
H	3.12602285740637	6.68499673163278	0.08685429982372
C	-0.93489472038476	-0.02922729810270	8.05784851415720
H	-1.63529254272320	-0.24729395275326	8.85141199096720

C	-1.28895295274645	2.87374261538186	5.23671996296253
H	-1.47359512387391	2.20980331577266	4.40536557385790
C	2.02532129554336	8.38624078838590	4.62159322333233
H	1.47710625417422	9.00169101092182	5.31710000436174
C	1.60215768535440	4.79718450539974	6.24561092992736
C	-2.55664829541389	6.92005635323174	-0.96441147472510
H	-2.74843130553289	7.97382570056125	-0.76332642246702
H	-1.98692401076995	6.88306429431705	-1.89701974867998
C	-2.17196871307965	7.17885224350561	5.17208135300219
H	-2.84164533337132	7.65778769739395	5.87065181263648
C	0.09088219580239	0.88505242419522	8.26527225477124
H	0.18865479962967	1.38318795875069	9.21956800254190
C	-2.77051382392348	4.07153232751750	-0.25958589092252
H	-2.56112460005106	3.02500258885539	-0.47702250439844
H	-3.31688474639449	4.09616160004791	0.68632169733913
C	2.11445266765692	5.69691561224815	7.17936799854663
H	1.47783385043918	6.09722467082867	7.95251431285412
C	0.23858082802845	4.28857788293036	6.27363440117086
C	6.28785262110125	0.48820636781776	6.25164137911129
H	7.30221762695914	0.33421196613086	6.59118499143745
C	-0.68179691710969	4.55549976217083	7.28695312404509
H	-0.42213415289172	5.21240188727750	8.10181413790846
C	4.23616492786043	5.56562545479299	6.09766296822342
H	5.27411868583680	5.84273393974615	5.99936757707125
C	1.95346117036569	5.85172452099402	-1.47976129128645
H	1.74463567304363	4.86596445479450	-1.89997883436458
H	1.02119602430821	6.22000606247722	-1.05736732799020
C	3.67363968168852	4.68544271242331	5.19964058382298
H	4.24904505317256	4.27212363185284	4.38859199430788
C	-3.86594172995941	6.16478700861415	-1.14148327999533
H	-4.48030645904764	6.29838855662524	-0.24680739505914
H	-4.43605311036703	6.58171341769645	-1.97123046639664
C	3.35787879950990	8.63534257837725	4.39269340069419
H	3.85709344522660	9.44702769418194	4.90020885863826

C	4.05442129318848	7.80943082238651	3.50593951592718
H	5.10581230260313	7.95217295018085	3.30943549319065
C	-3.62096301012252	4.68004834532373	-1.36564353523184
H	-3.11394211406043	4.53833922952876	-2.32342568923126
H	-4.56657154495231	4.14271052261328	-1.43794921477881
C	3.43510953548396	6.08857654558801	7.10816150397080
H	3.83731835474503	6.78578220313168	7.82798295049413
C	4.33571098289502	5.23479207257257	-0.92315024481869
H	5.06603612219390	5.15288792495701	-0.11717765657781
H	4.21231555619078	4.23343451975066	-1.34043817513187
C	-2.23294072802711	3.08788139939095	6.21891333067846
H	-3.18502879515688	2.58250948064535	6.16966690978489
C	2.45756556267812	6.78978528420110	-2.56544708135085
H	1.71960201387512	6.85543747961293	-3.36499438884480
H	2.55358566209194	7.79772137732593	-2.15284930728986
C	-1.92111570846359	3.95042188282884	7.26390842796160
H	-2.63371715245633	4.14083711675309	8.05279752610373
C	3.80233489493313	6.34139785989315	-3.11779416329232
H	3.67945264841308	5.38552335976041	-3.63350848906783
H	4.16049556957914	7.05041423850981	-3.86365187966602
C	4.82938763109767	6.18173061702778	-2.00652244780796
H	5.04037190889785	7.15882963000542	-1.56367801411834
H	5.77409662435379	5.81697633021189	-2.40962610650215

[6]$^+$

Rh	1.04669601487990	2.60245615090158	3.03299154096217
P	1.71016228412105	1.02360927696141	4.37771541890029
N	-0.55272649806058	3.24353861045799	4.35371337318080
C	2.23674023368164	1.80512230321453	1.46547278014070
N	1.74496783301548	4.45395525134569	3.99083337651696
C	0.95178235568560	-0.95007224341018	2.46330478151342
C	-0.64207714217144	3.41321731521406	0.90095345097351
C	-0.66320034435707	-0.51553745999821	0.72674980203576

H	-1.10174390963380	0.16844713699273	0.01412139685503
C	0.38046841031716	-0.06459042280258	1.53741843781179
C	3.31266049384241	-0.22010619895215	2.56515892791652
C	2.07023896629905	-0.51117588221476	3.35360344719832
H	2.28693084974686	-1.29754154677139	4.07412920019025
C	-0.08179252817154	2.33469754507783	1.02367428764029
C	0.81615180579758	1.33029241092620	1.44962010070308
C	2.54343698345991	2.97330020902232	0.72101271066995
C	3.27919391221340	1.22350339873938	5.27930474312809
C	-0.48900013241091	-0.37411670123096	5.43480290127527
H	-0.48858729243059	-0.95324941387464	4.52559919432380
C	3.36895567080705	0.86521842285526	1.67376290440261
C	4.43004457819958	-1.02392382879821	2.75524361287843
H	4.37169006203354	-1.84294199861483	3.45904890791439
C	2.82306042374800	4.00633765663639	0.15323216632123
C	4.26017372479870	2.05498593387338	4.74530509214772
H	4.04655657017807	2.61485554041137	3.84987755064859
C	-1.31813823185685	4.67517887788502	0.72134808638848
H	-1.38979680303646	4.86771915183649	-0.35542274853872
C	0.48534415820184	-2.25907073435287	2.53294457873708
H	0.93408611128423	-2.94081809779971	3.24299341459160
C	-1.12453425603908	-1.81759110396429	0.81221718270763
H	-1.92713920878098	-2.14774780146285	0.16864087760820
C	3.56484428621428	0.46037215135027	6.41136191815004
H	2.81536470255527	-0.19587494749825	6.82909937413824
C	0.48791190282693	0.59627654469910	5.65286335272476
C	-0.49847840390823	5.81989961266535	1.34128880711320
H	-0.38533851333124	5.61456681179699	2.40662165800840
H	0.50325845616456	5.82311352711603	0.91787051329133
C	-0.54613637604553	-2.69666676574549	1.71743566725935
H	-0.89269175070463	-3.71763335808801	1.78643425729058
C	4.56945785448830	1.08364987185759	0.98966024846347
H	4.62726966720408	1.90635876567028	0.29451506717306
C	-1.47404816534148	-0.59677725356076	6.38638046122440

H	-2.21707975033246	-1.36264682341506	6.21468040605781
C	5.67399713059041	0.27265873980265	1.18081505612186
H	6.58336035865756	0.46975834987107	0.63110921571212
C	5.61057107954747	-0.78731054496587	2.07229143956352
H	6.46905776926312	-1.42225062370659	2.23646745884548
C	5.50946452133238	2.13040906174126	5.33906607592392
H	6.26870328650079	2.77120854322850	4.91376927715128
C	0.44638943449271	1.35680670739202	6.82375095935898
H	1.19615870461148	2.11449998949061	7.00092000726612
C	4.81450523219040	0.54333378170585	7.00580686038413
H	5.02997032958673	-0.04763108957957	7.88460582034628
C	3.14074788474335	5.20258108780193	-0.59511976476784
H	2.99186675384666	6.07173687174016	0.05885270442719
C	-1.50465381400611	0.15503679353497	7.55151701219714
H	-2.27168496406000	-0.02216764910485	8.29178802704416
C	-1.71850497902936	2.59787721489811	4.42131410996420
H	-1.84646079858448	1.78601165936357	3.72244019022727
C	0.98419265743704	4.94998720523128	4.98791347054219
C	-1.18726133512993	7.15962626210551	1.14289969388686
H	-0.59953845447900	7.94654115143854	1.61642567154484
H	-1.21601932317664	7.39804918767862	0.07673757207247
C	-0.54333477485800	1.13487009780432	7.76585932883428
H	-0.56220351008897	1.72373686133438	8.67202716838199
C	-2.74326543100382	4.66391195950830	1.29436927752375
H	-3.31976556345271	3.86617482026037	0.82809632202520
H	-2.68280019132470	4.42978372450975	2.35874867506563
C	1.39745425057588	6.06372603596026	5.71338660900431
H	0.79552292527056	6.45201964770066	6.51925831921154
C	-0.30547453242273	4.27400087356686	5.18683423563459
C	5.78694496658902	1.37889599650616	6.47247524799859
H	6.76134142885673	1.43756671380929	6.93598043490624
C	-1.25277625666939	4.66436140062114	6.12875444387120
H	-1.05812729162096	5.48762338130436	6.79710742397827
C	3.33923145981377	6.19088921011440	4.32896313090342

H	4.26368117170910	6.65933340435806	4.02767855444283
C	2.20576702427752	5.37398483876178	-1.80304360891510
H	2.31135914560154	4.49760976767314	-2.44457867801303
H	1.17020534872960	5.38818751954272	-1.46498804901882
C	2.88056647243951	5.07182818805623	3.65934391538667
H	3.41389773161748	4.64942925534025	2.81959107588834
C	-2.60434795313779	7.14561277752073	1.69768569489262
H	-2.56314288058977	7.02479836909446	2.78421613531240
H	-3.09296379141193	8.10168024161564	1.51239478328462
C	-3.42239449341344	6.01056845870725	1.09897985250704
H	-3.56425679396870	6.19073109292852	0.03046143651392
H	-4.41914770899622	5.98610808721238	1.53901166867690
C	2.58987675755288	6.68459015729359	5.38683759763941
H	2.92262716401043	7.54823487434436	5.94318265612730
C	4.60955140173067	5.21757274882398	-1.04636618058245
H	5.26106390500087	5.12293904781896	-0.17741677939234
H	4.78605269496446	4.33757495730332	-1.66783154536545
C	-2.69926774311802	2.93777600713406	5.33318285311057
H	-3.62524482104330	2.38506050931081	5.35950882138932
C	2.53955047825132	6.63599539892813	-2.58308895159338
H	1.88270526809421	6.71749634574023	-3.44908043501173
H	2.33564745202408	7.51051023323338	-1.95865896595448
C	-2.45566607918067	3.98647375239042	6.20681141084932
H	-3.19484167742471	4.27651552048253	6.93874067846603
C	3.99751894871674	6.65646178809342	-3.01879698644171
H	4.16932470737789	5.84975277976443	-3.73588532329888
H	4.22274525424550	7.58580778728469	-3.54140933028119
C	4.93332722233017	6.47915721949281	-1.83191496853949
H	4.84951096082032	7.34713581623618	-1.17187850977092
H	5.97020687363891	6.44784973789229	-2.16614352609846

[6]•

Rh	1.22900135568628	2.63512694318050	3.11211771301021
P	1.75527388996588	0.99866939740551	4.41091166786112

N	-0.36775692131370	3.41091263885778	4.27970783145461
C	2.27687801233117	1.79425929272818	1.47871900425425
N	1.98818857384636	4.47884862915578	3.92152214594627
C	0.91470310140446	-0.91217571636222	2.49057403198726
C	-0.74561228978246	3.28665839999284	0.56817363944410
C	-0.73893650241301	-0.40151677871066	0.82144700377715
H	-1.18049431525119	0.30751464179908	0.13632170801376
C	0.35053309999662	0.01212859270812	1.59572493454783
C	3.30254693322686	-0.27138762735362	2.55478796399085
C	2.06667428832092	-0.53511695420188	3.36398752751058
H	2.27122964796461	-1.34793726838496	4.05864582737499
C	-0.04945232846471	2.36011196733519	0.93595480270490
C	0.82939058370420	1.39895505991757	1.49156667884991
C	2.60107239709402	2.95540355480713	0.73090400279758
C	3.32935549802005	1.07232264205498	5.33975244629129
C	-0.49420847634163	-0.33433260558880	5.48359275236030
H	-0.51046939553557	-0.93344817737644	4.58745756892691
C	3.37403864351159	0.81268517564140	1.65876637573567
C	4.40276394717841	-1.10229083766438	2.73048709929916
H	4.33154846434030	-1.91519309982203	3.44105853971828
C	2.86769560870109	3.99277439180805	0.16390940407378
C	4.35674876287578	1.86842538546225	4.84137528874669
H	4.17200036654935	2.47117029725199	3.96693567885961
C	-1.49694898232860	4.51730442286465	0.45377004091867
H	-1.58174363753919	4.80735669776386	-0.60047768841443
C	0.40236766459886	-2.20430357895144	2.56157976449704
H	0.85102819238696	-2.90589737097511	3.25317105814978
C	-1.24862021726220	-1.68478053943659	0.91097199416159
H	-2.08898007625335	-1.97287875138444	0.29425813852751
C	3.56363731816212	0.27320906953871	6.45786179037141
H	2.77315249547887	-0.34985271444368	6.85124441131365
C	0.51826724187751	0.60417568682319	5.68215626197633
C	-0.74717371500350	5.64287825440972	1.19006427552158
H	-0.61154388371538	5.33355443935229	2.22647960688207

H	0.25105746754219	5.74834442847088	0.77004006005261
C	-0.67431736179994	-2.59857884285286	1.78393312372815
H	-1.05824969930259	-3.60642355330066	1.85896796888423
C	4.57409638294899	0.99451206285998	0.96109260967411
H	4.64290441449210	1.81721476743896	0.26576370099883
C	-1.49471763175378	-0.49482839139115	6.43200928569593
H	-2.26641577773843	-1.23503071941066	6.26972889316585
C	5.66212808844306	0.15883869825904	1.14265334317478
H	6.57128394638551	0.33580892683661	0.58395501319525
C	5.58356424379614	-0.89830686309914	2.03645781202275
H	6.42896243485345	-1.55270310821964	2.19665657773682
C	5.60153363299872	1.86709891995980	5.45025421420700
H	6.39426825125729	2.48467993666610	5.05137263262822
C	0.48501267206623	1.40343870740797	6.82959644674611
H	1.24698066819062	2.15475283311751	6.98144163376943
C	4.80738290367831	0.27997597793702	7.07075702379218
H	4.98063994476923	-0.33886563963044	7.94070196672024
C	3.12647602575937	5.24992530714356	-0.50201194520319
H	2.84836553493317	6.06308558673186	0.18179104462846
C	-1.50963108699405	0.28746860520134	7.57723966174035
H	-2.29421956283429	0.16449250917923	8.31084952656659
C	-1.55107750478246	2.79075281636380	4.40518482076624
H	-1.69608248838780	1.93878710305726	3.75818836127200
C	1.19590222338464	5.10453355285890	4.84789427199623
C	-1.51189543836265	6.95474394240666	1.13430070661434
H	-0.96826854581015	7.72095989050004	1.68901548472292
H	-1.56509305355641	7.30262821690301	0.09837934508848
C	-0.51954906825059	1.24407962768319	7.76705250566063
H	-0.53410665185677	1.87279740011287	8.64667151459526
C	-2.91902108704160	4.37290650364388	1.01431332632835
H	-3.45206529677305	3.59728853353040	0.46476337627157
H	-2.84298672200260	4.03121726651773	2.04778970139474
C	1.64684028862709	6.26488380844087	5.48852464061111
H	1.02413445206580	6.75275510933962	6.22303189813388

C	-0.09291582107965	4.49581366677074	5.06788525917502
C	5.82842268401398	1.07655962223555	6.56820710971862
H	6.79863262548579	1.07737565462308	7.04570623038749
C	-1.04013891137665	4.94136892591461	5.99760246934667
H	-0.81742417811925	5.78634406417365	6.63136707949805
C	3.65977618220084	6.16394889634122	4.20044513319851
H	4.61931856158434	6.56058525747458	3.90393764713861
C	2.27102466175235	5.40998194925581	-1.76745506750637
H	2.50943803829581	4.59094564142215	-2.44889184150261
H	1.21870593236710	5.30030318534913	-1.50995999692948
C	3.17685760081448	5.01850664493192	3.61226087121077
H	3.72901377379605	4.50339399817823	2.83866456244598
C	-2.92181297668475	6.79853791780393	1.68608830217160
H	-2.86338214276079	6.55523597564961	2.75062030082261
H	-3.46722243999704	7.74050350405945	1.61122821153547
C	-3.67585318344121	5.69143929847374	0.96271644076757
H	-3.82860171853925	5.98144746537297	-0.08098297523898
H	-4.66968120666145	5.56360075747326	1.39413506166355
C	2.87678305876983	6.79638005191721	5.17502628953321
H	3.22481889732186	7.69374137076113	5.66594746200268
C	4.61314818264967	5.43144565495939	-0.83757177502050
H	5.20568557737222	5.34348782888318	0.07324633785352
H	4.92099059804433	4.61222251803923	-1.49092251291350
C	-2.52037516166572	3.20080533867875	5.28851071689736
H	-3.45899110178586	2.67087454968957	5.34215486716576
C	2.53063675035756	6.74611481749503	-2.44552563314502
H	1.93200842698939	6.82486625006033	-3.35399830753550
H	2.19863087465375	7.55430167176509	-1.78725871936908
C	-2.25400835163019	4.30338317450972	6.10986994452859
H	-2.98631841337173	4.64664994051676	6.82631085597692
C	4.00708622075446	6.93351514291514	-2.76559350656526
H	4.30753644140383	6.19333549908170	-3.51239267214307
H	4.17761775913245	7.91263802032537	-3.21541889986337
C	4.86935419376200	6.76549313329892	-1.52223518667854

H	4.65156516469062	7.57621213464115	-0.82089776526372
H	5.92614145593758	6.85576299609103	-1.77730813218772

[7]

Rh	1.46627304820894	1.75238289559378	5.56808045077238
Rh	0.97748154351770	2.43423494667649	2.99321102244889
P	2.10673661886405	1.86064457927400	7.65751278316330
N	-0.29649304808023	0.61137697937204	5.63887111066634
C	0.53666394427226	-0.26215189077927	1.90193734253714
C	4.04233760239695	3.81367768372996	7.05507818793328
N	2.15593598858601	-0.16336339949009	5.10512515849207
C	-0.78077471927617	0.20843728450912	2.19566087587656
N	-0.84129327711560	1.50052787844492	2.67801200788075
C	-0.12077699374277	5.70613547006588	6.66626007620053
C	6.44877779303908	4.30857544735021	5.70828419746751
H	7.38045695745780	4.47452602829332	5.18407243565146
N	1.53790509421360	0.66045961964501	2.11081551668556
C	0.82048427032686	4.66114442756017	6.66977832947195
C	3.86761605564171	-1.77040036150690	4.68996850020053
H	4.91673750205582	-1.96897201230385	4.53244136887305
C	2.99099952227394	4.47524455058116	1.43884219967427
H	2.76988867966902	5.53723010621205	1.29554423603032
C	3.35775847598909	0.61618138907698	8.11358222553409
C	4.50871430293780	4.34742033754470	1.49831452654777
H	4.89021330790538	4.90771006300778	2.35104806035207
H	4.76421310541601	3.30035562805418	1.67953105713732
C	1.72881264669480	4.59834044814673	7.75282609092509
C	0.85152323827418	-1.54545502143153	1.43797089217142
C	1.65602977509697	5.56631264786345	8.75067529946807
H	2.35111874901533	5.50911591870400	9.57825659678472
C	-1.52349454611608	1.07887818170270	5.90629745989409
H	-1.58884981840416	2.14721179965581	6.03706556405955
C	5.39745755506882	3.68653839637121	5.06476813675144
H	5.50909567627413	3.35378358559870	4.04461816505658

C	5.16756615878680	4.81973763693538	0.21077271121468
H	6.24883041262830	4.68609885857123	0.27350770551395
H	4.99815623343555	5.89436995577582	0.09534515313943
C	3.80094847798258	-1.73724186103170	8.44292489260998
H	3.43723257261317	-2.75334155796442	8.50953969060292
C	2.81938479536279	3.55428051519499	7.90256816484100
H	3.14237305566509	3.59703990835969	8.94205117302018
C	-3.22236851722871	1.31064206955283	2.84811788611328
H	-4.15767557940454	1.77790740318790	3.11841204268480
C	0.60300498720175	7.00002198948790	2.92477740627535
H	1.68198322909245	7.15955740650950	2.92480444874591
H	0.29704689399924	7.00323778687972	3.97243375667819
C	1.24705997191405	1.24768335072563	10.28496900546256
H	2.22557773853866	0.81849933072809	10.44065612770437
C	-1.95385549195449	-0.53481277982771	2.02668329169262
C	4.16413064399317	3.44678619604403	5.69771472533866
C	-1.26600411819149	2.36003085378263	9.90793743684687
H	-2.24197086206731	2.80044180399260	9.75678989246253
C	-1.60762678974917	7.88139704775773	2.11884046973722
C	0.72582066912038	6.59219403037720	8.72141056541207
C	0.90990768139656	1.77925883504111	9.04031176672790
C	-2.63493972137990	0.27460166475627	5.98107692852128
H	-3.59977465559227	0.71139093112619	6.18907562481882
C	5.61261693108005	-0.16511945197778	8.46805288242520
H	6.66757094355075	0.05277312620880	8.56453961030236
C	3.44179352924762	-0.48016533710310	4.90407493478302
H	4.12962886406597	0.35023536800063	4.92317523328470
C	-0.35599492154842	2.33526428333527	8.86336748606188
H	-0.61536272674472	2.76449552571000	7.91099528773047
C	4.61156277010010	4.09361309650746	-1.00547761770744
H	4.88216823329148	3.03537535678801	-0.94621584679192
H	5.06639853621824	4.47836226540392	-1.91967126928042
C	2.44665661931143	3.73154820270917	0.21772911278229
H	1.36368045702109	3.83356161145817	0.16858505790676

H	2.63521330142614	2.66599471629121	0.34911689389481
C	-0.10564158948580	8.11850173154538	2.17612902091818
H	0.11237014219099	9.07841921878310	2.64671496568795
H	0.28688401770064	8.17821786926040	1.15665692973411
C	6.30295120707876	4.71257062717050	7.02895872238180
H	7.11248665507170	5.20350456923092	7.55080287626076
C	-1.92315604976262	6.52256685325849	1.51108428820804
C	-3.17773477292328	0.00682621844744	2.33590306751244
C	-1.23357053373733	-1.58883824550979	5.47366637687020
H	-1.10266636546433	-2.64376783631709	5.28827972279115
C	0.28610622069115	5.62287441338522	2.32899209748280
H	0.66378054180940	5.61347822174566	1.30597111316496
C	2.15391812766475	-1.89552948226084	1.18218826345772
C	5.10793295722094	4.45811833242710	7.68080454054896
H	4.99663783401937	4.75380028364736	8.71628804015415
C	4.72072710449448	0.87508343290256	8.23803702571147
H	5.09551721168837	1.88086470505646	8.14392966354839
C	-0.92815752214856	1.81949599555223	11.14077138947433
H	-1.64011525262548	1.83407516458253	11.95452088831826
C	-0.17028654243960	6.65994964685638	7.66383676313907
C	3.09606293708096	4.21436590391488	-1.07056072082759
H	2.70497536421620	3.65059100917413	-1.91882230512473
H	2.82485441684197	5.26032230755459	-1.24327068272211
C	3.16287557331473	-0.94374503963449	1.39896210580235
C	-1.21652400504060	5.39770488930120	2.25193118108174
H	-1.61187030704311	5.32087504481986	3.26810172389767
H	-1.40756162807908	4.44215496510813	1.76640815578920
C	1.21611257847113	-1.15618508364488	5.12262545650600
C	2.90998253788368	-0.70569215883432	8.21010369021956
H	1.85741201757261	-0.92404637291487	8.09407657017680
C	-0.13483332203245	-0.72931038429755	5.40523364286785
C	0.33083985061036	1.26433766053946	11.32610868669293
H	0.60363880176220	0.84624387561712	12.28560262076989
C	-2.48733861401993	-1.09535887019856	5.75637642776525

H	-3.33843558339567	-1.75887170380849	5.80246990674140
C	1.59975410290224	-2.48216189739407	4.90776968320205
H	0.85808583743759	-3.26571742668805	4.91649476920124
C	2.81359201211098	0.29461105998231	1.86695826498053
C	2.92191311931545	-2.79505799791376	4.68360607953813
H	3.21801275773170	-3.82026506936205	4.51457756060093
C	-2.04825088442204	1.99927599455375	3.01296405244086
H	-2.03179744064181	2.99260653680501	3.43006944370670
C	5.15886708915380	-1.47067193016251	8.57390219264965
H	5.85683729143418	-2.27753505000256	8.74987406276413
C	3.09245397588299	2.84787956445105	4.92973254076508
C	2.33282964060535	4.06709957496802	2.73718948761490
C	0.54347981664796	3.99766717420215	4.31791636865485
C	1.00479959555628	4.57391890619448	3.12670859490751
C	0.77616810690443	3.69537744674365	5.58804801082220
C	2.82832945538914	3.14187992801733	3.66485300294945
H	-2.01591875323610	7.92487788730496	3.13281317967371
H	-2.09807104124314	8.67502983654582	1.55310044131006
H	-1.60738649155363	6.51711830627808	0.46376194585313
H	-3.00015284846942	6.34689435174027	1.50642338940820
H	-4.08323990995890	-0.56500536052352	2.19466435740159
H	-1.89362703006871	-1.54334113969102	1.64654943732111
H	2.39576575397430	-2.88517564643044	0.82297545108911
H	0.06224831729634	-2.26773709187993	1.29382990542519
H	4.20145675188057	-1.17288995917748	1.21357426998725
H	3.55347843134958	1.04985331680576	2.08454534942692
H	0.69309086945616	7.31781464442577	9.52201844881473
H	-0.91219114637535	7.44633692963455	7.62312006017604
H	-0.83747076189038	5.72901560129474	5.85799492804855

[8]$^{+}$

Rh	1.52805458367348	3.14170644506447	3.81097365925307
Rh	1.18205690899579	5.35517194920331	2.34023475225142
P	1.92341178394312	1.11896649459023	4.53474849450672

N	-0.25450126361399	6.17150247816954	3.55188318926818
N	2.27344351281134	6.84918510685114	3.33736488754149
N	0.07116017728089	3.63130776895061	5.27761442263261
C	2.24933322443200	2.66321560656479	1.88980439319318
N	2.61737935366197	4.29959707803425	5.26293415635087
C	0.58962842950623	-0.01192501956764	2.31854688302088
C	-0.94582139024547	4.12106038461212	0.88897481059402
C	-1.17439468034434	1.13069916522732	1.13332376262141
H	-1.59185541622217	2.04662536653161	0.72094275401664
C	0.06407425019968	1.20096474438415	1.80386311724026
C	3.07417508035553	0.28026474500850	2.19664266438651
C	1.89289125485584	-0.06400262918884	3.07148642646634
H	2.03726235670774	-1.08029926815506	3.46840297752444
C	0.08595040160482	3.78685343179887	1.61679739439051
C	0.74254291964899	2.50679696359337	1.97442811578774
C	2.56496472418509	3.97757350895650	1.41805219293083
C	3.55862011193083	0.72573091573850	5.25456687952041
C	-0.38993474442068	-0.22487197070166	5.50831398412851
H	-0.52643318738634	-0.64330490360697	4.51229777898958
C	0.18244985929581	7.13353920638132	4.44543774744143
C	3.21201206125528	1.57184853374437	1.63236166723837
C	4.07231729651302	-0.67748240260048	1.99447103171458
H	3.95670842801205	-1.65883943610866	2.45925225056282
C	1.56973837443328	7.51033406738933	4.32564715943598
C	2.75731283747571	5.12033299588132	0.92455603219467
C	4.68448493528219	1.36553739364482	4.71314467460833
H	4.54375481719776	2.09607483972066	3.91753273580089
C	-1.96579943135417	4.57450602167880	0.17588747009272
C	-0.10238499933458	-1.21491396627155	2.12980829487286
H	0.33385257789050	-2.13432221445312	2.52805150731226
C	-1.86437138360846	-0.06877106263000	0.97110293920533
H	-2.82007750628185	-0.07223506779463	0.44385977355584
C	-1.54705643551293	5.78786297376196	3.59343972364258
H	-1.81426955510608	5.01645570193478	2.87400623267656

C	3.73588715279486	-0.23993055993539	6.25629601960071
H	2.86880041270065	-0.74902204789584	6.67869287895076
C	0.74009832533361	0.55388077950584	5.79699749520919
C	-1.80135156200901	5.03950220724945	-1.25659314130864
H	-2.05460857112945	6.11614988849373	-1.31804232842257
H	-0.75229072450554	4.93577711773849	-1.56385984485936
C	-1.32769494586063	-1.25849636668043	1.46594021425046
H	-1.85097719128178	-2.20685068061641	1.33739693003649
C	4.36437601464174	1.83794764407159	0.86859081683777
H	4.47747198896169	2.83127273402188	0.43281788586337
C	-1.36362993814442	-0.44216622473450	6.48757219942549
H	-2.23663556474872	-1.05163970795697	6.24884146056420
C	5.35421110102870	0.87611387891190	0.67672149711019
H	6.23674014183340	1.11850517838883	0.08235974936502
C	-0.71236108471706	7.68347763925353	5.38394877410757
H	-0.35197582332347	8.42700517024223	6.09357447479848
C	5.21233740027709	-0.39324448574794	1.24217883575515
H	5.98343728700974	-1.15241293781019	1.10736720822281
C	5.96404452981062	1.04803160436816	5.16557180758824
H	6.83093117354255	1.54470367177296	4.72772861754146
C	3.59167816397101	7.09868861298091	3.21502998432791
H	4.10769782075848	6.50527774600814	2.45996054338645
C	0.86580969511736	1.12549972991889	7.07845287750766
H	1.72708679306406	1.75667885066730	7.30478049376246
C	-2.46568456713619	6.30733889132535	4.48770068473777
H	-3.49429001166519	5.95160503819243	4.46866151388997
C	5.01775733833241	-0.54813630115069	6.71711740867339
H	5.14521264571545	-1.29627854345662	7.50119316715391
C	3.23638421907041	6.09906964462440	-0.06396353378174
H	3.45494688744238	7.05672524560153	0.44631013231190
C	-1.22972047656162	0.12445141358142	7.75584188714570
H	-1.99735608984404	-0.03743252140794	8.51372590423424
C	-1.18057044891101	3.14518824212471	5.25317363933687
H	-1.42875765061760	2.53649116206752	4.38289120638999

C	2.21167978581896	8.45773008866221	5.14459971986156
H	1.64272253640179	8.97492156520103	5.91594768471632
C	1.87330980032401	4.80680587118764	6.30245035123606
C	-2.73507904678160	4.26267396282339	-2.20155334500340
H	-2.65601335065131	4.66182994423127	-3.22555515683663
H	-2.40636731859325	3.21010660146070	-2.24037582234728
C	-2.03269879352663	7.27356533357437	5.41734918281695
H	-2.72258040182732	7.69435255869521	6.14892671831311
C	-0.11083899366288	0.91250749425820	8.04709923289697
H	-0.00544440577826	1.37351470007885	9.03001675591145
C	-3.39452160841541	4.58226050525865	0.68179407513020
H	-3.44642582123987	4.14887462774921	1.69105385587136
H	-3.75304170885260	5.62755999878974	0.75737331981767
C	2.47209894783095	5.64584430403742	7.26119261166535
H	1.86908247239394	6.05808296284819	8.06932319730975
C	0.48159695433785	4.39529713366415	6.33736825008692
C	6.13344042334798	0.09435740194996	6.17326416033288
H	7.13396898166614	-0.15030529826564	6.53305145182606
C	-0.41621068432703	4.69662399275478	7.37869089096213
H	-0.08896635796088	5.30766340592639	8.21881383436824
C	4.56303246123358	5.44086894735769	6.09301621611752
H	5.61904154478726	5.67165494626324	5.96540190055307
C	2.13070128546585	6.38635954746078	-1.10515389009198
H	1.85358425434266	5.43165022512307	-1.58277873606401
H	1.23239200097168	6.74620255209819	-0.58225328460410
C	3.91774417025186	4.62259907066954	5.17692461403155
H	4.44333945612040	4.21650614869876	4.31378897188217
C	-4.18829254754810	4.32315985710256	-1.71649662861820
H	-4.54195815252177	5.36913191302752	-1.76216650396000
H	-4.84048179424843	3.74236637304186	-2.38774572668497
C	3.56017025309135	8.72708199799989	4.97980907459268
H	4.05785854102239	9.46200809765679	5.61250571855165
C	4.27385549145331	8.01811028068473	3.99810269470579
H	5.34027580831971	8.17544657928477	3.84163784479391

C	-4.32140344938526	3.81186157418292	-0.27712314755769
H	-4.05417110701663	2.74164832881735	-0.24564606853624
H	-5.36630783253098	3.89057047716521	0.06376240616178
C	3.81484803299083	5.96795525931582	7.16107652868638
H	4.27954604781479	6.62570299686970	7.89557753872754
C	4.52778376061605	5.62171473379861	-0.75718057530678
H	5.31181999973465	5.45174077714772	-0.00286854367886
H	4.32599343934062	4.64713650150030	-1.23349322634526
C	-2.10358050298697	3.39975516946643	6.25743674759745
H	-3.10495055418692	2.97751597659266	6.19331843752535
C	2.60581106130235	7.38934423250146	-2.15966002919902
H	1.81319640006386	7.55086462888234	-2.90649420026224
H	2.78606679832115	8.36630794248870	-1.67695062981267
C	-1.70773339869547	4.19676019710334	7.34397451129726
H	-2.40443381380807	4.42028816624959	8.15214874172237
C	3.89560093235755	6.91720364733044	-2.84209384144916
H	3.68535767513790	5.99603008734630	-3.41353225016173
H	4.24178857857519	7.66720906893085	-3.57024653785044
C	4.99602574145995	6.62886167737951	-1.81341510178971
H	5.28111213192945	7.57136879354138	-1.31301454089808
H	5.90198116705022	6.25365841197484	-2.31450384969772

[9]

Rh	1.29574528663557	3.04862956370964	3.81350355507610
Rh	0.75895520565945	5.19036748537474	2.27145409475328
P	1.66409620097401	0.99964488567389	4.56318863815925
N	-0.48547353850985	6.30006943707379	3.59720554891213
N	1.89288195343006	6.87306754799314	2.69947604917485
N	0.23872466699922	3.74351749782005	5.52024488470495
C	2.16677760986670	2.70031092542075	1.89264769019812
N	2.62341186150527	4.45574658164479	4.69917340768347
C	0.87832818468947	-0.22326055477738	2.12357292197673
C	-0.97845234591048	4.18175237531076	1.56952599603844
C	-0.82021920063626	0.78182131274747	0.73777301249090

H	-1.26661719109158	1.66988917818580	0.31687456190249
C	0.28066920303606	0.92175759956227	1.58411736706905
C	3.26775547791264	0.47226608567262	2.41937643803686
C	2.03002414645916	-0.09525459912540	3.06108611575620
H	2.27105401360520	-1.08095326075905	3.45146487633888
C	-0.06526960114256	3.36426176296472	2.07300691705179
C	0.77611421120482	2.25318262778615	1.91144643409305
C	2.19865953589715	4.07417298794978	1.31848490910846
C	3.11930097871764	0.55303064702843	5.58652764666958
C	-0.91862008950097	-0.08991540112896	4.83298344936714
H	-0.97579847969419	-0.06238660944063	3.75769253721097
C	0.12396235364519	7.36220601648281	4.20839460817865
C	3.31511440577144	1.77726575800110	1.87097155397071
C	4.41342571472755	-0.31354514098210	2.43747189733698
H	4.35794900735432	-1.29871912854492	2.88137491916322
C	1.44947046761966	7.66015897039806	3.73910375806077
C	2.87839505443982	4.51526241206929	0.30743449111855
C	4.11318052525384	1.50146540026493	5.79464299917069
H	3.97751406324878	2.49798813160578	5.40892186706902
C	-2.18109776912671	4.53990643848614	1.13071418868220
C	0.36584254823960	-1.47635632504512	1.80380331852782
H	0.82305293048467	-2.35779256480146	2.23385535685167
C	-1.32406680761843	-0.47052184668960	0.42884175279265
H	-2.17552368120979	-0.56104258270050	-0.23158020346505
C	-1.70682962009906	5.94015060906824	4.01832921806882
H	-2.12826636777878	5.08174238077154	3.52259378143025
C	3.29654778538366	-0.74097916946175	6.07673902614402
H	2.52621379343291	-1.48522518267304	5.92762487273413
C	0.26432923278171	0.26973697017438	5.48490090010380
C	-2.44817265925190	5.86153394723158	0.48593804242312
H	-3.09260990430743	6.46010884423876	1.14257479317213
H	-1.51567305399948	6.41069244845797	0.37712502336223
C	-0.72963062978235	-1.60632732997289	0.96431377407102
H	-1.11732786866254	-2.58803219238324	0.73030644467757

C	4.54740417115749	2.20923479301255	1.36335626400984
H	4.60721672404259	3.19150827899121	0.92896247898091
C	-2.03343618780154	-0.47423050779967	5.56159495471723
H	-2.93471507077287	-0.76396981658511	5.03869560707143
C	5.67935457237270	1.41232671560317	1.38772504804300
H	6.60745597166151	1.79309310433525	0.98290766644450
C	-0.54085643740062	8.08300373133830	5.20812203306392
H	-0.06086368178636	8.93446158385448	5.66501095887402
C	5.62043620993547	0.13914188646805	1.92888995936486
H	6.49677373552421	-0.49296569955548	1.96223985182324
C	5.27166381401035	1.16507556000344	6.48098046023929
H	6.03950283069535	1.91117099453996	6.63222443522611
C	3.12409422442059	7.11540487647633	2.20845810966239
H	3.41732602507923	6.47859774047576	1.39342047910041
C	0.28318242897056	0.27620845626039	6.88146818706124
H	1.17765556664013	0.57970718710640	7.40488118998634
C	-2.39828329052638	6.60398559912469	5.00474612396265
H	-3.37727685332657	6.25689638662766	5.29810294143329
C	4.45131310653434	-1.07556593683678	6.76509127888858
H	4.58029421746029	-2.08042554961078	7.14386288667996
C	3.48995588999473	5.00093144945039	-0.74815974019878
C	-1.99962678309992	-0.48062538493997	6.94994303483387
H	-2.87312558356148	-0.77482234888262	7.51519177767644
C	-0.98986894820494	3.34014696883667	5.86226633232022
H	-1.43228211820006	2.59762607518807	5.21776647280838
C	2.26242505010026	8.66318929347482	4.26817040465595
H	1.90908607032992	9.25535405452590	5.09825837385371
C	2.20484251711357	5.04127973732657	5.86041247323898
C	-3.15391210794038	5.68327081216550	-0.85875786577592
H	-3.41660848090135	6.65761547702990	-1.27369392036761
H	-2.45742608698543	5.21941144188144	-1.56102053193615
C	-1.80082661407483	7.70932566471797	5.61229355167336
H	-2.31580185379109	8.26581191536979	6.38243247566947
C	-0.83903767386005	-0.09570990912790	7.60578669740449

H	-0.80491747987238	-0.08183271357520	8.68665554784008
C	-3.37247141800886	3.63328852351811	1.24762406875414
H	-3.08292211001013	2.67167204027004	1.66689648710011
H	-4.09283192497569	4.07894056069163	1.94704638990615
C	3.06144297471923	5.88906171676645	6.57067557928330
H	2.73417896918553	6.33130922910584	7.49932905831936
C	0.87352022413500	4.68061875727613	6.28491180346619
C	5.44268076839586	-0.12216943800714	6.96715162368232
H	6.34540223263909	-0.38607118633117	7.50076903742023
C	0.23284846379620	5.21164609156498	7.40955309439131
H	0.72673345510385	5.96580014169593	8.00165624670099
C	4.72474657016297	5.56814525984864	4.88703195055564
H	5.69295691269387	5.77175083181557	4.45525752947214
C	2.86297141075196	4.96861213331340	-2.11702754345852
H	3.46202391696781	4.32884955268338	-2.77620962648298
H	1.86876758879696	4.53121947955610	-2.06278255586040
C	3.85973754368988	4.72054810061014	4.24312162968377
H	4.12594650229259	4.23898835793865	3.31442459432229
C	-4.39447445285696	4.81058059672013	-0.72846407601408
H	-5.13129593600037	5.32394091195431	-0.10298678188210
H	-4.86318929598168	4.67015803234550	-1.70375260606657
C	3.51510573261369	8.89863569651025	3.74799366334034
H	4.14168368067268	9.67902956005225	4.15460474840734
C	3.94926220143215	8.10488790527357	2.68318548751970
H	4.91635709205676	8.25370449007520	2.22563243816248
C	-4.06641023095179	3.46178594955108	-0.10330558128461
H	-3.40576045144100	2.90480767364530	-0.77224979893186
H	-4.97232136331594	2.86438618812218	0.01501958540747
C	4.31725455214965	6.16930027073219	6.08499570779664
H	4.97736880748363	6.83357596272698	6.62305440791703
C	4.84224065944622	5.66275882958989	-0.70836319298382
H	5.22547630230073	5.70254813766520	0.31090802167290
H	5.55089354578018	5.05414711562064	-1.28319240558694
C	-1.65736446901595	3.82432898890910	6.96620438671586

H	-2.64451225789737	3.45557451782112	7.19919678818656
C	2.81001230730244	6.37289224997183	-2.71685191412086
H	2.40648796059781	6.33246000776755	-3.73035028595999
H	2.12145603039511	6.98139033504822	-2.12609145182290
C	-1.02628586182511	4.78181240272582	7.75886368994200
H	-1.52129677455695	5.19455071045326	8.62618615833984
C	4.18524196728147	7.02581429384381	-2.72211925763595
H	4.84670791909508	6.46331565844244	-3.38823910483708
H	4.12416128464344	8.03609449891742	-3.12996375204452
C	4.79442642686012	7.05988591342213	-1.32715958252941
H	4.19289460994608	7.71066390756901	-0.68880544703592
H	5.79781488885890	7.48943532225078	-1.36084475767643

[**4**]

Rh	1.45962999979438	3.01767326631210	3.84386620815070
Rh	0.87887153682568	5.14033091135215	2.18479427425095
P	1.99392796222322	1.08003072362570	4.69165276098524
N	-0.56732432475565	5.94213620286274	3.36342946993238
N	1.93426800753424	6.59746296965564	3.13233956332080
N	0.04012237414930	3.58988052130387	5.29376552125977
C	2.16140696248675	2.48174807289443	1.94825367229169
N	2.55060103076545	4.30877661922875	5.14142633490837
C	0.67508598153839	-0.26794821498242	2.59502652971931
C	-0.57381989916653	4.23390145537318	0.88069748311937
C	-1.19946136743851	0.71482509660016	1.46083385784776
H	-1.68766668626033	1.58505669517560	1.05044520679690
C	0.06485512464876	0.87201562649198	2.04282724341474
C	3.11155750908241	0.17332335782619	2.35640373419990
C	1.99484007938339	-0.18642880149819	3.29345675661859
H	2.21961903551588	-1.15226661553901	3.74228924521851
C	-0.06243116275562	3.30475294678517	1.54462798312066
C	0.67514736854582	2.20711027159700	2.09365224145446
C	2.36873120958887	3.76864545290401	1.36213587554473
C	3.66115828440406	0.79296259046711	5.39336408391677

C	-0.17931774587804	-0.36246145139768	5.82419221884779
H	-0.30476766551846	-0.85922335445806	4.87646177089833
C	-0.13505387537263	6.92156141044969	4.24111196137313
C	3.16370537822335	1.42989798836058	1.72074210245487
C	4.13592099905839	-0.74347954715398	2.15511402136031
H	4.08936725632353	-1.69451606206687	2.66908417522476
C	1.23113912965421	7.28449824041207	4.11301492476463
C	2.44377220995951	4.84618892781881	0.73105470166639
C	4.75349746286293	1.39593769654686	4.77100455329211
H	4.58776668662823	2.03677856478399	3.92101475102969
C	-1.40114866810808	4.81127351361195	-0.18432298590683
H	-0.77046553379455	4.83736509090232	-1.08054939981169
C	0.04474873064105	-1.50467902554522	2.49709132747925
H	0.53921141560962	-2.37371634639506	2.91260096472014
C	-1.82904673691879	-0.51533540406921	1.39456531868046
H	-2.80458527778925	-0.59467434610994	0.93419810477629
C	-1.84684529106618	5.52589692389977	3.43691027573547
H	-2.10414219398239	4.72190434568056	2.76661112345460
C	3.88515340960057	-0.06696534065636	6.46773402380830
H	3.05170897533494	-0.55155580009900	6.95383283840804
C	0.87005889812976	0.53281298894514	6.01562321673959
C	-1.84808831661072	6.25038220655260	0.07783058631250
H	-2.47179217919296	6.27574186908787	0.97140353021835
H	-0.97675873748366	6.86306623309450	0.30279218954096
C	-1.20037234148196	-1.64201678536615	1.90499334013667
H	-1.67147865431328	-2.61327225557627	1.84689522595890
C	4.26356273210177	1.69854562965493	0.89803510010128
H	4.31555110281567	2.65755509886612	0.40627639912245
C	-1.08879542753185	-0.60390739492392	6.84583090601078
H	-1.89550191883150	-1.30582893482632	6.68340874353678
C	5.27971406041817	0.77743081773014	0.71031088752153
H	6.11481734008758	1.02369690301085	0.06835723392049
C	-1.04624321987127	7.49041909687286	5.14575510748373
H	-0.70541315824458	8.25640508600828	5.82568213354373

C	5.21975687897406	-0.45636216351259	1.34062212896343
H	6.00835765486385	-1.18380084812810	1.20896757434761
C	6.04171839427008	1.14981818654698	5.21613223700496
H	6.87841553931060	1.61895151747225	4.71708206164540
C	3.24302775004256	6.87399814149821	2.96944058438941
H	3.75475325951272	6.26602769009515	2.24118982555322
C	0.97567730894293	1.20006740756934	7.24123696947379
H	1.76821110480054	1.91904086137332	7.39390190127224
C	-2.75962401275572	6.04880450339082	4.31122421100594
H	-3.76768347807462	5.66364569171689	4.32532713141539
C	5.17531311277040	-0.30258482585334	6.91967894445545
H	5.33555819994082	-0.96740093090383	7.75766599944215
C	2.94177741008775	5.75877025618348	-0.29845401054824
H	3.13105186743473	6.73477931122466	0.16267452286014
C	-0.96935063087055	0.05052744243534	8.06183347008269
H	-1.68360985004824	-0.13387426589953	8.85231543888894
C	-1.19826293230573	3.08761898727427	5.34592178762658
H	-1.45773544301010	2.40910130092117	4.54655419241751
C	1.88107584460868	8.26817980506541	4.87080502366304
H	1.32909065592907	8.79679188893240	5.63307943183559
C	1.84554488803764	4.83765167106595	6.19232581298540
C	-2.62957724018598	6.80491714698636	-1.10155420043462
H	-2.94997221005727	7.82557852302418	-0.88944958950575
H	-1.97409611366856	6.86283163376965	-1.97583554098116
C	-2.34785455595404	7.06780257595983	5.18942677678215
H	-3.04298326689337	7.50711356856242	5.89013829901844
C	0.06514871436569	0.95873204970971	8.25333215915800
H	0.15527188686180	1.48992188627251	9.19071485781977
C	-2.60732329315186	3.92436777987552	-0.51108842028374
H	-2.26727538545603	2.91513694812548	-0.74052349619445
H	-3.23952365097268	3.85098792983173	0.37747092423998
C	2.48997018993319	5.65953322575868	7.12313290776540
H	1.93201040420700	6.07424737457558	7.94869432488558
C	0.46631168481577	4.43337338293480	6.28106081412396

C	6.25662904732518	0.30574131267925	6.29681831064481
H	7.26164602688562	0.11844597697807	6.64926300416419
C	-0.40744972984693	4.79226424247287	7.31354528456493
H	-0.07746156388569	5.46616030012060	8.08918586918506
C	4.52616193170332	5.41762328925001	5.89923720450869
H	5.56976376650158	5.63757780940294	5.73395515208233
C	1.90211680704248	5.99214413152861	-1.39937926383879
H	1.64565281203535	5.02766309132138	-1.84321764725630
H	0.99420687530088	6.38408615342965	-0.94723502714139
C	3.84918324834092	4.60887419717364	5.01862706598678
H	4.34001924217557	4.19387949968484	4.15285760892416
C	-3.83070776520166	5.92957476096243	-1.43098781102383
H	-4.53628398037854	5.96344552073149	-0.59593624964628
H	-4.36218637536516	6.32019761442254	-2.30004826866151
C	3.20395385578055	8.55933150967706	4.66201731969552
H	3.69690785713219	9.32196482931873	5.24706784295004
C	3.90832351366736	7.83095055574283	3.68851885840967
H	4.95759735061421	8.00458386412606	3.50293375584172
C	-3.41193868516094	4.48687356973681	-1.67448298711804
H	-2.80478018725655	4.43780037637461	-2.58271550931228
H	-4.28681702787931	3.86088647646095	-1.85670063554500
C	3.82382578907136	5.96105889709493	6.98003976079486
H	4.31827432346574	6.60394424676475	7.69378435332178
C	4.26027759441753	5.26378793886554	-0.90136299589407
H	4.99826133284685	5.13262890635050	-0.10905757482550
H	4.09177305671986	4.27818030894395	-1.34082728239846
C	-2.09246370224227	3.40464267939773	6.34179660963428
H	-3.08150818558103	2.97271027170395	6.33441503983953
C	2.42484804255984	6.93839569194783	-2.46860343506595
H	1.67746889577027	7.05941310129663	-3.25405994871633
H	2.57211059745354	7.92897561660309	-2.02927889531003
C	-1.68498128219376	4.28515572596531	7.34732069118356
H	-2.35977836828707	4.56258991292879	8.14441640183008
C	3.74065207176922	6.45038047271963	-3.05741905551752

H	3.56792065795736	5.51323615331827	-3.59393925210571
H	4.11655408061425	7.16331767535694	-3.79254881491587
C	4.77787347283503	6.21704682531864	-1.96817253454420
H	5.03359351886651	7.17324739170155	-1.50302632871038
H	5.70149252655183	5.82730771618914	-2.39859508966475

Crystallographic data

*Crystal data and structure refinement for **1**.*

Identification code	2313840
Empirical formula	$C_{172}H_{164}P_4$
Formula weight	2354.90
Temperature/K	100.01(10)
Crystal system	monoclinic
Space group	$P2_1/c$
a/Å	34.4780(2)
b/Å	10.96194(4)
c/Å	38.1963(3)
α/°	90
β/°	116.6055(9)
γ/°	90
Volume/Å^3	12907.50(17)
Z	4
ρ_{calc}g/cm^3	1.212
μ/mm^{-1}	0.963
F(000)	5024.0
Crystal size/mm^3	0.25 × 0.21 × 0.17
Radiation	Cu Kα (λ = 1.54184)
2Θ range for data collection/°	4.66 to 160.554
Index ranges	$-44 \le h \le 43$, $-13 \le k \le 13$, $-48 \le l \le 48$
Reflections collected	514049
Independent reflections	27919 [R_{int} = 0.0953, R_{sigma} = 0.0330]
Data/restraints/parameters	27919/0/1585
Goodness-of-fit on F^2	1.050
Final R indexes [I>=2σ (I)]	R_1 = 0.0450, wR_2 = 0.1155
Final R indexes [all data]	R_1 = 0.0551, wR_2 = 0.1214
Largest diff. peak/hole / e Å^{-3}	0.32/-0.41

*Crystal data and structure refinement for [**2**].*

Identification code	2313435
Empirical formula	$C_{100}H_{98}Cl_4P_2Rh_4$
Formula weight	1915.16
Temperature/K	100.00(10)
Crystal system	triclinic
Space group	P-1
a/Å	11.9627(2)
b/Å	13.1950(2)
c/Å	14.07830(10)
α/°	96.1990(10)
β/°	101.4310(10)
γ/°	104.3630(10)
Volume/Å^3	2080.97(5)
Z	1
ρ_{calc}g/cm^3	1.528
μ/mm^{-1}	8.216
F(000)	976.0
Crystal size/mm^3	0.15 × 0.04 × 0.035
Radiation	Cu Kα (λ = 1.54184)
2Θ range for data collection/°	6.494 to 160.568
Index ranges	-15 ≤ h ≤ 15, -16 ≤ k ≤ 15, -17 ≤ l ≤ 17
Reflections collected	32201
Independent reflections	8866 [R_{int} = 0.0517, R_{sigma} = 0.0465]
Data/restraints/parameters	8866/136/590
Goodness-of-fit on F^2	1.166
Final R indexes [I>=2σ (I)]	R_1 = 0.0435, wR_2 = 0.1256
Final R indexes [all data]	R_1 = 0.0465, wR_2 = 0.1282
Largest diff. peak/hole / e Å^{-3}	1.11/-1.98

*Crystal data and structure refinement for [**3**].*

Identification code	2313858
Empirical formula	$C_{45}H_{41}F_6O_6PRh_2S_2$
Formula weight	1092.69
Temperature/K	100.00(10)
Crystal system	triclinic
Space group	P-1
a/Å	10.3184(3)
b/Å	10.6661(3)
c/Å	20.2792(6)
α/°	100.752(3)
β/°	99.799(3)
γ/°	100.050(3)
Volume/Å^3	2111.09(11)
Z	2
ρ_{calc}g/cm^3	1.719
μ/mm^{-1}	8.271
F(000)	1100.0
Crystal size/mm^3	0.079 × 0.049 × 0.044
Radiation	Cu Kα (λ = 1.54184)
2Θ range for data collection/°	8.636 to 103.856
Index ranges	-10 ≤ h ≤ 10, -10 ≤ k ≤ 10, -16 ≤ l ≤ 20
Reflections collected	14157
Independent reflections	4623 [R_{int} = 0.0395, R_{sigma} = 0.0435]
Data/restraints/parameters	4623/0/560
Goodness-of-fit on F^2	1.033
Final R indexes [I>=2σ (I)]	R_1 = 0.0276, wR_2 = 0.0642
Final R indexes [all data]	R_1 = 0.0312, wR_2 = 0.0668
Largest diff. peak/hole / e Å^{-3}	0.38/-0.50

*Crystal data and structure refinement for [**4**](OTf)$_2$.*

Identification code	2313437

Empirical formula	$C_{73}H_{73}F_6N_4O_8PRh_2S_2$
Formula weight	1549.26
Temperature/K	100.00(12)
Crystal system	triclinic
Space group	P-1
a/Å	13.8174(4)
b/Å	16.2833(5)
c/Å	17.0904(5)
α/°	110.982(3)
β/°	105.836(3)
γ/°	96.016(2)
Volume/Å^3	3365.06(19)
Z	2
ρ_{calc}g/cm^3	1.529
μ/mm^{-1}	0.653
F(000)	1588.0
Crystal size/mm^3	0.14 × 0.07 × 0.025
Radiation	Mo Kα (λ = 0.71073)
2Θ range for data collection/°	3.682 to 52.744
Index ranges	-17 ≤ h ≤ 17, -20 ≤ k ≤ 20, -21 ≤ l ≤ 21
Reflections collected	78472
Independent reflections	13728 [R_{int} = 0.0750, R_{sigma} = 0.0658]
Data/restraints/parameters	13728/186/943
Goodness-of-fit on F^2	1.060
Final R indexes [I>=2σ (I)]	R_1 = 0.0562, wR_2 = 0.1212
Final R indexes [all data]	R_1 = 0.0796, wR_2 = 0.1346
Largest diff. peak/hole / e Å^{-3}	1.47/-0.87

*Crystal data and structure refinement for [**4**](OTf).*

Identification code	2313436
Empirical formula	$C_{66.58}H_{59.67}F_3N_{4.5}O_3PRh_2S$

Formula weight	1296.69
Temperature/K	100.15
Crystal system	monoclinic
Space group	$P2_1/n$
a/Å	9.93520(10)
b/Å	43.1896(5)
c/Å	13.45180(10)
α/°	90
β/°	98.4280(10)
γ/°	90
Volume/Å^3	5709.80(10)
Z	4
ρ_{calc}g/cm^3	1.508
μ/mm^{-1}	5.791
F(000)	2651.0
Crystal size/mm^3	0.091 × 0.04 × 0.038
Radiation	Cu Kα (λ = 1.54184)
2Θ range for data collection/°	4.092 to 160.818
Index ranges	$-12 \le h \le 12$, $-54 \le k \le 52$, $-10 \le l \le 16$
Reflections collected	53414
Independent reflections	12189 [R_{int} = 0.0439, R_{sigma} = 0.0372]
Data/restraints/parameters	12189/215/745
Goodness-of-fit on F^2	1.076
Final R indexes [I>=2σ (I)]	R_1 = 0.0642, wR_2 = 0.1794
Final R indexes [all data]	R_1 = 0.0690, wR_2 = 0.1835
Largest diff. peak/hole / e Å^{-3}	1.40/-1.16

*Crystal data and structure refinement for [**5**].*

Identification code	2313428
Empirical formula	$C_{128}H_{130}Cl_2P_2Rh_2$
Formula weight	2006.97

Temperature/K	100.01(10)
Crystal system	triclinic
Space group	P-1
a/Å	13.1434(3)
b/Å	15.1938(4)
c/Å	15.2523(3)
α/°	79.262(2)
β/°	65.253(2)
γ/°	67.904(2)
Volume/Å^3	2561.48(12)
Z	1
ρ_{calc}g/cm^3	1.301
μ/mm^{-1}	0.457
F(000)	1052.0
Crystal size/mm^3	0.234 × 0.155 × 0.125
Radiation	MoKα (λ = 0.71073)
2Θ range for data collection/°	3.62 to 56.976
Index ranges	$-17 \le h \le 16, -19 \le k \le 20, -20 \le l \le 20$
Reflections collected	59764
Independent reflections	11623 [R_{int} = 0.0467, R_{sigma} = 0.0403]
Data/restraints/parameters	11623/90/737
Goodness-of-fit on F^2	1.049
Final R indexes [I>=2σ (I)]	R_1 = 0.0344, wR_2 = 0.0751
Final R indexes [all data]	R_1 = 0.0412, wR_2 = 0.0790
Largest diff. peak/hole / e Å^{-3}	0.61/-0.54

*Crystal data and structure refinement for [**6**](OTf).*

Identification code	2313426
Empirical formula	$C_{55}H_{50}Cl_3F_3N_2O_3PRhS$
Formula weight	1116.26
Temperature/K	100.00(10)

Crystal system	triclinic
Space group	P-1
a/Å	9.65720(10)
b/Å	12.00310(10)
c/Å	22.2473(2)
α/°	100.3980(10)
β/°	96.5460(10)
γ/°	99.7380(10)
Volume/Å^3	2471.99(4)
Z	2
ρ_{calc}g/cm^3	1.500
μ/mm^{-1}	5.469
F(000)	1144.0
Crystal size/mm^3	? × ? × ?
Radiation	Cu Kα (λ = 1.54184)
2Θ range for data collection/°	7.634 to 161.9
Index ranges	-12 ≤ h ≤ 12, -15 ≤ k ≤ 13, -28 ≤ l ≤ 28
Reflections collected	93869
Independent reflections	10595 [R_{int} = 0.0658, R_{sigma} = 0.0309]
Data/restraints/parameters	10595/0/631
Goodness-of-fit on F^2	1.056
Final R indexes [I>=2σ (I)]	R_1 = 0.0459, wR_2 = 0.1252
Final R indexes [all data]	R_1 = 0.0505, wR_2 = 0.1284
Largest diff. peak/hole / e Å^{-3}	1.58/-0.85

*Crystal data and structure refinement for [**6**]$_2$.*

Identification code	2313427
Empirical formula	$C_{118}H_{122}N_4O_3P_2Rh_2$
Formula weight	1911.95
Temperature/K	100.00(10)
Crystal system	monoclinic

Space group	$P2_1/c$
a/Å	19.23010(10)
b/Å	25.78890(10)
c/Å	21.44500(10)
α/°	90
β/°	112.2980(10)
γ/°	90
Volume/Å^3	9839.81(10)
Z	4
ρ_{calc}g/cm^3	1.291
μ/mm^{-1}	3.441
F(000)	4008.0
Crystal size/mm^3	0.168 × 0.021 × 0.018
Radiation	Cu Kα (λ = 1.54184)
2Θ range for data collection/°	4.966 to 160.574
Index ranges	$-24 \leq h \leq 24$, $-32 \leq k \leq 30$, $-27 \leq l \leq 27$
Reflections collected	234983
Independent reflections	21411 [R_{int} = 0.0569, R_{sigma} = 0.0266]
Data/restraints/parameters	21411/73/1196
Goodness-of-fit on F^2	1.083
Final R indexes [I>=2σ (I)]	R_1 = 0.0458, wR_2 = 0.1176
Final R indexes [all data]	R_1 = 0.0483, wR_2 = 0.1191
Largest diff. peak/hole / e Å^{-3}	1.49/-0.94

*Crystal data and structure refinement for [**7**].*

Identification code	2313511
Empirical formula	$C_{68}H_{62}N_5PRh_2$
Formula weight	1186.01
Temperature/K	100.01(11)
Crystal system	triclinic
Space group	P-1

a/Å	9.8012(4)
b/Å	13.3777(4)
c/Å	21.7694(6)
α/°	102.651(2)
β/°	96.454(3)
γ/°	96.355(3)
Volume/Å^3	2740.32(16)
Z	2
ρ_{calc}g/cm^3	1.437
μ/mm^{-1}	5.510
F(000)	1220.0
Crystal size/mm^3	0.11 × 0.008 × 0.007
Radiation	Cu Kα (λ = 1.54184)
2Θ range for data collection/°	4.202 to 136.498
Index ranges	-11 ≤ h ≤ 11, -12 ≤ k ≤ 16, -26 ≤ l ≤ 25
Reflections collected	37113
Independent reflections	10005 [R_{int} = 0.1018, R_{sigma} = 0.0918]
Data/restraints/parameters	10005/0/685
Goodness-of-fit on F^2	1.008
Final R indexes [I>=2σ (I)]	R_1 = 0.0625, wR_2 = 0.1545
Final R indexes [all data]	R_1 = 0.0865, wR_2 = 0.1704
Largest diff. peak/hole / e Å^{-3}	2.26/-1.72

*Crystal data and structure refinement for [**8**](OTf).*

Identification code	2313512
Empirical formula	$C_{68}H_{67}F_3N_4O_4PRh_2S$
Formula weight	1326.85
Temperature/K	100.00(10)
Crystal system	monoclinic
Space group	$P2_1/c$
a/Å	21.5586(6)

b/Å	9.9941(3)
c/Å	26.8646(10)
α/°	90
β/°	90.466(3)
γ/°	90
Volume/$Å^3$	5788.0(3)
Z	4
ρ_{calc}g/cm^3	1.523
μ/mm^{-1}	5.733
F(000)	2725.0
Crystal size/mm^3	0.17 × 0.05 × 0.04
Radiation	Cu Kα (λ = 1.54184)
2Θ range for data collection/°	6.58 to 161.874
Index ranges	-27 ≤ h ≤ 27, -8 ≤ k ≤ 12, -34 ≤ l ≤ 31
Reflections collected	50039
Independent reflections	12345 [R_{int} = 0.0349, R_{sigma} = 0.0320]
Data/restraints/parameters	12345/164/958
Goodness-of-fit on F^2	1.114
Final R indexes [I>=2σ (I)]	R_1 = 0.0623, wR_2 = 0.1366
Final R indexes [all data]	R_1 = 0.0671, wR_2 = 0.1390
Largest diff. peak/hole / e $Å^{-3}$	1.07/-1.19

[53] B. Taljaard, J. H. Taljaard, C. Imrie, M. R. Caira, *European J Org Chem* 2005, 2607.
[54] H. Werner, M. Bosch, M. E. Schneider, C. Hahn, F. Kukla, M. Manger, B. Windmüller, B. Weberndörfer, M. Laubender, *Journal of the Chemical Society, Dalton Transactions* 1998, 3549.
[55] F. Neese, F. Wennmohs, U. Becker, C. Riplinger, *Journal of Chemical Physics* 2020, 152, DOI 10.1063/5.0004608.
[56] F. Neese, Wiley Interdiscip Rev Comput Mol Sci 2018, 8, DOI 10.1002/wcms.1327.
[57] R. A. Kendall, H. A. Früchtl, *Theoretical Chemistry Accounts: Theory, Computation, and Modeling (Theoretica Chimica Acta)* 1997, 97, 158.
[58] F. Neese, F. Wennmohs, A. Hansen, U. Becker, *Chem Phys* 2009, 356, 98.
[59] S. Grimme, S. Ehrlich, L. Goerigk, *J Comput Chem* 2011, 32, 1456.
[60] S. Grimme, J. Antony, S. Ehrlich, H. Krieg, *J Chem Phys* 2010, 132, DOI 10.1063/1.3382344
