# Supplementary material for: Structural Changes in the Carbon Sphere of a Dirhodium Complex Induced by Redox or Deprotonation Reactions
Source: Adv Sci (Weinh). 2024 Mar 23;11(22):2400072. doi: 10.1002/advs.202400072 (PMC11165463; doi:10.1002/advs.202400072)

checkCIF/PLATON report

Structure factors have been supplied for datablock(s) cs2_00rh2cl2_auto

THIS REPORT IS FOR GUIDANCE ONLY. IF USED AS PART OF A REVIEW PROCEDURE FOR PUBLICATION, IT SHOULD NOT REPLACE THE EXPERTISE OF AN EXPERIENCED CRYSTALLOGRAPHIC REFEREE.

No syntax errors found. CIF dictionary Interpreting this report

Datablock: cs2_00rh2cl2_auto

Bond precision: C-C = 0.0060 A Wavelength=1.54184

Cell:	a=11.9627(2)	b=13.1950(2)	c=14.0783(1)
	alpha=96.199(1)	beta=101.431(1)	gamma=104.363(1)
Temperature:	100 K		

	Calculated	Reported
Volume	2080.97(5)	2080.97(5)
Space group	P -1	P -1
Hall group	-P 1	-P 1
Moiety formula	C86 H82 Cl4 P2 Rh4, 2(C7 H8)	C86 H82 Cl4 P2 Rh4, 2(C7 H8)
Sum formula	C100 H98 Cl4 P2 Rh4	C100 H98 Cl4 P2 Rh4
Mr	1915.16	1915.16
Dx,g cm-3	1.528	1.528
Z	1	1
Mu (mm-1)	8.216	8.216
F000	976.0	976.0
F000'	980.32	
h,k,lmax	15,16,17	15,16,17
Nref	9106	8866
Tmin,Tmax	0.708,0.750	0.380,1.000
Tmin'	0.278	

Correction method= # Reported T Limits: Tmin=0.380 Tmax=1.000
AbsCorr = MULTI-SCAN

Data completeness= 0.974 Theta(max)= 80.284

R(reflections)= 0.0435(8051) wR2(reflections)= 0.1282(8866)

S = 1.166 Npar= 590

The following ALERTS were generated. Each ALERT has the format
test-name_ALERT_alert-type_alert-level.
Click on the hyperlinks for more details of the test.

Alert level C

```
PLAT213_ALERT_2_C Atom C91A            has ADP max/min Ratio .....        3.1 prolat
PLAT234_ALERT_4_C Large Hirshfeld Difference C4A      --C5A      .       0.19 Ang.
PLAT241_ALERT_2_C High   'MainMol' Ueq as Compared to Neighbors of        C47 Check
PLAT241_ALERT_2_C High   'MainMol' Ueq as Compared to Neighbors of        C90 Check
PLAT242_ALERT_2_C Low    'MainMol' Ueq as Compared to Neighbors of        C83 Check
PLAT250_ALERT_2_C Large U3/U1 Ratio for Average U(i,j) Tensor ....        3.4 Note
PLAT250_ALERT_2_C Large U3/U1 Ratio for Average U(i,j) Tensor ....        3.5 Note
PLAT338_ALERT_4_C Small Aver Tau in Cyclohexane C47      -C91A          31.06 Degree
PLAT911_ALERT_3_C Missing FCF Refl Between Thmin & STh/L=    0.600          6 Report
                0  5  0,  -4  9  2,  -5-13  4,  -6-12  4,  -6-12  5,  -7-11  7,
PLAT972_ALERT_2_C Check Calcd Resid. Dens.  0.74Ang From Rh1            -1.96 eA-3
PLAT972_ALERT_2_C Check Calcd Resid. Dens.  0.71Ang From Rh2            -1.93 eA-3
PLAT972_ALERT_2_C Check Calcd Resid. Dens.  0.78Ang From Rh2            -1.88 eA-3
PLAT972_ALERT_2_C Check Calcd Resid. Dens.  0.81Ang From Rh1            -1.83 eA-3
PLAT972_ALERT_2_C Check Calcd Resid. Dens.  0.74Ang From Rh1            -1.70 eA-3
PLAT972_ALERT_2_C Check Calcd Resid. Dens.  0.73Ang From Rh1            -1.69 eA-3
PLAT972_ALERT_2_C Check Calcd Resid. Dens.  0.77Ang From Rh2            -1.66 eA-3
PLAT972_ALERT_2_C Check Calcd Resid. Dens.  0.85Ang From Rh2            -1.56 eA-3
```

Alert level G

```
PLAT002_ALERT_2_G Number of Distance or Angle Restraints on AtSite          7 Note
PLAT003_ALERT_2_G Number of Uiso or Uij Restrained non-H Atoms ...          7 Report
PLAT154_ALERT_1_G The s.u.'s on the Cell Angles are Equal ..(Note)      0.001 Degree
PLAT172_ALERT_4_G The CIF-Embedded .res File Contains DFIX Records          3 Report
PLAT173_ALERT_4_G The CIF-Embedded .res File Contains DANG Records          5 Report
PLAT174_ALERT_4_G The CIF-Embedded .res File Contains FLAT Records          1 Report
PLAT176_ALERT_4_G The CIF-Embedded .res File Contains SADI Records          3 Report
PLAT178_ALERT_4_G The CIF-Embedded .res File Contains SIMU Records          1 Report
PLAT187_ALERT_4_G The CIF-Embedded .res File Contains RIGU Records          1 Report
PLAT191_ALERT_3_G A Non-default SADI Restraint Value has been used     0.0400 Report
PLAT191_ALERT_3_G A Non-default SADI Restraint Value has been used     0.0400 Report
PLAT230_ALERT_2_G Hirshfeld Test Diff for    C90      --C93A     .        6.6 s.u.
PLAT232_ALERT_2_G Hirshfeld Test Diff (M-X)  Rh1      --Cl1_a    .        9.8 s.u.
PLAT232_ALERT_2_G Hirshfeld Test Diff (M-X)  Rh1      --Cl2_a    .        6.8 s.u.
PLAT232_ALERT_2_G Hirshfeld Test Diff (M-X)  Rh2      --C53      .        5.9 s.u.
PLAT301_ALERT_3_G Main Residue  Disorder ..............(Resd  1  )         6% Note
PLAT302_ALERT_4_G Anion/Solvent/Minor-Residue Disorder (Resd  2  )       100% Note
PLAT302_ALERT_4_G Anion/Solvent/Minor-Residue Disorder (Resd  3  )       100% Note
PLAT304_ALERT_4_G Non-Integer Number of Atoms in ..... (Resd  2  )       7.95 Check
PLAT304_ALERT_4_G Non-Integer Number of Atoms in ..... (Resd  3  )       7.05 Check
PLAT343_ALERT_2_G Unusual sp?      Angle Range in Main Residue for        C51 Check
PLAT343_ALERT_2_G Unusual sp?      Angle Range in Main Residue for        C73 Check
PLAT371_ALERT_2_G Long   C(sp2)-C(sp1) Bond  C27      - C53      .       1.45 Ang.
PLAT371_ALERT_2_G Long   C(sp2)-C(sp1) Bond  C28      - C55      .       1.45 Ang.
PLAT410_ALERT_2_G Short Intra H...H Contact  H83B     ..H47A     .       2.08 Ang.
                                                      x,y,z  =      1_555 Check
PLAT720_ALERT_4_G Number of Unusual/Non-Standard Labels ..........          3 Note
              H2AA    H2AB    H2AC
PLAT790_ALERT_4_G Centre of Gravity not Within Unit Cell: Resd.  #          2 Note
```

```
             C7 H8
PLAT790_ALERT_4_G Centre of Gravity not Within Unit Cell: Resd.  #          3 Note
             C7 H8
PLAT860_ALERT_3_G Number of Least-Squares Restraints ............         136 Note
PLAT912_ALERT_4_G Missing # of FCF Reflections Above STh/L=  0.600        233 Note
PLAT941_ALERT_3_G Average HKL Measurement Multiplicity ..........         3.6 Low
PLAT978_ALERT_2_G Number C-C Bonds with Positive Residual Density.          2 Info
```

```
  0 ALERT level A = Most likely a serious problem - resolve or explain
  0 ALERT level B = A potentially serious problem, consider carefully
 17 ALERT level C = Check. Ensure it is not caused by an omission or oversight
 32 ALERT level G = General information/check it is not something unexpected

  1 ALERT type 1 CIF construction/syntax error, inconsistent or missing data
 26 ALERT type 2 Indicator that the structure model may be wrong or deficient
  6 ALERT type 3 Indicator that the structure quality may be low
 16 ALERT type 4 Improvement, methodology, query or suggestion
  0 ALERT type 5 Informative message, check
```

It is advisable to attempt to resolve as many as possible of the alerts in all categories. Often the minor alerts point to easily fixed oversights, errors and omissions in your CIF or refinement strategy, so attention to these fine details can be worthwhile. In order to resolve some of the more serious problems it may be necessary to carry out additional measurements or structure refinements. However, the purpose of your study may justify the reported deviations and the more serious of these should normally be commented upon in the discussion or experimental section of a paper or in the "special_details" fields of the CIF. checkCIF was carefully designed to identify outliers and unusual parameters, but every test has its limitations and alerts that are not important in a particular case may appear. Conversely, the absence of alerts does not guarantee there are no aspects of the results needing attention. It is up to the individual to critically assess their own results and, if necessary, seek expert advice.

Publication of your CIF in IUCr journals

A basic structural check has been run on your CIF. These basic checks will be run on all CIFs submitted for publication in IUCr journals (*Acta Crystallographica*, *Journal of Applied Crystallography*, *Journal of Synchrotron Radiation*); however, if you intend to submit to *Acta Crystallographica Section C* or *E* or *IUCrData*, you should make sure that full publication checks are run on the final version of your CIF prior to submission.

Publication of your CIF in other journals

Please refer to the *Notes for Authors* of the relevant journal for any special instructions relating to CIF submission.

PLATON version of 29/11/2023; check.def file version of 14/09/2023

Datablock cs2_00rh2cl2_auto - ellipsoid plot

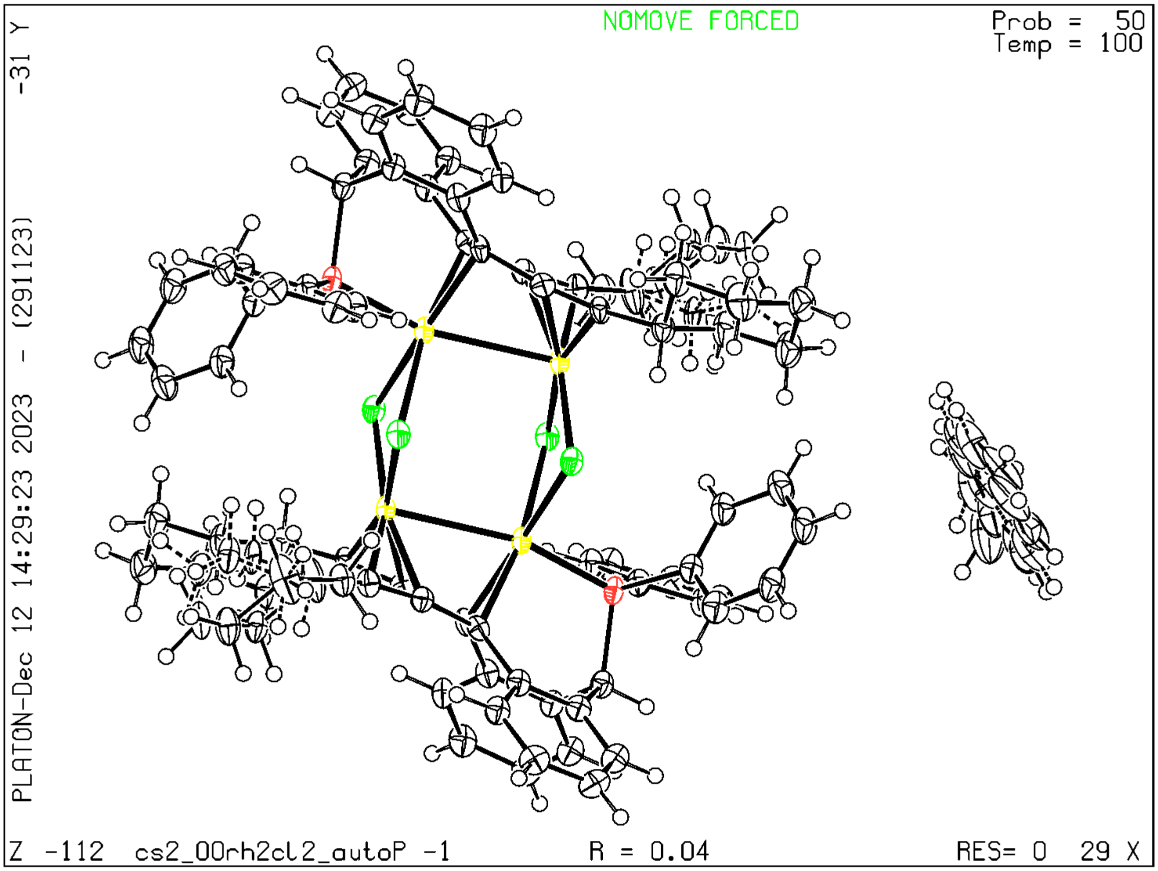

Supplement: Supplementary file 2 — Supporting Information [file ADVS-11-2400072-s001.zip › [2]_Rh2Cl2_2313435_cifreport.pdf]
