# Supplementary material for: Structural Changes in the Carbon Sphere of a Dirhodium Complex Induced by Redox or Deprotonation Reactions
Source: Adv Sci (Weinh). 2024 Mar 23;11(22):2400072. doi: 10.1002/advs.202400072 (PMC11165463; doi:10.1002/advs.202400072)

checkCIF/PLATON report

Structure factors have been supplied for datablock(s) cs3_142_rh2otf2_auto

THIS REPORT IS FOR GUIDANCE ONLY. IF USED AS PART OF A REVIEW PROCEDURE FOR PUBLICATION, IT SHOULD NOT REPLACE THE EXPERTISE OF AN EXPERIENCED CRYSTALLOGRAPHIC REFEREE.

No syntax errors found. CIF dictionary Interpreting this report

Datablock: cs3_142_rh2otf2_auto

Bond precision: C-C = 0.0060 A Wavelength=1.54184

Cell:	a=10.3184(3)	b=10.6661(3)	c=20.2792(6)
	alpha=100.752(3)	beta=99.799(3)	gamma=100.050(3)
Temperature:	100 K		

	Calculated	Reported
Volume	2111.09(11)	2111.09(11)
Space group	P -1	P -1
Hall group	-P 1	-P 1
Moiety formula	C45 H41 F6 O6 P Rh2 S2	C45 H41 F6 O6 P Rh2 S2
Sum formula	C45 H41 F6 O6 P Rh2 S2	C45 H41 F6 O6 P Rh2 S2
Mr	1092.69	1092.69
Dx,g cm-3	1.719	1.719
Z	2	2
Mu (mm-1)	8.271	8.271
F000	1100.0	1100.0
F000'	1105.45	
h,k,lmax	10,10,20	10,10,20
Nref	4717	4623
Tmin,Tmax	0.646,0.695	0.622,1.000
Tmin'	0.495	

Correction method= # Reported T Limits: Tmin=0.622 Tmax=1.000
AbsCorr = MULTI-SCAN

Data completeness= 0.980 Theta(max)= 51.928

R(reflections)= 0.0278(4170) wR2(reflections)= 0.0676(4623)

S = 1.030 Npar= 559

The following ALERTS were generated. Each ALERT has the format
test-name_ALERT_alert-type_alert-level.
Click on the hyperlinks for more details of the test.

Alert level A

```
THETM01_ALERT_3_A  The value of sine(theta_max)/wavelength is less than 0.550
            Calculated sin(theta_max)/wavelength =    0.5106
```

Alert level C

```
PLAT088_ALERT_3_C Poor Data / Parameter Ratio ....................       8.27 Note
PLAT911_ALERT_3_C Missing FCF Refl Between Thmin & STh/L=    0.511         93 Report
              -10  5  0,   7  6  0,   5  8  0,  -8 -5  1,  -1  0  1,  10  0  1,
              -10  5  1,   6  7  1,   1 10  1,  -4 -9  2,   9 -7  2,  -8 -5  2,
               10 -4  2,   3  9  2,  10 -3  3,   8  4  3,  -4 -9  4, -10  5  4,
               -8  8  4,   2  9  4,  -4 -9  5,   9 -6  5,   9  1  5,   9  0  6,
                9 -4  7,   9 -1  7, -10  4  7,  -9  6  7,  -7  8  7,   0  9  7,
                6 -9  8,   7 -8  8, -10 -1  8,  -8  7  8,   4-10  9,  -4 -9  9,
                8 -6  9,  -9  5  9,  -6  8  9,  -1-10 10,   3-10 10,  -8 -5 10,
                8 -1 10,  -8  6 10,  -7  7 10,  -5  8 10,   0-10 11,   1-10 11,
                2-10 11,  -5 -8 11,   6 -8 11,  -7 -6 11,  -9  4 11,  -3 -9 12,
                4 -9 12,   6  2 12,  -8  5 12,  -7  6 12,   2  6 12,  -2 -9 13,
               -8 -4 13,  -9 -1 13,  -9  2 13,   1  6 13,  -1 -9 14,  -3 -8 15,
               -7 -4 15,  -2 -8 16,   2 -8 16,  -4 -7 16,  -6 -5 16,  -6 -4 16,
               -7 -3 16,   4  1 16,  -7  3 16,  -6  4 16,   4 -5 17,  -6 -4 17,
               -1 -7 18,   0 -7 18,   3 -5 18,   3 -4 18,   2  1 18,  -1 -6 19,
                0 -6 19,   2 -1 19,   1  0 19,  -4  2 19,  -3  2 19,  -1  2 19,
               -3 -4 20,   0 -1 20,  -1  0 20,
```

Alert level G

```
PLAT154_ALERT_1_G The s.u.'s on the Cell Angles are Equal ..(Note)      0.003 Degree
PLAT343_ALERT_2_G Unusual sp?      Angle Range in Main Residue for         C4 Check
PLAT343_ALERT_2_G Unusual sp?      Angle Range in Main Residue for         C7 Check
PLAT371_ALERT_2_G Long   C(sp2)-C(sp1) Bond  C1       - C6       .       1.46 Ang.
PLAT371_ALERT_2_G Long   C(sp2)-C(sp1) Bond  C2       - C3       .       1.45 Ang.
PLAT909_ALERT_3_G Percentage of I>2sig(I) Data at Theta(Max) Still        83% Note
PLAT910_ALERT_3_G Missing # of FCF Reflection(s) Below Theta(Min).          1 Note
                0  0  1,
PLAT941_ALERT_3_G Average HKL Measurement Multiplicity ...........        3.1 Low
PLAT978_ALERT_2_G Number C-C Bonds with Positive Residual Density.          0 Info
```

1 **ALERT level A** = Most likely a serious problem - resolve or explain
0 **ALERT level B** = A potentially serious problem, consider carefully
2 **ALERT level C** = Check. Ensure it is not caused by an omission or oversight
9 **ALERT level G** = General information/check it is not something unexpected

1 ALERT type 1 CIF construction/syntax error, inconsistent or missing data
5 ALERT type 2 Indicator that the structure model may be wrong or deficient
6 ALERT type 3 Indicator that the structure quality may be low
0 ALERT type 4 Improvement, methodology, query or suggestion
0 ALERT type 5 Informative message, check

It is advisable to attempt to resolve as many as possible of the alerts in all categories. Often the minor alerts point to easily fixed oversights, errors and omissions in your CIF or refinement strategy, so attention to these fine details can be worthwhile. In order to resolve some of the more serious problems it may be necessary to carry out additional measurements or structure refinements. However, the purpose of your study may justify the reported deviations and the more serious of these should normally be commented upon in the discussion or experimental section of a paper or in the "special_details" fields of the CIF. checkCIF was carefully designed to identify outliers and unusual parameters, but every test has its limitations and alerts that are not important in a particular case may appear. Conversely, the absence of alerts does not guarantee there are no aspects of the results needing attention. It is up to the individual to critically assess their own results and, if necessary, seek expert advice.

Publication of your CIF in IUCr journals

A basic structural check has been run on your CIF. These basic checks will be run on all CIFs submitted for publication in IUCr journals (*Acta Crystallographica*, *Journal of Applied Crystallography*, *Journal of Synchrotron Radiation*); however, if you intend to submit to *Acta Crystallographica Section C* or *E* or *IUCrData*, you should make sure that full publication checks are run on the final version of your CIF prior to submission.

Publication of your CIF in other journals

Please refer to the *Notes for Authors* of the relevant journal for any special instructions relating to CIF submission.

Validation response form

Please find below a validation response form (VRF) that can be filled in and pasted into your CIF.

```
# start Validation Reply Form
_vrf_THETM01_cs3_142_rh2otf2_auto
;
PROBLEM: The value of sine(theta_max)/wavelength is less than 0.550
RESPONSE: ...
;
# end Validation Reply Form
```

PLATON version of 29/11/2023; check.def file version of 14/09/2023

Datablock cs3_142_rh2otf2_auto - ellipsoid plot

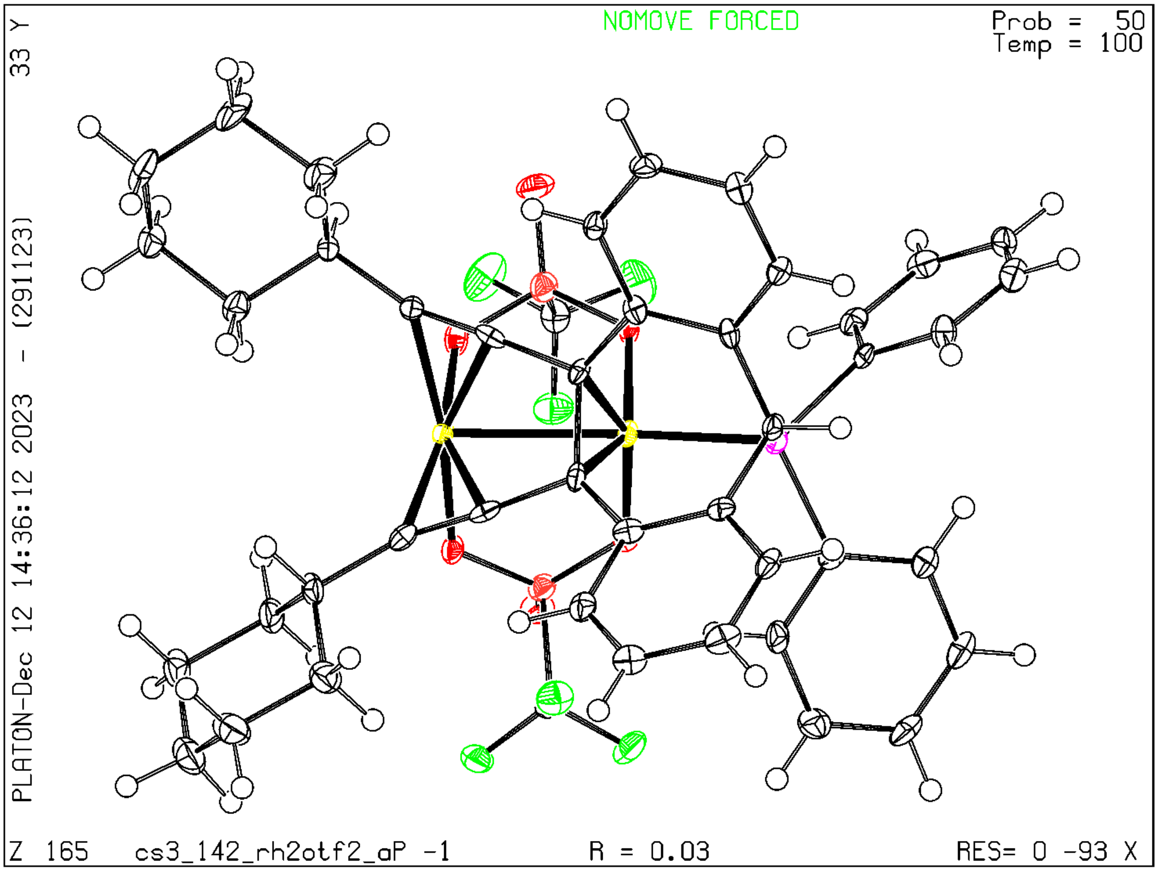

Supplement: Supplementary file 2 — Supporting Information [file ADVS-11-2400072-s001.zip › [3]_Rh2OTf2_2313858_cifreport.pdf]
