# Supplementary material for: Structural Changes in the Carbon Sphere of a Dirhodium Complex Induced by Redox or Deprotonation Reactions
Source: Adv Sci (Weinh). 2024 Mar 23;11(22):2400072. doi: 10.1002/advs.202400072 (PMC11165463; doi:10.1002/advs.202400072)

checkCIF/PLATON report

Structure factors have been supplied for datablock(s) cs2_065

THIS REPORT IS FOR GUIDANCE ONLY. IF USED AS PART OF A REVIEW PROCEDURE FOR PUBLICATION, IT SHOULD NOT REPLACE THE EXPERTISE OF AN EXPERIENCED CRYSTALLOGRAPHIC REFEREE.

No syntax errors found.   CIF dictionary   Interpreting this report

Datablock: cs2_065

Bond precision: C-C = 0.0089 A   Wavelength=0.71073

Cell:	a=13.8174(4)	b=16.2833(5)	c=17.0904(5)
	alpha=110.982(3)	beta=105.836(3)	gamma=96.016(2)
Temperature:	100 K		

	Calculated	Reported
Volume	3365.1(2)	3365.06(19)
Space group	P -1	P -1
Hall group	-P 1	-P 1
Moiety formula	C63 H57 N4 P Rh2, 2(C F3 O3 S), 2(C4 H8 O)	C63 H57 N4 P Rh2, 2(C F3 O3 S), 2(C4 H8 O)
Sum formula	C73 H73 F6 N4 O8 P Rh2 S2	C73 H73 F6 N4 O8 P Rh2 S2
Mr	1549.27	1549.26
Dx,g cm-3	1.529	1.529
Z	2	2
Mu (mm-1)	0.653	0.653
F000	1588.0	1588.0
F000’	1584.95	
h,k,lmax	17,20,21	17,20,21
Nref	13779	13727
Tmin,Tmax	0.947,0.984	0.986,1.000
Tmin’	0.913	

Correction method= # Reported T Limits: Tmin=0.986 Tmax=1.000
AbsCorr = MULTI-SCAN

Data completeness= 0.996   Theta(max)= 26.372

R(reflections)= 0.0563(10589)   wR2(reflections)= 0.1333(13727)

S = 1.041   Npar= 943

The following ALERTS were generated. Each ALERT has the format
test-name_ALERT_alert-type_alert-level.
Click on the hyperlinks for more details of the test.

Alert level C

```
PLAT213_ALERT_2_C Atom C21             has ADP max/min Ratio .....        3.5 prolat
PLAT213_ALERT_2_C Atom C22             has ADP max/min Ratio .....        3.1 prolat
PLAT220_ALERT_2_C NonSolvent   Resd 1  C   Ueq(max)/Ueq(min) Range        5.5 Ratio
PLAT222_ALERT_3_C NonSolvent Resd 1  H   Uiso(max)/Uiso(min) Range        4.3 Ratio
PLAT234_ALERT_4_C Large Hirshfeld Difference S2       --O5       .       0.16 Ang.
PLAT234_ALERT_4_C Large Hirshfeld Difference S2       --C73      .       0.18 Ang.
PLAT234_ALERT_4_C Large Hirshfeld Difference F3A      --C73      .       0.18 Ang.
PLAT234_ALERT_4_C Large Hirshfeld Difference F3       --C73      .       0.18 Ang.
PLAT241_ALERT_2_C High   'MainMol' Ueq as Compared to Neighbors of        C46 Check
PLAT243_ALERT_4_C High   'Solvent' Ueq as Compared to Neighbors of        C69 Check
PLAT244_ALERT_4_C Low    'Solvent' Ueq as Compared to Neighbors of         S2 Check
PLAT244_ALERT_4_C Low    'Solvent' Ueq as Compared to Neighbors of         O8 Check
PLAT244_ALERT_4_C Low    'Solvent' Ueq as Compared to Neighbors of        C70 Check
PLAT260_ALERT_2_C Large Average Ueq of Residue Including        S2      0.133 Check
PLAT260_ALERT_2_C Large Average Ueq of Residue Including        O8      0.167 Check
PLAT260_ALERT_2_C Large Average Ueq of Residue Including       O2A      0.104 Check
PLAT342_ALERT_3_C Low Bond Precision on  C-C Bonds ..............    0.00888 Ang.
PLAT906_ALERT_3_C Large K Value in the Analysis of Variance ......      6.595 Check
PLAT910_ALERT_3_C Missing # of FCF Reflection(s) Below Theta(Min).          6 Note
               1  0  0,  -1  1  0,   0  1  0,   0 -1  1,  -1  0  1,   0  0  1,
```

Alert level G

```
PLAT002_ALERT_2_G Number of Distance or Angle Restraints on AtSite         19 Note
PLAT083_ALERT_2_G SHELXL Second Parameter in WGHT  Unusually Large       6.93 Why ?
PLAT171_ALERT_4_G The CIF-Embedded .res File Contains EADP Records          2 Report
PLAT176_ALERT_4_G The CIF-Embedded .res File Contains SADI Records          7 Report
PLAT187_ALERT_4_G The CIF-Embedded .res File Contains RIGU Records          5 Report
PLAT231_ALERT_4_G Hirshfeld Test (Solvent)   S2       --O15      .        8.9 s.u.
PLAT231_ALERT_4_G Hirshfeld Test (Solvent)   F2A      --C73      .        5.1 s.u.
PLAT231_ALERT_4_G Hirshfeld Test (Solvent)   F5A      --C73      .        6.5 s.u.
PLAT244_ALERT_4_G Low    'Solvent' Ueq as Compared to Neighbors of        C73 Check
PLAT244_ALERT_4_G Low    'Solvent' Ueq as Compared to Neighbors of        C72 Check
PLAT302_ALERT_4_G Anion/Solvent/Minor-Residue Disorder (Resd  2  )        75% Note
PLAT302_ALERT_4_G Anion/Solvent/Minor-Residue Disorder (Resd  4  )       100% Note
PLAT302_ALERT_4_G Anion/Solvent/Minor-Residue Disorder (Resd  6  )       100% Note
PLAT304_ALERT_4_G Non-Integer Number of Atoms in ..... (Resd  4  )       8.02 Check
PLAT304_ALERT_4_G Non-Integer Number of Atoms in ..... (Resd  6  )       4.98 Check
PLAT343_ALERT_2_G Unusual sp?      Angle Range in Main Residue for        C17 Check
PLAT343_ALERT_2_G Unusual sp?      Angle Range in Main Residue for        C25 Check
PLAT371_ALERT_2_G Long   C(sp2)-C(sp1) Bond  C8       - C16      .       1.45 Ang.
PLAT371_ALERT_2_G Long   C(sp2)-C(sp1) Bond  C9       - C24      .       1.44 Ang.
PLAT398_ALERT_2_G Deviating  C-O-C   Angle From 120 for O2       .      102.5 Degree
PLAT398_ALERT_2_G Deviating  C-O-C   Angle From 120 for O8       .      106.0 Degree
PLAT432_ALERT_2_G Short Inter X...Y Contact  O3A      ..C55      .       2.91 Ang.
                                              -x,1-y,1-z  =      2_566 Check
PLAT432_ALERT_2_G Short Inter X...Y Contact  O15      ..C53      .       2.78 Ang.
                                             1-x,1-y,1-z  =      2_666 Check
PLAT432_ALERT_2_G Short Inter X...Y Contact  O15      ..C52      .       2.95 Ang.
                                             1-x,1-y,1-z  =      2_666 Check
```

```
PLAT790_ALERT_4_G Centre of Gravity not Within Unit Cell: Resd.  #          5 Note
              C4 H8 O
PLAT860_ALERT_3_G Number of Least-Squares Restraints ............         186 Note
PLAT912_ALERT_4_G Missing # of FCF Reflections Above STh/L=  0.600         46 Note
PLAT933_ALERT_2_G Number of HKL-OMIT Records in Embedded .res File          1 Note
               -1  1  0,
PLAT978_ALERT_2_G Number C-C Bonds with Positive Residual Density.          0 Info
```

```
   0 ALERT level A = Most likely a serious problem - resolve or explain
   0 ALERT level B = A potentially serious problem, consider carefully
  19 ALERT level C = Check. Ensure it is not caused by an omission or oversight
  29 ALERT level G = General information/check it is not something unexpected

   0 ALERT type 1 CIF construction/syntax error, inconsistent or missing data
  20 ALERT type 2 Indicator that the structure model may be wrong or deficient
   5 ALERT type 3 Indicator that the structure quality may be low
  23 ALERT type 4 Improvement, methodology, query or suggestion
   0 ALERT type 5 Informative message, check
```

It is advisable to attempt to resolve as many as possible of the alerts in all categories. Often the minor alerts point to easily fixed oversights, errors and omissions in your CIF or refinement strategy, so attention to these fine details can be worthwhile. In order to resolve some of the more serious problems it may be necessary to carry out additional measurements or structure refinements. However, the purpose of your study may justify the reported deviations and the more serious of these should normally be commented upon in the discussion or experimental section of a paper or in the "special_details" fields of the CIF. checkCIF was carefully designed to identify outliers and unusual parameters, but every test has its limitations and alerts that are not important in a particular case may appear. Conversely, the absence of alerts does not guarantee there are no aspects of the results needing attention. It is up to the individual to critically assess their own results and, if necessary, seek expert advice.

Publication of your CIF in IUCr journals

A basic structural check has been run on your CIF. These basic checks will be run on all CIFs submitted for publication in IUCr journals (*Acta Crystallographica*, *Journal of Applied Crystallography*, *Journal of Synchrotron Radiation*); however, if you intend to submit to *Acta Crystallographica Section C* or *E* or *IUCrData*, you should make sure that full publication checks are run on the final version of your CIF prior to submission.

Publication of your CIF in other journals

Please refer to the *Notes for Authors* of the relevant journal for any special instructions relating to CIF submission.

PLATON version of 29/11/2023; check.def file version of 14/09/2023

Datablock cs2_065 - ellipsoid plot

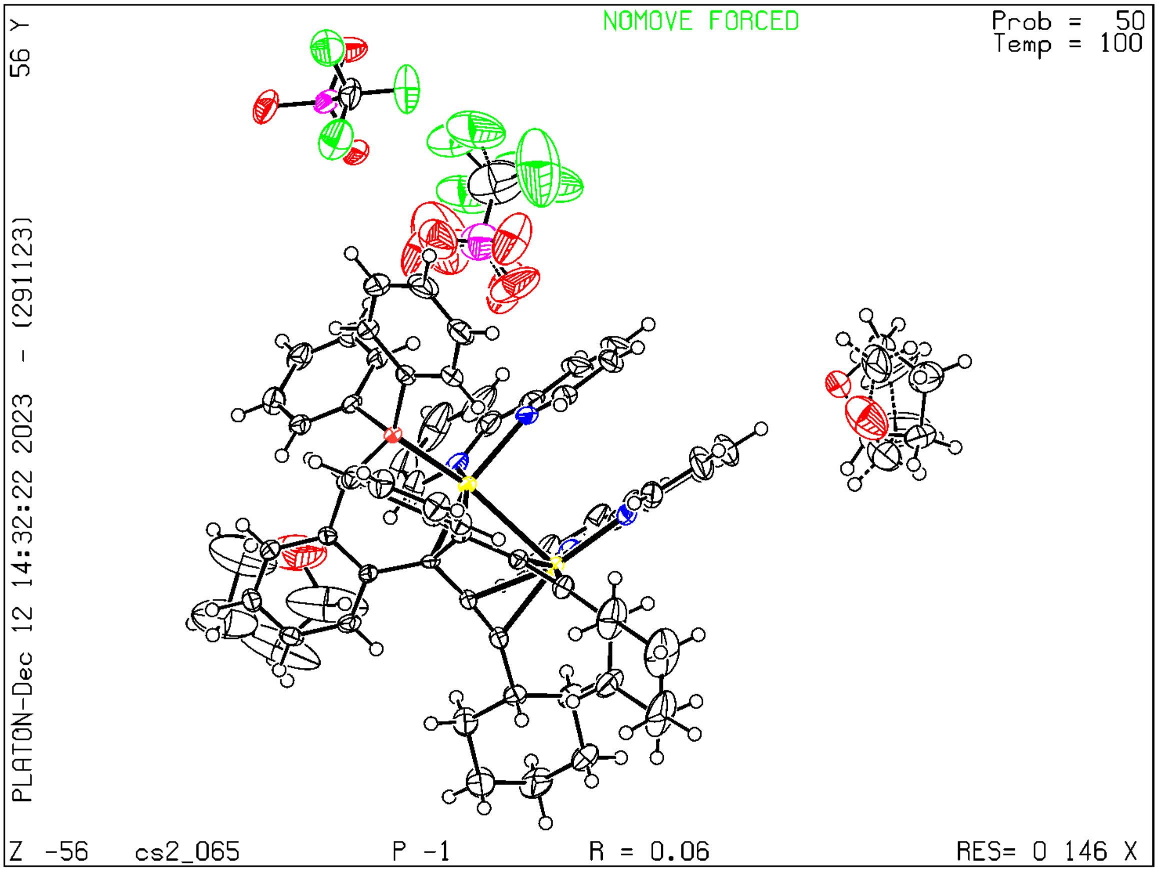

Supplement: Supplementary file 2 — Supporting Information [file ADVS-11-2400072-s001.zip › [4](OTf)2_Rh2bipy2_2313437_cifreport.pdf]
