# Supplementary material for: Structural Changes in the Carbon Sphere of a Dirhodium Complex Induced by Redox or Deprotonation Reactions
Source: Adv Sci (Weinh). 2024 Mar 23;11(22):2400072. doi: 10.1002/advs.202400072 (PMC11165463; doi:10.1002/advs.202400072)

checkCIF/PLATON report

Structure factors have been supplied for datablock(s) cs3_099_auto

THIS REPORT IS FOR GUIDANCE ONLY. IF USED AS PART OF A REVIEW PROCEDURE FOR PUBLICATION, IT SHOULD NOT REPLACE THE EXPERTISE OF AN EXPERIENCED CRYSTALLOGRAPHIC REFEREE.

No syntax errors found. CIF dictionary Interpreting this report

Datablock: cs3_099_auto

Bond precision:	C-C = 0.0096 A		Wavelength=1.54184
Cell:	a=9.9352(1)	b=43.1896(5)	c=13.4518(1)
	alpha=90	beta=98.428(1)	gamma=90
Temperature:	100 K		

	Calculated	Reported
Volume	5709.80(10)	5709.80(10)
Space group	P 21/n	P 1 21/n 1
Hall group	-P 2yn	-P 2yn
Moiety formula	2(C63.08 H57.17 N4 P Rh2), 2(C2.50 H2.50 N0.50), 2(C F3 O3 S)	C65.583 H59.667 N4.5 P Rh2, C F3 O3 S
Sum formula	C133.17 H119.33 F6 N9 O6 P2 Rh4 S2	C66.58 H59.67 F3 N4.50 O3 P Rh2 S
Mr	2593.41	1296.69
Dx,g cm-3	1.508	1.508
Z	2	4
Mu (mm-1)	5.791	5.791
F000	2650.7	2651.0
F000'	2660.65	
h,k,lmax	12,55,17	12,54,16
Nref	12516	12189
Tmin,Tmax	0.757,0.802	0.789,1.000
Tmin'	0.590	

Correction method= # Reported T Limits: Tmin=0.789 Tmax=1.000
AbsCorr = MULTI-SCAN

Data completeness= 0.974 Theta(max)= 80.409

```
R(reflections)= 0.0642( 11048)                         wR2(reflections)=
                                                       0.1835( 12189)
S = 1.076                  Npar= 745
```

The following ALERTS were generated. Each ALERT has the format
test-name_ALERT_alert-type_alert-level.
Click on the hyperlinks for more details of the test.

Alert level C

```
PLAT042_ALERT_1_C Calc. and Reported MoietyFormula Strings  Differ     Please Check
              Calc: 2(C63.08 H57.17 N4 P Rh2), 2(C2.50 H2.50 N0.50), 2(C F3 O3 S
              Rep.: C65.583 H59.667 N4.5 P Rh2, C F3 O3 S
PLAT220_ALERT_2_C NonSolvent   Resd 1  C   Ueq(max)/Ueq(min) Range        5.7 Ratio
PLAT222_ALERT_3_C NonSolvent Resd 1  H   Uiso(max)/Uiso(min) Range        5.4 Ratio
PLAT234_ALERT_4_C Large Hirshfeld Difference C5       --C24      .       0.19 Ang.
PLAT244_ALERT_4_C Low    'Solvent' Ueq as Compared to Neighbors of         S1 Check
PLAT250_ALERT_2_C Large U3/U1 Ratio for Average U(i,j) Tensor ....        2.2 Note
PLAT342_ALERT_3_C Low Bond Precision on  C-C Bonds ..............    0.00958 Ang.
PLAT906_ALERT_3_C Large K Value in the Analysis of Variance ......      3.612 Check
PLAT911_ALERT_3_C Missing FCF Refl Between Thmin & STh/L=    0.600         21 Report
               -1  6  1,  -2  8  1,   1 23  4,   9 23  4,   1 24  4,  -1 13  5,
               -1 23  5,  -1 24  5,   2 40  7,   2  0  8,  -8 20 11,  -8 21 11,
                4 24 12,   5  0 13,   4  1 13,   5  1 13,   4  2 13,   5  2 13,
                4  3 13,   4  4 13,  -3  1 16,
PLAT918_ALERT_3_C Reflection(s) with I(obs) much Smaller I(calc) .          1 Check
PLAT971_ALERT_2_C Check Calcd Resid. Dens.  1.28Ang From C19             1.61 eA-3
PLAT976_ALERT_2_C Check Calcd Resid. Dens.  0.84Ang From O2      .      -0.55 eA-3
PLAT977_ALERT_2_C Check Negative Difference Density on H8        .      -0.54 eA-3
PLAT977_ALERT_2_C Check Negative Difference Density on H17B      .      -0.35 eA-3
PLAT977_ALERT_2_C Check Negative Difference Density on H19B      .      -0.53 eA-3
PLAT977_ALERT_2_C Check Negative Difference Density on H21A      .      -0.47 eA-3
PLAT977_ALERT_2_C Check Negative Difference Density on H22B      .      -0.31 eA-3
```

Alert level G

```
PLAT002_ALERT_2_G Number of Distance or Angle Restraints on AtSite         17 Note
PLAT003_ALERT_2_G Number of Uiso or Uij Restrained non-H Atoms ...         17 Report
PLAT045_ALERT_1_G Calculated and Reported Z Differ by a Factor ...      0.500 Check
PLAT068_ALERT_1_G Reported F000 Differs from Calcd (or Missing)...     Please Check
PLAT083_ALERT_2_G SHELXL Second Parameter in WGHT  Unusually Large      20.39 Why ?
PLAT143_ALERT_4_G s.u. on c - Axis Small or Missing .............    0.00010 Ang.
PLAT171_ALERT_4_G The CIF-Embedded .res File Contains EADP Records          4 Report
PLAT172_ALERT_4_G The CIF-Embedded .res File Contains DFIX Records          1 Report
PLAT173_ALERT_4_G The CIF-Embedded .res File Contains DANG Records          1 Report
PLAT176_ALERT_4_G The CIF-Embedded .res File Contains SADI Records          5 Report
PLAT178_ALERT_4_G The CIF-Embedded .res File Contains SIMU Records          3 Report
PLAT187_ALERT_4_G The CIF-Embedded .res File Contains RIGU Records          1 Report
PLAT191_ALERT_3_G A Non-default SADI Restraint Value has been used     0.0400 Report
PLAT232_ALERT_2_G Hirshfeld Test Diff (M-X)  Rh2      --C7       .        7.6 s.u.
PLAT244_ALERT_4_G Low    'Solvent' Ueq as Compared to Neighbors of        C68 Check
PLAT300_ALERT_4_G Atom Site Occupancy of C14        Constrained at       0.75 Check
PLAT300_ALERT_4_G Atom Site Occupancy of C15        Constrained at       0.75 Check
PLAT300_ALERT_4_G Atom Site Occupancy of C16        Constrained at       0.75 Check
PLAT300_ALERT_4_G Atom Site Occupancy of C17        Constrained at       0.75 Check
```

```
PLAT300_ALERT_4_G Atom Site Occupancy of C18        Constrained at       0.75 Check
PLAT300_ALERT_4_G Atom Site Occupancy of C24        Constrained at       0.25 Check
PLAT300_ALERT_4_G Atom Site Occupancy of C25        Constrained at       0.25 Check
PLAT300_ALERT_4_G Atom Site Occupancy of C26        Constrained at       0.25 Check
PLAT300_ALERT_4_G Atom Site Occupancy of C27        Constrained at     0.3333 Check
PLAT300_ALERT_4_G Atom Site Occupancy of C28        Constrained at       0.25 Check
PLAT300_ALERT_4_G Atom Site Occupancy of H5A        Constrained at       0.75 Check
PLAT300_ALERT_4_G Atom Site Occupancy of H14A       Constrained at       0.75 Check
PLAT300_ALERT_4_G Atom Site Occupancy of H14B       Constrained at       0.75 Check
PLAT300_ALERT_4_G Atom Site Occupancy of H15A       Constrained at       0.75 Check
PLAT300_ALERT_4_G Atom Site Occupancy of H15B       Constrained at       0.75 Check
PLAT300_ALERT_4_G Atom Site Occupancy of H16A       Constrained at       0.75 Check
PLAT300_ALERT_4_G Atom Site Occupancy of H16B       Constrained at       0.75 Check
PLAT300_ALERT_4_G Atom Site Occupancy of H17A       Constrained at       0.75 Check
PLAT300_ALERT_4_G Atom Site Occupancy of H17B       Constrained at       0.75 Check
PLAT300_ALERT_4_G Atom Site Occupancy of H18A       Constrained at       0.75 Check
PLAT300_ALERT_4_G Atom Site Occupancy of H18B       Constrained at       0.75 Check
PLAT300_ALERT_4_G Atom Site Occupancy of H5B        Constrained at       0.25 Check
PLAT300_ALERT_4_G Atom Site Occupancy of H24A       Constrained at       0.25 Check
PLAT300_ALERT_4_G Atom Site Occupancy of H24B       Constrained at       0.25 Check
PLAT300_ALERT_4_G Atom Site Occupancy of H25A       Constrained at       0.25 Check
PLAT300_ALERT_4_G Atom Site Occupancy of H25B       Constrained at       0.25 Check
PLAT300_ALERT_4_G Atom Site Occupancy of H26A       Constrained at       0.25 Check
PLAT300_ALERT_4_G Atom Site Occupancy of H26B       Constrained at       0.25 Check
PLAT300_ALERT_4_G Atom Site Occupancy of H27A       Constrained at     0.3333 Check
PLAT300_ALERT_4_G Atom Site Occupancy of H27B       Constrained at     0.3333 Check
PLAT300_ALERT_4_G Atom Site Occupancy of H28A       Constrained at       0.25 Check
PLAT300_ALERT_4_G Atom Site Occupancy of H28B       Constrained at       0.25 Check
PLAT300_ALERT_4_G Atom Site Occupancy of C70        Constrained at        0.5 Check
PLAT300_ALERT_4_G Atom Site Occupancy of C72        Constrained at        0.5 Check
PLAT300_ALERT_4_G Atom Site Occupancy of C73        Constrained at        0.5 Check
PLAT300_ALERT_4_G Atom Site Occupancy of N5         Constrained at       0.25 Check
PLAT300_ALERT_4_G Atom Site Occupancy of N5A        Constrained at       0.25 Check
PLAT300_ALERT_4_G Atom Site Occupancy of C11A       Constrained at       0.25 Check
PLAT300_ALERT_4_G Atom Site Occupancy of C71        Constrained at       0.25 Check
PLAT300_ALERT_4_G Atom Site Occupancy of C74        Constrained at       0.25 Check
PLAT300_ALERT_4_G Atom Site Occupancy of C75        Constrained at       0.25 Check
PLAT300_ALERT_4_G Atom Site Occupancy of H73        Constrained at        0.5 Check
PLAT300_ALERT_4_G Atom Site Occupancy of H11A       Constrained at       0.25 Check
PLAT300_ALERT_4_G Atom Site Occupancy of H70        Constrained at       0.25 Check
PLAT300_ALERT_4_G Atom Site Occupancy of H70A       Constrained at       0.25 Check
PLAT300_ALERT_4_G Atom Site Occupancy of H71        Constrained at       0.25 Check
PLAT300_ALERT_4_G Atom Site Occupancy of H72        Constrained at       0.25 Check
PLAT300_ALERT_4_G Atom Site Occupancy of H72A       Constrained at       0.25 Check
PLAT300_ALERT_4_G Atom Site Occupancy of H74        Constrained at       0.25 Check
PLAT300_ALERT_4_G Atom Site Occupancy of H75        Constrained at       0.25 Check
PLAT301_ALERT_3_G Main Residue  Disorder .............(Resd  1  )          7% Note
PLAT302_ALERT_4_G Anion/Solvent/Minor-Residue Disorder (Resd  2  )       100% Note
PLAT304_ALERT_4_G Non-Integer Number of Atoms in ..... (Resd  1  )     127.25 Check
PLAT304_ALERT_4_G Non-Integer Number of Atoms in ..... (Resd  2  )       5.50 Check
PLAT371_ALERT_2_G Long   C(sp2)-C(sp1) Bond  C1       - C6       .       1.46 Ang.
PLAT371_ALERT_2_G Long   C(sp2)-C(sp1) Bond  C2       - C3       .       1.45 Ang.
PLAT411_ALERT_2_G Short Inter H...H Contact  H59      ..H24B     .       2.13 Ang.
                                                    -1+x,y,z  =      1_455 Check
PLAT432_ALERT_2_G Short Inter X...Y Contact  C59      ..C24      .       3.12 Ang.
                                                    -1+x,y,z  =      1_455 Check
PLAT860_ALERT_3_G Number of Least-Squares Restraints ............        215 Note
```

```
PLAT912_ALERT_4_G Missing # of FCF Reflections Above STh/L=  0.600        277 Note
PLAT941_ALERT_3_G Average HKL Measurement Multiplicity ..........         4.4 Low
PLAT978_ALERT_2_G Number C-C Bonds with Positive Residual Density.          2 Info
```

```
   0 ALERT level A = Most likely a serious problem - resolve or explain
   0 ALERT level B = A potentially serious problem, consider carefully
  17 ALERT level C = Check. Ensure it is not caused by an omission or oversight
  77 ALERT level G = General information/check it is not something unexpected

   3 ALERT type 1 CIF construction/syntax error, inconsistent or missing data
  18 ALERT type 2 Indicator that the structure model may be wrong or deficient
   9 ALERT type 3 Indicator that the structure quality may be low
  64 ALERT type 4 Improvement, methodology, query or suggestion
   0 ALERT type 5 Informative message, check
```

It is advisable to attempt to resolve as many as possible of the alerts in all categories. Often the minor alerts point to easily fixed oversights, errors and omissions in your CIF or refinement strategy, so attention to these fine details can be worthwhile. In order to resolve some of the more serious problems it may be necessary to carry out additional measurements or structure refinements. However, the purpose of your study may justify the reported deviations and the more serious of these should normally be commented upon in the discussion or experimental section of a paper or in the "special_details" fields of the CIF. checkCIF was carefully designed to identify outliers and unusual parameters, but every test has its limitations and alerts that are not important in a particular case may appear. Conversely, the absence of alerts does not guarantee there are no aspects of the results needing attention. It is up to the individual to critically assess their own results and, if necessary, seek expert advice.

Publication of your CIF in IUCr journals

A basic structural check has been run on your CIF. These basic checks will be run on all CIFs submitted for publication in IUCr journals (*Acta Crystallographica*, *Journal of Applied Crystallography*, *Journal of Synchrotron Radiation*); however, if you intend to submit to *Acta Crystallographica Section C* or *E* or *IUCrData*, you should make sure that full publication checks are run on the final version of your CIF prior to submission.

Publication of your CIF in other journals

Please refer to the *Notes for Authors* of the relevant journal for any special instructions relating to CIF submission.

PLATON version of 29/11/2023; check.def file version of 14/09/2023

Datablock cs3_099_auto - ellipsoid plot

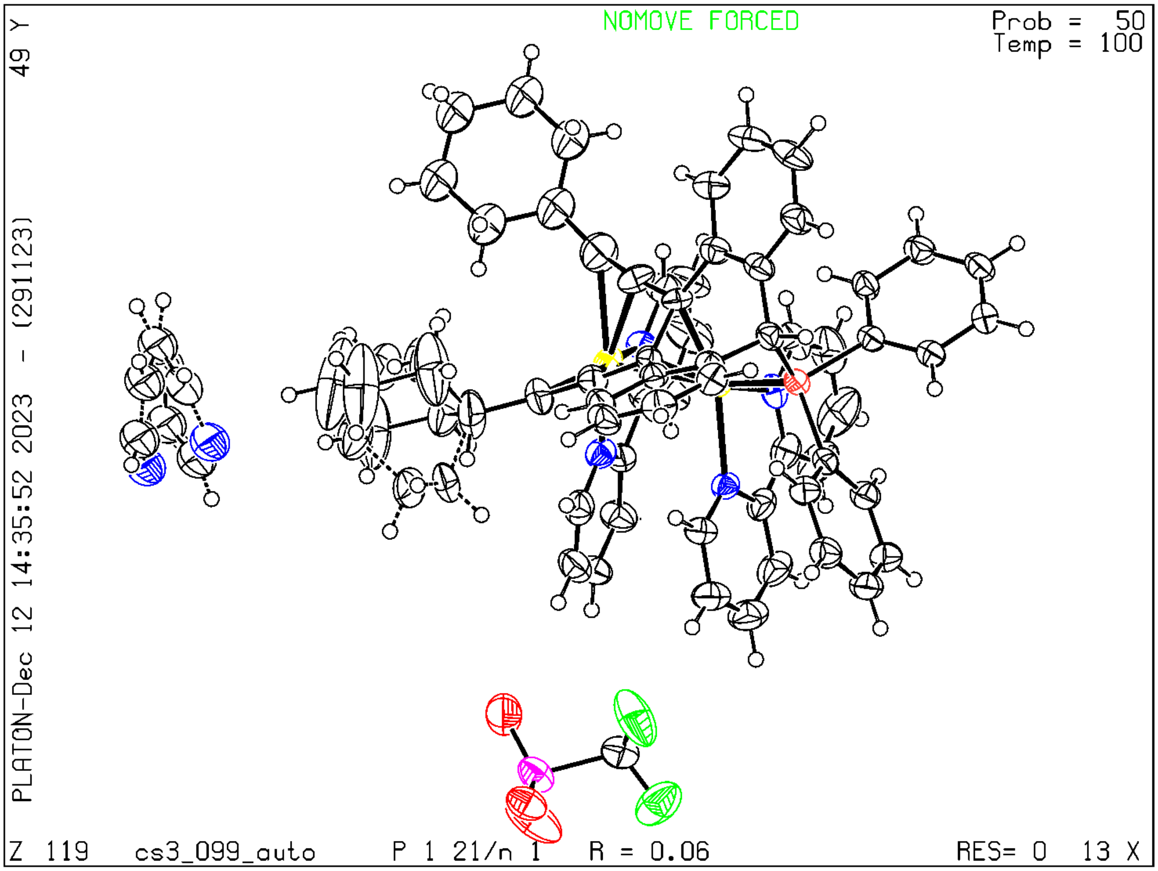

Supplement: Supplementary file 2 — Supporting Information [file ADVS-11-2400072-s001.zip › [4]OTf_Rh2_radical_2313436_cifreport.pdf]
