# Supplementary material for: Structural Changes in the Carbon Sphere of a Dirhodium Complex Induced by Redox or Deprotonation Reactions
Source: Adv Sci (Weinh). 2024 Mar 23;11(22):2400072. doi: 10.1002/advs.202400072 (PMC11165463; doi:10.1002/advs.202400072)

checkCIF/PLATON report

Structure factors have been supplied for datablock(s) cs129

THIS REPORT IS FOR GUIDANCE ONLY. IF USED AS PART OF A REVIEW PROCEDURE FOR PUBLICATION, IT SHOULD NOT REPLACE THE EXPERTISE OF AN EXPERIENCED CRYSTALLOGRAPHIC REFEREE.

No syntax errors found. CIF dictionary Interpreting this report

Datablock: cs129

Bond precision:	C-C = 0.0035 A		Wavelength=0.71073
Cell:	a=13.1434(3)	b=15.1938(4)	c=15.2523(3)
	alpha=79.262(2)	beta=65.253(2)	gamma=67.904(2)
Temperature:	100 K		

	Calculated	Reported
Volume	2561.47(12)	2561.48(12)
Space group	P -1	P -1
Hall group	-P 1	-P 1
Moiety formula	C86 H82 Cl2 P2 Rh2, 6(C7 H8)	C86 H82 Cl2 P2 Rh2, 6(C7 H8)
Sum formula	C128 H130 Cl2 P2 Rh2	C128 H130 Cl2 P2 Rh2
Mr	2006.98	2006.97
Dx,g cm-3	1.301	1.301
Z	1	1
Mu (mm-1)	0.457	0.457
F000	1052.0	1052.0
F000'	1050.58	
h,k,lmax	17,20,20	17,20,20
Nref	12970	11623
Tmin,Tmax	0.919,0.944	0.936,1.000
Tmin'	0.899	

Correction method= # Reported T Limits: Tmin=0.936 Tmax=1.000
AbsCorr = MULTI-SCAN

Data completeness= 0.896 Theta(max)= 28.488

R(reflections)= 0.0345(10424) wR2(reflections)= 0.0791(11623)

S = 1.051 Npar= 737

The following ALERTS were generated. Each ALERT has the format
test-name_ALERT_alert-type_alert-level.
Click on the hyperlinks for more details of the test.

Alert level C

```
PLAT220_ALERT_2_C NonSolvent   Resd 1  C   Ueq(max)/Ueq(min) Range        3.5 Ratio
PLAT250_ALERT_2_C Large U3/U1 Ratio for Average U(i,j) Tensor ....        3.0 Note
PLAT250_ALERT_2_C Large U3/U1 Ratio for Average U(i,j) Tensor ....        2.2 Note
```

Alert level G

```
PLAT154_ALERT_1_G The s.u.'s on the Cell Angles are Equal ..(Note)      0.002 Degree
PLAT187_ALERT_4_G The CIF-Embedded .res File Contains RIGU Records          2 Report
PLAT232_ALERT_2_G Hirshfeld Test Diff (M-X)  Rh1      --Cl2_a    .        6.3 s.u.
PLAT302_ALERT_4_G Anion/Solvent/Minor-Residue Disorder (Resd  3  )       100% Note
PLAT302_ALERT_4_G Anion/Solvent/Minor-Residue Disorder (Resd  4  )       100% Note
PLAT302_ALERT_4_G Anion/Solvent/Minor-Residue Disorder (Resd  5  )       100% Note
PLAT302_ALERT_4_G Anion/Solvent/Minor-Residue Disorder (Resd  6  )       100% Note
PLAT304_ALERT_4_G Non-Integer Number of Atoms in ..... (Resd  3  )       8.90 Check
PLAT304_ALERT_4_G Non-Integer Number of Atoms in ..... (Resd  4  )       7.89 Check
PLAT304_ALERT_4_G Non-Integer Number of Atoms in ..... (Resd  5  )       6.11 Check
PLAT304_ALERT_4_G Non-Integer Number of Atoms in ..... (Resd  6  )       7.11 Check
PLAT371_ALERT_2_G Long   C(sp2)-C(sp1) Bond  C65      - C88       .      1.45 Ang.
PLAT371_ALERT_2_G Long   C(sp2)-C(sp1) Bond  C66      - C67       .      1.45 Ang.
PLAT790_ALERT_4_G Centre of Gravity not Within Unit Cell: Resd.  #          4 Note
              C7 H8
PLAT790_ALERT_4_G Centre of Gravity not Within Unit Cell: Resd.  #          6 Note
              C7 H8
PLAT860_ALERT_3_G Number of Least-Squares Restraints .............         90 Note
PLAT910_ALERT_3_G Missing # of FCF Reflection(s) Below Theta(Min).          2 Note
                0  1  0,   0  0  1,
PLAT912_ALERT_4_G Missing # of FCF Reflections Above STh/L=  0.600       1344 Note
PLAT933_ALERT_2_G Number of HKL-OMIT Records in Embedded .res File          1 Note
                3 -5  1,
PLAT978_ALERT_2_G Number C-C Bonds with Positive Residual Density.          4 Info
```

0 **ALERT level A** = Most likely a serious problem - resolve or explain
0 **ALERT level B** = A potentially serious problem, consider carefully
3 **ALERT level C** = Check. Ensure it is not caused by an omission or oversight
20 **ALERT level G** = General information/check it is not something unexpected

1 ALERT type 1 CIF construction/syntax error, inconsistent or missing data
8 ALERT type 2 Indicator that the structure model may be wrong or deficient
2 ALERT type 3 Indicator that the structure quality may be low
12 ALERT type 4 Improvement, methodology, query or suggestion
0 ALERT type 5 Informative message, check

It is advisable to attempt to resolve as many as possible of the alerts in all categories. Often the minor alerts point to easily fixed oversights, errors and omissions in your CIF or refinement strategy, so attention to these fine details can be worthwhile. In order to resolve some of the more serious problems it may be necessary to carry out additional measurements or structure refinements. However, the purpose of your study may justify the reported deviations and the more serious of these should normally be commented upon in the discussion or experimental section of a paper or in the "special_details" fields of the CIF. checkCIF was carefully designed to identify outliers and unusual parameters, but every test has its limitations and alerts that are not important in a particular case may appear. Conversely, the absence of alerts does not guarantee there are no aspects of the results needing attention. It is up to the individual to critically assess their own results and, if necessary, seek expert advice.

Publication of your CIF in IUCr journals

A basic structural check has been run on your CIF. These basic checks will be run on all CIFs submitted for publication in IUCr journals (*Acta Crystallographica*, *Journal of Applied Crystallography*, *Journal of Synchrotron Radiation*); however, if you intend to submit to *Acta Crystallographica Section C* or *E* or *IUCrData*, you should make sure that full publication checks are run on the final version of your CIF prior to submission.

Publication of your CIF in other journals

Please refer to the *Notes for Authors* of the relevant journal for any special instructions relating to CIF submission.

PLATON version of 29/11/2023; check.def file version of 14/09/2023

Datablock cs129 - ellipsoid plot

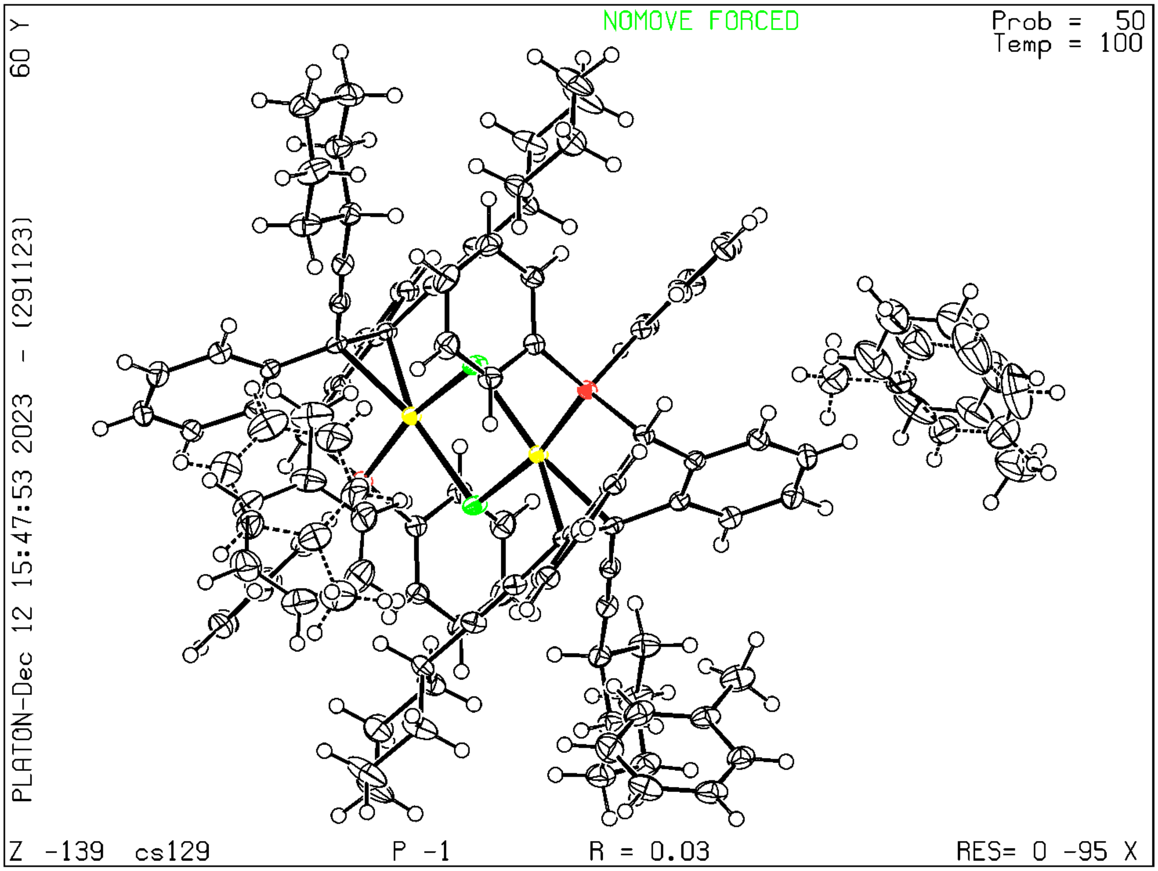

Supplement: Supplementary file 2 — Supporting Information [file ADVS-11-2400072-s001.zip › [5]_RhCl_2313428_cifreport.pdf]
