# Supplementary material for: Structural Changes in the Carbon Sphere of a Dirhodium Complex Induced by Redox or Deprotonation Reactions
Source: Adv Sci (Weinh). 2024 Mar 23;11(22):2400072. doi: 10.1002/advs.202400072 (PMC11165463; doi:10.1002/advs.202400072)

checkCIF/PLATON report

Structure factors have been supplied for datablock(s) cs4_072_thf_hex2_auto

THIS REPORT IS FOR GUIDANCE ONLY. IF USED AS PART OF A REVIEW PROCEDURE FOR PUBLICATION, IT SHOULD NOT REPLACE THE EXPERTISE OF AN EXPERIENCED CRYSTALLOGRAPHIC REFEREE.

No syntax errors found. CIF dictionary Interpreting this report

Datablock: cs4_072_thf_hex2_auto

```
Bond precision:   C-C = 0.0045 A          Wavelength=1.54184

Cell:            a=19.2301(1)     b=25.7889(1)        c=21.4450(1)
                 alpha=90         beta=112.298(1)     gamma=90
Temperature:     100 K
```

	Calculated	Reported
Volume	9839.81(10)	9839.81(10)
Space group	P 21/c	P 1 21/c 1
Hall group	-P 2ybc	-P 2ybc
Moiety formula	C106 H98 N4 P2 Rh2, 3(C4 H8 O) [+ solvent]	C106 H98 N4 P2 Rh2, 3(C4 H8 O)
Sum formula	C118 H122 N4 O3 P2 Rh2 [+ solvent]	C118 H122 N4 O3 P2 Rh2
Mr	1911.96	1911.95
Dx,g cm-3	1.291	1.291
Z	4	4
Mu (mm-1)	3.441	3.441
F000	4008.0	4008.0
F000'	4020.39	
h,k,lmax	24,32,27	24,32,27
Nref	21528	21411
Tmin,Tmax	0.917,0.940	0.540,1.000
Tmin'	0.561	

```
Correction method= # Reported T Limits: Tmin=0.540 Tmax=1.000
AbsCorr = MULTI-SCAN

Data completeness= 0.995            Theta(max)= 80.287
```

R(reflections)= 0.0458(20161)

wR2(reflections)= 0.1191(21411)

S = 1.083   Npar= 1196

The following ALERTS were generated. Each ALERT has the format
test-name_ALERT_alert-type_alert-level.
Click on the hyperlinks for more details of the test.

Alert level C

```
PLAT220_ALERT_2_C NonSolvent   Resd 1  C   Ueq(max)/Ueq(min) Range        3.4 Ratio
PLAT230_ALERT_2_C Hirshfeld Test Diff for    C6       --C10      .        5.2 s.u.
PLAT250_ALERT_2_C Large U3/U1 Ratio for Average U(i,j) Tensor ....        2.3 Note
PLAT906_ALERT_3_C Large K Value in the Analysis of Variance ......      2.890 Check
PLAT971_ALERT_2_C Check Calcd Resid. Dens.  0.82Ang From C1              1.68 eA-3
PLAT971_ALERT_2_C Check Calcd Resid. Dens.  1.43Ang From C39             1.58 eA-3
```

Alert level G

```
PLAT002_ALERT_2_G Number of Distance or Angle Restraints on AtSite          5 Note
PLAT003_ALERT_2_G Number of Uiso or Uij Restrained non-H Atoms ...          5 Report
PLAT083_ALERT_2_G SHELXL Second Parameter in WGHT  Unusually Large      19.56 Why ?
PLAT093_ALERT_1_G No s.u.'s on H-positions, Refinement Reported as      mixed Check
PLAT142_ALERT_4_G s.u. on b - Axis Small or Missing .............     0.00010 Ang.
PLAT143_ALERT_4_G s.u. on c - Axis Small or Missing .............     0.00010 Ang.
PLAT171_ALERT_4_G The CIF-Embedded .res File Contains EADP Records          1 Report
PLAT172_ALERT_4_G The CIF-Embedded .res File Contains DFIX Records          3 Report
PLAT173_ALERT_4_G The CIF-Embedded .res File Contains DANG Records          1 Report
PLAT176_ALERT_4_G The CIF-Embedded .res File Contains SADI Records          2 Report
PLAT178_ALERT_4_G The CIF-Embedded .res File Contains SIMU Records          1 Report
PLAT187_ALERT_4_G The CIF-Embedded .res File Contains RIGU Records          1 Report
PLAT191_ALERT_3_G A Non-default SADI Restraint Value has been used     0.0400 Report
PLAT302_ALERT_4_G Anion/Solvent/Minor-Residue Disorder (Resd  2  )        20% Note
PLAT302_ALERT_4_G Anion/Solvent/Minor-Residue Disorder (Resd  4  )       100% Note
PLAT302_ALERT_4_G Anion/Solvent/Minor-Residue Disorder (Resd  5  )       100% Note
PLAT304_ALERT_4_G Non-Integer Number of Atoms in ..... (Resd  4  )       8.58 Check
PLAT304_ALERT_4_G Non-Integer Number of Atoms in ..... (Resd  5  )       4.42 Check
PLAT343_ALERT_2_G Unusual sp?      Angle Range in Main Residue for         C6 Check
PLAT343_ALERT_2_G Unusual sp?      Angle Range in Main Residue for        C16 Check
PLAT371_ALERT_2_G Long   C(sp2)-C(sp1) Bond  C4       - C20      .       1.46 Ang.
PLAT371_ALERT_2_G Long   C(sp2)-C(sp1) Bond  C26      - C58      .       1.45 Ang.
PLAT398_ALERT_2_G Deviating  C-O-C   Angle From 120 for O4       .      108.7 Degree
PLAT398_ALERT_2_G Deviating  C-O-C   Angle From 120 for O1       .      105.3 Degree
PLAT398_ALERT_2_G Deviating  C-O-C   Angle From 120 for O115     .      105.5 Degree
PLAT411_ALERT_2_G Short Inter H...H Contact  H23      ..H59D     .       2.08 Ang.
                                                      x,y,z  =      1_555 Check
PLAT605_ALERT_4_G Largest Solvent Accessible VOID in the Structure        357 A**3
PLAT720_ALERT_4_G Number of Unusual/Non-Standard Labels ..........          4 Note
              H7BC    H7BD    H1AA    H1AB
PLAT773_ALERT_2_G Check long C-C Bond in CIF: C1A      --C59A            1.72 Ang.
PLAT790_ALERT_4_G Centre of Gravity not Within Unit Cell: Resd.  #          4 Note
              C4 H8 O
PLAT790_ALERT_4_G Centre of Gravity not Within Unit Cell: Resd.  #          5 Note
              C4 H8 O
PLAT860_ALERT_3_G Number of Least-Squares Restraints ............          73 Note
```

```
PLAT868_ALERT_4_G ALERTS Due to the Use of _smtbx_masks Suppressed          ! Info
PLAT912_ALERT_4_G Missing # of FCF Reflections Above STh/L=  0.600        117 Note
PLAT933_ALERT_2_G Number of HKL-OMIT Records in Embedded .res File          1 Note
              -19  0 22,
PLAT978_ALERT_2_G Number C-C Bonds with Positive Residual Density.          4 Info
```

```
   0 ALERT level A = Most likely a serious problem - resolve or explain
   0 ALERT level B = A potentially serious problem, consider carefully
   6 ALERT level C = Check. Ensure it is not caused by an omission or oversight
  36 ALERT level G = General information/check it is not something unexpected

   1 ALERT type 1 CIF construction/syntax error, inconsistent or missing data
  19 ALERT type 2 Indicator that the structure model may be wrong or deficient
   3 ALERT type 3 Indicator that the structure quality may be low
  19 ALERT type 4 Improvement, methodology, query or suggestion
   0 ALERT type 5 Informative message, check
```

It is advisable to attempt to resolve as many as possible of the alerts in all categories. Often the minor alerts point to easily fixed oversights, errors and omissions in your CIF or refinement strategy, so attention to these fine details can be worthwhile. In order to resolve some of the more serious problems it may be necessary to carry out additional measurements or structure refinements. However, the purpose of your study may justify the reported deviations and the more serious of these should normally be commented upon in the discussion or experimental section of a paper or in the "special_details" fields of the CIF. checkCIF was carefully designed to identify outliers and unusual parameters, but every test has its limitations and alerts that are not important in a particular case may appear. Conversely, the absence of alerts does not guarantee there are no aspects of the results needing attention. It is up to the individual to critically assess their own results and, if necessary, seek expert advice.

Publication of your CIF in IUCr journals

A basic structural check has been run on your CIF. These basic checks will be run on all CIFs submitted for publication in IUCr journals (*Acta Crystallographica*, *Journal of Applied Crystallography*, *Journal of Synchrotron Radiation*); however, if you intend to submit to *Acta Crystallographica Section C* or *E* or *IUCrData*, you should make sure that full publication checks are run on the final version of your CIF prior to submission.

Publication of your CIF in other journals

Please refer to the *Notes for Authors* of the relevant journal for any special instructions relating to CIF submission.

PLATON version of 29/11/2023; check.def file version of 14/09/2023

Datablock cs4_072_thf_hex2_auto - ellipsoid plot

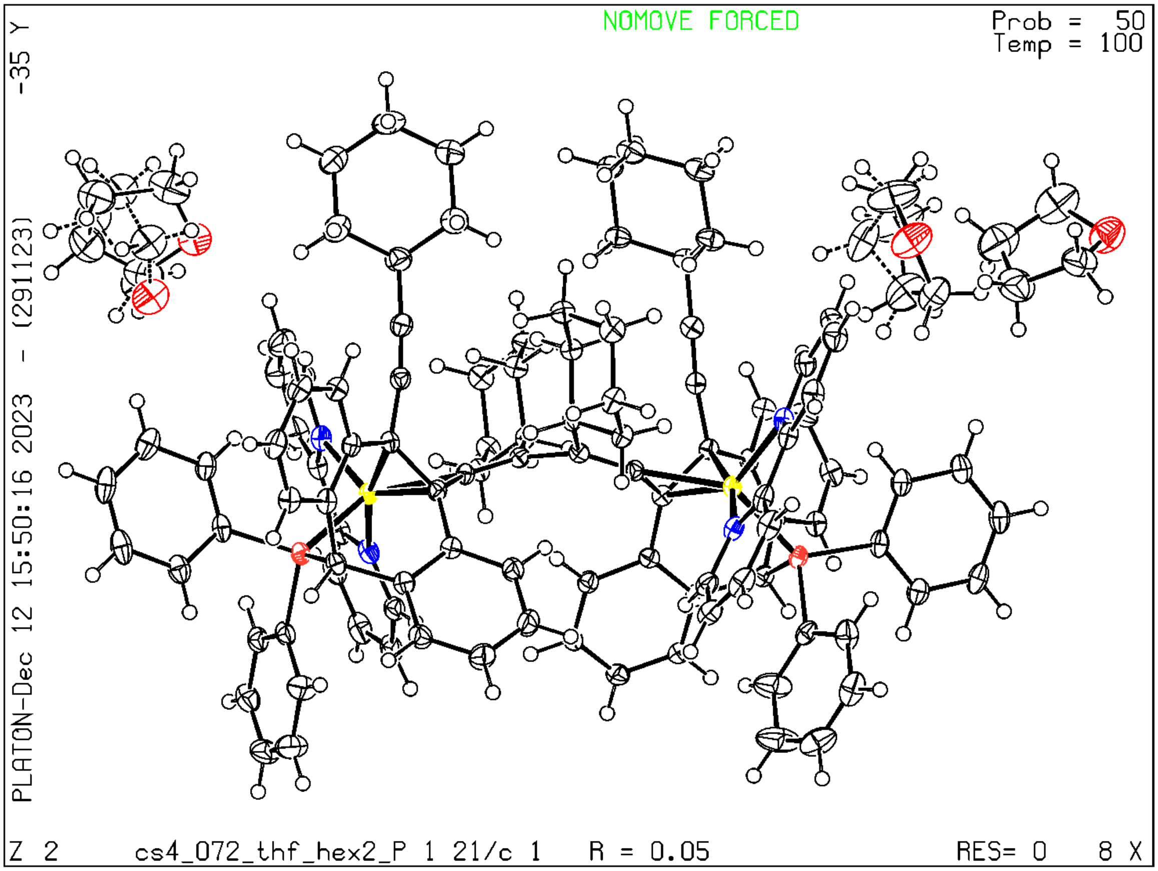

Supplement: Supplementary file 2 — Supporting Information [file ADVS-11-2400072-s001.zip › [6]2_Rh-Rh_2313427_cifreport.pdf]
