# Supplementary material for: Structural Changes in the Carbon Sphere of a Dirhodium Complex Induced by Redox or Deprotonation Reactions
Source: Adv Sci (Weinh). 2024 Mar 23;11(22):2400072. doi: 10.1002/advs.202400072 (PMC11165463; doi:10.1002/advs.202400072)

checkCIF/PLATON report

Structure factors have been supplied for datablock(s) cs4_077_chcl3_hex_auto

THIS REPORT IS FOR GUIDANCE ONLY. IF USED AS PART OF A REVIEW PROCEDURE FOR PUBLICATION, IT SHOULD NOT REPLACE THE EXPERTISE OF AN EXPERIENCED CRYSTALLOGRAPHIC REFEREE.

No syntax errors found. CIF dictionary Interpreting this report

Datablock: cs4_077_chcl3_hex_auto

Bond precision: C-C = 0.0052 A Wavelength=1.54184

Cell:	a=9.6572(1)	b=12.0031(1)	c=22.2473(2)
	alpha=100.398(1)	beta=96.546(1)	gamma=99.738(1)
Temperature:	100 K		

	Calculated	Reported
Volume	2471.99(4)	2471.99(4)
Space group	P -1	P -1
Hall group	-P 1	-P 1
Moiety formula	C53 H49 N2 P Rh, C F3 O3 S, C H Cl3	C53 H49 N2 P Rh, C F3 O3 S, C H Cl3
Sum formula	C55 H50 Cl3 F3 N2 O3 P Rh S	C55 H50 Cl3 F3 N2 O3 P Rh S
Mr	1116.26	1116.26
Dx,g cm-3	1.500	1.500
Z	2	2
Mu (mm-1)	5.469	5.469
F000	1144.0	1144.0
F000'	1150.44	
h,k,lmax	12,15,28	12,15,28
Nref	10892	10595
Tmin,Tmax	0.754,0.826	0.811,1.000
Tmin'	0.465	

Correction method= # Reported T Limits: Tmin=0.811 Tmax=1.000
AbsCorr = MULTI-SCAN

Data completeness= 0.973 Theta(max)= 80.950

R(reflections)= 0.0464(9611) wR2(reflections)= 0.1296(10595)

S = 1.059 Npar= 631

The following ALERTS were generated. Each ALERT has the format
test-name_ALERT_alert-type_alert-level.
Click on the hyperlinks for more details of the test.

Alert level C

```
PLAT220_ALERT_2_C NonSolvent   Resd 1  C   Ueq(max)/Ueq(min) Range        3.4 Ratio
PLAT241_ALERT_2_C High   'MainMol' Ueq as Compared to Neighbors of         C5 Check
PLAT242_ALERT_2_C Low    'MainMol' Ueq as Compared to Neighbors of        C45 Check
PLAT244_ALERT_4_C Low    'Solvent' Ueq as Compared to Neighbors of         S1 Check
PLAT250_ALERT_2_C Large U3/U1 Ratio for Average U(i,j) Tensor ....        2.2 Note
PLAT336_ALERT_2_C Long Bond Distance for ..... C14      -Cl2            2.001 Ang.
PLAT911_ALERT_3_C Missing FCF Refl Between Thmin & STh/L=    0.600         10 Report
                2-14  7,   4-14  7,   1-14  8,   2-14  8,   3-14  8,   1-14  9,
                2-14  9,   3-14  9,   1-14 10,   2-14 10,
PLAT934_ALERT_3_C Number of (Iobs-Icalc)/Sigma(W) > 10 Outliers ..          1 Check
               -2  3  3,
PLAT971_ALERT_2_C Check Calcd Resid. Dens.  1.09Ang From C5              1.55 eA-3
PLAT977_ALERT_2_C Check Negative Difference Density on H16B      .      -0.31 eA-3
PLAT977_ALERT_2_C Check Negative Difference Density on H53A      .      -0.32 eA-3
```

Alert level G

```
PLAT002_ALERT_2_G Number of Distance or Angle Restraints on AtSite          5 Note
PLAT083_ALERT_2_G SHELXL Second Parameter in WGHT  Unusually Large       5.16 Why ?
PLAT154_ALERT_1_G The s.u.'s on the Cell Angles are Equal ..(Note)      0.001 Degree
PLAT176_ALERT_4_G The CIF-Embedded .res File Contains SADI Records          1 Report
PLAT244_ALERT_4_G Low    'Solvent' Ueq as Compared to Neighbors of        C23 Check
PLAT300_ALERT_4_G Atom Site Occupancy of Cl1        Constrained at        0.9 Check
PLAT300_ALERT_4_G Atom Site Occupancy of Cl2        Constrained at        0.1 Check
PLAT300_ALERT_4_G Atom Site Occupancy of H14        Constrained at        0.9 Check
PLAT300_ALERT_4_G Atom Site Occupancy of H14A       Constrained at        0.1 Check
PLAT302_ALERT_4_G Anion/Solvent/Minor-Residue Disorder (Resd  3  )        25% Note
PLAT371_ALERT_2_G Long   C(sp2)-C(sp1) Bond  C1       - C6       .       1.44 Ang.
PLAT371_ALERT_2_G Long   C(sp2)-C(sp1) Bond  C2       - C3       .       1.44 Ang.
PLAT432_ALERT_2_G Short Inter X...Y Contact  Cl2      ..C53      .       2.91 Ang.
                                                 -x,-y,1-z  =      2_556 Check
PLAT432_ALERT_2_G Short Inter X...Y Contact  Cl2      ..C51      .       2.95 Ang.
                                                 -x,-y,1-z  =      2_556 Check
PLAT860_ALERT_3_G Number of Least-Squares Restraints ............           6 Note
PLAT910_ALERT_3_G Missing # of FCF Reflection(s) Below Theta(Min).          1 Note
                0  0  1,
PLAT912_ALERT_4_G Missing # of FCF Reflections Above STh/L=  0.600        285 Note
PLAT978_ALERT_2_G Number C-C Bonds with Positive Residual Density.          0 Info
```

0 **ALERT level A** = Most likely a serious problem - resolve or explain
0 **ALERT level B** = A potentially serious problem, consider carefully
11 **ALERT level C** = Check. Ensure it is not caused by an omission or oversight
18 **ALERT level G** = General information/check it is not something unexpected

1 ALERT type 1 CIF construction/syntax error, inconsistent or missing data
15 ALERT type 2 Indicator that the structure model may be wrong or deficient
4 ALERT type 3 Indicator that the structure quality may be low
9 ALERT type 4 Improvement, methodology, query or suggestion
0 ALERT type 5 Informative message, check

It is advisable to attempt to resolve as many as possible of the alerts in all categories. Often the minor alerts point to easily fixed oversights, errors and omissions in your CIF or refinement strategy, so attention to these fine details can be worthwhile. In order to resolve some of the more serious problems it may be necessary to carry out additional measurements or structure refinements. However, the purpose of your study may justify the reported deviations and the more serious of these should normally be commented upon in the discussion or experimental section of a paper or in the "special_details" fields of the CIF. checkCIF was carefully designed to identify outliers and unusual parameters, but every test has its limitations and alerts that are not important in a particular case may appear. Conversely, the absence of alerts does not guarantee there are no aspects of the results needing attention. It is up to the individual to critically assess their own results and, if necessary, seek expert advice.

Publication of your CIF in IUCr journals

A basic structural check has been run on your CIF. These basic checks will be run on all CIFs submitted for publication in IUCr journals (*Acta Crystallographica*, *Journal of Applied Crystallography*, *Journal of Synchrotron Radiation*); however, if you intend to submit to *Acta Crystallographica Section C* or *E* or *IUCrData*, you should make sure that full publication checks are run on the final version of your CIF prior to submission.

Publication of your CIF in other journals

Please refer to the *Notes for Authors* of the relevant journal for any special instructions relating to CIF submission.

PLATON version of 29/11/2023; check.def file version of 14/09/2023

Datablock cs4_077_chcl3_hex_auto - ellipsoid plot

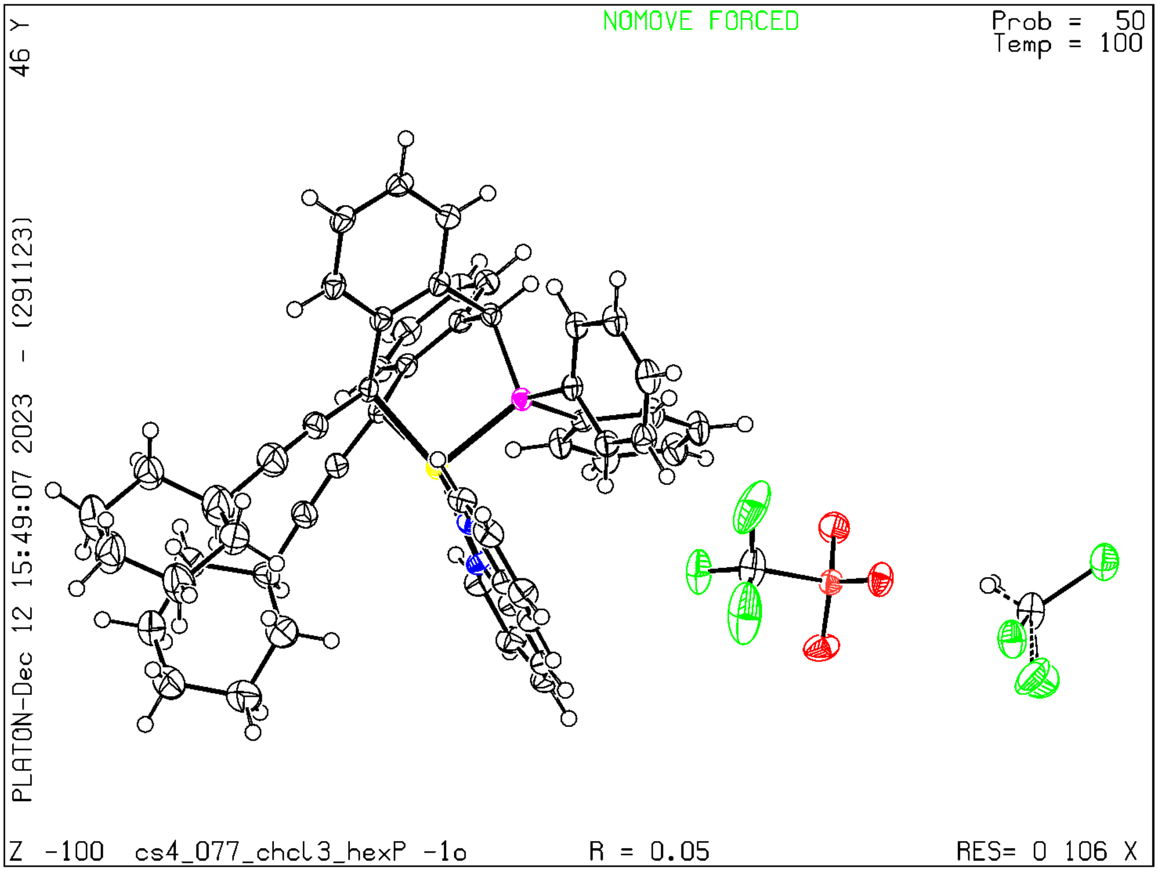

Supplement: Supplementary file 2 — Supporting Information [file ADVS-11-2400072-s001.zip › [6]OTf_RhbipyOTf_2313426_cifreport.pdf]
