# Supplementary material for: Structural Changes in the Carbon Sphere of a Dirhodium Complex Induced by Redox or Deprotonation Reactions
Source: Adv Sci (Weinh). 2024 Mar 23;11(22):2400072. doi: 10.1002/advs.202400072 (PMC11165463; doi:10.1002/advs.202400072)

checkCIF/PLATON report

Structure factors have been supplied for datablock(s) cs3_100_needle_auto

THIS REPORT IS FOR GUIDANCE ONLY. IF USED AS PART OF A REVIEW PROCEDURE FOR PUBLICATION, IT SHOULD NOT REPLACE THE EXPERTISE OF AN EXPERIENCED CRYSTALLOGRAPHIC REFEREE.

No syntax errors found. CIF dictionary Interpreting this report

Datablock: cs3_100_needle_auto

Bond precision: C-C = 0.0099 A Wavelength=1.54184

Cell:	a=9.8012(4)	b=13.3777(4)	c=21.7694(6)
	alpha=102.651(2)	beta=96.454(3)	gamma=96.355(3)
Temperature:	100 K		

	Calculated	Reported
Volume	2740.32(16)	2740.32(16)
Space group	P -1	P -1
Hall group	-P 1	-P 1
Moiety formula	C63 H57 N4 P Rh2, C5 H5 N [+ solvent]	C63 H57 N4 P Rh2, C5 H5 N
Sum formula	C68 H62 N5 P Rh2 [+ solvent]	C68 H62 N5 P Rh2
Mr	1186.02	1186.01
Dx,g cm-3	1.437	1.437
Z	2	2
Mu (mm-1)	5.510	5.510
F000	1220.0	1220.0
F000’	1223.67	
h,k,lmax	11,16,26	11,16,26
Nref	10026	10005
Tmin,Tmax	0.948,0.962	0.642,1.000
Tmin’	0.545	

Correction method= # Reported T Limits: Tmin=0.642 Tmax=1.000
AbsCorr = MULTI-SCAN

Data completeness= 0.998 Theta(max)= 68.249

R(reflections)= 0.0625(7387) wR2(reflections)= 0.1704(10005)

S = 1.008 Npar= 685

The following ALERTS were generated. Each ALERT has the format
test-name_ALERT_alert-type_alert-level.
Click on the hyperlinks for more details of the test.

Alert level C

```
PLAT342_ALERT_3_C Low Bond Precision on  C-C Bonds ...............     0.0099 Ang.
PLAT411_ALERT_2_C Short Inter H...H Contact  H61       ..H65      .       2.09 Ang.
                                                  x,y,z  =      1_555 Check
PLAT911_ALERT_3_C Missing FCF Refl Between Thmin & STh/L=    0.600          13 Report
                0  1  0,  -1 -5  1,  -1 -1  1,  11  1  4,   6 11  4,   1-16  5,
                2-16  5,   1-16  6,   2-16  6,   1-16  7,   2-15  7,   3-15  7,
                7  6 12,
PLAT971_ALERT_2_C Check Calcd Resid. Dens.  1.05Ang From Rh1            2.42 eA-3
PLAT971_ALERT_2_C Check Calcd Resid. Dens.  1.28Ang From C65            1.77 eA-3
PLAT971_ALERT_2_C Check Calcd Resid. Dens.  1.02Ang From Rh2            1.76 eA-3
PLAT971_ALERT_2_C Check Calcd Resid. Dens.  1.05Ang From Rh2            1.73 eA-3
PLAT971_ALERT_2_C Check Calcd Resid. Dens.  1.03Ang From Rh1            1.62 eA-3
PLAT971_ALERT_2_C Check Calcd Resid. Dens.  1.14Ang From C31            1.61 eA-3
PLAT972_ALERT_2_C Check Calcd Resid. Dens.  0.79Ang From Rh1           -1.70 eA-3
PLAT972_ALERT_2_C Check Calcd Resid. Dens.  0.81Ang From Rh2           -1.54 eA-3
PLAT977_ALERT_2_C Check Negative Difference Density on H23A      .     -0.34 eA-3
```

Alert level G

```
PLAT072_ALERT_2_G SHELXL First  Parameter in WGHT  Unusually Large       0.11 Report
PLAT343_ALERT_2_G Unusual sp?      Angle Range in Main Residue for         C3 Check
PLAT343_ALERT_2_G Unusual sp?      Angle Range in Main Residue for         C6 Check
PLAT605_ALERT_4_G Largest Solvent Accessible VOID in the Structure        159 A**3
PLAT790_ALERT_4_G Centre of Gravity not Within Unit Cell: Resd.  #          2 Note
              C5 H5 N
PLAT868_ALERT_4_G ALERTS Due to the Use of _smtbx_masks Suppressed          ! Info
PLAT912_ALERT_4_G Missing # of FCF Reflections Above STh/L=  0.600          8 Note
PLAT941_ALERT_3_G Average HKL Measurement Multiplicity ...........        3.7 Low
PLAT978_ALERT_2_G Number C-C Bonds with Positive Residual Density.          1 Info
```

---

0 **ALERT level A** = Most likely a serious problem - resolve or explain
0 **ALERT level B** = A potentially serious problem, consider carefully
12 **ALERT level C** = Check. Ensure it is not caused by an omission or oversight
9 **ALERT level G** = General information/check it is not something unexpected

0 ALERT type 1 CIF construction/syntax error, inconsistent or missing data
14 ALERT type 2 Indicator that the structure model may be wrong or deficient
3 ALERT type 3 Indicator that the structure quality may be low
4 ALERT type 4 Improvement, methodology, query or suggestion
0 ALERT type 5 Informative message, check

It is advisable to attempt to resolve as many as possible of the alerts in all categories. Often the minor alerts point to easily fixed oversights, errors and omissions in your CIF or refinement strategy, so attention to these fine details can be worthwhile. In order to resolve some of the more serious problems it may be necessary to carry out additional measurements or structure refinements. However, the purpose of your study may justify the reported deviations and the more serious of these should normally be commented upon in the discussion or experimental section of a paper or in the "special_details" fields of the CIF. checkCIF was carefully designed to identify outliers and unusual parameters, but every test has its limitations and alerts that are not important in a particular case may appear. Conversely, the absence of alerts does not guarantee there are no aspects of the results needing attention. It is up to the individual to critically assess their own results and, if necessary, seek expert advice.

Publication of your CIF in IUCr journals

A basic structural check has been run on your CIF. These basic checks will be run on all CIFs submitted for publication in IUCr journals (*Acta Crystallographica*, *Journal of Applied Crystallography*, *Journal of Synchrotron Radiation*); however, if you intend to submit to *Acta Crystallographica Section C* or *E* or *IUCrData*, you should make sure that full publication checks are run on the final version of your CIF prior to submission.

Publication of your CIF in other journals

Please refer to the *Notes for Authors* of the relevant journal for any special instructions relating to CIF submission.

PLATON version of 29/11/2023; check.def file version of 14/09/2023

Datablock cs3_100_needle_auto - ellipsoid plot

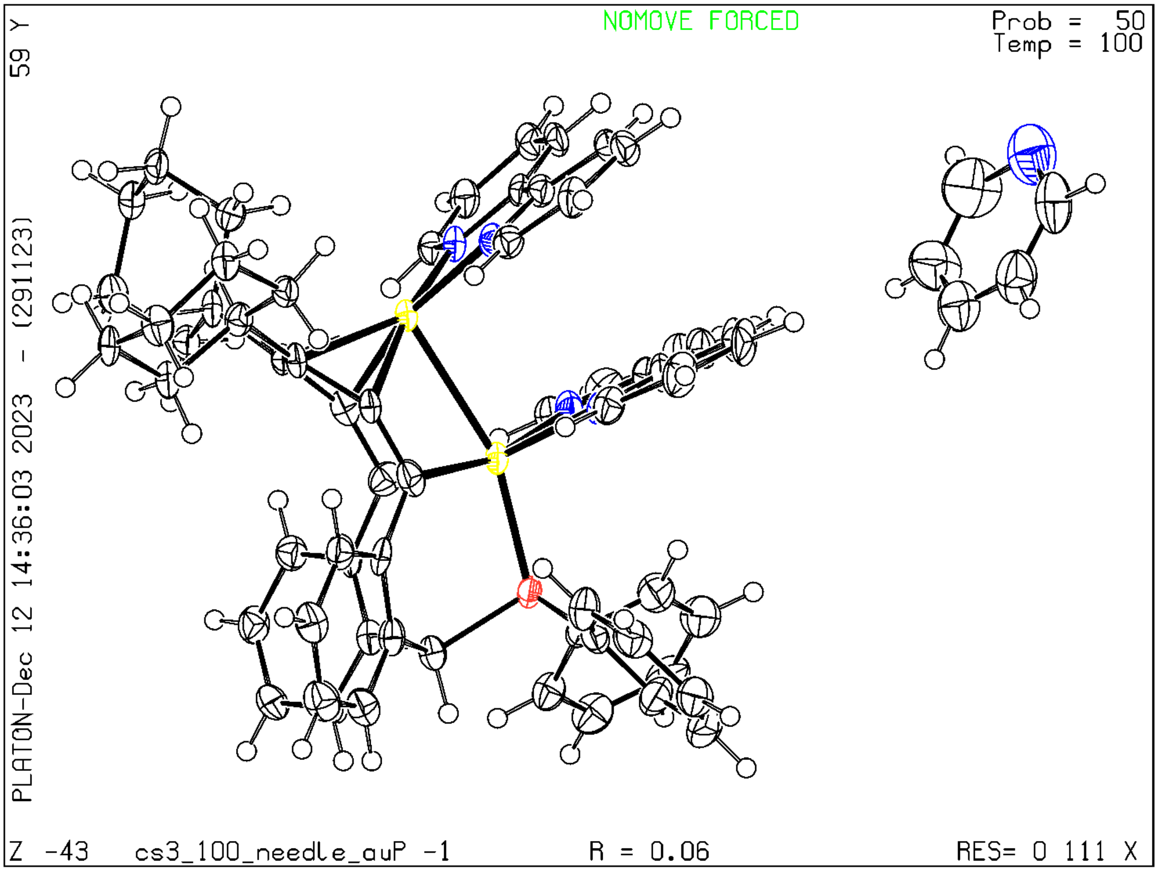

Supplement: Supplementary file 2 — Supporting Information [file ADVS-11-2400072-s001.zip › [7]_Rh2(0)_2313511_cifreport.pdf]
