# Supplementary material for: Structural Changes in the Carbon Sphere of a Dirhodium Complex Induced by Redox or Deprotonation Reactions
Source: Adv Sci (Weinh). 2024 Mar 23;11(22):2400072. doi: 10.1002/advs.202400072 (PMC11165463; doi:10.1002/advs.202400072)

checkCIF/PLATON report

Structure factors have been supplied for datablock(s) cs2_100_1_auto-part1and2,occup0.667and0.333

THIS REPORT IS FOR GUIDANCE ONLY. IF USED AS PART OF A REVIEW PROCEDURE FOR PUBLICATION, IT SHOULD NOT REPLACE THE EXPERTISE OF AN EXPERIENCED CRYSTALLOGRAPHIC REFEREE.

No syntax errors found. CIF dictionary Interpreting this report

Datablock: cs2_100_1_auto-part1and2,occup0.667and0.333

```
Bond precision:   C-C = 0.0092 A          Wavelength=1.54184

Cell:             a=21.5586(6)     b=9.9941(3)        c=26.8646(10)
                  alpha=90         beta=90.466(3)     gamma=90
Temperature:      100 K
```

	Calculated	Reported
Volume	5788.0(3)	5788.0(3)
Space group	P 21/c	P 1 21/c 1
Hall group	-P 2ybc	-P 2ybc
Moiety formula	4(C62.75 H53.67 N4 P Rh2), 3(C F3 O3 S), 4(C0.13 F0.68 O0.75 S0	C62.748 H53.67 N4 P Rh2, 0.75(C F3 O3 S), C4 H9 O, C0.13 F0.68
Sum formula	C270.53 H250.68 F11.72 N16 O16 P4 Rh8 S4	C67.88 H66.49 F2.93 N4 O4 P Rh2 S
Mr	5280.07	1326.85
Dx,g cm-3	1.515	1.523
Z	1	4
Mu (mm-1)	5.732	5.733
F000	2703.4	2725.0
F000’	2713.55	
h,k,lmax	27,12,34	27,12,34
Nref	12750	12345
Tmin,Tmax	0.744,0.795	0.879,1.000
Tmin’	0.359	

```
Correction method= # Reported T Limits: Tmin=0.879 Tmax=1.000
AbsCorr = MULTI-SCAN

Data completeness= 0.968          Theta(max)= 80.937
```

R(reflections)= 0.0623(11356) wR2(reflections)= 0.1390(12345)

S = 1.114 Npar= 958

The following ALERTS were generated. Each ALERT has the format
test-name_ALERT_alert-type_alert-level.
Click on the hyperlinks for more details of the test.

Alert level B

```
PLAT230_ALERT_2_B Hirshfeld Test Diff for    C13      --C49      .       14.0 s.u.
```

Alert level C

```
PLAT041_ALERT_1_C Calc. and Reported SumFormula    Strings  Differ     Please Check
              Calc: C67.63 H62.67 F2.93 N4 O4 P Rh2 S
              Rep.: C67.88 H66.49 F2.93 N4 O4 P Rh2 S
PLAT042_ALERT_1_C Calc. and Reported MoietyFormula Strings  Differ     Please Check
              Calc: 4(C62.75 H53.67 N4 P Rh2), 3(C F3 O3 S), 4(C0.13 F0.68 O0.75
              Rep.: C62.748 H53.67 N4 P Rh2, 0.75(C F3 O3 S), C4 H
                    9 O, C0.13 F0.68 O0.75 S0.25
PLAT043_ALERT_1_C Calculated and Reported Mol. Weight Differ by ..      27.33 Check
PLAT068_ALERT_1_C Reported F000 Differs from Calcd (or Missing)...     Please Check
PLAT077_ALERT_4_C Unitcell Contains Non-integer Number of Atoms ..     Please Check
PLAT213_ALERT_2_C Atom C57             has ADP max/min Ratio .....        3.1 prolat
PLAT214_ALERT_2_C Atom F5        (Anion/Solvent) ADP max/min Ratio        5.0 prolat
PLAT220_ALERT_2_C NonSolvent   Resd 1  C    Ueq(max)/Ueq(min) Range       3.9 Ratio
PLAT234_ALERT_4_C Large Hirshfeld Difference C9       --C39      .       0.16 Ang.
PLAT234_ALERT_4_C Large Hirshfeld Difference C27      --C47      .       0.17 Ang.
PLAT234_ALERT_4_C Large Hirshfeld Difference C61      --C67      .       0.20 Ang.
PLAT234_ALERT_4_C Large Hirshfeld Difference F1       --C51      .       0.17 Ang.
PLAT244_ALERT_4_C Low    'Solvent' Ueq as Compared to Neighbors of        C63 Check
PLAT250_ALERT_2_C Large U3/U1 Ratio for Average U(i,j) Tensor ....        2.4 Note
PLAT260_ALERT_2_C Large Average Ueq of Residue Including        S6      0.118 Check
PLAT260_ALERT_2_C Large Average Ueq of Residue Including        O3      0.139 Check
PLAT329_ALERT_4_C Carbon Atom Hybridisation Unclear for .........         C31 Check
PLAT329_ALERT_4_C Carbon Atom Hybridisation Unclear for .........         C61 Check
PLAT342_ALERT_3_C Low Bond Precision on  C-C Bonds ..............     0.0092 Ang.
PLAT360_ALERT_2_C Short  C(sp3)-C(sp3) Bond  C33       - C63      .       1.39 Ang.
PLAT411_ALERT_2_C Short Inter H...H Contact  H35      ..H44      .       2.14 Ang.
                                            2-x,1/2+y,1/2-z  =      2_755 Check
PLAT767_ALERT_4_C INS Embedded LIST 6 Instruction Should be LIST 4     Please Check
PLAT906_ALERT_3_C Large K Value in the Analysis of Variance ......      5.818 Check
PLAT911_ALERT_3_C Missing FCF Refl Between Thmin & STh/L=    0.600          3 Report
               11  0  0,   6 10 15,   6 10 16,
PLAT975_ALERT_2_C Check Calcd Resid. Dens.  1.06Ang From C59      .       0.53 eA-3
PLAT976_ALERT_2_C Check Calcd Resid. Dens.  0.71Ang From O3       .      -0.48 eA-3
PLAT977_ALERT_2_C Check Negative Difference Density on H7         .      -0.35 eA-3
PLAT977_ALERT_2_C Check Negative Difference Density on H45        .      -0.58 eA-3
```

Alert level G

```
FORMU01_ALERT_1_G  There is a discrepancy between the atom counts in the
            _chemical_formula_sum and _chemical_formula_moiety. This is
            usually due to the moiety formula being in the wrong format.
```

```
            Atom count from _chemical_formula_sum:   C67.88 H66.49 F2.93 N4 O4
            Atom count from _chemical_formula_moiety:C67.62799 H62.67 F2.93 N4
FORMU01_ALERT_2_G  There is a discrepancy between the atom counts in the
            _chemical_formula_sum and the formula from the _atom_site* data.
            Atom count from _chemical_formula_sum:C67.88 H66.49 F2.93 N4 O4 P1
            Atom count from the _atom_site data:  C67.63149 H62.66941 F2.93 N4
CELLZ01_ALERT_1_G Difference between formula and atom_site contents detected.
CELLZ01_ALERT_1_G ALERT: Large difference may be due to a
            symmetry error - see SYMMG tests
           From the CIF: _cell_formula_units_Z    4
           From the CIF: _chemical_formula_sum  C67.88 H66.49 F2.93 N4 O4 P Rh
           TEST: Compare cell contents of formula and atom_site data

           atom    Z*formula  cif sites diff
           C        271.52    270.53    0.99
           H        265.96    250.68   15.28
           F         11.72     11.72    0.00
           N         16.00     16.00    0.00
           O         16.00     16.00    0.00
           P          4.00      4.00    0.00
           Rh         8.00      8.00   -0.00
           S          4.00      4.00    0.00
PLAT002_ALERT_2_G Number of Distance or Angle Restraints on AtSite         12 Note
PLAT003_ALERT_2_G Number of Uiso or Uij Restrained non-H Atoms ...         12 Report
PLAT045_ALERT_1_G Calculated and Reported Z Differ by a Factor ...      0.250 Check
PLAT083_ALERT_2_G SHELXL Second Parameter in WGHT  Unusually Large      26.98 Why ?
PLAT172_ALERT_4_G The CIF-Embedded .res File Contains DFIX Records          4 Report
PLAT173_ALERT_4_G The CIF-Embedded .res File Contains DANG Records          4 Report
PLAT176_ALERT_4_G The CIF-Embedded .res File Contains SADI Records          8 Report
PLAT178_ALERT_4_G The CIF-Embedded .res File Contains SIMU Records          2 Report
PLAT187_ALERT_4_G The CIF-Embedded .res File Contains RIGU Records          2 Report
PLAT191_ALERT_3_G A Non-default SADI Restraint Value has been used     0.0400 Report
PLAT191_ALERT_3_G A Non-default SADI Restraint Value has been used     0.0400 Report
PLAT191_ALERT_3_G A Non-default SADI Restraint Value has been used     0.0400 Report
PLAT191_ALERT_3_G A Non-default SADI Restraint Value has been used     0.0400 Report
PLAT191_ALERT_3_G A Non-default SADI Restraint Value has been used     0.0400 Report
PLAT230_ALERT_2_G Hirshfeld Test Diff for    C57      --C65      .        5.6 s.u.
PLAT232_ALERT_2_G Hirshfeld Test Diff (M-X)  Rh3      --N3       .       12.0 s.u.
PLAT232_ALERT_2_G Hirshfeld Test Diff (M-X)  Rh3      --N4       .       18.7 s.u.
PLAT232_ALERT_2_G Hirshfeld Test Diff (M-X)  Rh2      --N4       .       15.0 s.u.
PLAT300_ALERT_4_G Atom Site Occupancy of Rh3        Constrained at      0.667 Check
PLAT300_ALERT_4_G Atom Site Occupancy of Rh2        Constrained at     0.3333 Check
PLAT300_ALERT_4_G Atom Site Occupancy of C3         Constrained at     0.6667 Check
PLAT300_ALERT_4_G Atom Site Occupancy of C4         Constrained at     0.6667 Check
PLAT300_ALERT_4_G Atom Site Occupancy of C6         Constrained at     0.6667 Check
PLAT300_ALERT_4_G Atom Site Occupancy of C8         Constrained at     0.6667 Check
PLAT300_ALERT_4_G Atom Site Occupancy of C10        Constrained at     0.6667 Check
PLAT300_ALERT_4_G Atom Site Occupancy of C12        Constrained at     0.6667 Check
PLAT300_ALERT_4_G Atom Site Occupancy of C14        Constrained at     0.6667 Check
PLAT300_ALERT_4_G Atom Site Occupancy of C16        Constrained at     0.6667 Check
PLAT300_ALERT_4_G Atom Site Occupancy of C31        Constrained at      0.667 Check
PLAT300_ALERT_4_G Atom Site Occupancy of C57        Constrained at      0.667 Check
PLAT300_ALERT_4_G Atom Site Occupancy of C61        Constrained at      0.667 Check
PLAT300_ALERT_4_G Atom Site Occupancy of C65        Constrained at      0.667 Check
PLAT300_ALERT_4_G Atom Site Occupancy of C67        Constrained at      0.667 Check
PLAT300_ALERT_4_G Atom Site Occupancy of C83        Constrained at     0.6667 Check
PLAT300_ALERT_4_G Atom Site Occupancy of C1         Constrained at     0.3333 Check
```

```
PLAT300_ALERT_4_G Atom Site Occupancy of C1A        Constrained at     0.3333 Check
PLAT300_ALERT_4_G Atom Site Occupancy of C1B        Constrained at     0.3333 Check
PLAT300_ALERT_4_G Atom Site Occupancy of C1C        Constrained at     0.3333 Check
PLAT300_ALERT_4_G Atom Site Occupancy of C1F        Constrained at     0.3333 Check
PLAT300_ALERT_4_G Atom Site Occupancy of C69        Constrained at     0.3333 Check
PLAT300_ALERT_4_G Atom Site Occupancy of C70        Constrained at     0.3333 Check
PLAT300_ALERT_4_G Atom Site Occupancy of C71        Constrained at     0.3333 Check
PLAT300_ALERT_4_G Atom Site Occupancy of C72        Constrained at     0.3333 Check
PLAT300_ALERT_4_G Atom Site Occupancy of C73        Constrained at     0.3333 Check
PLAT300_ALERT_4_G Atom Site Occupancy of C74        Constrained at     0.3333 Check
PLAT300_ALERT_4_G Atom Site Occupancy of C75        Constrained at     0.3333 Check
PLAT300_ALERT_4_G Atom Site Occupancy of C76        Constrained at     0.3333 Check
PLAT300_ALERT_4_G Atom Site Occupancy of C77        Constrained at     0.3333 Check
PLAT300_ALERT_4_G Atom Site Occupancy of H8A        Constrained at     0.6667 Check
PLAT300_ALERT_4_G Atom Site Occupancy of H8B        Constrained at     0.6667 Check
PLAT300_ALERT_4_G Atom Site Occupancy of H10A       Constrained at     0.6667 Check
PLAT300_ALERT_4_G Atom Site Occupancy of H10B       Constrained at     0.6667 Check
PLAT300_ALERT_4_G Atom Site Occupancy of H12A       Constrained at     0.6667 Check
PLAT300_ALERT_4_G Atom Site Occupancy of H12B       Constrained at     0.6667 Check
PLAT300_ALERT_4_G Atom Site Occupancy of H14A       Constrained at     0.6667 Check
PLAT300_ALERT_4_G Atom Site Occupancy of H14B       Constrained at     0.6667 Check
PLAT300_ALERT_4_G Atom Site Occupancy of H16A       Constrained at     0.6667 Check
PLAT300_ALERT_4_G Atom Site Occupancy of H16B       Constrained at     0.6667 Check
PLAT300_ALERT_4_G Atom Site Occupancy of H31        Constrained at      0.667 Check
PLAT300_ALERT_4_G Atom Site Occupancy of H57A       Constrained at      0.667 Check
PLAT300_ALERT_4_G Atom Site Occupancy of H57B       Constrained at      0.667 Check
PLAT300_ALERT_4_G Atom Site Occupancy of H61A       Constrained at      0.667 Check
PLAT300_ALERT_4_G Atom Site Occupancy of H61B       Constrained at      0.667 Check
PLAT300_ALERT_4_G Atom Site Occupancy of H65A       Constrained at      0.667 Check
PLAT300_ALERT_4_G Atom Site Occupancy of H65B       Constrained at      0.667 Check
PLAT300_ALERT_4_G Atom Site Occupancy of H67A       Constrained at      0.667 Check
PLAT300_ALERT_4_G Atom Site Occupancy of H67B       Constrained at      0.667 Check
PLAT300_ALERT_4_G Atom Site Occupancy of H1AA       Constrained at     0.3333 Check
PLAT300_ALERT_4_G Atom Site Occupancy of H1AB       Constrained at     0.3333 Check
PLAT300_ALERT_4_G Atom Site Occupancy of H1BA       Constrained at     0.3333 Check
PLAT300_ALERT_4_G Atom Site Occupancy of H1BB       Constrained at     0.3333 Check
PLAT300_ALERT_4_G Atom Site Occupancy of H1CA       Constrained at     0.3333 Check
PLAT300_ALERT_4_G Atom Site Occupancy of H1CB       Constrained at     0.3333 Check
PLAT300_ALERT_4_G Atom Site Occupancy of H70        Constrained at     0.3333 Check
PLAT300_ALERT_4_G Atom Site Occupancy of H71A       Constrained at     0.3333 Check
PLAT300_ALERT_4_G Atom Site Occupancy of H71B       Constrained at     0.3333 Check
PLAT300_ALERT_4_G Atom Site Occupancy of H72A       Constrained at     0.3333 Check
PLAT300_ALERT_4_G Atom Site Occupancy of H72B       Constrained at     0.3333 Check
PLAT300_ALERT_4_G Atom Site Occupancy of H73A       Constrained at     0.3333 Check
PLAT300_ALERT_4_G Atom Site Occupancy of H73B       Constrained at     0.3333 Check
PLAT300_ALERT_4_G Atom Site Occupancy of H74A       Constrained at     0.3333 Check
PLAT300_ALERT_4_G Atom Site Occupancy of H74B       Constrained at     0.3333 Check
PLAT300_ALERT_4_G Atom Site Occupancy of H75A       Constrained at     0.3333 Check
PLAT300_ALERT_4_G Atom Site Occupancy of H75B       Constrained at     0.3333 Check
PLAT300_ALERT_4_G Atom Site Occupancy of H77        Constrained at     0.3333 Check
PLAT300_ALERT_4_G Atom Site Occupancy of S1         Constrained at       0.75 Check
PLAT300_ALERT_4_G Atom Site Occupancy of F1         Constrained at       0.75 Check
PLAT300_ALERT_4_G Atom Site Occupancy of F2         Constrained at       0.75 Check
PLAT300_ALERT_4_G Atom Site Occupancy of F3         Constrained at       0.75 Check
PLAT300_ALERT_4_G Atom Site Occupancy of O1         Constrained at       0.75 Check
PLAT300_ALERT_4_G Atom Site Occupancy of O2         Constrained at       0.75 Check
PLAT300_ALERT_4_G Atom Site Occupancy of O4         Constrained at       0.75 Check
```

```
PLAT300_ALERT_4_G Atom Site Occupancy of C51       Constrained at       0.75 Check
PLAT300_ALERT_4_G Atom Site Occupancy of S6        Constrained at       0.25 Check
PLAT300_ALERT_4_G Atom Site Occupancy of F4        Constrained at       0.25 Check
PLAT300_ALERT_4_G Atom Site Occupancy of F5        Constrained at       0.18 Check
PLAT300_ALERT_4_G Atom Site Occupancy of F6        Constrained at       0.25 Check
PLAT300_ALERT_4_G Atom Site Occupancy of O7        Constrained at       0.25 Check
PLAT300_ALERT_4_G Atom Site Occupancy of O8        Constrained at       0.25 Check
PLAT300_ALERT_4_G Atom Site Occupancy of O9        Constrained at       0.25 Check
PLAT300_ALERT_4_G Atom Site Occupancy of C1D       Constrained at       0.13 Check
PLAT301_ALERT_3_G Main Residue  Disorder .............(Resd  1  )        23% Note
PLAT302_ALERT_4_G Anion/Solvent/Minor-Residue Disorder (Resd  2  )       100% Note
PLAT302_ALERT_4_G Anion/Solvent/Minor-Residue Disorder (Resd  3  )       100% Note
PLAT304_ALERT_4_G Non-Integer Number of Atoms in ..... (Resd  1  )     123.42 Check
PLAT304_ALERT_4_G Non-Integer Number of Atoms in ..... (Resd  3  )       1.81 Check
PLAT315_ALERT_2_G Singly Bonded Carbon Detected (H-atoms Missing).        C1F Check
PLAT410_ALERT_2_G Short Intra H...H Contact  H47      ..H70      .       2.11 Ang.
                                                        x,y,z  =      1_555 Check
PLAT413_ALERT_2_G Short Inter XH3 .. XHn     H33A     ..H71B     .       1.95 Ang.
                                                        x,y,z  =      1_555 Check
PLAT432_ALERT_2_G Short Inter X...Y Contact  O7       ..C28      .       3.02 Ang.
                                                x,3/2-y,1/2+z  =      4_576 Check
PLAT432_ALERT_2_G Short Inter X...Y Contact  C33      ..C71      .       3.18 Ang.
                                                        x,y,z  =      1_555 Check
PLAT720_ALERT_4_G Number of Unusual/Non-Standard Labels ..........          6 Note
              H1AA    H1AB    H1BA    H1BB    H1CA    H1CB
PLAT790_ALERT_4_G Centre of Gravity not Within Unit Cell: Resd.  #          4 Note
              C4 H9 O
PLAT811_ALERT_5_G No ADDSYM Analysis: Too Many Excluded Atoms ....          ! Info
PLAT860_ALERT_3_G Number of Least-Squares Restraints ............         164 Note
PLAT910_ALERT_3_G Missing # of FCF Reflection(s) Below Theta(Min).          1 Note
                1  0  0,
PLAT912_ALERT_4_G Missing # of FCF Reflections Above STh/L=  0.600        401 Note
PLAT941_ALERT_3_G Average HKL Measurement Multiplicity ...........        4.1 Low
PLAT978_ALERT_2_G Number C-C Bonds with Positive Residual Density.          0 Info
```

---

   0 **ALERT level A** = Most likely a serious problem - resolve or explain
   1 **ALERT level B** = A potentially serious problem, consider carefully
  28 **ALERT level C** = Check. Ensure it is not caused by an omission or oversight
 123 **ALERT level G** = General information/check it is not something unexpected

```
   8 ALERT type 1 CIF construction/syntax error, inconsistent or missing data
  27 ALERT type 2 Indicator that the structure model may be wrong or deficient
  12 ALERT type 3 Indicator that the structure quality may be low
 104 ALERT type 4 Improvement, methodology, query or suggestion
   1 ALERT type 5 Informative message, check
```

It is advisable to attempt to resolve as many as possible of the alerts in all categories. Often the minor alerts point to easily fixed oversights, errors and omissions in your CIF or refinement strategy, so attention to these fine details can be worthwhile. In order to resolve some of the more serious problems it may be necessary to carry out additional measurements or structure refinements. However, the purpose of your study may justify the reported deviations and the more serious of these should normally be commented upon in the discussion or experimental section of a paper or in the "special_details" fields of the CIF. checkCIF was carefully designed to identify outliers and unusual parameters, but every test has its limitations and alerts that are not important in a particular case may appear. Conversely, the absence of alerts does not guarantee there are no aspects of the results needing attention. It is up to the individual to critically assess their own results and, if necessary, seek expert advice.

Publication of your CIF in IUCr journals

A basic structural check has been run on your CIF. These basic checks will be run on all CIFs submitted for publication in IUCr journals (*Acta Crystallographica*, *Journal of Applied Crystallography*, *Journal of Synchrotron Radiation*); however, if you intend to submit to *Acta Crystallographica Section C* or *E* or *IUCrData*, you should make sure that full publication checks are run on the final version of your CIF prior to submission.

Publication of your CIF in other journals

Please refer to the *Notes for Authors* of the relevant journal for any special instructions relating to CIF submission.

PLATON version of 29/11/2023; check.def file version of 14/09/2023

Datablock cs2_100_1_auto-part1and2,occup0.667and0.333 - ellipsoid plot

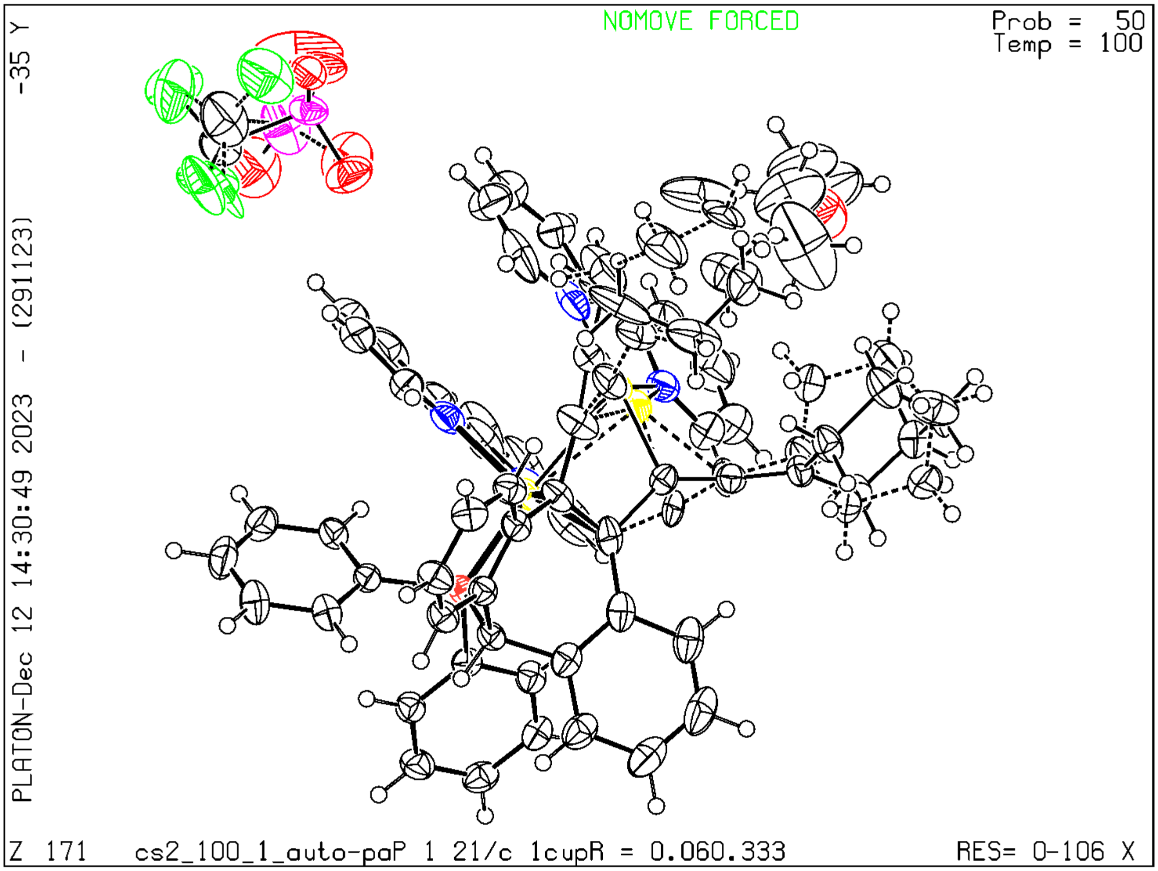

Supplement: Supplementary file 2 — Supporting Information [file ADVS-11-2400072-s001.zip › [8]OTf_Rh2-H_2313512_cifreport.pdf]
